# Regio- and chemoselective Csp^3^–H arylation of benzylamines by single electron transfer/hydrogen atom transfer synergistic catalysis†
†Electronic supplementary information (ESI) available: Experimental procedure, CV data, characterization data of products. See DOI: 10.1039/c8sc02965b


**DOI:** 10.1039/c8sc02965b

**Published:** 2018-09-12

**Authors:** Takafumi Ide, Joshua P. Barham, Masashi Fujita, Yuji Kawato, Hiromichi Egami, Yoshitaka Hamashima

**Affiliations:** a School of Pharmaceutical Sciences , University of Shizuoka , 52-1 Yada, Suruga-ku , Shizuoka 422-8526 , Japan . Email: hamashima@u-shizuoka-ken.ac.jp

## Abstract

We present a highly regio- and chemoselective Csp^3^–H arylation of benzylamines mediated by synergy of single electron transfer (SET) and hydrogen atom transfer (HAT) catalysis. Under well precedented SET catalysis alone, the arylation reaction of *N*,*N*-dimethylbenzylamine proceeded *via* aminium radical cation formation and selectively targeted the *N*-methyl group. In contrast, addition of PhC(O)SH as a HAT catalyst precursor completely switched the regioselectivity to Csp^3^–H arylation at the *N*-benzylic position. Measurement of oxidation potentials indicated that the conjugate base of PhC(O)SH is oxidized in preference to the substrate amine. The discovery of the thiocarboxylate as a novel HAT catalyst allowed for the selective generation of the sulfur-centered radical, so that the *N*-benzyl selectivity was achieved by overriding the inherent *N*-methyl and/or *N*-methylene selectivity under SET catalysis conditions. While visible light-driven α-C–H functionalization of amines has mostly been demonstrated with aniline derivatives and tetrahydroisoquinolines (THIQs), our method is applicable to a variety of primary, secondary and tertiary benzylamines for efficient *N*-benzylic C–H arylation. Functional group tolerance was high, and various 1,1-diarylmethylamines, including an α,α,α-trisubstituted amine, were obtained in good to excellent yield (up to 98%). Importantly, the reaction is applicable to late-stage functionalization of pharmaceuticals.

## Abstract 2

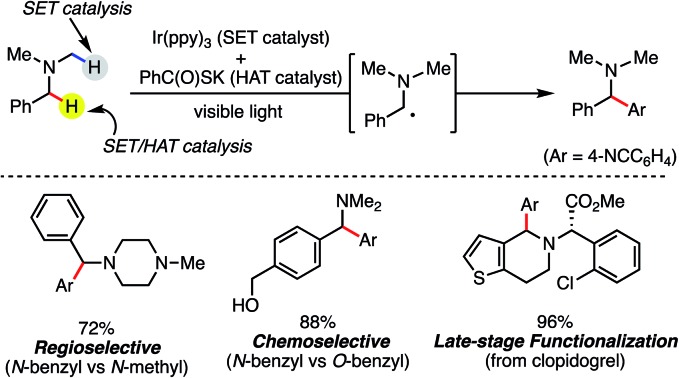
Catalyst controlled regio-, and chemo-selective C-H arylation of benzylamines.

## Introduction

**Fig. 1:**
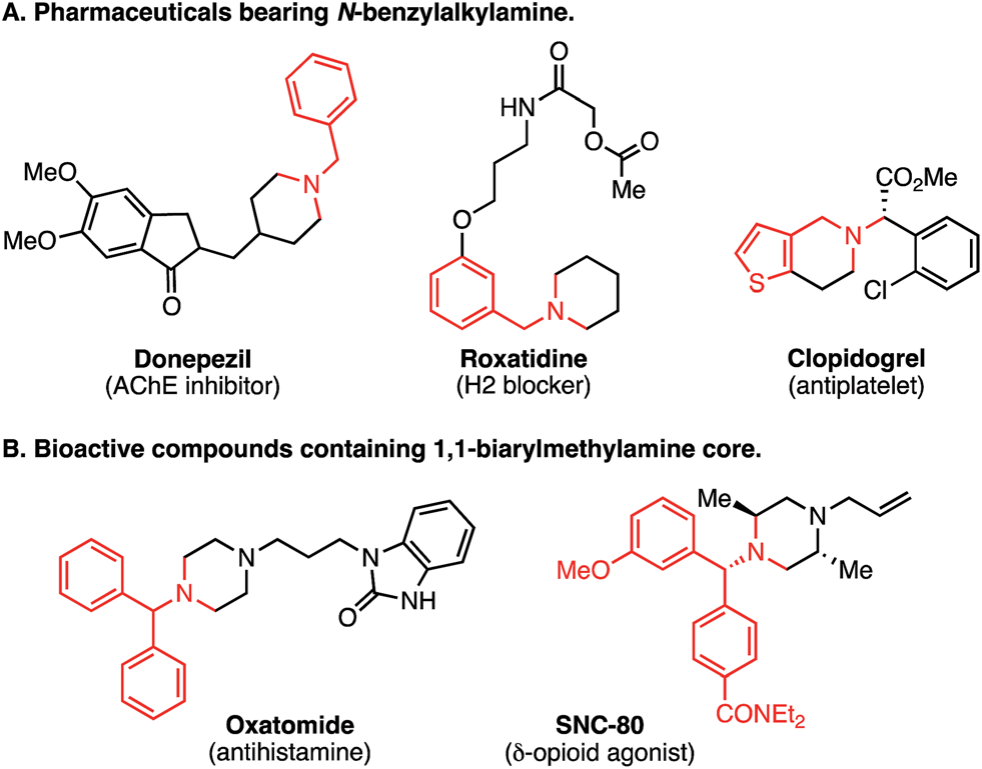
Bioactive compounds containing benzylamines.

The amino group is found in many kinds of molecules, including naturally occurring bioactive compounds, pharmaceutical drugs, agrochemicals, and functional materials. Therefore, direct and selective Csp^3^–H functionalization of amine compounds is extremely useful for rapid derivatization.^1^,^2^ In addition, the benzylamine unit is contained in various synthetic intermediates and is a core structure of many pharmaceuticals (Fig. 1A), as exemplified by donepezil (AChE inhibitor) and roxatidine (H2 blocker). To expedite structure–activity-relationship studies, regio- and chemoselective C–H functionalization of benzylamine derivatives is highly attractive. Moreover, 1,1-diarylmethylamine unit is a well-characterized pharmacophore (Fig. 1B).^3^ Considering the importance of such substructures in medicinal chemistry, mild and efficient methods to construct the 1,1-diarylmethylamine framework are of great interest, and benzylic Csp^3^–H arylation of benzylamines might be a straightforward and expedient approach.

**Scheme 1:**
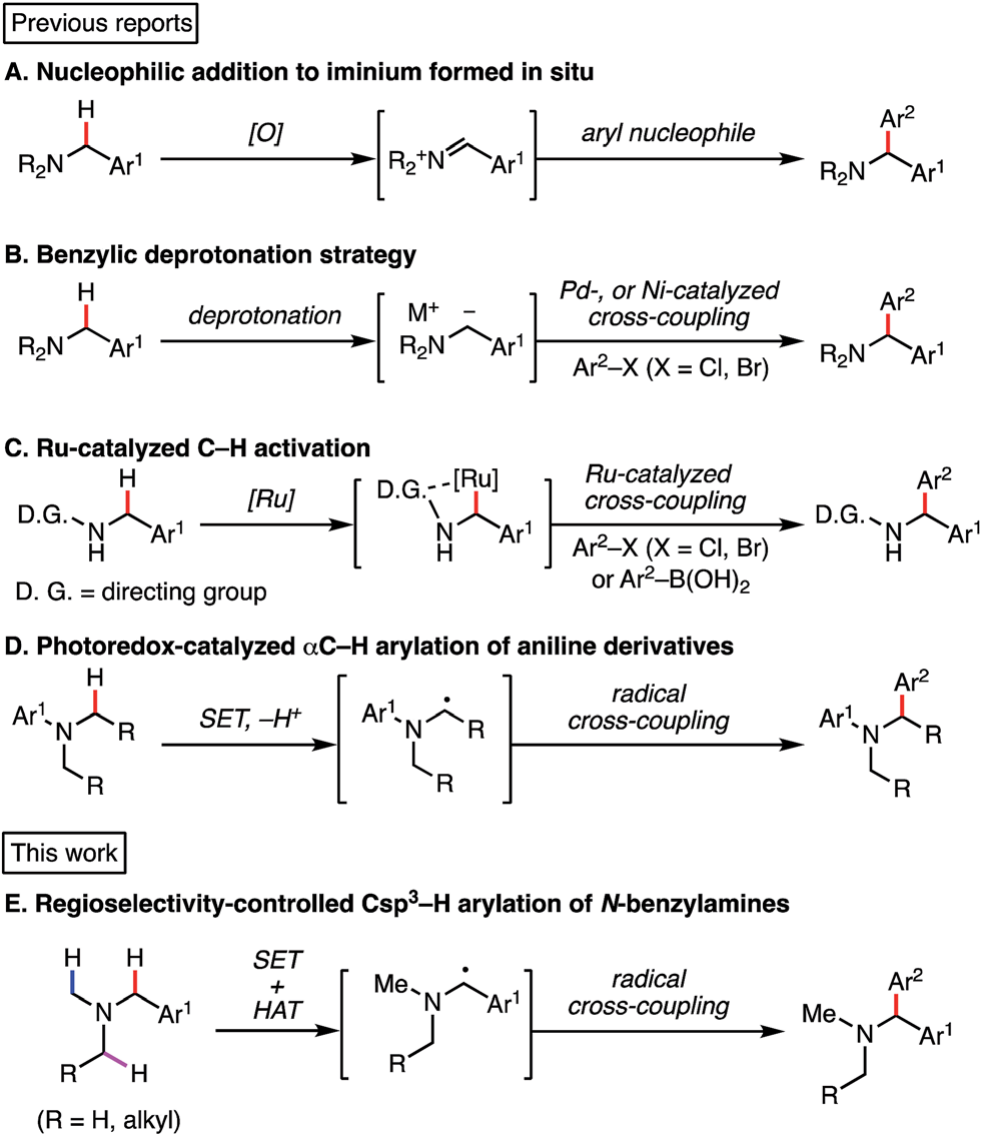
Previous benzylamine arylation strategies and this work.

Nucleophilic addition of an aryl nucleophile to an imine or an iminium ion is a commonly used strategy to construct the 1,1-diarylmethylamine core.^4^ On the other hand, recent work has explored direct benzylic Csp^3^–H arylation of amine derivatives (Scheme 1). Li and co-workers reported an oxidative cross-dehydrogenative coupling reaction of *N*-aryl tetrahydroisoquinolines (THIQs) *via in situ* oxidation to iminium ions (Scheme 1A).^5^ Stephenson's group achieved similar oxidative transformations under photoredox catalysis conditions.^6^ In addition to the oxidative approach, other researchers have employed a protocol involving deprotonation at the *N*-benzylic position, followed by transition-metal-catalyzed cross-coupling reaction (Scheme 1B).^7^ Although a variety of aromatic groups can be incorporated, these approaches normally require harsh reaction conditions, such as strong base and high temperature. Elegant methodologies using transition metal-catalyzed C–H activation have also been disclosed, though in these cases, a directing group is required for the interaction between the transition metal and the benzylic C–H bond (Scheme 1C).^8^

Photoinduced electron transfer has long been examined for C–H functionalization adjacent to a nitrogen atom.^9^,^10^ In 2011, MacMillan and co-workers reported a redox-neutral α-C–H arylation of various aniline derivatives using electron-deficient arenes as arylating partners in the presence of a photoredox catalyst (Scheme 1D).^11^ Following that work, we recently reported a redox-neutral α-C–H alkylation and cyanation of *N*-aryl-THIQs using activated alkyl halides and tosylcyanide.^12^ While visible light photoredox functionalization of amines has been well investigated, the substrate scope is generally limited to THIQ and aniline derivatives. On the other hand, there are some reports on photo-mediated SET oxidation of tertiary alkyl amines (*e.g.*, Et_3_N and ^i^Pr_2_NEt) though they are used as sacrificial reducing agents.^13^,^14^ As for benzylic C–H arylation, THIQ derivatives have been investigated actively as benchmark substrates.6e,^15^,^16^ It should be noted that THIQs are special compounds in terms of both the oxidation potential and the bond dissociation energy (*vide infra*), indicating that simple *N*-alkyl benzylamines should be considered different from standard THIQs. Therefore, selective benzylic transformation of simple alkylamines is still a challenging subject. To our knowledge, there is no general and selective method available for *N*-benzylic C–H arylation. Herein, we report a regio- and chemoselective C–H functionalization of benzylamines *via* redox-neutral SET and HAT synergistic catalysis (Scheme 1E). The distinct features of the present reaction are listed below: (1) in addition to well-studied aniline-type compounds, various unprotected *N*-alkyl benzylamines are available in this reaction. (2) Since PhC(O)SH is easily deprotonated and converted to the corresponding sulfur-centered radical, single electron oxidation of the amine substrates is blocked effectively. SET/HAT synergistic catalysis enables excellent *N*-benzyl selectivity in preference to inherent *N*-methyl and *N*-methylene selectivity observed under SET catalysis conditions. (3) Due to the efficient generation of the HAT catalyst, as little as 1 mol% of PhC(O)SH is sufficient. In addition, an excess amount of starting amine is not required in our reaction. (4) The chemoselectivity is high, and the late-stage functionalization of pharmaceutical drugs was demonstrated successfully.

## Results and discussion

**Scheme 2:**
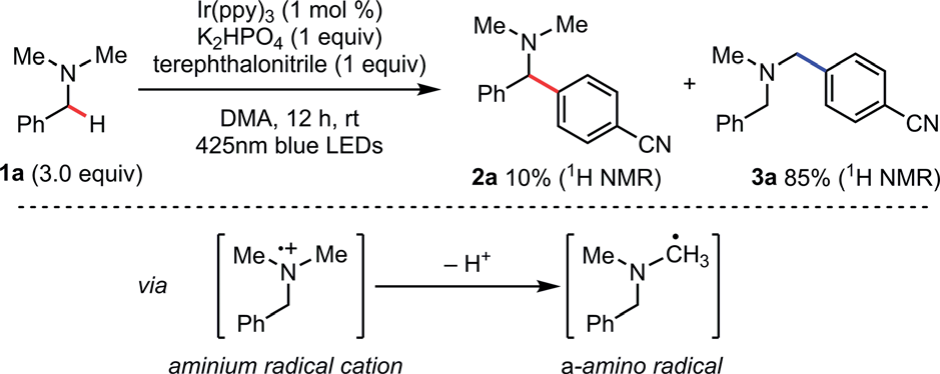
Csp^3^–H arylation of *N*,*N*-dimethylbenzylamine under photoredox catalysis.

According to the reported reaction conditions of photoredox catalysis *via* an aminium radical cation intermediate,^11^ we began with the reaction of *N*,*N*-dimethylbenzylamine (**1a**) with terephthalonitrile expecting that the most stable (benzylic and 2°) α-amino radical would be generated (Scheme 2). With Ir(ppy)_3_ (1 mol%) as a photoredox catalyst and K_2_HPO_4_ as base, the reaction afforded two compounds, **2a** and **3a**, in excellent mass balance (95%), though a long reaction time (12 h) was required for completion. Contrary to our expectation, the major product was not **2a** but **3a**, which is derived from the thermodynamically less stable non-benzylic and 1° α-amino radical.^17^

While the origin of this regioselectivity is unclear at this point, we think that the kinetic acidity in terms of stereoelectronic and steric factors are crucial. Thus, deprotonation of aminium radical cations to give α-amino radicals might be accelerated by the overlap of the breaking Csp^3^–H bond orbital with the SOMO orbital on the aminium radical cation.^18^,^19^ These results suggested that well-precedented SET catalysis is not applicable and distinct reaction conditions should be devised for achieving the targeted *N*-benzylic transformation.

**Scheme 3:**
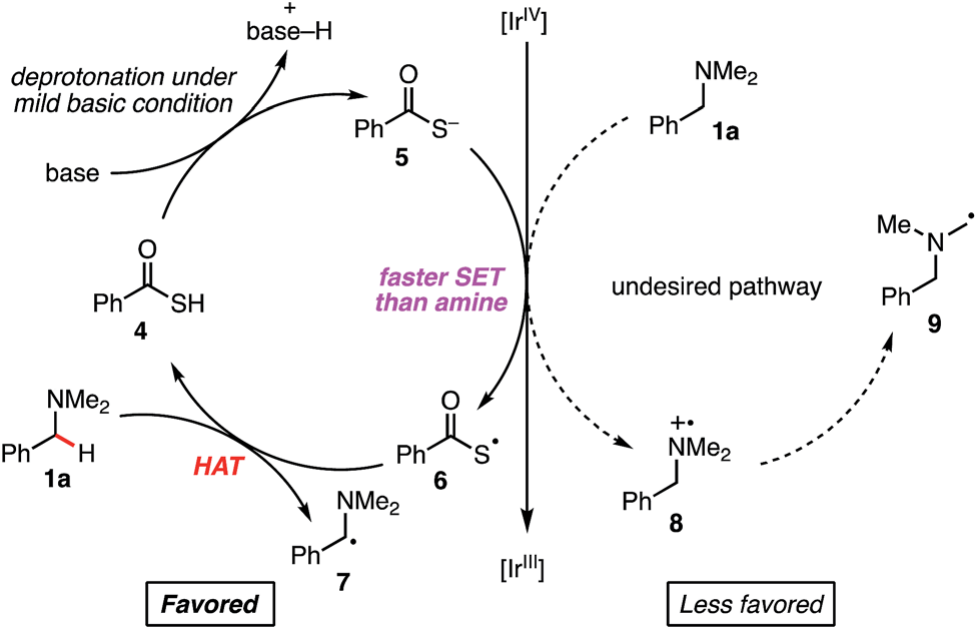
Working hypothesis for regioselective arylation at the *N*-benzylic position.

We considered that a HAT strategy might alter the regioselectivity (Scheme 3). A significant determinant of the selectivity of a HAT process is the C–H bond dissociation energy (BDE). Since BDE is directly associated with the stability of the radical products, a large difference between *N*-methyl C–H and *N*-benzyl C–H BDEs should be anticipated. According to the previous studies,^20^,^21^ we expected that sulfur-centered radicals would be HAT agents of choice for selective activation of the *N*-benzyl group. Importantly, however, in order to avoid direct single electron oxidation of *N*-benzylamine **1a** leading to the SET catalysis pathway (Scheme 3, right), the sulfur-centered radical precursor should have a lower anodic peak potential than that of *N*-benzylamine **1a**. For this purpose, we envisaged that the anion of a sulfur atom would undergo more facile oxidation than a typical thiol such as cysteine (*E*_1/2_ = +0.85 V *vs.* SCE for cysteine in CH_3_CN, p*K*_a_ = 9.35 for cysteine methyl ester)^22^ and that a more acidic sulfur compound would be more favorable. Therefore, we selected thiocarboxylic acid **4**, whose conjugate base could be generated with a weak base (p*K*_a_ = 3.2 for thioacetic acid).^23^ The negatively charged, electron-rich thiocarboxylate species **5** might undergo SET oxidation much faster than the substrate amine, thereby affording the sulfur-centered radical **6** preferentially (Scheme 3, left). This scenario would lead to regioselective C–H activation at the *N*-benzylic position by overriding the natural *N*-methyl selectivity observed in Scheme 2.

**Fig. 2:**
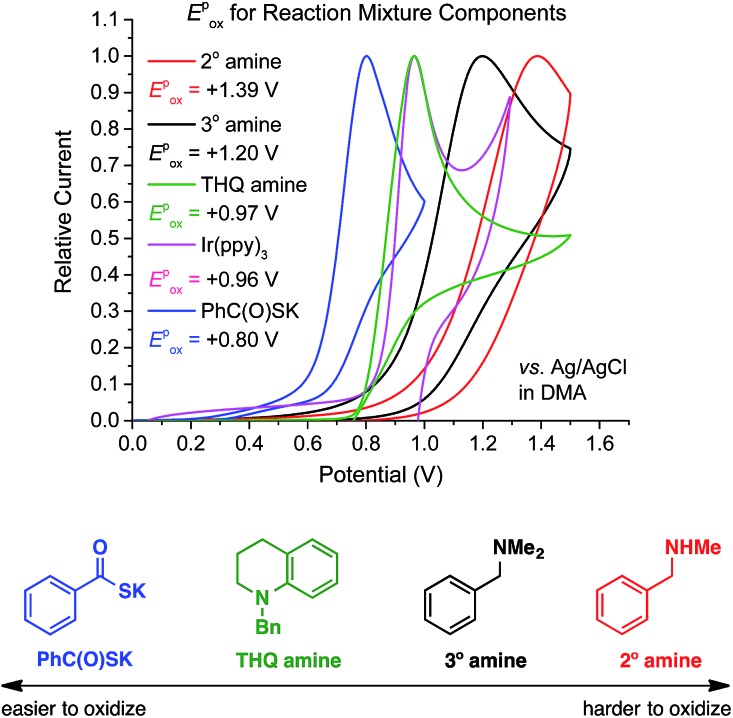
Comparison of oxidation peak potentials measured by cyclic voltammetry.

To validate our working hypothesis, we measured the oxidation potentials of potassium thiobenzoate (PhC(O)SK) and three *N*-benzylamines by cyclic voltammetry (CV) in DMA (Fig. 2; see ESI† for potentials *vs.* SCE and further CV studies). Gratifyingly, PhC(O)SK was found to have a less positive anodic peak potential (*E*pox (**6**/**5**) = +0.80 V *vs.* Ag/AgCl in DMA) than all the amine compounds examined, even when compared to the aniline-type *N*-benzyl tetrahydroquinoline (THQ) (*E*pox = +0.97 V *vs.* Ag/AgCl in DMA). Therefore, thermodynamics predicted that selective oxidation of the thiobenzoate anion would occur even in the presence of the reducing amine substrates. Furthermore, CV revealed that oxidation of PhC(O)SK by [Ir^IV^(ppy)_3_]^+^ (*E*_1/2_ (Ir^IV^/Ir^III^) = +0.96 V *vs.* Ag/AgCl in DMA) was exergonic, while oxidation of the amines was always endergonic. According to these data, a faster reaction profile is expected compared to the reaction shown in Scheme 2.

With the promising CV results in mind, we revisited benzylic C–H arylation of *N*,*N*-dimethylbenzylamine (**1a**) in the presence of PhC(O)SH (**4**) and other HAT catalyst precursors (Table 1). The arylation proceeded in 91% yield at the benzylic position with excellent regioselectivity (r.r = >20 : 1, entry 2), which is consistent with the idea of a mechanistic switchover from SET catalysis to SET/HAT synergistic catalysis. It should be noted that the reaction reached completion within 1 h (entry 3), compared to 12 h required when PhC(O)SH was absent (entry 1). This rate-acceleration might be due to exergonic SET oxidation of the thiocarboxylate and a favorable difference in BDE between PhC(O)S–H (87.4 kcal mol^–1^)^24^ and the targeted *N*-benzylic C–H bond (84.9 kcal mol^–1^).^24^ Precatalysts **10** and **11** gave **2a** with excellent *N*-benzyl selectivity. However, the yields were much lower even in the presence of 20 mol% of HAT catalysts (entries 4, 5). We assume that these less acidic thiols are not deprotonated under the reaction conditions, and thus SET oxidation might not be efficient compared to the case of PhC(O)SK (*E*_1/2_ = +0.85 V *vs.* SCE for cysteine in CH_3_CN,^22^*E*pox (**6**/**5**) = +0.65 V *vs.* SCE in CH_3_CN; see ESI†). Considering the p*K*_a_ values of the HAT precatalysts, **12** was also examined as a more acidic thiol (entry 6). However, almost no reaction was observed, probably because the generated thiyl radical would be less reactive in the subsequent HAT process (S–H BDE of TolSH = 77–83 kcal mol^–1^)^20^ (entry 4). Recently, quinuclidine and DABCO aminium radical cations have been reported to perform HAT with alkylamines at the α position to the nitrogen.^25^ Given its accessible oxidation potential (*E*pox = +0.69 V *vs.* SCE in CH_3_CN),25*b* DABCO would undergo SET oxidation selectively over **1a**. However, when PhC(O)SK was replaced with DABCO, the reaction did not proceed (entry 7). The reason is not clear at present, but back electron transfer processes such as quenching of the DABCO radical cation by arene radical anion might be sufficiently rapid to interfere with the HAT process.^26^ When PhC(O)SK was replaced with quinuclidine, the product was formed in only very low yield with low regioselectivity (entry 8), probably because the strong reactivity of quinuclidine radical cation (N^+^–H BDE of quinuclidine = 100 kcal mol^–1^)^27^ cannot effectively differentiate between the C–H bonds at the *N*-methyl and *N*-benzyl positions. These results strongly indicate the superiority of PhC(O)SK for the selective *N*-benzyl arylation over other HAT catalysts.

**Table 1:** Screening of conditions for benzylic Csp^3^–H arylation of *N*,*N*-dimethylbenzylamine^*a*^

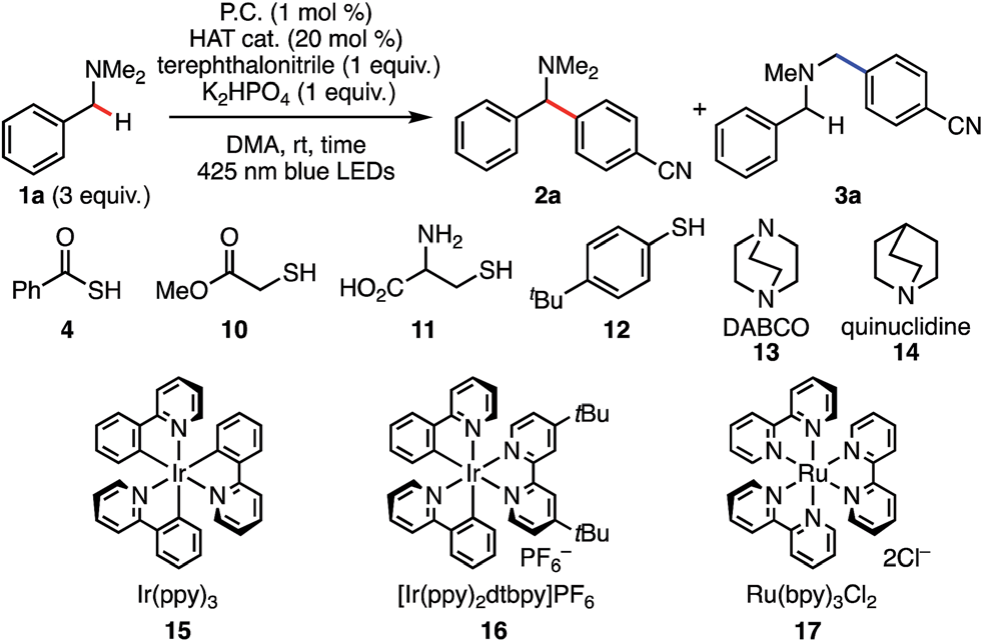					
Entry	HAT cat.	P.C.	Time (h)	Yield^*b*^ (%)	**2a** : **3a**^*b*^
1^*c*^	—	**15**	12	95	1 : 8.5
2	**4**	**15**	12	91	>20 : 1
3	**4**	**15**	1	93	>20 : 1
4	**10**	**15**	1	23	>20 : 1
5	**11**	**15**	1	43	>20 : 1
6	**12**	**15**	1	Trace	—
7	**13**	**15**	1	Trace	—
8	**14**	**15**	1	12	1 : 2
9	**4**	**16**	1	42	>20 : 1
10	**4**	**17**	1	No reaction	
11^*d*^ ^,^^*e*^	**4**	**15**	2	90 (87)^*f*^	>20 : 1
12^*d*^ ^,^^*g*^	**4**	**15**	2	72	>20 : 1

^*a*^The reactions were run on 0.2 mmol scale.

^*b*^Yield and regioisomeric ratio were determined by ^1^H NMR analysis using 1,1,2,2-tetrachloroethane as an internal standard.

^*c*^Data from Scheme 3.

^*d*^1 mol% of PhC(O)SH and 0.5 mol% of Ir(ppy)_3_ were used on a 1 mmol scale.

^*e*^2 equiv. of *N*,*N*-dimethylbenzylamine was used.

^*f*^Isolated yield.

^*g*^1 equiv. of *N*,*N*-dimethylbenzylamine was used. P.C.: photocatalyst.

We also examined other photoredox catalysts. When [Ir(ppy)_2_dtbbpy]PF_6_ was used, the product was obtained in a lower yield (entry 9). In this case, reductive quenching of [Ir^III^]* (*E*_1/2_ (*Ir^III^/Ir^II^) = +0.66 V *vs.* SCE in CH_3_CN)^28^ from the thiocarboxylate (*E*pox = +0.65 V *vs.* SCE in CH_3_CN) is plausible. Since the subsequent SET process between [Ir^II^] (*E*_1/2_ (Ir^III^/Ir^II^) = –1.51 V *vs.* SCE in CH_3_CN)^28^ and terephthalonitrile (*E*red1/2 = –1.61 V *vs.* SCE in CH_3_CN)^29^ is thermodynamically disfavored, the reaction might be retarded. On the other hand, the reaction did not proceed with Ru(bpy)_3_Cl_2_ (entry 10). Considering the redox potential after the favorable reductive quenching of [Ru^II^]* (*E*_1/2_ (*Ru^II^/Ru^I^) = +0.77 V *vs.* SCE in CH_3_CN) by PhC(O)SK, SET from [Ru^I^] (*E*_1/2_ (Ru^II^/Ru^I^) = –1.33 V *vs.* SCE in CH_3_CN)^30^ to terephthalonitrile (*E*red1/2 = –1.61 V *vs.* SCE in CH_3_CN)^29^ is energetically unfavorable. Gratifyingly, we succeeded in decreasing the amount of the catalysts. When we used 0.5 mol% of Ir(ppy)_3_ and as little as 1 mol% of PhC(O)SH, the reaction proceeded without difficulty to give **2a** in 90% yield (entry 11). In contrast, the reaction using 1 mol% of **11** resulted in lower yield and regioselectivity (31% yield, benzyl/Me = 5.2 : 1). Moreover, a stoichiometric amount of **1a** was sufficient to produce **2a** in good yield (entry 12), in contrast to most of the preceding examples in which a large excess amount of amine substrate (at least three equivalents of starting amines) was generally needed to achieve good yields. A control experiment confirmed that the reaction does not occur in the dark (see ESI†).

**Scheme 4:**
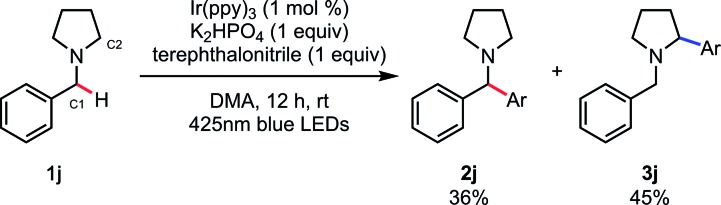
Arylation of **1j** under SET conditions.

With the optimal reaction conditions identified (Table 1, entry 11), we turned our attention to the substrate scope of the SET/HAT synergistic catalysis for Csp^3^–H arylation of *N*-benzylamines (Table 2). *N*,*N*-Dimethylbenzylamine derivatives with varying electronic properties were well tolerated, and the desired products (**2b–f**) were obtained in good yields. Electron-rich derivatives were the highest-yielding, which is consistent with the polarity effects.^31^ However, *para*-ester-substituent retarded the reaction and the corresponding arylated product was not obtained, suggesting that strongly electron-withdrawing groups are not suitable for this reaction. Compared to the *para*-methyl-substituted derivative (**2f**), the *ortho*-methyl-substituted derivative gave **2g** in a lower yield (75%), probably due to the steric interaction. Similarly, the reaction of the starting benzylamine was much faster than that of the dibenzylamine, so that over-reaction of the products was not observed. Nevertheless, we found that treating sterically congested **1h** under the reaction conditions successfully afforded α,α,α-trisubstituted amine **2h** in good yield (74%). Different substituted groups on the nitrogen atom did not prohibit the reaction, since ethyl groups (**2i**), aliphatic cyclic substituents (**2j**, **2k**), and heteroatom-bearing cyclic substituents (**2l**, **2m**) performed equally well under the reaction conditions. *N*-Methyl THIQ provided **2n** in 94% yield as an expected regioisomer.^32^ Notably, secondary and primary amines having no protective group were also applicable, affording **2o** and **2p** in 98% and 98% yield, respectively. These are relatively difficult substrates for SET catalysis due to their more positive anodic peak potentials, and so have been less well studied in the literature. Glycine derivative **1q** underwent selective *N*-benzyl functionalization to afford **2q** in 97% yield. Moreover, heterocyclic aromatic derivatives were tolerated, providing the corresponding arylated products **2r–2t**. Aside from terephthalonitrile, 4-cyanopyridine derivatives and 1-cyanoisoquinoline reacted in a regioselective manner, providing **2u–2y** in good to excellent yields. When cyanobenzenes bearing different *para*-electron-withdrawing groups were examined, the reaction proceeded with excellent regioselectivity, though in less satisfactory yields (**2z**). For a comparative example, the reaction of **1j** without PhC(O)SH was carried out, and a mixture of the C1-arylated **2j** (36%) and the C2-arylated **3j** (45%) was obtained after 12 h (Scheme 4). These results clearly indicate that the excellent *N*-benzyl selectivity observed in our reaction is attributed to SET/HAT synergistic catalysis that can outcompete the inherent *N*-methyl and/or *N*-methylene selectivity observed under SET catalysis conditions.

**Table 2:** Substrate scope for benzylic Csp^3^–H arylation of *N*-benzylamines^*a*^

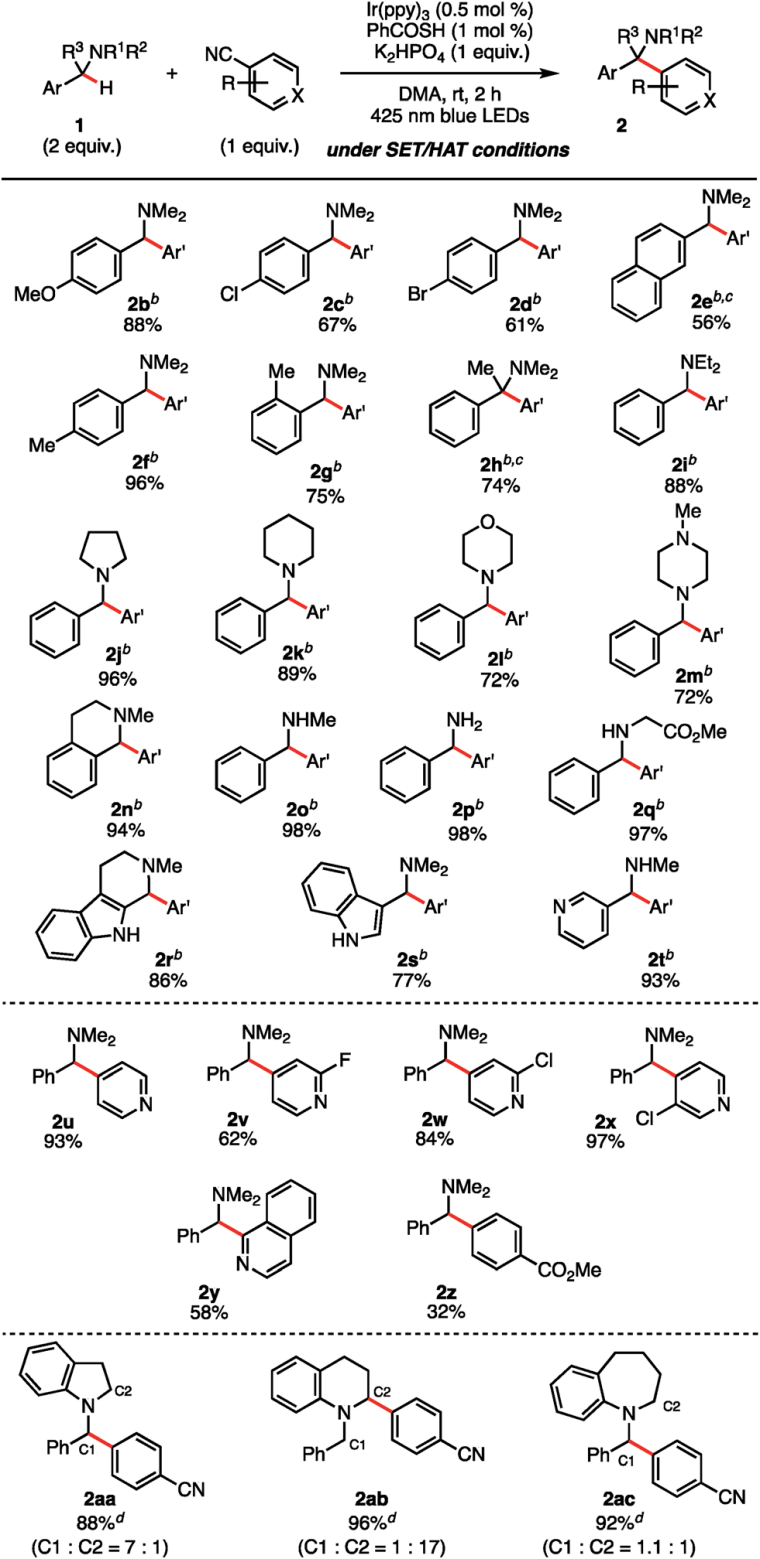

^*a*^All reactions were conducted on 1 mmol scale.

^*b*^Terephthalonitrile was used as an arylating reagent (Ar′ = 4-NCC_6_H_4_).

^*c*^The reaction was carried out for 6 h.

^*d*^Yield and regioisomeric ratio were determined by ^1^H NMR analysis using 1,1,2,2-tetrachloroethane as an internal standard.

An interesting phenomenon was observed when comparing cyclic *N*-benzylaniline-type substrates. Treatment of *N*-benzyl indoline (**1aa**) under our reaction conditions resulted mainly in *N*-benzyl arylation. Nonetheless, under photocatalyst-mediated SET catalysis alone, the regioselectivity was inverted completely from the *N*-benzyl position to the cyclic *N*-methylene position (90% yield, C1/C2 = 1 : 6.7). In contrast, *N*-benzyltetrahydroquinoline (**1ab**) reacted under our conditions to give the cyclic *N*-methylene-arylated product **2ab** with high regioselectivity (C1/C2 = 1 : 17). We postulate that the increase in steric repulsion around the *N*-benzylic position in moving from the 5- to the 6-membered ring system may be responsible. However, on the other hand, *N*-benzyltetrahydrobenzoazepine (**1ac**) gave a 1.1 : 1 mixture of the C1/C2 regioisomers. The origin of the regioselectivity remains unclear at this point, but we assume that the conformational flexibility within the nitrogen atom-containing ring is important to achieve orbital overlap of the *n*-orbital on the nitrogen atom with the σ* orbital of the breaking C–H bond, which should have an impact on the BDE.

**Scheme 5:**
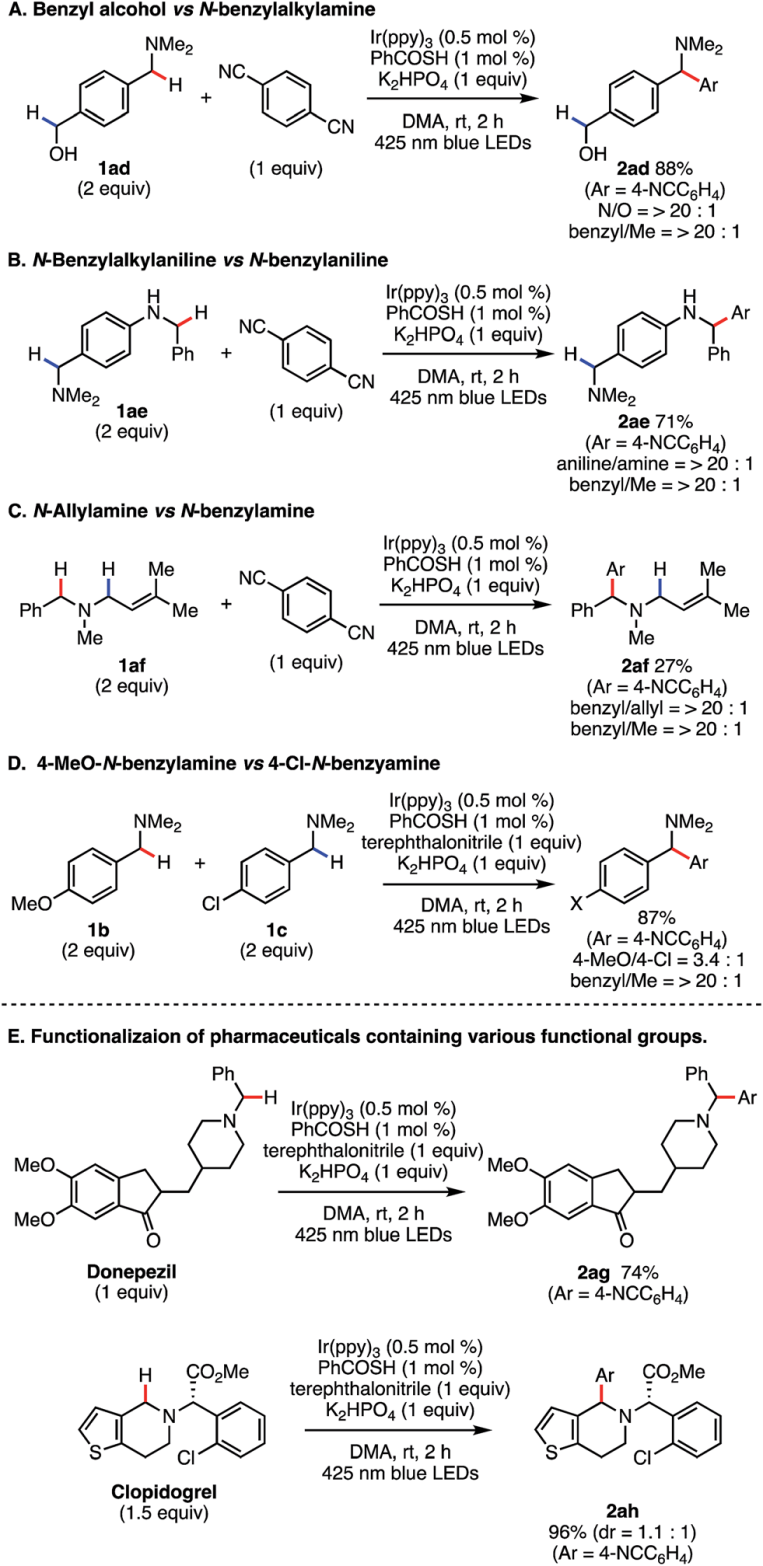
Regio- and chemoselective Csp^3^–H arylation.

Next, we evaluated the chemoselectivity of our reaction in more detail. For substrate **1ad**, exclusive *N*-benzyl functionalization occurred in the presence of the benzylic alcohol (Scheme 5A), reflecting the difference in C–H BDEs between the benzylic alcohol side (C–H BDE of benzyl alcohol = 87.5 kcal mol^–1^)^24^ and the *N*-benzyl group (C–H BDE of *N*,*N*-dimethylbenzylamine = 84.9 kcal mol^–1^).^24^ An intramolecular competitive experiment with **1ae** targeted the *N*-benzylaniline side to produce **2ae** in 71% yield (Scheme 5B). We next examined the reaction of a more challenging substrate (Scheme 5C). The C–H arylation reaction of **1af** occurred only at the *N*-benzylic position, even though the BDEs of both *N*-benzylic C–H bond and *N*-allylic C–H bond are reported to be close (*ca.* 85 kcal mol^–1^ and 83 kcal mol^–1^, respectively).^24^ The yield was only moderate due to competitive reactions at the C–C double bond, but no arylation product at the *N*-allylic position was detected in this reaction. Moreover, we conducted an intermolecular competition experiment using **1b** and **1c** (1 : 1). In this case, the reaction mainly proceeded with **1b** bearing an electron-donating group, which is indicative of a polarity matching effect (Scheme 5D).^31^

To confirm the synthetic utility of our SET/HAT synergistic catalysis, we evaluated its efficiency in late-stage functionalization. When treated in a 1 : 1 ratio, donepezil and **7** underwent regio- and chemoselective coupling reaction smoothly to give 1,1-diarylmethylamine **2ag** in good yield (74%). Further, even with more complex clopidogrel, C–H arylation at the endocyclic *N*-benzylic position proceeded regioselectively, furnishing **2ah** in 96% yield. These examples clearly demonstrate that the present reaction is applicable to late-stage derivatization of structurally complex bioactive compounds, and thus should be useful to facilitate structure–activity-relationship studies.

All reactions were conducted on 1 mmol scale and isolated yields are shown. Chemo- and regioselectivity were determined by ^1^H NMR analysis.

**Scheme 6:**
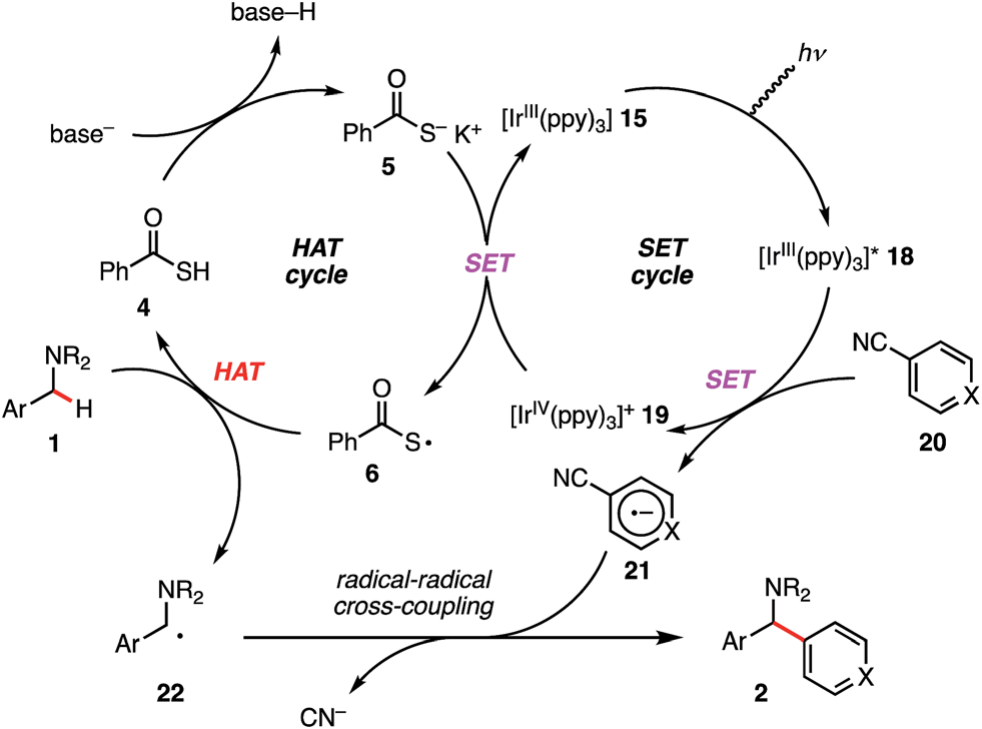
Proposed catalytic cycle.

Finally, on the basis of the observed selectivity and CV measurements, we propose the catalytic cycle shown in Scheme 6. *fac*-Tris(2-phenylpyridinato)iridium(iii) [Ir^III^(ppy)_3_] (**15**) is excited by photo-irradiation (blue LED, 425 nm) and [Ir^III^(ppy)_3_]* (**18**) is generated. This reductant [Ir(ppy)_3_]* (**18**) (*E*_1/2_ (Ir^IV^/*Ir^III^) = –1.73 V *vs.* SCE in CH_3_CN)^33^ undergoes SET to electron-deficient arene **20** to afford radical anion intermediate **21** along with [Ir^IV^(ppy)_3_]^+^ (**19**). Subsequently, [Ir^IV^(ppy)_3_]^+^ (**19**) (*E*_1/2_ (Ir^IV^/Ir^III^) = +0.77 V *vs.* SCE in CH_3_CN)^33^ performs SET oxidation of the electron-rich PhC(O)SK (*E*pox (**6**/**5**) = +0.65 V *vs.* SCE in CH_3_CN, see ESI†), which outcompetes SET oxidation of the amine substrate leading to the different reaction pathway. The generated sulfur-centered radical **6** regioselectively undergoes HAT regioselectively depending on C–H BDEs. As seen in the previous reports, the resulting *N*-benzyl radical **22** reacts with the separately formed radical anion intermediate **21** to yield the desired 1,1-diarylmethylamine **2**.^34^

## Conclusions

In conclusion, we have developed a highly regio- and chemoselective Csp^3^–H arylation of a variety of benzylamine derivatives by employing SET/HAT synergistic catalysis. Under SET catalysis alone, Csp^3^–H arylation of benzylamines proceeded at the *N*-methyl and/or cyclic *N*-methylene positions through an aminium radical cation intermediate. In contrast, synergistic SET and HAT catalysis inverts the regioselectivity. The reaction was completed within 2 h in the presence of as little as 0.5 mol% of the Ir complex and 1 mol% of PhC(O)SH as a HAT catalyst, and various 1,1-diarylmethylamines were obtained in good to excellent yields (56–98%). Importantly, high yields were achieved even when a stoichiometric amount of the benzylamine substrate was employed. From a mechanistic viewpoint, the use of PhC(O)SH as a HAT catalyst precursor is the key to success: SET oxidation of the 3° amine substrates was suppressed effectively due to the favorable oxidation of PhC(O)SK, and the resulting sulfur radical abstracts C–H bonds selectively to give *N*-benzyl radicals. The excellent regio- and chemoselectivity enables the late-stage *N*-benzyl arylation of pharmaceuticals with reactive functional groups. Further studies including *N*-methyl selective functionalization are underway in our laboratory.

## Conflicts of interest

There are no conflicts to declare.

## Supplementary Material

Supplementary information
