# Supplementary material for: Regio- and chemoselective Csp3–H arylation of benzylamines by single electron transfer/hydrogen atom transfer synergistic catalysis
Source: Chem Sci. 2018 Sep 12;9(44):8453–60. doi: 10.1039/c8sc02965b (PMC6244453; doi:10.1039/c8sc02965b)
Supplement: Supplementary file 1 [file SC-009-C8SC02965B-s001.pdf]

Electronic Supplementary Material (ESI) for Chemical Science.
This journal is © The Royal Society of Chemistry 2018

Electronic Supplementary Information

Regio- and chemoselective Csp^3–H arylation of benzylamines by single electron transfer/hydrogen atom transfer synergistic catalysis

Takafumi Ide, Joshua P. Barham, Masashi Fujita, Yuji Kawato, Hiromichi Egami, and Yoshitaka Hamashima*

School of Pharmaceutical Sciences, University of Shizuoka, 52-1 Yada, Suruga-ku, Shizuoka 422-8526, Japan

hamashima@u-shizuoka-ken.ac.jp

Table of Contents

1. General information
2. Preparation of amine substrates
3. C–H arylation with Ir complex under photo-irradiation
4. Cyclic voltammetry
5. Screening of the reaction conditions for benzylic C–H arylation
6. Benzylic C–H arylation under SET-HAT synergistic catalysis
7. Limitation regarding electron-deficient aromatic compounds
8. References
9. NMR spectra

1. General information

^{1}H NMR spectra were measured on a JEOL JNM-ECA-500 spectrometer at 500 MHz. ^{13}C NMR spectra were recorded on a JEOL JNM-ECA-500 spectrometer at 125 MHz. Chemical shifts were reported in parts per million (ppm) downfield from TMS (= 0) or $CDCl_3$ for ^{1}H NMR. For ^{13}C NMR, chemical shifts were reported in the scale relative to $CDCl_3$. Infrared spectra were measured on a SHIMADZU IRPrestige-21 and only diagnostic absorptions are listed below. ESI-MS data were taken on a Thermo SCIENTIFIC ACCELA Exactive liquid chromatography–mass spectrometer (LC–MS). Column chromatography was performed with silica gel N-60 (40-100 μm) purchased from Kanto Chemical Co., Inc. TLC analysis was performed on Silica gel 60 F254-coated glass plates (Merck). Visualization was accomplished by means of ultraviolet (UV) irradiation at 254 nm or by spraying 12-molybdo(VI)phosphoric acid ethanol solution as a developing agent. GPC purification was conducted on a Shimadzu recycling preparative HPLC system [LC-20AR; column, YMC-GPC T-2000; chloroform]. Blue light irradiation was performed with a RelyOn LED lamp (3 W, λ = 425 nm).

Dehydrated *N*,*N*-dimethylacetamide (DMA), *N*,*N*-dimethylformamide (DMF), acetonitrile (CH_3CN), acetone and tetrahydrofuran (THF) were purchased from Kanto Chemical Co. Dehydrated dichloromethane (CH_2Cl_2) and diethyl ether (Et_2O) were purchased from Wako Pure Chemical Industries, Ltd. Photoredox catalysts were purchased from Sigma-Aldrich. Benzylamine substrates were prepared according to the literatures.[1-3] Thiobenzoic acid was purchased from Tokyo Chemical Industry Co., Ltd. (TCI). Other reagents were purified by usual methods.

2. Preparation of amine substrates

2.1. Synthesis of (4-((dimethylamino)methyl)phenyl)methanol (1ad)

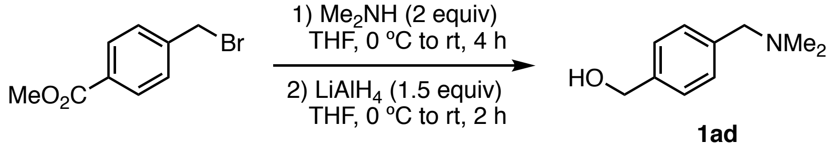

1ad
(88% in 2 steps)

To a solution of methyl 4-(bromomethyl)benzoate (2.0 g, 8.7 mmol) in THF (50 mL) was added Me_2NH (2 M in THF, 8.7 mL, 17.4 mmol) at 0 °C. The reaction mixture was stirred for 4 h at ambient temperature, and then H_2O was added to the mixture. The organic material was extracted with EtOAc (15 mL × 2). The combined organic layers were washed with brine and dried over $MgSO_4$. After filtration, the filtrate was concentrated *in vacuo* and the crude mixture was used without further purification in the next step.

To a solution of $LiAlH_4$ (495 mg, 13.1 mmol) in THF (30 mL) was added a solution of the crude mixture in THF (10 mL) at 0 °C. The resultant mixture was stirred for 2 h. The reaction was quenched with H_2O (0.5 mL), and 1 N NaOH aq. (0.5 mL) and H_2O (1.5 mL) were carefully added to the mixture. After filtration, the filtrate was concentrated *in vacuo* and the residue was purified by column chromatography on silica gel (*n*-hexane/EtOAc = 5/1) to provide **1ad** (1.27 g, 88% yield in 2 steps) as a colorless oil.

1ad: ^{1}H NMR (500 MHz, $CDCl_3$): δ = 7.33 (d, J = 8.0 Hz, 2H), 7.30 (d, J = 8.0 Hz, 2H), 4.69 (s, 2H), 3.42 (s, 2H), 2.23 (s, 6H); ^{13}C NMR (125 MHz, $CDCl_3$): δ = 140.3, 137.1, 129.3, 126.8, 64.5, 63.8, 45.0; HRMS (ESI$^+$): Calcd. for $[C_{10}H_{15}NO+H]^+$: m/z = 166.1226, Found: 166.1225.

2.2. Synthesis of *N*-benzyl-4-((dimethylamino)methyl)aniline (1ae)

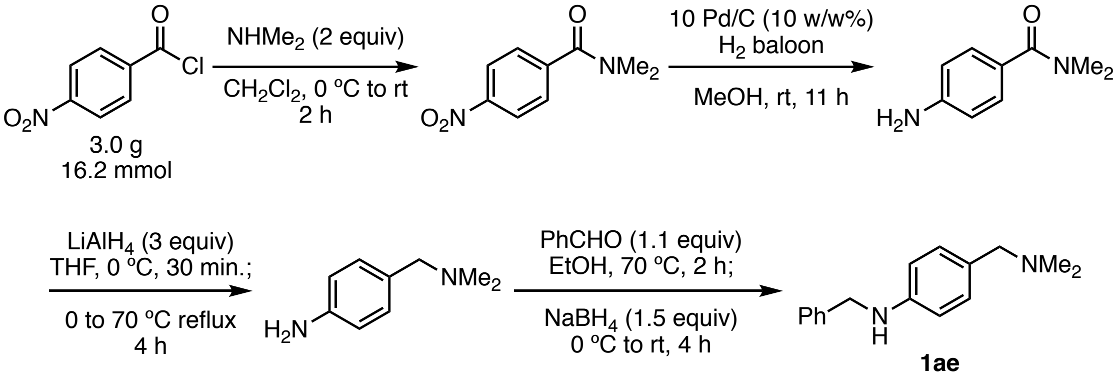

1ae
43% in 4 steps

To a solution of 4-nitrobenzoyl chloride (3.0 g, 16.2 mmol) in CH_2Cl_2 (50 mL) was added Me_2NH (2 M in THF, 16.2 mL, 32.4 mmol) at 0 °C. The reaction mixture was stirred for 2 h at ambient temperature and concentrated *in vacuo*. The residue was diluted with EtOAc (30 mL) and was washed with H_2O and brine. The combined organic layers were dried over $MgSO_4$. After filtration, the filtrate was concentrated *in vacuo* and the crude mixture (2.81 g) was used without further purification in the next step.

A round-bottom flask (100 mL) equipped with a Teflon septum and a magnetic stir bar was charged with the above crude mixture (2.81 g) and 10% Pd/C (281 mg, 10 w/w%). The flask was purged with a stream of argon and MeOH (30 mL) was added to the flask via a syringe. The reaction mixture was degassed and the flask was backfilled with hydrogen. After being stirred for 18 h, the mixture was filtered through a pad of Celite. The filtrate was concentrated *in vacuo* and the crude mixture (2.37 g) was used without further purification in the next step.

To a solution of $LiAlH_4$ (1.64 g, 43.3 mmol) in THF (60 mL) was added a solution of the crude mixture in THF (50 mL) at 0 °C. The reaction mixture was stirred for 3 h and was quenched with H_2O (1.6 mL) at 0 ˚C, followed by the addition of 1 N NaOH aq. (1.6 mL), and H_2O (4.8 mL). After filtration through a pad of Celite, the filtrate was concentrated *in vacuo* and the residue (2.00 g) was used without further purification in the next step.

To a solution of the crude product in EtOH (30 mL) was added benzaldehyde (1.69 g, 16.0 mmol) at ambient temperature. The reaction mixture was stirred for 2 h at 70 ˚C, and then the reaction mixture was cooled to 0 ˚C. Subsequently, $NaBH_4$ (754 mg, 20 mmol) was added to the reaction mixture. After stirring for 4 h at ambient temperature, H_2O (50 mL) was added to the mixture at 0 ˚C. The mixture was evaporated to remove EtOH. The organic material was extracted with Et_2O (40 mL×3) and the organic layers were dried over $MgSO_4$. After filtration, the filtrate was concentrated *in vacuo* and the residue was purified by column chromatography on silica gel (CH_2Cl_2/MeOH = 4/1) to provide **1ae** (1.68 g, 6.99 mmol, 43% in 4 steps) as a colorless oil.

1ae: ^{1}H NMR (500 MHz, $CDCl_3$): δ = 7.38-7.33 (m, 4H), 7.29-7.26 (m, 1H), 7.10 (d, J = 8.3 Hz, 2H), 6.60 (d, J = 8.3 Hz, 2H), 4.32 (d, J = 5.2 Hz, 2H), 4.00 (brs, 1H), 3.32 (s, 2H), 2.22 (s, 6H); ^{13}C NMR (125 MHz, $CDCl_3$): δ = 147.2, 139.3, 130.2, 128.5, 127.4, 127.2, 127.0, 112.5, 63.7, 48.2, 44.9; HRMS (ESI$^+$): Calcd. for $[C_{16}H_{20}N_2+H]^+$: m/z = 241.1699, Found: 241.1695.

2.3. 2-((1-Benzylpiperidin-4-yl)methyl)-5,6-dimethoxy-2,3-dihydro-1*H*-inden-1-one (1ag)

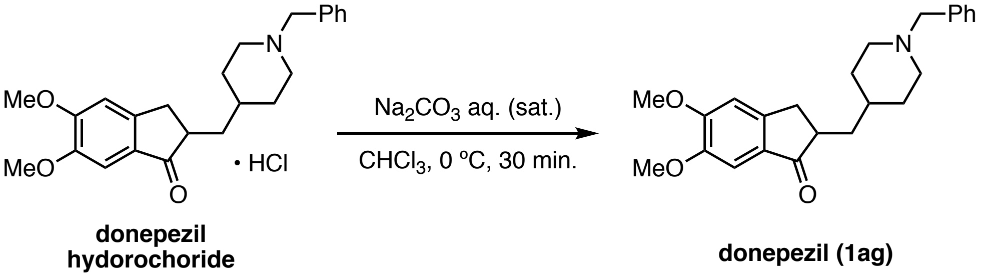

To a suspension of donepezile hydrochloride (1.0 g, 2.4 mmol) in $CHCl_3$ (200 mL) was added aqueous saturated Na_2CO_3 (60 mL) at 0 °C. After stirring for 30 min at ambient temperature, the organic material was extracted with $CHCl_3$ (60 mL×2). The combined organic layers were washed with brine and dried over $MgSO_4$. After filtration, the filtrate was concentrated *in vacuo* to provide donepezil (**1ag**) (100%, 910 mg, 2.4 mmol) as a white solid.

^{1}H NMR (500 MHz, $CDCl_3$): δ = 7.32-7.30 (m, 4H), 7.26-7.23 (m, 1H), 7.17 (s, 1H), 6.85 (s, 1H), 3.96 (s, 3H), 3.91 (s, 3H), 3.51 (s, 2H), 3.23 (dd, J = 8.0, 17.8 Hz, 1H), 2.92-2.88 (m, 2H), 2.72-2.68 (m, 2H), 2.00-1.89 (m, 3H), 1.75-1.65 (m, 2H), 1.53-1.46 (m, 1H), 1.41-1.29

(m, 3H); ^{13}C NMR (125 MHz, $CDCl_3$): δ = 207.8, 155.4, 149.4, 148.7, 138.5, 129.3, 129.2, 128.1, 126.8, 107.3, 104.3, 63.4, 56.2, 56.1, 53.8, 53.8, 45.4, 38.7, 34.5, 33.3, 33.0, 31.8; HRMS (ESI$^+$): Calcd. for $[C_{24}H_{29}NO_3+H]^+$: m/z = 380.2220, Found: 380.2221.

2.4. Methyl (*S*)-(+)-2-(2-chlorophenyl)-2-(6,7-dihydro-4*H*-thioeno[3,2-*c*]pyridin-5-yl)-acetate (1ah)

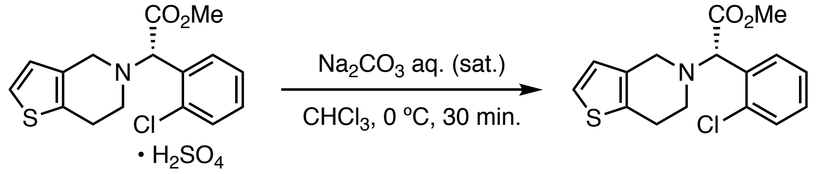

(*S*)-(+)-Clopidogrel sulfate **(*S*)-(+)-Clopidogrel (1ah)**

According to the above procedure (section 2.3.), (*S*)-(+)-clopidogrel sulfate (1.0 g, 2.4 mmol) was treated with aqueous saturated Na_2CO_3 to afford **1ah** (92%, 720 mg, 2.2 mmol) as a colorless oil.

^{1}H NMR (500 MHz, $CDCl_3$): δ = 7.70 (dd, J = 2.0, 7.2 Hz, 1H), 7.41 (dd, J = 1.7, 7.5 Hz, 1H), 7.31-7.26 (m, 2H), 7.06 (d, J = 5.2 Hz, 1H), 6.67 (d, J = 5.2 Hz, 1H), 4.92 (s, 1H), 3.76 (d, J = 14.3 Hz, 1H), 3.73 (s, 3H), 3.63 (d, J = 14.3 Hz, 1H), 2,88 (brs, 4H); ^{13}C NMR (125 MHz, $CDCl_3$): δ = 171.2, 134.6, 133.7, 133.2, 133.1, 129.9, 129.7, 129.3, 127.1, 125.1, 122.7, 67.8, 50.6, 48.2, 25.4; HRMS (ESI$^+$): Calcd. for $[C_{23}H_{19}ClN_2O_2S+H]^+$: m/z = 322.0663, Found: 322.0671.

3. C−H arylation with Ir complex under photo-irradiation

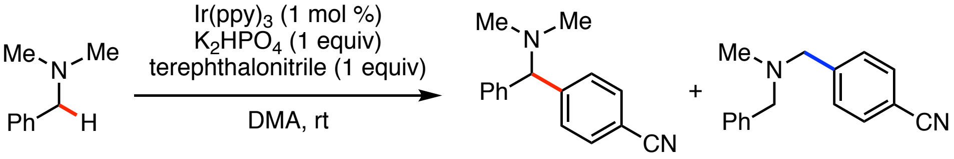

1a (3 equiv) **2a** 10% (^{1}H NMR) **3a** 85% (^{1}H NMR)

3.1. Experimental procedure

An oven-dried 5 mL Schlenk flask equipped with a Teflon septum and a magnetic stir bar was charged with Ir(ppy)$_3$ (tris[2-phenylpyridinato-C^2,*N*]iridium(III), 1.3 mg, 1.0 mol %), terephtharonitrile (25.6 mg, 0.2 mmol, 1.0 equiv) and K_2HPO_4 (34.8 mg, 1.0 mmol, 1.0 equiv). The flask was purged with a stream of argon, and DMA (1.0 mL) and *N*,*N*-dimethylbenzylamine (0.6 mmol, 3.0 equiv) were added to the flask.. The reaction mixture was then degassed with a Freeze-Pump-Thaw cycling (3 cycles) and the flask was backfilled with argon. Then, the flask was sealed with a screw cap and was placed on a stirrer (approximately 2 cm away from a 3 W blue LED (425 nm)). After being stirred for 12 h at room temperature, the reaction mixture was diluted with Et_2O (5 mL) and the organic solution was washed with H_2O and brine. The organic layer was dried over $MgSO_4$. After filtration, the filtrate was concentrated *in vacuo* and the residue was purified by column chromatography on silica gel (*n*-hexane/EtOAc = 20/1) to give **2a** (3.3 mg, 7%) as a colorless solid and **3a** (38.8 mg, 82%) as a colorless oil.

3.2.1. 1-(4-Cyanophenyl)-*N*,*N*-dimethyl-1-phenyl-methanamine (2a)

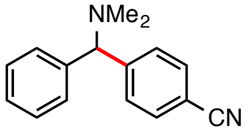

Colorless solid; 3.3 mg, 7% isolated yield; ^{1}H NMR (500 MHz, $CDCl_3$): δ = 7.57 (s, 4H), 7.36 (d, J = 7.5 Hz, 2H), 7.30-7.27 (m, 2 H), 7.23-7.19 (m, 1H), 4.12 (s, 1H), 2.18 (s, 6H); ^{13}C NMR (125 MHz, $CDCl_3$): δ = 149.1, 141.8, 132.4, 128.7, 128.3, 127.7, 127.5, 118.9, 110.6, 77.5, 44.5; IR (neat): 2820, 2774, 2226, 1607, 1491, 1452, 1016, 885, 729, 698 cm^{-1}; HRMS (ESI$^+$): Calcd. for $[C_{16}H_{16}N_2+H]^+$: *m/z* = 237.1386, Found: 237.1384.

3.2.2. 4-((Benzyl(methyl)amino)methyl)benzonitrile (3a)

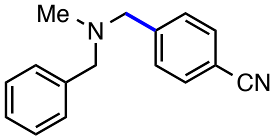

Colorless oil; 38.8 mg, 82% isolated yield; ^{1}H NMR (500 MHz, $CDCl_3$): δ = 7.61 (d, J = 8.3 Hz, 2H), 7.48 (d, J = 8.3 Hz, 2H), 7.37-7.32 (m, 4 H), 7.28-7.24 (m, 1H), 3.55 (s, 2H), 3.54 (s, 2H), 2.18 (s, 3H); ^{13}C NMR (125 MHz, $CDCl_3$): δ = 145.3, 138.8, 132.1, 129.3, 128.8, 128.3, 127.2, 119.0, 110.7, 62.0, 61.2, 42.3; IR (neat): 2789, 2226, 1607, 1495, 1452, 1366, 1018, 814, 737, 696 cm^{-1}; HRMS (ESI$^+$): Calcd. for $[C_{16}H_{16}N_2+H]^+$: *m/z* = 237.1386, Found: 237.1383.

4. Cyclic voltammetry

4.1. General experimental for cyclic voltammetry

Ferrocene was purified by recrystallisation twice from *n*-hexane prior to use. *n*-Bu_4NPF_6 was used as supplied commercially from Aldrich. Unless otherwise stated, all solutions were prepared at 10.0 mM concentration (in 0.1 M *n*-Bu_4NPF_6/MeCN or DMA as solvent) using anhydrous CH_3CN or DMA. Unless otherwise stated, a default scan rate of 100 mV s^{-1} was used and potentials are given relative to the saturated calomel electrode (*vs.* SCE). Peak heights are given in amps (A). Ferrocene was used as an external standard, measured both before and after running any series of analytes, to ensure consistency. Measurements performed vs. Ag/AgCl were converted to vs. SCE by subtracting 45 mV. The anodic-cathodic peak separation for ferrocene ΔE^p obtained was 117 mV (compared to 59 mV/n for an ideal one-electron transfer), indicating a high degree of reversibility (and so rapid kinetics) for the electron transfer process. For reductions, the sample was first degassed by Ar bubbling for 5 min. Cyclic voltammetry was conducted using a three-electrode setup consisting of a platinum wire working electrode (d = 1.60 mm) and platinum gauge counter electrode. The reference electrode was a Ag/AgCl electrode (containing 3.0 M NaCl saturated with AgCl). Electrochemical measurements were carried out in a 20 mL cell (SCV-3 Voltammetry cell) using BAS ALS660E potentiostat.

4.2. Results

The ferrocene peak height (*ca.* 7.0 x 10^{-5} A) corresponds to a one-electron oxidation. All oxidations of analytes gave a similar peak height (*ca.* 3.5 x 10^{-5} A to 7.0 x 10^{-5} A) to ferrocene, corresponding to a one-electron oxidation (except Ir(ppy)$_3$ which was analyzed at 1.0 mM concentration and was not fully soluble at that concentration). No peaks were observed for electrochemical reductions of the substrates chosen.

4.2.1. Results in CH_3CN

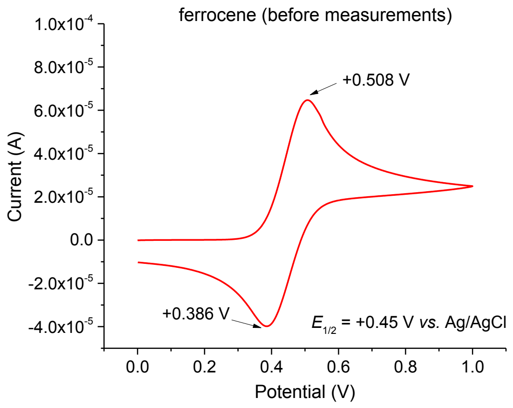

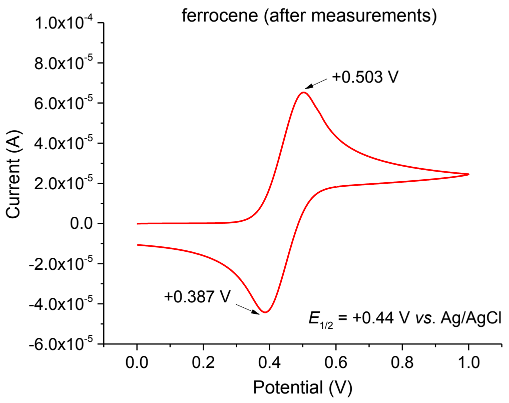

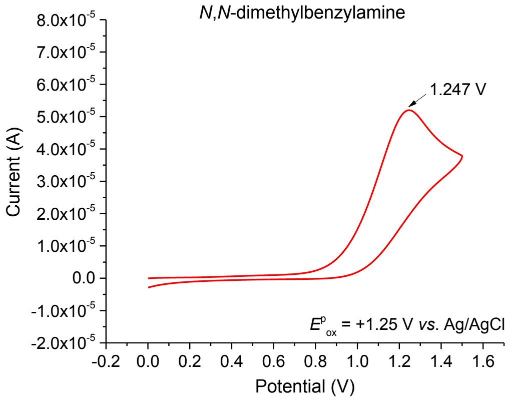
N,N-dimethylbenzylamine
1.247 V
Current (A)
Potential (V)
E^p_ox = +1.25 V vs. Ag/AgCl

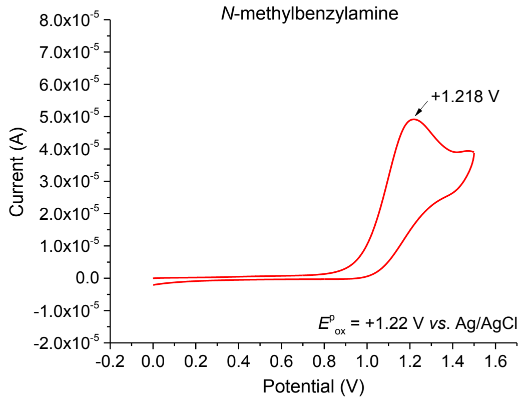
N-methylbenzylamine
+1.218 V
Current (A)
Potential (V)
E^p_ox = +1.22 V vs. Ag/AgCl

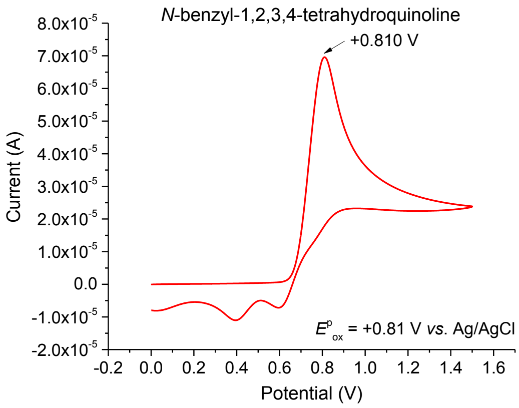
N-benzyl-1,2,3,4-tetrahydroquinoline
+0.810 V
Current (A)
Potential (V)
E^p_ox = +0.81 V vs. Ag/AgCl

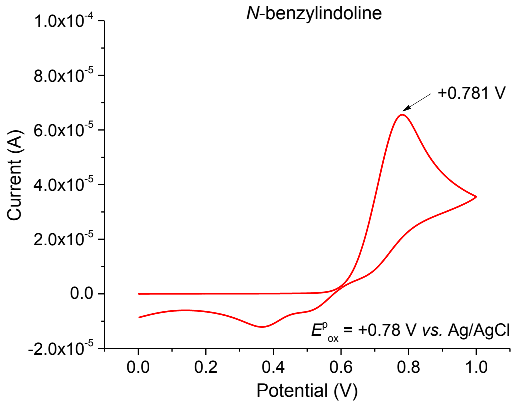
N-benzylindoline
+0.781 V
Current (A)
Potential (V)
E^p_ox = +0.78 V vs. Ag/AgCl

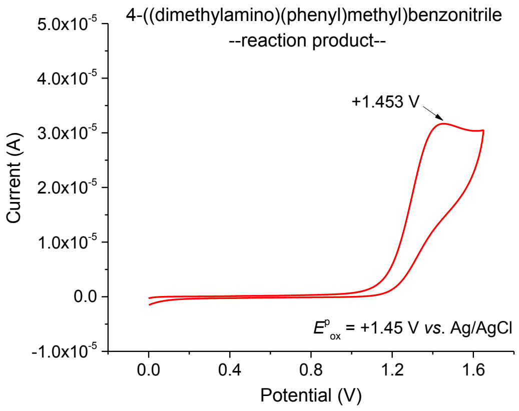
4-((dimethylamino)(phenyl)methyl)benzonitrile
--reaction product--
+1.453 V
Current (A)
Potential (V)
E^p_ox = +1.45 V vs. Ag/AgCl

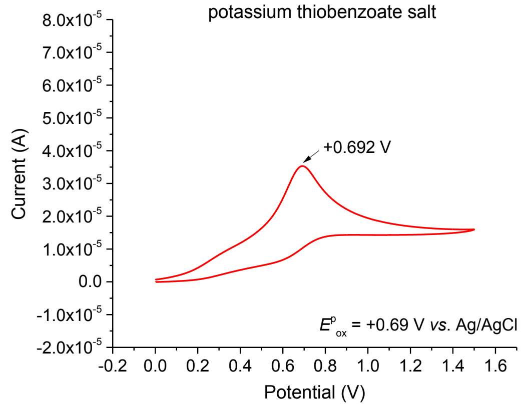
potassium thiobenzoate salt
+0.692 V
Current (A)
Potential (V)
E^p_ox = +0.69 V vs. Ag/AgCl

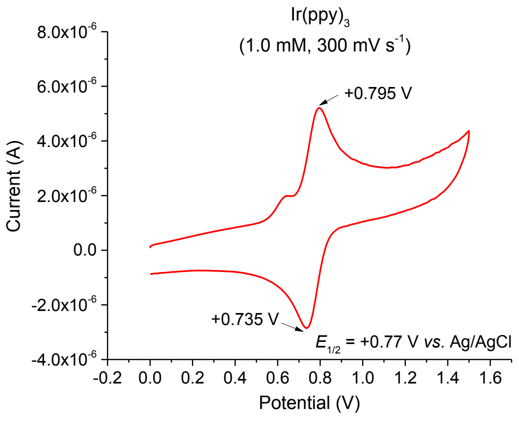
Ir(ppy)3
(1.0 mM, 300 mV s-1)
+0.795 V
+0.735 V
Current (A)
Potential (V)
E1/2 = +0.77 V vs. Ag/AgCl

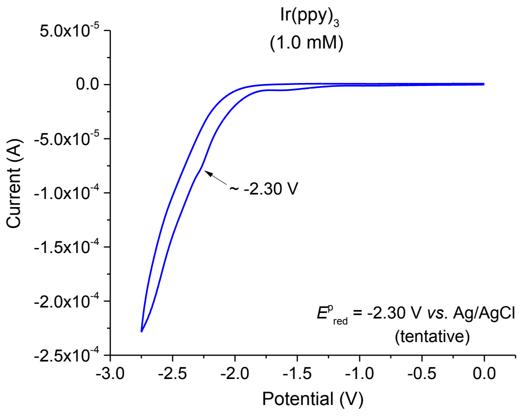
Ir(ppy)3
(1.0 mM)
~ -2.30 V
Current (A)
Potential (V)
E^p_red = -2.30 V vs. Ag/AgCl
(tentative)

All CV data in CH_3CN are summarised in Table S1 (these data have been converted from vs. Ag/AgCl as shown in Section 4.2.1. to vs. SCE in Table S1). Where possible, the data in the literature are shown for comparison (and calibrated to vs. SCE by using ferrocene, if necessary and if reported in the literature study). The reported redox potentials are quoted *vs.* SCE and are in good agreement with measured values herein. As far as possible, the redox potentials quoted have been obtained using the identical CV conditions (298 K, 0.1 M *n*-Bu_4NPF_6 in CH_3CN), but variations occur in some cases. Readers are referred to the literature referenced in Table S1 for details.

Table S1: Comparison of measured potentials in CH_3CN with those in the literature .

Entry	Redox active species	E (V vs. SCE)[a]	Literature $E_{1/2}$ (V vs. SCE)
1	Ferrocene	$E_{1/2}$ = +0.40 (E^{p}_{ox} = +0.46)[b]	+0.40[4]
2	Ir(ppy)$_3$ (reduction to Ir^{II})	$E_{1/2}$ = -2.29 (E^{p1}_{red} = *ca.* -2.35)[c]	-2.19[5]
3	Ir(ppy)$_3$ (oxidation to Ir^{IV})	$E_{1/2}$ = +0.73 (E^{p}_{ox} = +0.79)[d]	+0.77[5]
4	*N*,*N*-dimethylbenzylamine	E^{p}_{ox} = +1.17[e]	+0.92,[6] +1.01[7]
5	*N*-methylbenzylamine	E^{p}_{ox} = +1.17	ca. +1.20[8]
6	*N*-benzylindoline	E^{p}_{ox} = +0.74	-
7	*N*-benzyl-1,2,3,4-tetrahydroquinoline	E^{p}_{ox} = +0.77	-
8	Reaction product from *N*,*N*-dimethylbenzylamine	E^{p}_{ox} = +1.41	-
9	Potassium thiobenzoate salt	E^{p}_{ox} = +0.65	-

[a]Values are calibrated to ferrocene as an external standard, run before and after a set of analytes to ensure consistency. All values are in CH_3CN vs. SCE. Where possible, both half potentials $E_{1/2}$ and peak potentials E_p are given. All samples were measured vs. Ag/AgCl and were converted to vs. SCE by subtracting 45 mV. [b]Average of 6 measurements. [c]Since only the E^{p}_{red} could be observed in the CV measurement of the ground state, the value was converted to vs. SCE and then $E_{1/2}$ was estimated by addition of 60 mV (the difference in potentials between E^{p}_{ox} and $E_{1/2}$ for ferrocene) to E^{p}_{red}. [d]Calibrated by using ferrocene analyzed at 300 mV s^{-1} and ferrocene analysed at 100 mV s^{-1} to give values comparable at 100 mV s^{-1}. [e]Average of 2 measurements.

4.2.2. Results in DMA

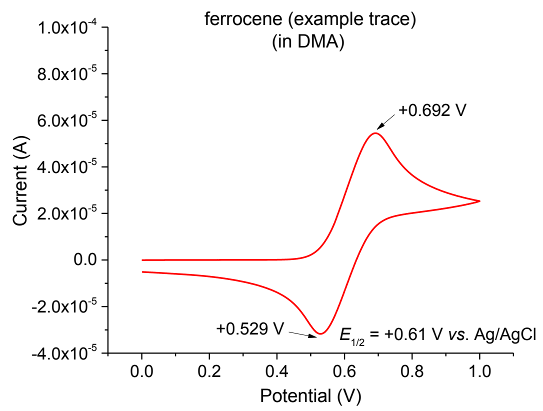

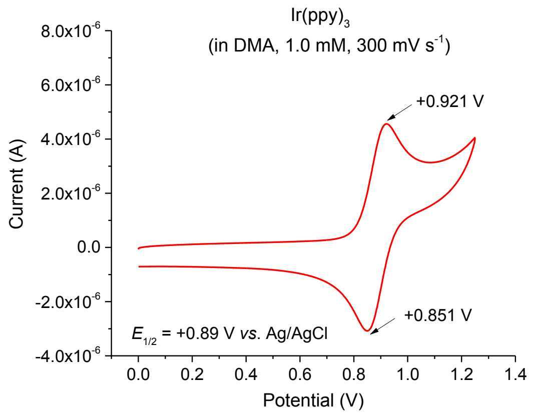

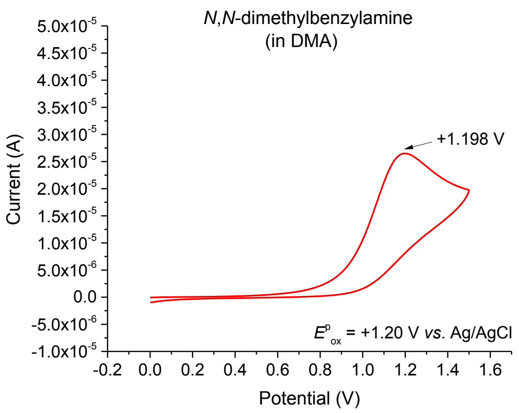

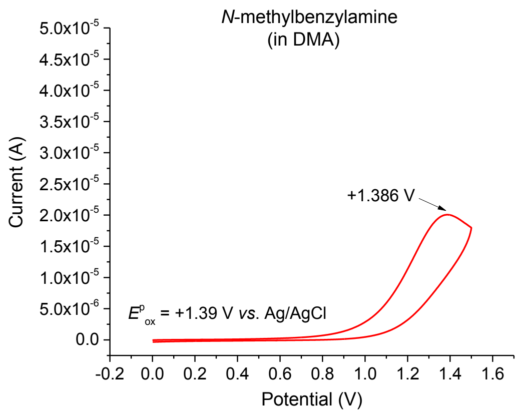

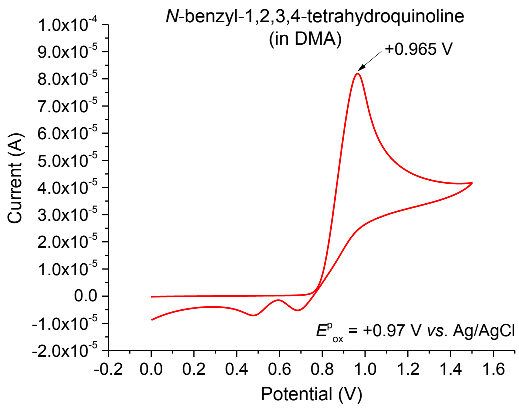

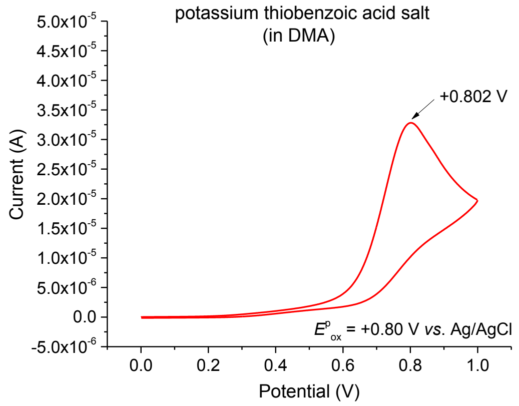

All CV data in DMA are summarised in Table S2. Where possible, the data in the literature are shown for comparison (and calibrated to vs. SCE by using ferrocene, if necessary and if reported in the literature study). The reported redox potentials are quoted *vs.* SCE. As far as possible, literature redox potentials quoted have been obtained using the identical CV conditions (298 K, 0.1 M *n*-Bu_4NPF_6 in DMA), but variations occur in some cases. Readers are referred to the literature referenced in Table S2 for details.

Table S2: Comparison of measured potentials in DMA with those in the literature.

Entry	Redox active species	$E_{1/2}$ (V vs. SCE)[a]	Literature $E_{1/2}$ (V vs. SCE)
**1**	Ferrocene	+0.53 (E^{p}_{ox} = +0.65)[b]	+0.55[9]
**2**	Ir(ppy)$_3$ (oxidation to Ir^{IV})	+0.84 (E^{p}_{ox} = +0.92)[c]	-
**3**	*N*,*N*-dimethylbenzylamine	E^{p}_{ox} = +1.15	-
**4**	*N*-methylbenzylamine	E^{p}_{ox} = +1.34	-
**5**	*N*-benzyl-1,2,3,4-tetrahydroquinoline	E^{p}_{ox} = +0.92	-
**6**	Potassium thiobenzoate salt	E^{p}_{ox} = +0.76	+0.72[10]

[a]Values are calibrated to ferrocene as an external standard, run before and after a set of analytes to ensure consistency. Unless otherwise stated, all values are half-wave potentials ($E_{1/2}$) in DMA vs. SCE. All samples were measured vs. Ag/AgCl and were converted to vs. SCE by subtracting 45 mV. [b]Average of 2 measurements. [c]Calibrated by using ferrocene analyzed at 300 mV s^{-1} and ferrocene analysed at 100 mV s^{-1} to give values comparable at 100 mV s^{-1}.

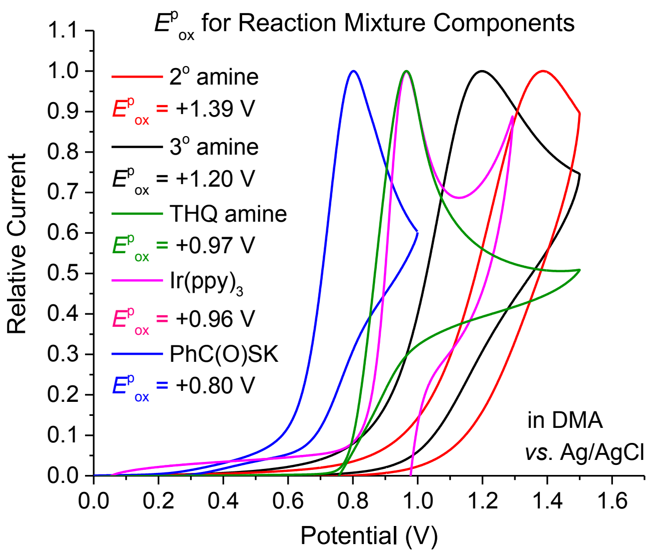

4.3. Comparison of the measured potentials of the above compounds

The above results clearly show that potassium thiobenzoate salt undergoes most facile oxidation under the reaction conditions. The *N*-benzyl-1,2,3,4-tetrahydroquinoline has an E^{p}_{ox} value almost identical to that of Ir(ppy)$_3$, however the potassium thiobenzoate salt is present under the reaction conditions. The difference in the observed reaction selectivity between *N*-benzyl-1,2,3,4-tetrahydroquinoline and *N*-benzylindoline cannot be explained by the change

in reaction mechanism to SET only catalysis, since these compounds have almost identical E^{p}_{ox} values in MeCN. The SET/HAT synergistic catalytic mechanism is occurring for these substrates (not oxidation by $[Ir(ppy)_3]^{4+}$) and the change in the reaction selectivity must be attributed to either sterics, BDEs or conformation of the 5-/6-/7-membered rings to allow the formation of the 2° α-amino radical (See, Scheme 3B).

5. Screening of the reaction conditions for benzylic C–H arylation

Table S3: Optimization of the reaction conditions.[a]

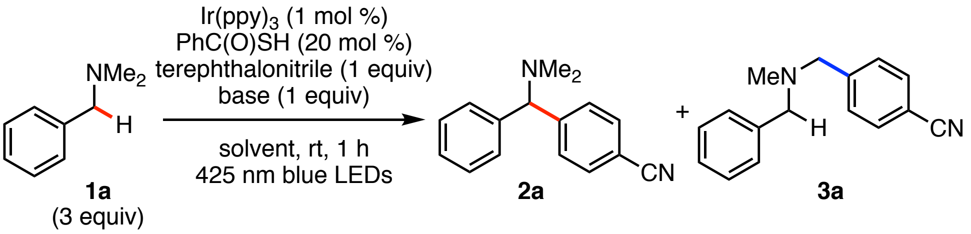

entry	base	solvent	yield (%)[b]	**2a : 3a**[b]
1	K_2HPO_4	DMA	93	> 20 : 1
2	K_3PO_4	DMA	85	> 20 : 1
3	KOAc	DMA	89	> 20 : 1
4	K_2CO_3	DMA	91	> 20 : 1
5	Na_2CO_3	DMA	90	> 20 : 1
6	K_2HPO_4	DMF	89	> 20 : 1
7	K_2HPO_4	CH_3CN	31	> 20 : 1
8	K_2HPO_4	acetone	23	> 20 : 1
9	K_2HPO_4	THF	trace	–
10	K_2HPO_4	CH_2Cl_2	23	> 20 : 1
11[c]	K_2HPO_4	DMA	90 (87)[d]	> 20 : 1
12[c,e]	K_2HPO_4	DMA	no reaction	

[a]The reactions were carried out on a 0.2 mmol scale. [b]Yield and regioisomeric ratio were determined by ^{1}H NMR analysis using 1,1,2,2-tetrachloroethane as an internal standard. [c]Run with $Ir(ppy)_3$ (0.5 mol %), thiobenzoic acid (1 mol %), and **1a** (2 equiv) for 2 h. [d]Isolated yield. [e]Run under the dark conditions.

6. Benzylic C−H arylation under SET-HAT synergistic catalysis

6.1. Typical experimental procedure for the benzylic arylation of benzylamines

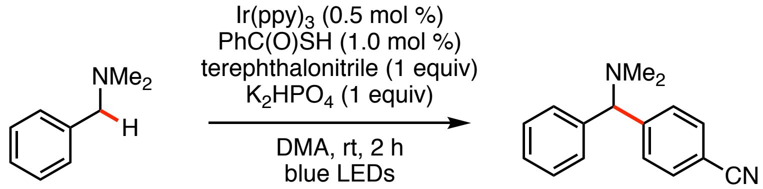

1a (2 equiv) → **2a** 87% (r.r. = >20 : 1)

An oven-dried 25 mL Schlenk flask equipped with a Teflon septum and a magnetic stir bar was charged with $Ir(ppy)_3$ (tris[2-phenylpyridinato-C^2,*N*] iridium(III), 3.3 mg, 0.5 mol %), terephthalonitrile (128.1 mg, 1.0 mmol, 1.0 equiv) and K_2HPO_4 (174.2 mg, 1.0 mmol, 1.0 equiv). The flask was purged with a stream of argon, and a DMA solution (5 mL) of ,*N*,*N*-dimethylbenzylamine (300 μL, 2 mmol, 2.0 equiv) and thiobenzoic acid (1.2 μL, 1 mol %) was added to the flask. The reaction mixture was then degassed with a Freeze-Pump-Thaw cycling (3 cycles) and the flask was backfilled with argon. Then, the flask was sealed with a screw cap and was placed on a stirrer (approximately 2 cm away from a 3 W blue LED (425 nm)). After stirring for 2 h at room temperature, the reaction mixture was diluted with Et_2O (5 mL) and the organic solution was washed with aqueous saturated $NaHCO_3$ and brine. The organic layer was dried over $MgSO_4$. After filtration, the filtrate was concentrated *in vacuo* and the residue was purified by column chromatography on silica gel (*n*-hexane/EtOAc = 20/1) to give **2a** (205.6 mg, 87% yield) as a colorless solid.

6.2.1. 1-(4-Cyanophenyl)-*N*,*N*-dimethyl-1-(4-methoxyphenyl)methanamine (2b)

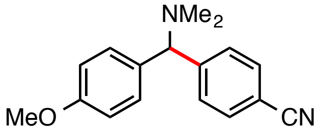

Colorless solid; 234.4 mg, 88% isolated yield; ^{1}H NMR (500 MHz, $CDCl_3$): δ = 7.57 (d, J = 8.6 Hz, 2H), 7.54 (d, J = 8.6 Hz, 2H), 7.26 (d, J = 9.2 Hz, 2H), 6.82 (d, J = 9.2 Hz, 2H), 4.08 (s, 1H), 3.76 (s, 3H), 2.17 (s, 6H); ^{13}C NMR (125 MHz, $CDCl_3$): δ = 158.8, 149.5, 133.9, 132.3, 128.8, 128.1, 118.9, 114.0, 110.4, 76.7, 55.2, 44.5; IR (neat): 2957, 2810, 2769, 2227, 2158, 2031, 1977, 1607, 1512, 1240, 1029, 887, 812 cm^{-1}; HRMS (ESI$^+$): Calcd. for $[C_{17}H_{18}N_2O+H]^+$: m/z = 267.1492, Found: 267.1492.

6.2.2. 1-(4-Chlorophenyl)-1-(4-cyanophenyl)-*N*,*N*-dimethyl-methanamine (2c)

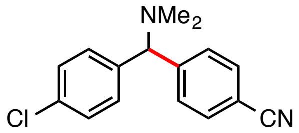

Colorless solid; 181.4 mg, 67% isolated yield; ^{1}H NMR (500 MHz, $CDCl_3$): δ = 7.58 (d, J = 8.0 Hz, 2H), 7.52 (d, J = 8.0 Hz, 2H), 7.30 (d, J = 8.6 Hz, 2H), 7.26 (d, J = 8.6 Hz, 2H), 4.11

(s, 1H), 2.17 (s, 6H); ^{13}C NMR (125 MHz, $CDCl_3$): δ = 148.5, 140.4, 133.1, 132.4, 129.0, 128.9, 128.2, 118.7, 110.8, 76.6, 44.4; IR (neat): 2776, 2228, 2100, 1607, 1487, 1406, 1089, 1012, 889, 812 cm^{-1}; HRMS (ESI$^+$): Calcd. for $[C_{16}H_{15}ClN_2+H]^+$: *m/z* = 271.0997, Found: 271.0994.

6.2.3. 1-(4-Bromophenyl)-1-(4-cyanophenyl)-*N*,*N*-dimethyl-methanamine (2d)

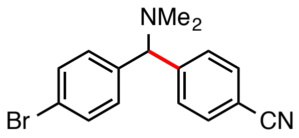

Colorless solid; 192.3 mg, 61% isolated yield; ^{1}H NMR (500 MHz, $CDCl_3$): δ = 7.58 (d, *J* = 8.3 Hz, 2H), 7.52 (d, *J* = 8.3 Hz, 2H), 7.42 (d, *J* = 8.6 Hz, 2H), 7.25 (d, *J* = 8.6 Hz, 2H), 4.10 (s, 1H), 2.17 (s, 6H); ^{13}C NMR (125 MHz, $CDCl_3$): δ = 148.4, 140.9, 132.4, 131.8, 129.3, 128.2, 121.2, 118.7, 110.9, 76.6, 44.4; IR (neat): 2949, 2874, 2783, 2228, 1607, 1487, 1474, 1460, 1402, 1169, 1009, 891, 808 cm^{-1}; HRMS (ESI$^+$): Calcd. for $[C_{16}H_{15}BrN_2+H]^+$: *m/z* = 315.0491, Found: 315.0490.

6.2.4. 1-(4-Cyanophenyl)-*N*,*N*-dimethyl-1-(naphthalen-2-yl)methanamine (2e)

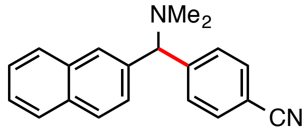

Colorless solid; 160.4mg, 56% isolated yield; ^{1}H NMR (500 MHz, $CDCl_3$): δ = 7.81-7.76 (m, 4H), 7.63 (d, *J* = 8.3 Hz, 2H), 7.57 (d, *J* = 8.3 Hz, 2H), 7.51 (dd, *J* = 1.7, 8.6 Hz, 1H), 7.48-7.42 (m, 2H), 4.30 (s, 1H), 2.24 (s, 6H); ^{13}C NMR (125 MHz, $CDCl_3$): δ = 148.9, 139.4, 133.3, 132.8, 132.4, 128.6, 128.4, 127.8, 127.6, 126.6, 126.2, 126.0, 125.3, 118.8, 110.7, 77.6, 44.6; IR (neat): 3075, 2820, 2770, 2224, 1607, 1503, 1456, 1269, 1028, 899, 818 cm^{-1}; HRMS (ESI$^+$): Calcd. for $[C_{20}H_{18}N_2+H]^+$: *m/z* = 287.1543, Found: 287.1538.

6.2.5. 1-(4-Cyanophenyl)-*N*,*N*-dimethyl-1-(*p*-tolyl)methanamine (2f)

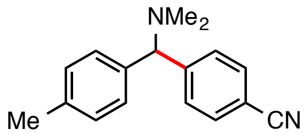

Colorless oil; 240.3 mg, 96% isolated yield; ^{1}H NMR (500 MHz, $CDCl_3$): δ = 7.56 (s, 4H), 7.24 (d, *J* = 7.7 Hz, 2H), 7.10 (d, *J* = 7.7 Hz, 2H), 4.08 (s, 1H), 2.29 (s, 3H), 2.18 (s, 6H); ^{13}C NMR (125 MHz, $CDCl_3$): δ = 149.4, 138.8, 137.1, 132.3, 129.4, 128.2, 127.6, 118.9, 110.5, 77.1, 44.5, 21.0; IR (neat): 2987, 2951, 2818, 2774, 2226, 1605, 1501, 1456, 1410, 1252, 1182, 1016, 889, 810 cm^{-1}; HRMS (ESI$^+$): Calcd. for $[C_{17}H_{18}N_2+H]^+$: *m/z* = 251.1543, Found: 251.1540.

6.2.6. 1-(4-Cyanophenyl)-*N*,*N*-dimethyl-1-(*o*-tolyl)methanamine (2g)

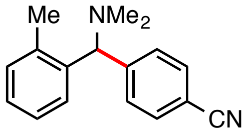

Colorless oil; 187.8 mg, 75% isolated yield; ^{1}H NMR (500 MHz, $CDCl_3$): δ = 7.66 (d, J = 8.0 Hz, 1H), 7.56 (d, J = 8.6 Hz, 2H), 7.53 (d, J = 8.6 Hz, 2H), 7.23-7.20 (m, 1H), 7.13-7.07 (m, 2H), 4.35 (s, 1H), 2.36 (s, 3H), 2.18 (s, 6H); ^{13}C NMR (125 MHz, $CDCl_3$): δ = 148.4, 140.1, 135.4, 132.2, 130.7, 128.9, 127.2, 127.0, 126.5, 118.8, 110.6, 72.3, 44.7, 20.0; IR (neat): 2988, 2951, 2818, 2771, 2226, 1605, 1458, 1016, 891, 745 cm^{-1}; HRMS (ESI^{+}): Calcd. for $[C_{17}H_{18}N_2+H]^+$: m/z = 251.1543, Found: 251.1545.

6.2.7. 1-(4-Cyanophenyl)-*N*,*N*-dimethyl-1-phenyl-ethanamine (2h)

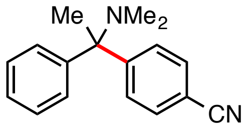

Colorless solid; 185.3 mg, 74% isolated yield; ^{1}H NMR (500 MHz, $CDCl_3$): δ = 7.62 (d, J = 8.6 Hz, 2H), 7.57 (d, J = 8.6 Hz, 2H), 7.39 (d, J = 7.5 Hz, 2H), 7,31-7.27 (m, 2H), 7.21-7.19 (m, 1H), 2.13 (s, 6H), 1.75 (s, 3H); ^{13}C NMR (125 MHz, $CDCl_3$): δ = 152.2, 143.9, 131.7, 128.2, 128.0, 127.5, 126.6, 119.0, 109.8, 67.0, 39.7, 18.8; IR (neat): 2982, 2945, 2822, 2778, 2224, 1605, 1443, 1215, 1045, 959, 858, 694 cm^{-1}; HRMS (ESI^{+}): Calcd. for $[C_{17}H_{18}N_2+H]^+$: m/z = 251.1543, Found: 251.1539.

6.2.8. 1-(4-Cyanophenyl)-*N*,*N*-diethyl-1-phenyl-methanamine (2i)

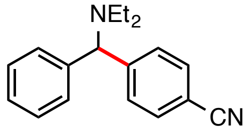

Colorless oil; 232.6 mg, 88% isolated yield; ^{1}H NMR (500 MHz, $CDCl_3$): δ = 7.56 (s, 4H), 7.35 (d, J = 7.5 Hz, 2H), 7.30-7.27 (m, 2H), 7.22-7.20 (m, 1H), 4.75 (s, 1H), 2.60-2.48 (m, 4H), 0.97 (t, J = 6.9 Hz, 6H); ^{13}C NMR (125 MHz, $CDCl_3$): δ = 150.7, 140.3, 131.9, 128.3, 128.3, 128.2, 126.8, 119.1, 110.3, 57.6, 53.8, 42.9, 15.0, 12.1; IR (neat): 2968, 2226, 1607, 1491, 1452, 1373, 1165, 1053, 912, 812, 729, 698 cm^{-1}; HRMS (ESI^{+}): Calcd. for $[C_{18}H_{20}N_2+H]^+$: m/z = 265.1699, Found: 265.1695.

6.2.9. *N*-((4-Cyanophenyl)(phenyl)methyl)-pyrrolidine (2j)

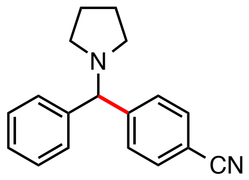

Colorless oil; 251.9 mg, 96% isolated yield; ^{1}H NMR (500 MHz, $CDCl_3$): δ = 7.59 (d, *J* = 8.6 Hz, 2H), 7.56 (d, *J* = 8.6 Hz, 2H), 7.40 (d, *J* = 7.5 Hz, 2H), 7.29-7.26 (m, 2H), 7.21-7.18 (m, 1H), 4.21 (s, 1H), 2.40 (brs, 4H), 1.78-1.77 (m, 4H); ^{13}C NMR (125 MHz, $CDCl_3$): δ = 149.7, 142.8, 132.3, 128.6, 128.1, 127.4, 127.3, 118.9, 110.5, 75.9, 53.4, 23.5; IR (neat): 2967, 2787, 2226, 1607, 1491, 1452, 1128, 893, 812, 729 cm^{-1}; HRMS (ESI^{+}): Calcd. for $[C_{18}H_{18}N_2+H]^+$: *m*/*z* = 263.1543, Found: 263.1538.

6.2.10. *N*-((4-Cyanophenyl)(phenyl)methyl)-piperidine (2k)

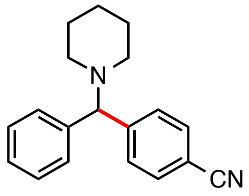

Colorless solid; 246.0 mg, 89% isolated yield; ^{1}H NMR (500 MHz, $CDCl_3$): δ = 7.56 (d, *J* = 8.6 Hz, 2H), 7.54 (d, *J* = 8.6 Hz, 2H), 7.34-7.32 (m, 2H), 7.29-7.26 (m, 2H), 7.22-7.19 (m, 1H), 4.28 (s, 1H), 2.29 (brs, 4H), 1.59-1.55 (m, 4H), 1.46-1.42 (m, 2H); ^{13}C NMR (125 MHz, $CDCl_3$): δ = 149.1, 141.5, 132.2, 128.6, 128.5, 128.0, 127.3, 119.0, 110.4, 76.3, 53.0, 26.1, 24.5; IR (neat): 2945, 2916, 2799, 2231, 1607, 1489, 1450, 1256, 1095, 991, 877, 820, 727 cm^{-1}; HRMS (ESI^{+}): Calcd. for $[C_{19}H_{20}N_2+H]^+$: *m*/*z* = 277.1699, Found: 277.1696.

6.2.11. *N*-((4-Cyanophenyl)(phenyl)methyl)-morpholine (2l)

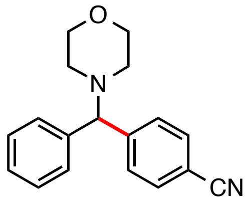

Colorless oil; 200.4 mg, 72% isolated yield; ^{1}H NMR (500 MHz, $CDCl_3$): δ = 7.58 (s, 4H), 7.37-7.35 (m, 2H), 7.31-7.28 (m, 2H), 7.24-7.20 (m, 1H), 4.26 (s, 1H), 3.72 (t, *J* = 4.9 Hz, 4H), 2.40-2.34 (m, 4H); ^{13}C NMR (125 MHz, $CDCl_3$): δ = 148.0, 140.6, 132.4, 128.8, 128.5, 127.9, 127.6, 118.7, 110.8, 76.2, 67.0, 52.4; IR (neat): 2957, 2851, 2806, 2226, 1607, 1450, 1277, 1115, 1011, 876, 729 cm^{-1}; HRMS (ESI^{+}): Calcd. for $[C_{18}H_{18}N_2O+H]^+$: *m*/*z* = 279.1492, Found: 279.1488.

6.2.12. 1-((4-Cyanophenyl)(phenyl)methyl)-4-methylpiperazine (2m)

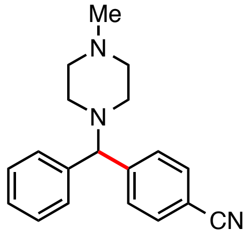

Colorless oil; 209.8 mg, 72% isolated yield; ^{1}H NMR (500 MHz, $CDCl_3$): δ = 7.56 (s, 4H), 7.36-7.34 (m, 2H), 7.30-7.26 (m, 2H), 7.22-7.19 (m, 1H), 4.27 (s, 1H), 2.42 (brs, 8H), 2.29 (s, 3H); ^{13}C NMR (125 MHz, $CDCl_3$): δ = 148.5, 141.1, 132.4, 128.7, 128.4, 127.9, 127.5, 118.8, 110.6, 75.8, 55.2, 51.8, 45.9; IR (neat): 2936, 2795, 2226, 1607, 1452, 1290, 1157, 1142, 1009, 912, 812, 729 cm^{-1}; HRMS (ESI^{+}): Calcd. for $[C_{19}H_{21}N_3+H]^+$: m/z = 292.1808, Found: 292.1802.

6.2.13. 1-(4-Cyanophenyl)-2-methy-l,2,3,4-tetrahydoroisoquinoline (2n)

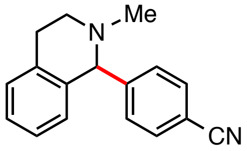

Colorless solid; 233.4 mg, 94% isolated yield; ^{1}H NMR (500 MHz, $CDCl_3$): δ = 7.61 (d, J = 8.3 Hz, 2H), 7.42 (d, J = 8.3 Hz, 2H), 7.15-7.10 (m, 2H), 7.01-6.98 (m, 1H), 6.54 (d, J = 7.5 Hz, 1H), 4.31 (s, 1H), 3.28-3.22 (m, 1H), 3.11 (ddd, J = 2.9, 5.4, 11.5, 1H), 2.85-2.80 (m, 1H), 2.65 (ddd, J = 4.0, 11.5, 11.5 Hz, 1H), 2.22 (s, 3H); ^{13}C NMR (125 MHz, $CDCl_3$): δ = 149.9, 137.1, 134.3, 132.1, 130.2, 128.6, 128.2, 126.3, 125.8, 118.9, 111.1, 71.0, 52.0, 44.3, 29.4; IR (neat): 2785, 2766, 2693, 2226, 1601, 1493, 1450, 1284, 1128, 1095, 1031. 887, 826, 739 cm^{-1}; HRMS (ESI^{+}): Calcd. for $[C_{17}H_{16}N_2+H]^+$: m/z = 249.1386, Found: 249.1385.

6.2.14. 1-(4-Cyanophenyl)-*N*-methyl-1-phenyl-methanamine (2o)

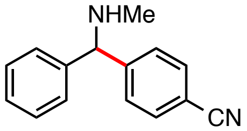

Colorless oil; 217.8 mg, 98% isolated yield; ^{1}H NMR (500 MHz, $CDCl_3$): δ = 7.59 (d, J = 8.6 Hz, 2H), 7.54 (d, J = 8.6 Hz, 2H), 7.35-7.30 (m, 4H), 7.26-7.22 (m, 1H), 4.73 (s, 1H), 2.40 (s, 3H); ^{13}C NMR (125 MHz, $CDCl_3$): δ = 149.3, 142.7, 132.3, 128.7, 127.9, 127.5, 127.1, 118.9, 110.6, 69.2, 34.9; IR (neat): 2847, 2791, 2226, 1607, 1491, 1452, 1126, 1018, 874, 816, 729, 700 cm^{-1}; HRMS (ESI^{+}): Calcd. for $[C_{15}H_{14}N_2+H]^+$: m/z = 223.1230, Found: 223.1228.

6.2.15. (4-Cyanophenyl)(phenyl)methanamine (2p)

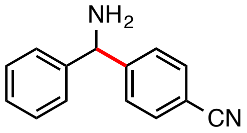

Colorless solid; 204.1 mg, 98% isolated yield; ^{1}H NMR (500 MHz, $CDCl_3$): δ = 7.61 (d, J = 8.5 Hz, 2H), 7.53 (d, J = 8.5 Hz, 2H), 7.34-7.33 (m, 4H), 7.28-7.24 (m, 1H), 5.26 (s, 1H), 1.77 (brs, 2H); ^{13}C NMR (125 MHz, $CDCl_3$): δ = 150.7, 144.3, 132.0, 128.6, 127.5, 127.4, 126.7, 118.8, 110.5, 59.4; IR (neat): 3374, 3028, 2837, 2224, 1601, 1492, 1450, 1406, 1275,

1186, 921, 893, 853, 752 cm^{-1}; HRMS (ESI^{+}): Calcd. for $[C_{14}H_{12}N_2+H]^+$: m/z = 209.1073, Found: 209.1067.

6.2.16. Methyl *N*-((4-cyanophenyl)(phenyl)methyl)-glycinate (2q)

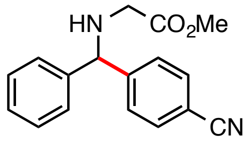

Colorless oil; 271.9 mg, 97% isolated yield; ^{1}H NMR (500 MHz, $CDCl_3$): δ = 7.60 (d, J = 8.6 Hz, 2H), 7.55 (d, J = 8.6 Hz, 2H), 7.36-7.35 (m, 2H), 7.33-7.30 (m, 2H), 7.27-7.24 (m, 1H), 4.93 (s, 1H), 3.73 (s, 3H), 3.41-3.32 (m, 2H), 2.22 (brs, 1H); ^{13}C NMR (125 MHz, $CDCl_3$): δ = 172.7, 148.6, 141.9, 132.4, 128.8, 128.0, 127.8, 127.3, 118.8, 111.0, 66.1, 51.8, 48.6; IR (neat): 2226, 1736, 1607, 1491, 1435, 1204, 1138, 986, 818, 729, 700 cm^{-1}; HRMS (ESI^{+}): Calcd. for $[C_{17}H_{16}N_2O_2+H]^+$: m/z = 281.1285, Found: 281.1278.

6.2.17. 1-(4-Cyanophenyl)-2-methyl-2,3,4,9-tetrahydro-1*H*-pyrido[3,4-*b*]indole (2r)

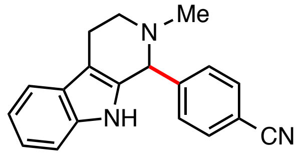

Yellow solid; 247.1 mg, 86% isolated yield; ^{1}H NMR (500 MHz, $CDCl_3$): δ = 7.65 (d, J = 8.6 Hz, 2H), 7.55-7.53 (m, 1H), 7.50 (d, J = 8.6 Hz, 2H), 7.23 (brs, 1H), 7.21-7.19 (m, 1H), 7.16-7.10 (m, 2H), 4.38 (s, 1H), 3.22 (ddd, J = 2.6, 5.4, 11.5 Hz, 1H), 3.11-3.05 (m, 1H), 2.89-2.84 (m, 1H), 2.80-2.75 (m, 1H), 2.33 (s, 3H); ^{13}C NMR (125 MHz, $CDCl_3$): δ = 146.8, 136.4, 133.2, 132.5, 129.8, 126.8, 122.0, 119.6, 118.6, 118.4, 112.0, 110.9, 109.4, 66.7, 52.7, 43.5, 21.4; IR (neat): 3375, 3032, 2941, 2845, 2806, 2230, 1607, 1454, 1306, 1265, 1061, 920, 833, 737 cm^{-1}; HRMS (ESI^{+}): Calcd. for $[C_{19}H_{17}N_3+H]^+$: m/z = 288.1495, Found: 288.1487.

6.2.18. 1-(1*H*-Indol-3-yl)-*N*,*N*-dimethyl-1-(4-cyanophenyl)methanamine (2s)

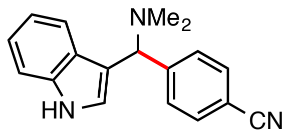

Colorless oil; 212.0 mg, 77% isolated yield; ^{1}H NMR (500 MHz, $CDCl_3$): δ = 8.10 (brs, 1H), 7.70 (d, J = 8.0 Hz, 1H), 7.65 (d, J = 8.3 Hz, 2H), 7.56 (d, J = 8.3 Hz, 2H), 7.35 (d, J = 8.0 Hz, 1H), 7.20-7.17 (m, 1H), 7.13-7.09 (m, 2H), 4.59 (s, 1H), 2.26 (s, 6H); ^{13}C NMR (125 MHz, $CDCl_3$): δ = 149.1, 136.3, 132.2, 128.5, 126.3, 122.7, 122.3, 119.8, 119.8, 119.1, 116.7, 111.2, 110.3, 69.0, 44.3 ppm; IR (neat): 2860, 2818, 2226, 1605, 1454, 1338, 1011, 736 cm^{-1}; HRMS (ESI^{+}): Calcd. for $[C_{18}H_{17}N_2+H]^+$: m/z = 276.1495, Found: 276.1491.

6.2.19. 1-(4-Cyanophenyl)-*N*-methyl-1-(pyridin-3-yl)methanamine (2t)

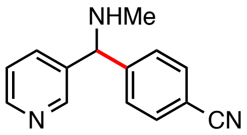

Yellow solid; 207.7 mg, 93% isolated yield; ^{1}H NMR (500 MHz, $CDCl_3$): δ = 8.62 (d, J = 1.7 Hz, 1H), 8.50 (dd, J = 1.7, 5.2 Hz, 1H), 7.65-7.64 (m, 1H), 7.62 (d, J = 8.3, 2H), 7.54 (d, J = 8.3 Hz, 2H), 7.24 (dd, J = 5.2, 7.7 Hz, 1H), 4.78 (s, 1H), 2.41 (s, 3H); ^{13}C NMR (125 MHz, $CDCl_3$): δ = 149.1, 148.9, 148.2, 138.1, 134.6, 132.5, 127.8, 123.7, 118.6, 111.2, 66.9, 34.9; IR (neat): 3291, 2945, 2793, 2228, 1605, 1501, 1425, 1304, 1132, 881, 813, 714 cm^{-1}; HRMS (ESI^{+}): Calcd. for $[C_{14}H_{13}N_3+H]^{+}$: m/z = 244.1182, Found: 244.1179.

6.2.20. *N,N*-Dimethyl-1-phenyl-1-(pyridin-4-yl)methanamine (2u)

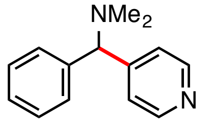

Colorless solid; 197.4 mg, 93% isolated yield; ^{1}H NMR (500 MHz, $CDCl_3$): δ = 8.50-8.49 (m, 2H), 7.38-7.36 (m, 4H), 7.29 (dd, J = 7.5, 7.5 Hz, 2H), 7.24-7.20 (m, 1H), 4.07 (s, 1H), 2.19 (s, 6H); ^{13}C NMR (125 MHz, $CDCl_3$): δ = 152.3, 150.0, 141.5, 128.7, 127.8, 127.5, 122.8, 76.8, 44.4; IR (neat): 2943, 2866, 2822, 2778, 1591, 1452, 1412, 1308, 1151, 1022, 887, 802, 741, 700 cm^{-1}; HRMS (ESI^{+}): Calcd. for $[C_{14}H_{16}N_2+H]^{+}$: m/z = 213.1386, Found: 213.1379.

6.2.21. 1-(2-Fluoropyridin-4-yl)-*N,N*-dimethyl-1-phenyl-methanamine (2v)

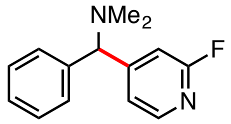

Colorless solid; 142.8 mg, 62% isolated yield; ^{1}H NMR (500 MHz, $CDCl_3$): δ = 8.10 (d, J = 5.2 Hz, 1H), 7.36-7.29 (m, 4H), 7.26-7.23 (m, 2H), 7.04 (brs, 1H), 4.12 (s, 1H), 2.19 (s, 6H); ^{13}C NMR (125 MHz, $CDCl_3$): δ = 164.1 (d, J = 238.7 Hz), 158.6 (d, J = 8.4 Hz), 147.6 (d, J = 15.6 Hz), 140.7, 128.8, 127.9, 127.8, 120.5 (d, J = 3.6 Hz), 108.2 (d, J = 37.2 Hz), 76.4 (d, J = 2.4 Hz), 44.3; IR (neat): 2992, 2963, 2818, 2770, 1611, 1566, 1480, 1285, 1267, 1145, 1022, 949, 758, 706 cm^{-1}; HRMS (ESI^{+}): Calcd. for $[C_{14}H_{15}FN_2+H]^{+}$: m/z = 231.1292, Found: 231.1290.

6.2.22. 1-(2-Chloropyridin-4-yl)-*N,N*-dimethyl-1-phenyl-methanamine (2w)

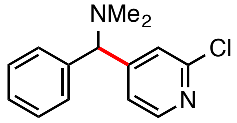

Colorless oil; 207.3 mg, 84% isolated yield; ^{1}H NMR (500 MHz, $CDCl_3$): δ = 8.27 (d, J = 5.2 Hz, 1H), 7.43 (brs, 1H), 7.35-7.29 (m, 5H), 7.26-7.23 (m, 1H), 4.07 (s, 1H), 2.19 (s, 6H); ^{13}C NMR (125 MHz, $CDCl_3$): δ = 156.0, 151.7, 149.7, 140.6, 128.8, 127.9, 127.8, 123.1, 121.5, 76.3, 44.3; IR (neat): 2988, 2953, 2820, 2776, 1585, 1545, 1454, 1379, 1078, 1022, 926, 819, 721, 700 cm^{-1}; HRMS (ESI$^+$): Calcd. for $[C_{14}H_{15}ClN_2+H]^+$: m/z = 247.0997, Found: 247.0990.

6.2.23. 1-(3-Chloropyridin-4-yl)-*N*,*N*-dimethyl-1-phenyl-methanamine (2x)

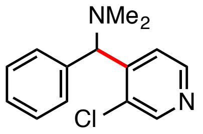

Yellow oil; 239.3 mg, 97% idolated yield; ^{1}H NMR (500 MHz, $CDCl_3$): δ = 8.47 (s, 1H), 8.46 (d, J = 5.2 Hz, 1H), 7.78 (d, J = 5.2 Hz, 1H), 7.43 (d, J = 7.5 Hz, 2H), 7.30-7.21 (m, 3H), 4.57 (s, 1H), 2.19 (s, 6H); ^{13}C NMR (125 MHz, $CDCl_3$): δ = 149.8, 149.5, 148.0, 140.1, 131.4, 128.6, 128.4, 127.7, 123.0, 71.7, 44.5; IR (neat): 2994, 2957, 2820, 2776, 2494, 2158, 2031, 1977, 1578, 1468, 1395, 1090, 1018, 885, 823, 700 cm^{-1}; HRMS (ESI$^+$): Calcd. for $[C_{14}H_{15}ClN_2+H]^+$: m/z = 247.0997, Found: 247.0993.

6.2.24. 1-(Isoquinolin-1-yl)-*N*,*N*-dimethyl-1-phenylmethanamine (2y)

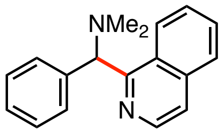

Colorless solid; 151.2 mg, 58% isolated yield; ^{1}H NMR (500 MHz, $CDCl_3$): δ = 8.65 (d, J = 8.6 Hz, 1H), 8.60 (d, J = 5.7 Hz, 1H), 7.75 (d, J = 8.0 Hz, 1H), 7.65 (d, J = 6.9 Hz, 2H), 7.61-7.34 (m, 2H), 7.49 (d, J = 5.7 Hz, 1H), 7.26-7.23 (m, 2H), 7.17-7.12 (m, 1H), 5.08 (s, 1H), 2.29 (s, 6H); ^{13}C NMR (125 MHz, $CDCl_3$): δ = 160.3, 142.2, 140.7, 136.6, 129.5, 128.8, 128.2, 127.5, 127.2, 126.9, 126.7, 124.8, 119.7, 75.2, 44.8; IR (neat): 3039, 3010, 2851, 1651, 1512, 1485, 1248, 1033, 812, 689 cm^{-1}; HRMS (ESI$^+$): Calcd. for $[C_{18}H_{18}N_2+H]^+$: m/z = 263.1543, Found: 263.1541.

6.2.25. Methyl 4-((dimethylamino)(phenyl)methyl)benzoate (2z)

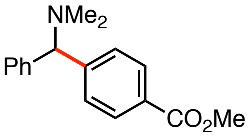

Colorless solid; 85.8 mg, 32% isolated yield; ^{1}H NMR (500 MHz, $CDCl_3$): δ =7.95 (d, J = 8.3 Hz, 2H), 7.52 (d, J = 8.3 Hz, 2H), 7.40 (d, J = 7.5 Hz, 2H), 7.29-7.26 (m, 2H), 7.19 (t, J = 7.5 Hz, 1H), 4.12 (s, 1H), 3.87 (s, 3H), 2.19 (s, 6H); ^{13}C NMR (125 MHz, $CDCl_3$): δ = 160.9 148.8, 142.5, 129.9, 128.8, 128.6, 127.8, 127.6, 127.2, 77.7, 52.0, 44.6; IR (neat): 3035, 2961,

2791, 1719, 1643, 1552, 1433, 1279, 1018, 802, 667 cm^{-1}; HRMS (ESI^{+}): Calcd. for $[C_{17}H_{19}NO_2+H]^+$: *m/z* = 270.1489, Found: 254.1489.

6.2.26. 1-((4-Cyanophenyl)(phenyl)methyl)-indoline (2aa)

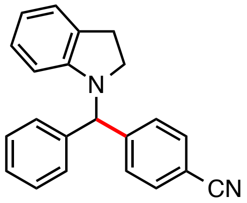

Colorless oil; 217.3 mg, 70% isolated yield; ^{1}H NMR (500 MHz, $CDCl_3$): δ = 7.62 (d, *J* = 8.3 Hz, 2H), 7.50 (d, *J* = 8.3 Hz, 2H), 7.35-7.26 (m, 5H), 7.10 (d, *J* = 7.5 Hz, 1H), 6.95-6.92 (m, 1H), 6.69-6.66 (m, 1H), 6.12 (d, *J* = 8.0 Hz, 1H), 5.53, (s, 1H), 3.23-3.18 (m, 1H), 3.13-3.08 (m, 1H), 3.01-2.90 (m, 2H); ^{13}C NMR (125 MHz, $CDCl_3$): δ = 151.4, 147.2, 139.6, 132.4, 130.4, 128.8, 128.7, 128.7, 127.8, 127.1, 124.5, 118.8, 118.2, 111.0, 108.2, 66.7, 51.6, 28.3; IR (neat): 2843, 2226, 1603, 1483, 1242, 1026, 810, 745, 700 cm^{-1}; HRMS (ESI^{+}): Calcd. for $[C_{23}H_{20}N_2+H]^+$: *m/z* = 311.1543, Found: 311.1544.

6.2.27. 1-Benzyl-2-(4-cyanophenyl)-1,2,3,4-tetrahydroquinoline (2ab)

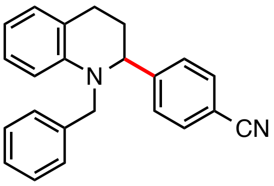

Colorless solid; 295.2 mg, 91% isolated yield; ^{1}H NMR (500 MHz, $CDCl_3$): δ = 7.60 (d, *J* = 8.0 Hz, 2H), 7.32-7.30 (m, 4H), 7.26-7.23 (m, 1H), 7.20 (d, *J* = 6.9 Hz, 2H), 7.07-7.04 (m, 1H), 7.02 (d, *J* = 6.9 Hz, 1H), 6.68-6.65 (m, 1H), 6.61 (d, *J* = 8.0 Hz, 1H), 4.77-4.72 (m, 2H), 4.15 (d, *J* = 17.2 Hz, 1H), 2.65 (ddd, *J* = 3.4, 4.0, 15.5 Hz, 1H), 2.56-2.50 (m, 1H), 2.37-2.30 (m, 1H), 2.10-2.05 (m, 1H); ^{13}C NMR (125 MHz, $CDCl_3$): δ = 149.6, 144.4, 137.8, 132.2, 128.9, 128.6, 127.5, 127.4, 126.8, 126.2, 121.6, 118.7, 116.2, 110.7, 110.5, 60.9, 52.8, 28.9, 23.2; IR (neat): 3026, 2936, 2228, 1601, 1493, 1449, 1342, 1252, 1173, 970, 844, 745 cm^{-1}; HRMS (ESI^{+}): Calcd. for $[C_{23}H_{20}N_2+H]^+$: *m/z* = 325.1699, Found: 325.1695.

6.2.28. 1-((4-Cyanophenyl)(phenyl)methyl)-2,3,4,5-tetrahydro-1*H*-benzo[*b*]azepine (2ac)

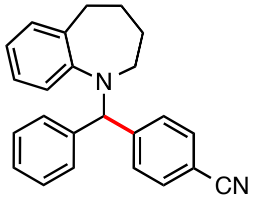

Colorless amorphous; 142.1 mg, 42% isolated yield; ^{1}H NMR (500 MHz, $CDCl_3$): δ = 7.50 (d, *J* = 8.3 Hz, 2H), 7.47 (d, *J* = 8.3 Hz, 2H), 7.39 (d, *J* = 7.5 Hz, 2H), 7.29-7.26 (m, 2H),

7.22-7.19 (m, 1H), 7.12 (d, *J* = 6.3 Hz, 1H), 6.92-6.89 (m, 1H), 6.83-6.80 (m, 1H), 6.70 (d, *J* = 8.0 Hz, 1H), 5.69 (s, 1H), 3.07-3.00 (m, 2H), 2.93-2.83 (m, 2H), 1.73-1.69 (m, 1H), 1.59-1.42 (m, 3H); ^{13}C NMR (125 MHz, $CDCl_3$): δ = 150.5, 149.3, 142.2, 136.9, 132.3, 129.8, 128.7, 128.4, 128.0, 127.4, 126.2, 121.9, 120.2, 118.8, 110.4, 71.0, 52.1, 34.7, 29.7, 25.6; IR (neat): 2228, 1595, 1491, 1451, 1236, 1132, 810, 750, 698 cm^{-1}; HRMS (ESI$^+$): Calcd. for $[C_{24}H_{22}N_2+H]^+$: *m*/*z* = 339.1856, Found: 339.1852.

6.2.29. 1-Benzyl-2-(4-cyanophenyl)-2,3,4,5-tetrahydro-1*H*-benzo[*b*]azepine (2ac')

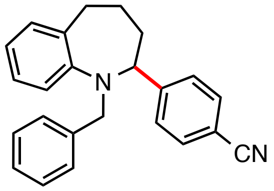

Colorless amorphous; 125.2 mg, 37% isolated yield; ^{1}H NMR (500 MHz, $CDCl_3$): δ = 7.61 (d, *J* = 8.3 Hz, 2H), 7.32 (d, *J* = 8.3 Hz, 2H), 7.28-7.25 (m, 2H), 7.22-7.12 (m, 5H), 6.98-6.93 (m, 2H), 4.28 (d, *J* = 14.9 Hz, 1H), 4.22 (dd, *J* = 2.9, 8.0 Hz, 1H), 3.99 (d, *J* = 14.9 Hz, 1H), 3.05-3.00 (m, 1H), 2.89-2.84 (m, 1H), 1.77-1.59 (m, 4H); ^{13}C NMR (125 MHz, $CDCl_3$): δ = 150.0, 147.1, 138.3, 135.2, 132.1, 128.8, 128.2, 128.0, 127.9, 127.0, 126.8, 122.4, 121.0, 118.9, 110.5, 63.4, 55.6, 32.2, 30.7, 21.1; IR (neat): 2226, 1597, 1487, 1450, 1230, 1138, 937, 827, 754, 696 cm^{-1}; HRMS (ESI$^+$): Calcd. for $[C_{24}H_{22}N_2+H]^+$: *m*/*z* = 339.1856, Found: 339.1849.

6.2.30. 1-(4-Cyanophenyl)-*N*,*N*-dimethyl-1-(4-(hydroxymethyl)-phenyl)methanamine (2ad)

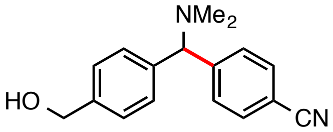

Colorless oil; 234.4 mg, 88% isolated yield; ^{1}H NMR (500 MHz, $CDCl_3$): δ = 7.57 (d, *J* = 8.6 Hz, 2H), 7.55 (d, *J* = 8.6 Hz, 2H), 7.36 (d, *J* = 8.0 Hz, 2H), 7.30 (d, *J* = 8.0 Hz, 2H), 4.64 (d, *J* = 5.4 Hz, 2H), 4.13 (s, 1H), 2.18 (s, 6H), 1.62 (t, *J* = 5.4 Hz, 1H); ^{13}C NMR (125 MHz, $CDCl_3$): δ = 149.0, 141.3, 140.2, 132.4, 128.3, 127.9, 127.4, 118.8, 110.7, 77.2, 64.9, 44.5; IR (neat): 2990, 2953, 2864, 2822, 2778, 2228, 1605, 1501, 1458, 1416, 1015, 891, 816, 787, 731 cm^{-1}; HRMS (ESI$^+$): Calcd. for $[C_{17}H_{18}N_2O+H]^+$: *m*/*z* = 267.1492, Found: 267.1485.

6.2.31. *N*-((4-Cyanophenyl)(phenyl)methyl)-4-((dimethylamino)methyl)aniline (2ae)

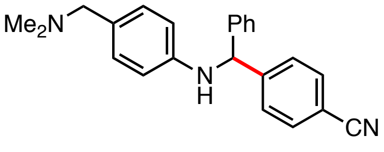

Colorless oil; 242.4 mg, 71% isolated yield; ^{1}H NMR (500 MHz, $CDCl_3$): δ = 7.62 (d, J = 8.0 Hz, 2H), 7.52 (d, J = 8.0 Hz, 2H), 7.36-7.26 (m, 5H), 7.08 (d, J = 8.3 Hz, 2H), 6.47 (d, J = 8.3 Hz, 2H), 5.51 (d, J = 3.4 Hz, 1H), 4.23 (d, J = 3.4 Hz, 1H), 3.37 (s, 2H), 2.25 (s, 6H); ^{13}C NMR (125 MHz, $CDCl_3$): δ = 148.0, 146.0, 141.6, 132.5, 130.3, 129.0, 128.0, 127.8, 127.5, 126.9, 118.7, 113.3, 111.0, 63.3, 62.8, 44.6; IR (neat): 2502, 2158, 2031, 1977, 1612, 1516, 1267, 1018, 851, 698 cm^{-1}; HRMS (ESI$^+$): Calcd. for $[C_{17}H_{18}N_2O+H]^+$: m/z = 342.1965, Found: 342.1969.

6.2.32. *N*-((4-Cyanophenyl)(phenyl)methyl)-*N*,3-dimethyl-but-2-en-1-amine(2af)

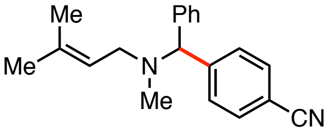

Colorless oil; 78.4 mg, 27% isolated yield; ^{1}H NMR (500 MHz, $CDCl_3$): δ = 7.56 (s, 4H), 7.35 (d, J = 7.5 Hz, 2H), 7.30-7.27 (m, 2H), 7.22-7.19 (m, 1H), 5.28 (t, J = 6.6 Hz, 1H), 4.40 (s, 1H), 2.97-2.88 (m, 2H), 2.10 (s, 3H), 1.73 (s, 3H), 1.50 (s, 3H); ^{13}C NMR (125 MHz, $CDCl_3$): δ = 149.1, 141.6, 135.2, 132.3, 128.6, 128.5, 128.0, 127.4, 121.1, 118.9, 110.5, 75.0, 52.9, 40.2, 25.9, 17.9; IR (neat): 2228, 1607, 1490, 1452, 1126, 1013, 814, 754, 729, 698 cm^{-1}; HRMS (ESI$^+$): Calcd. for $[C_{17}H_{18}N_2O+H]^+$: m/z = 291.856, Found: 291.1854.

6.2.33. 2-((1-((4-Cyanophenyl)(phenyl)methyl)piperidin-4-yl)methyl)-5,6-dimethoxy-2,3-dihydro-1*H*-inden-1-one (2ag)

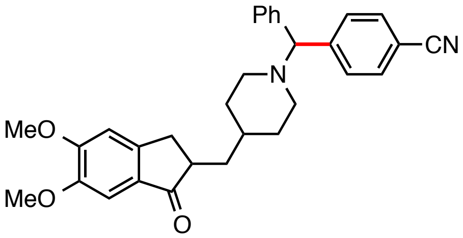

Colorless oil; 355.7mg, 74% isolated yield; ^{1}H NMR (500 MHz, $CDCl_3$): δ = 7.57 (d, J = 8.3 Hz, 2H), 7.55 (d, J = 8.3 Hz, 2H), 7.33 (d, J = 7.5 Hz, 2H), 7.30-7.27 (m, 2H), 7.23-7.20 (m, 1H), 7.16 (s, 1H), 6.84 (s, 1H), 4.31 (s, 1H), 3.96 (s, 3H), 3.90 (s, 3H), 3.22 (dd, J = 8.0, 17.2 Hz, 1H), 2.86-2.81 (m, 2H), 2.71-2.70 (m, 2H), 1.95-1.82 (m, 3H), 1.73-1.70 (m, 1H), 1.64-1.50 (m, 2H), 1.42-1.32 (m, 3H); ^{13}C NMR (125 MHz, $CDCl_3$): δ = 207.6, 155.3, 149.3, 148.9, 148.6, 141.3, 132.2, 129.1, 128.5, 128.4, 127.8, 127.2, 118.8, 110.3, 107.2, 104.2, 75.8, 56.1, 55.9, 52.6, 52.5, 51.8, 45.3, 38.5, 34.3, 33.1, 31.8; IR (neat): 2914, 2839, 2224, 1690, 1591, 1499, 1312, 1263,1121, 1034, 912, 727 cm^{-1}; HRMS (ESI$^+$): Calcd. for $[C_{17}H_{18}N_2O+H]^+$: m/z = 481.2486, Found: 481.2481.

6.2.34. Methyl (*R*)-2-(2-chlorophenyl)-2-(4-(4-cyanophenyl)-6,7-dihydrothieno[3,2-*c*] pyridin-5(4*H*)-yl)acetate (2af)

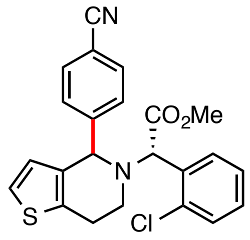

2ah: Colorless amorphous; 194.6 mg, 46% isolated yield; ^{1}H NMR (500 MHz, $CDCl_3$): δ = 7.71 (dd, *J* = 2.3, 7.5 Hz, 1H), 7.59 (d, *J* = 8.6 Hz, 2H), 7.56 (d, *J* = 8.6 Hz, 2H), 7.39 (dd, *J* = 1.7, 7.5 Hz, 1H), 7.31-7.25 (m, 2H), 7.15 (d, *J* = 5.2 Hz, 1H), 6.65 (d, *J* = 5.2 Hz, 1H), 5.11 (s, 1H), 5.07 (s, 1H), 3.64 (s, 3H), 2.92-2.82 (m, 2H), 2.66-2.58 (m, 2H); ^{13}C NMR (125 MHz, $CDCl_3$): δ = 172.4, 148.4, 135.0, 134.5, 133.5, 132.3, 131.8, 130.3, 129.9, 129.6, 129.5, 127.2, 126.8, 122.9, 119.0, 110.9, 63.4, 61.1, 52.2, 41.1, 22.2; IR (neat): 2230, 1736, 1605, 1436, 1192, 1165, 1030, 847, 756 cm^{-1}; HRMS (ESI^+): Calcd. for $[C_{17}H_{18}N_2O+H]^+$: *m*/*z* = 267.1492, Found: 267.1485.

2ah': Colorless amorphous; 211.46 mg, 50% isolated yield; ^{1}H NMR (500 MHz, $CDCl_3$): δ = 7.60 (d, *J* = 7.5 Hz, 1H), 7.57 (d, *J* = 8.0 Hz, 2H), 7.44 (d, *J* = 8.0 Hz, 2H), 7.33-7.30 (m, 2H), 7.27-7.24 (m, 1H), 7.00 (d, *J* = 5.2 Hz, 1H), 6.28 (d, *J* = 5.2 Hz, 1H), 5.10 (s, 1H), 4.66 (s, 1H), 3.76 (s, 3H), 3.26-3.23 (m, 1H), 3.08-3.00 (m, 2H), 2.91-2.86 (m, 1H); ^{13}C NMR (125 MHz, $CDCl_3$): δ = 170.6, 147.8, 135.7, 134.4, 134.2, 134.0, 132.2, 129.9, 129.7, 129.2, 129.2, 126.8, 126.1, 123.0, 118.8, 111.5, 63.8, 63.7, 51.9, 44.3, 25.0; IR (neat): 2839, 2228, 1734, 1607, 1433, 1202, 1161, 999, 827, 752 cm^{-1}; HRMS (ESI^+): Calcd. for $[C_{17}H_{18}N_2O+H]^+$: *m*/*z* = 267.1492, Found: 267.1489.

6.2.35. 4-(1-Benzylpyrrolidin-2-yl)benzonitrile (3j) (equation 1, 0.2 mmol scale)

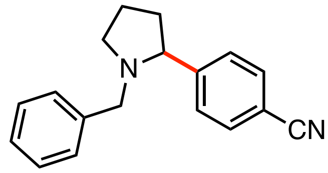

Colorless oil; 23.8 mg, 45% isolated yield; ^{1}H NMR (500 MHz, $CDCl_3$): δ = 7.63 (d, *J* = 8.6 Hz, 2H), 7.57 (d, *J* = 8.6 Hz, 2H), 7.30-7.21 (m, 5H), 3.76 (d, *J* = 13.2 Hz, 1H), 3.47 (t, *J* = 8.3 Hz, 1H), 3.14-3.10 (m, 2H), 2.29-2.19 (m, 2H), 1.94-1.76 (m, 2H), 1.70-1.62 (m, 1H); ^{13}C NMR (125 MHz, $CDCl_3$): δ = 150.2, 139.1, 132.3, 128.5, 128.2, 128.1, 126.9, 119.1, 110.6, 69.0, 58.2, 53.4, 35.3, 22.6; IR ($CHCl_3$): 3049, 2800, 2233, 1606, 1493, 835, 694 cm^{-1}; HRMS (ESI^+): Calcd. for $[C_{18}H_{18}N_2+H]^+$: *m*/*z* = 263.1543, Found: 263.1547.

7. Limitation regarding electron-deficient aromatic compounds

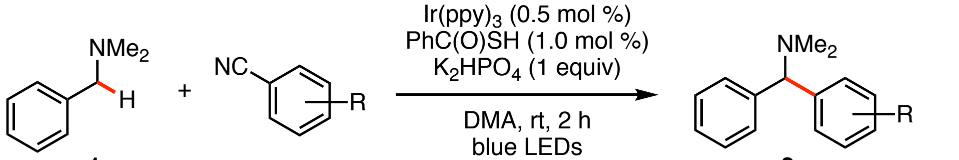

1a (2 equiv) (1 equiv) **2**

Arylating reagents:

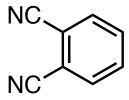

36%

Less than 20% of the products were detected when the following aryl cyanides were used.

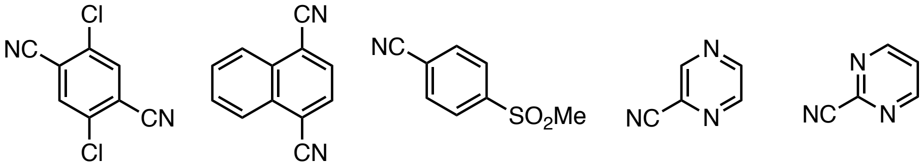

No reaction was observed when the following aryl cyanides were used.

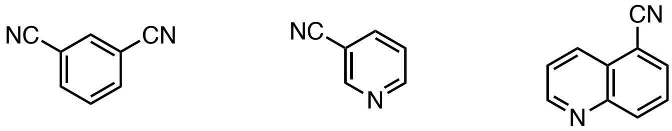

8. References

1) R. Feng, J. Yao, Z. Liang, Z. Liu and Y. Zhang, *J. Org. Chem.*, 2013, **78**, 3688-3696.
2) Z. Li, C. Gelbaum, J. S. Fisk, B. Holden, A. Jaganathan, G. T. Whiteker, P. Pollet and C. L. Liotta, *J. Org. Chem.*, 2016, **81**, 8520-8529.
3) E. Sperotto, G. P. M. Klink, J. G. Vries and G. Koten, *Tetrahedron*, 2010, **66**, 3478-3484.
4) N. G. Connelly and W. E. Geiger, *Chem. Rev.*, 1996, **96**, 877-910.
5) C. K. Prier, D. A. Rankic and D. W. C. MacMillan, *Chem. Rev.*, 2013, **113**, 5322-5363.
6) K. Hoshino, T. Ogawa, S. Yasuike, H. Seki, J. Kurita, T. Tokunaga, K. Yamaguchi, *J. Phys. Chem. B*, 2004, **108**, 18698-18704.
7) R. Ballardini, G. Varani, M. T. Indelli, F. Scandola and V. Balzani, *J. Am. Chem. Soc.*, 1978, **100***,* 7219-7223.
8) J. Athilakshmi and D. K. Chand, *Tetrahedron Lett.*, 2010, **51**, 6760-6762.
9) K. D. Welch, D. P. Harrison, M. Sabat, E. Z. Hejazi, B. T. Parr, M. G. Fanelli, N. A. Gianfrancesco, D. S. Nagra, W. H. Myers and W. D. Harman, *Organometallics*, 2009, **28**, 5960-5967.
10) J. Robert, M. Anouti and J. Paris, *J. Chem. Soc. Perkin Trans. 2*, 1997, 473-478; Also see: R. Zhao, J. Lind, G. Merényi and T. E. Eriksen, *J. Am. Chem. Soc.*, 1998, **120**, 2811-2816.

9. NMR spectra

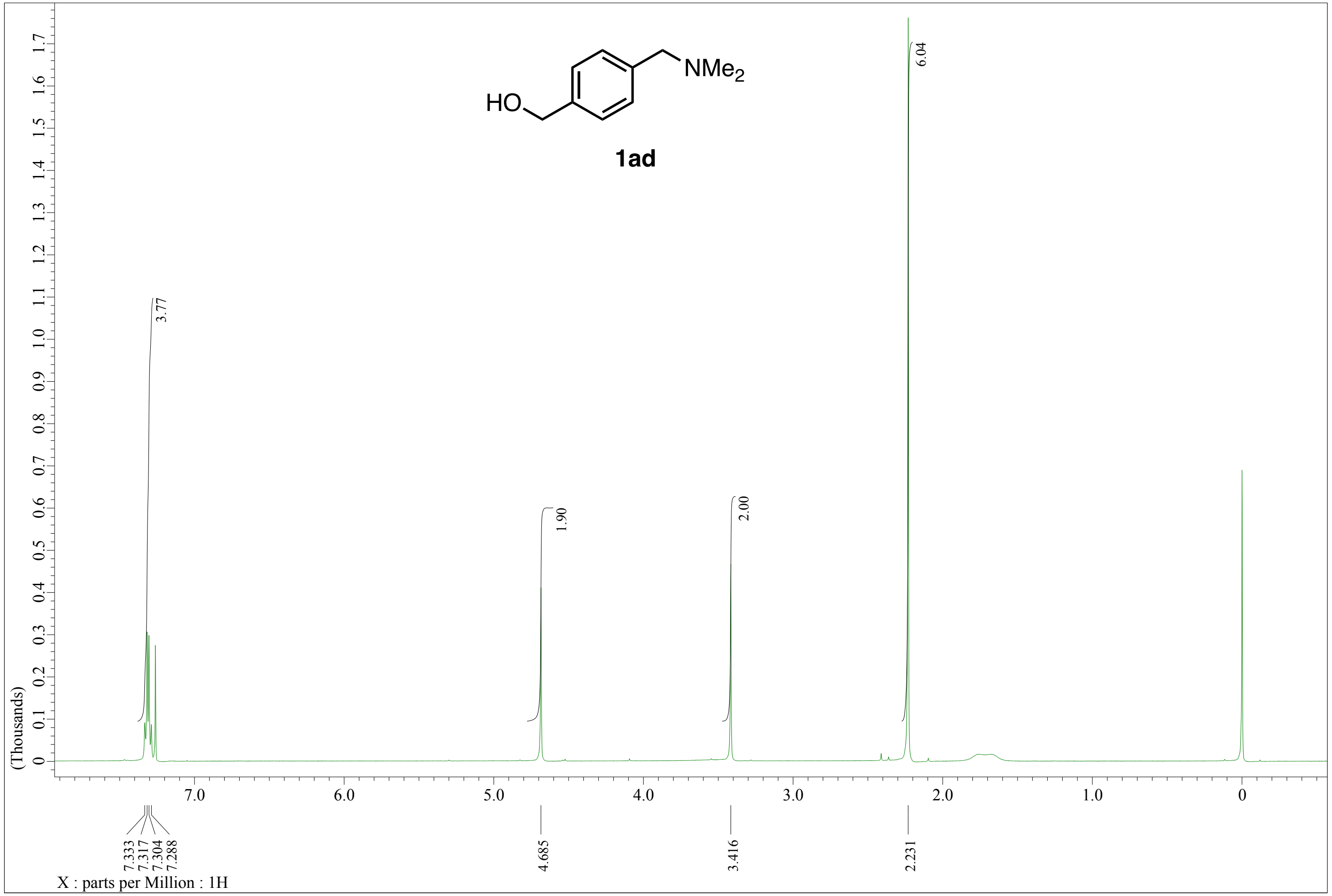
HO
NMe2
1ad
3.77
1.90
2.00
6.04
(Thousands)
7.333
7.317
7.304
7.288
4.685
3.416
2.231
X : parts per Million : 1H

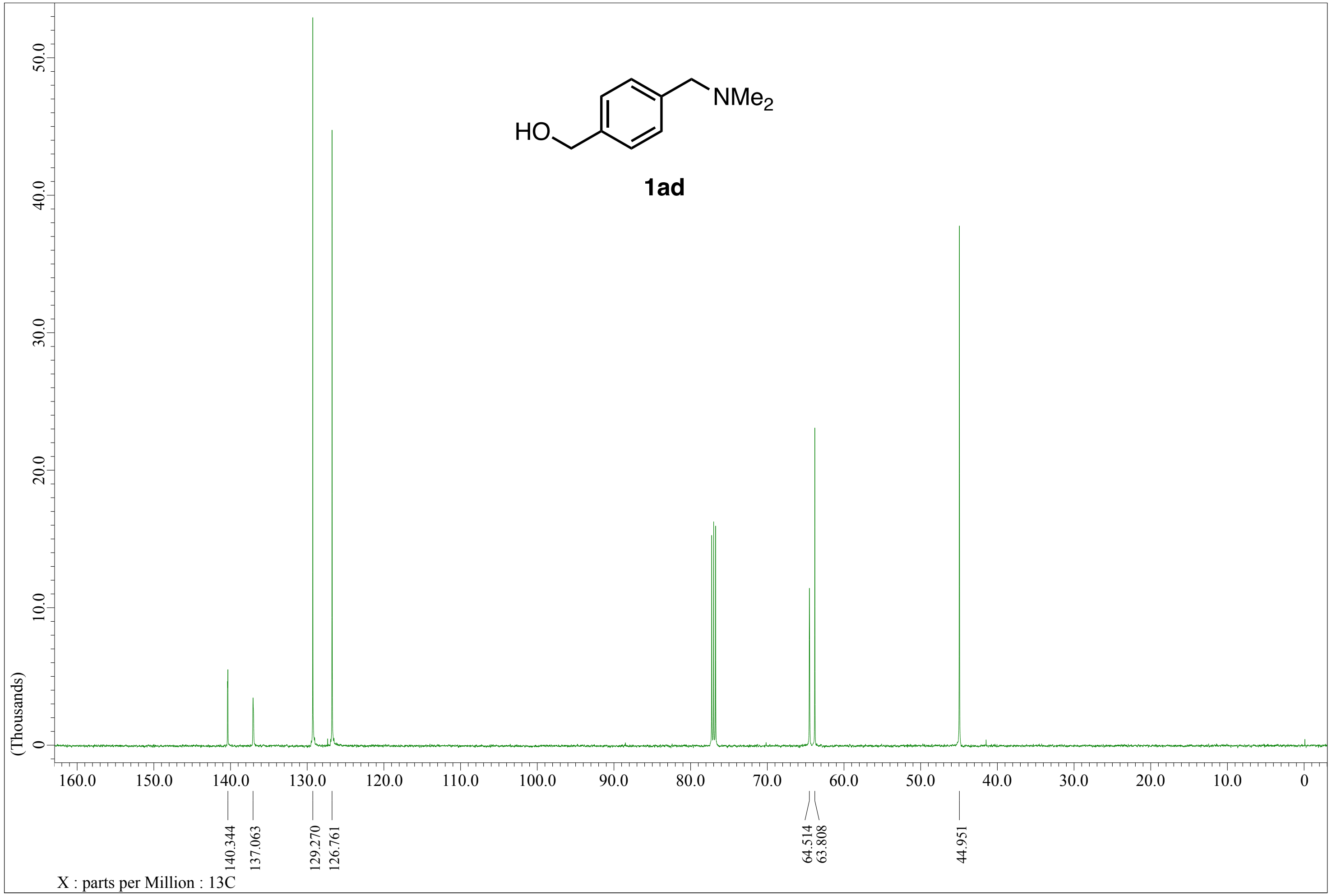
HO
NMe2
1ad
(Thousands)
50.0
40.0
30.0
20.0
10.0
0
160.0 150.0 140.0 130.0 120.0 110.0 100.0 90.0 80.0 70.0 60.0 50.0 40.0 30.0 20.0 10.0 0
140.344
137.063
129.270
126.761
64.514
63.808
44.951
X : parts per Million : 13C

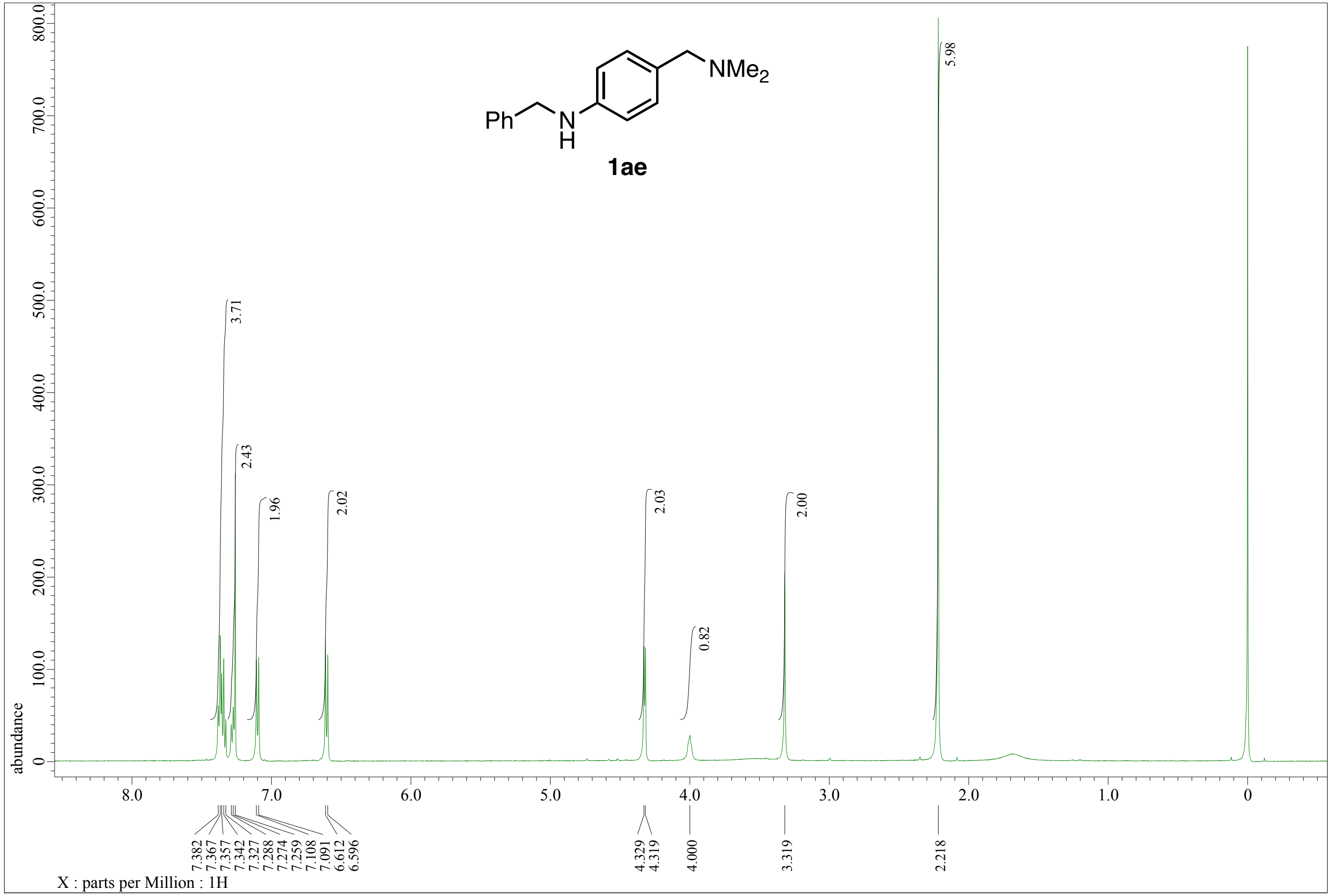

Ph
N
H
NMe2
1ae
abundance
800.0
700.0
600.0
500.0
400.0
300.0
200.0
100.0
0
3.71
2.43
1.96
2.02
2.03
0.82
2.00
5.98
8.0
7.0
6.0
5.0
4.0
3.0
2.0
1.0
0
7.382
7.367
7.357
7.342
7.327
7.288
7.274
7.259
7.108
7.091
6.612
6.596
4.329
4.319
4.000
3.319
2.218
X : parts per Million : 1H

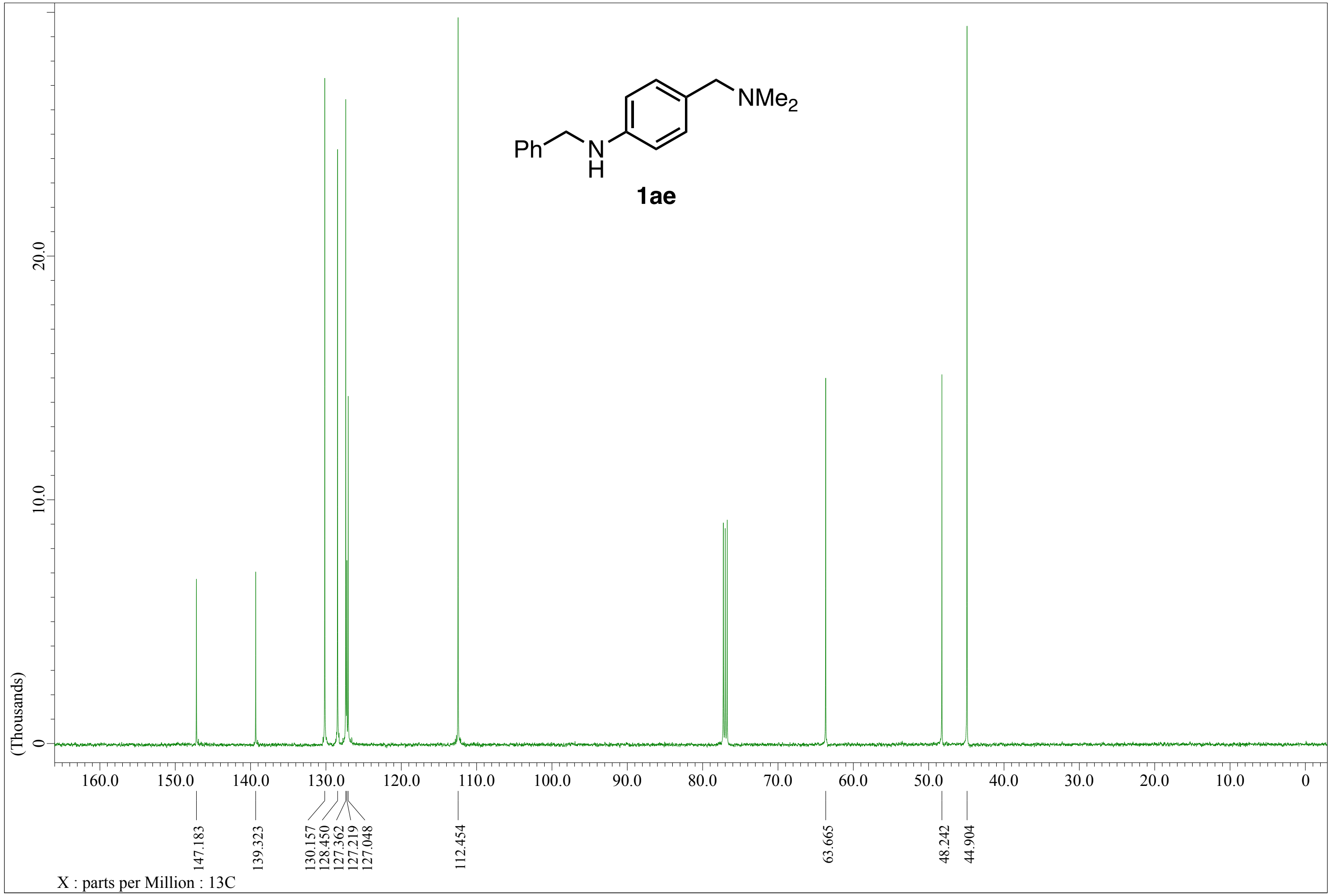

Ph
N
H
NMe2
1ae
(Thousands)
0
10.0
20.0
160.0
150.0
140.0
130.0
120.0
110.0
100.0
90.0
80.0
70.0
60.0
50.0
40.0
30.0
20.0
10.0
0
147.183
139.323
130.157
128.450
127.362
127.219
127.048
112.454
63.665
48.242
44.904
X : parts per Million : 13C

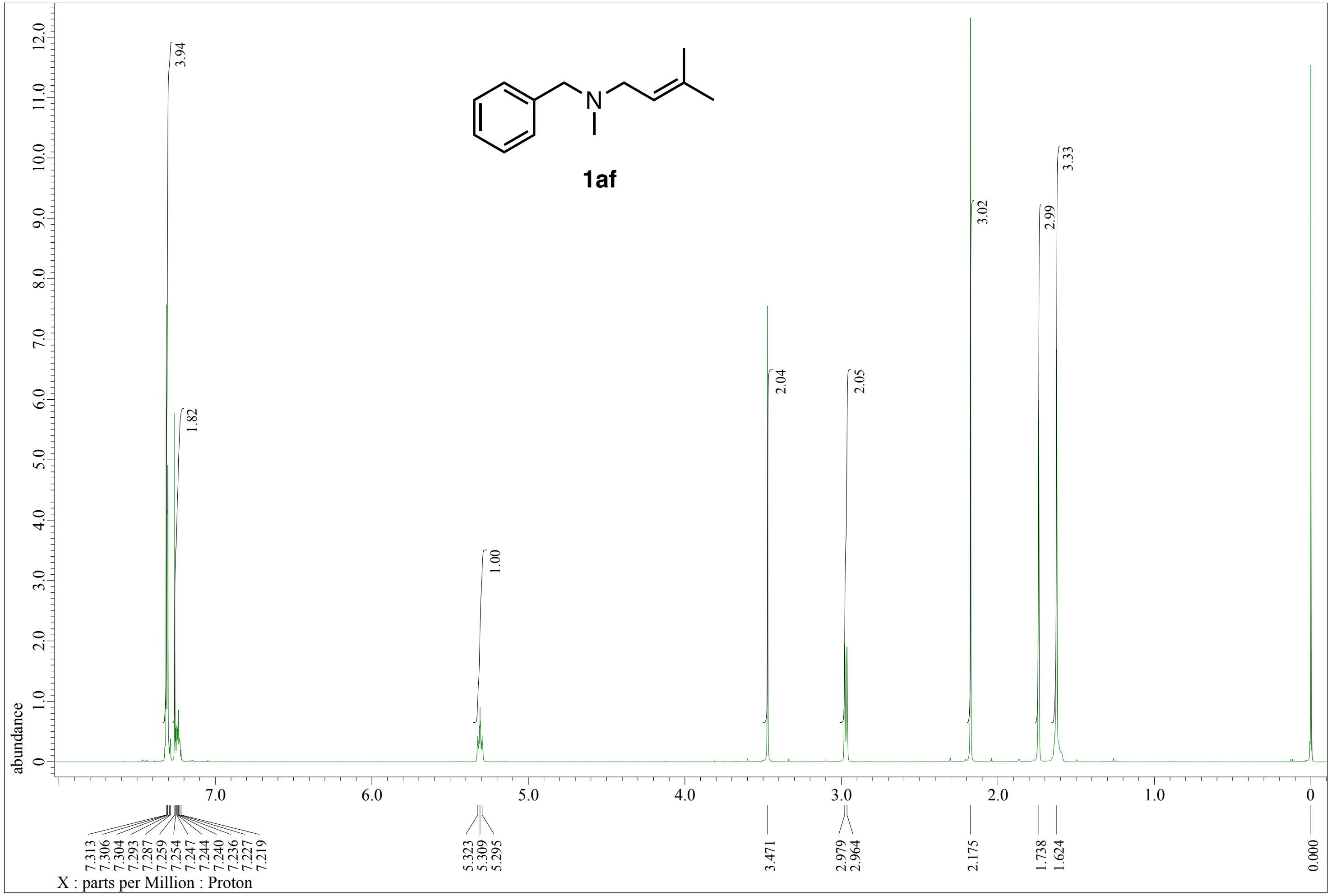
1af
abundance
X : parts per Million : Proton
3.94
1.82
1.00
2.04
2.05
3.02
2.99
3.33
7.313
7.306
7.304
7.293
7.287
7.259
7.254
7.247
7.244
7.240
7.236
7.227
7.219
5.323
5.309
5.295
3.471
2.979
2.964
2.175
1.738
1.624
0.000

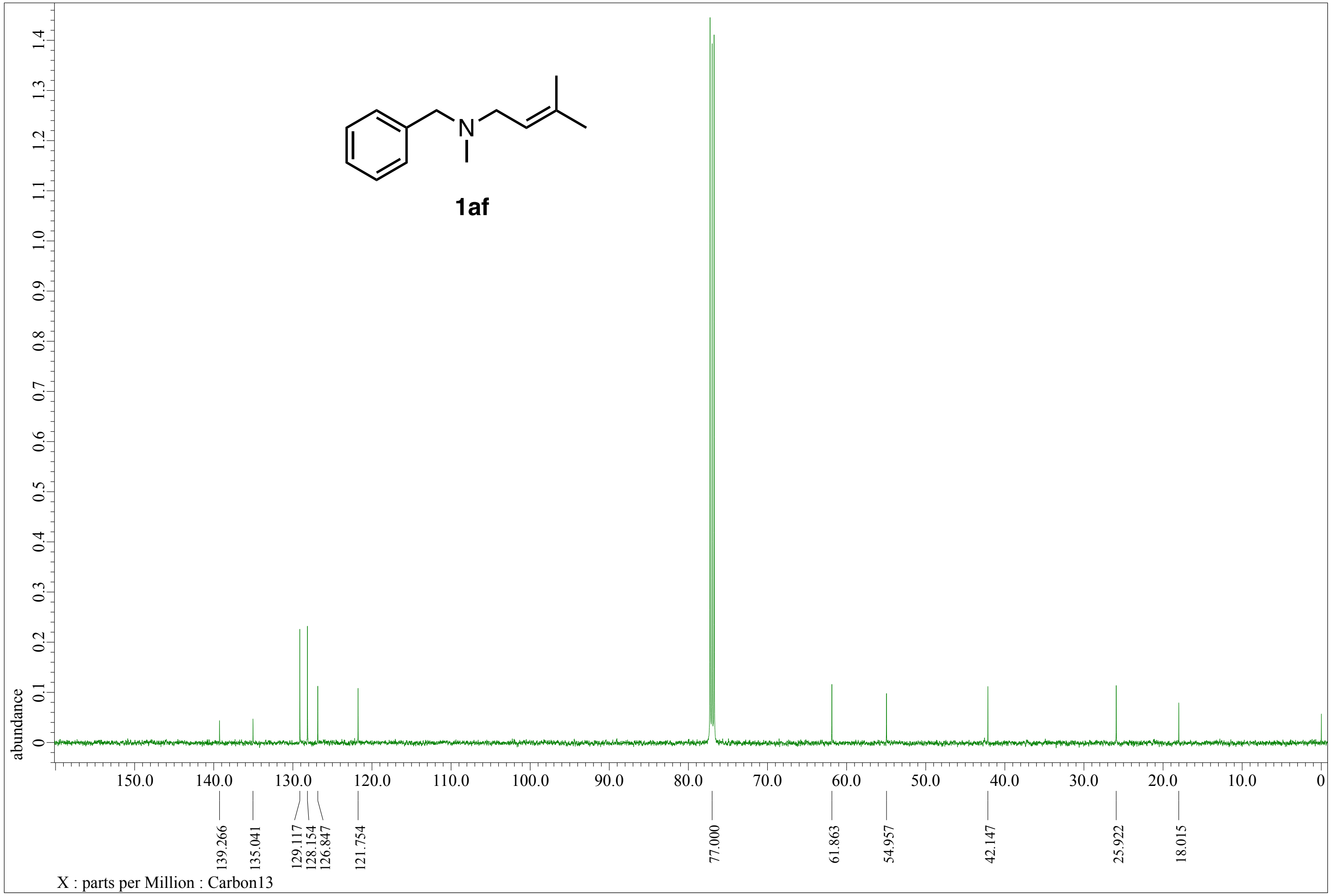
1af
abundance
0
0.1
0.2
0.3
0.4
0.5
0.6
0.7
0.8
0.9
1.0
1.1
1.2
1.3
1.4
150.0
140.0
130.0
120.0
110.0
100.0
90.0
80.0
70.0
60.0
50.0
40.0
30.0
20.0
10.0
0
139.266
135.041
129.117
128.154
126.847
121.754
77.000
61.863
54.957
42.147
25.922
18.015
X : parts per Million : Carbon13

09

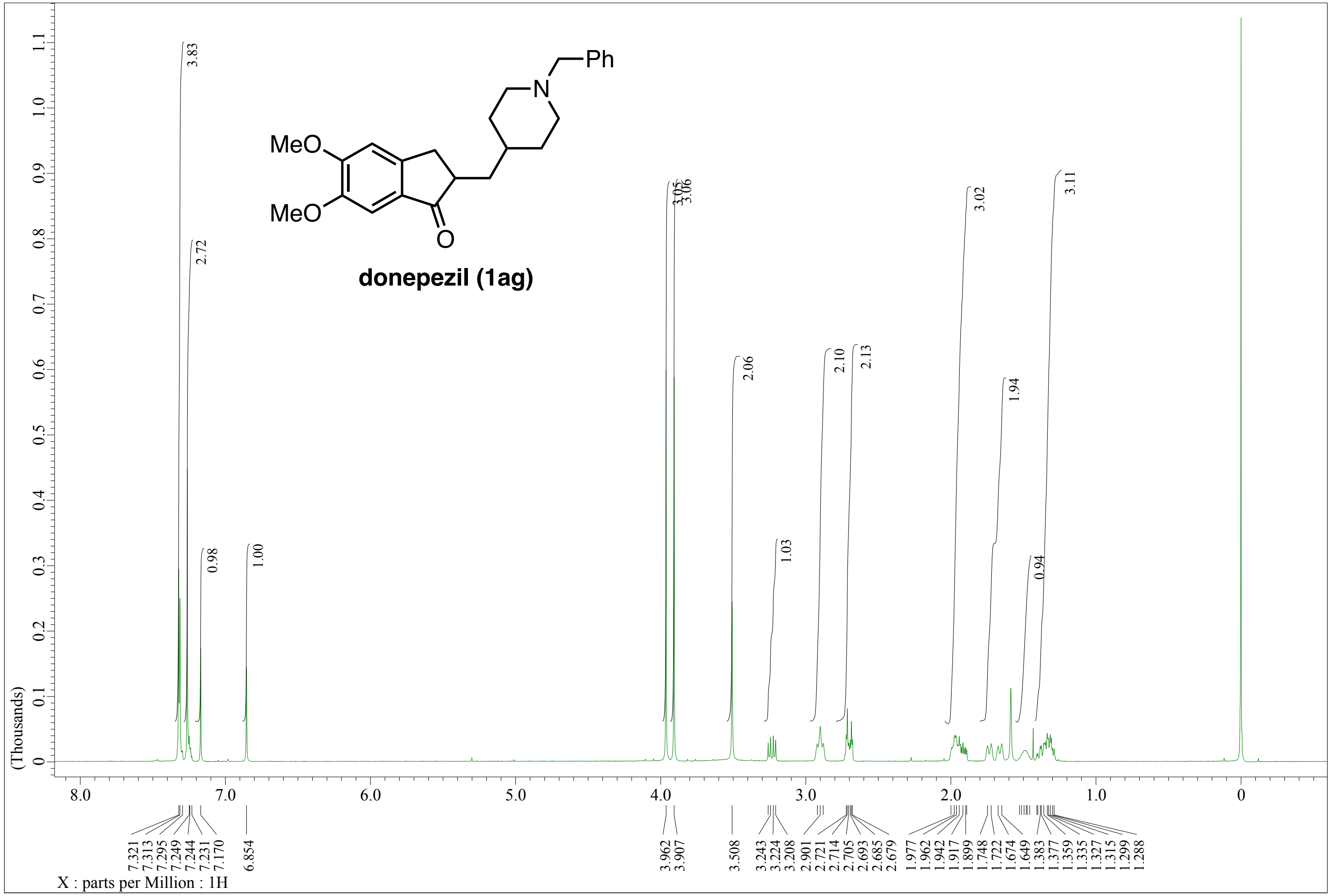

MeO
MeO
O
N
Ph
donepezil (1ag)
X : parts per Million : 1H
(Thousands)

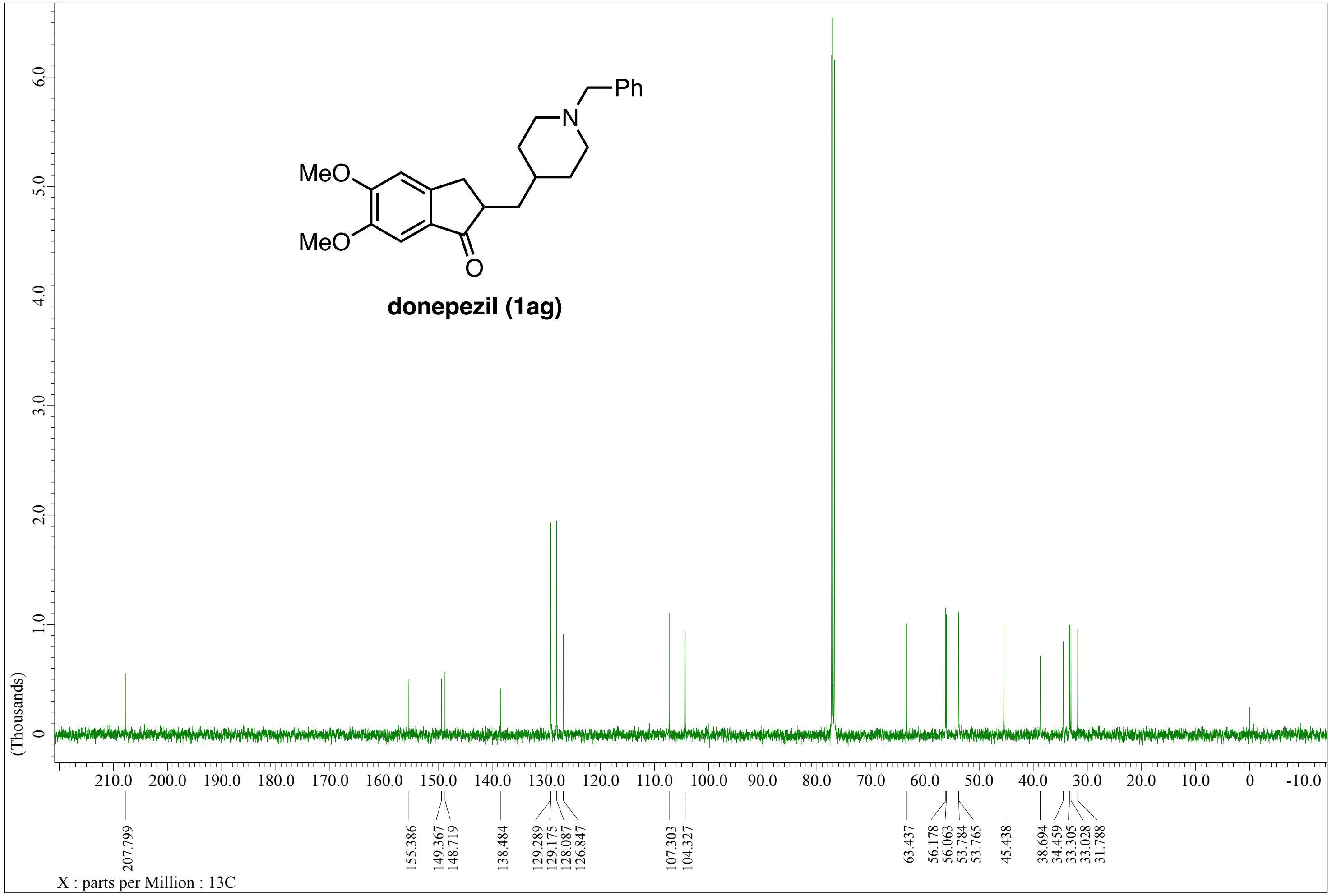
MeO
MeO
O
N
Ph
donepezil (1ag)
(Thousands)
X : parts per Million : 13C
207.799
155.386
149.367
148.719
138.484
129.289
129.175
128.087
126.847
107.303
104.327
63.437
56.178
56.063
53.784
53.765
45.438
38.694
34.459
33.305
33.028
31.788

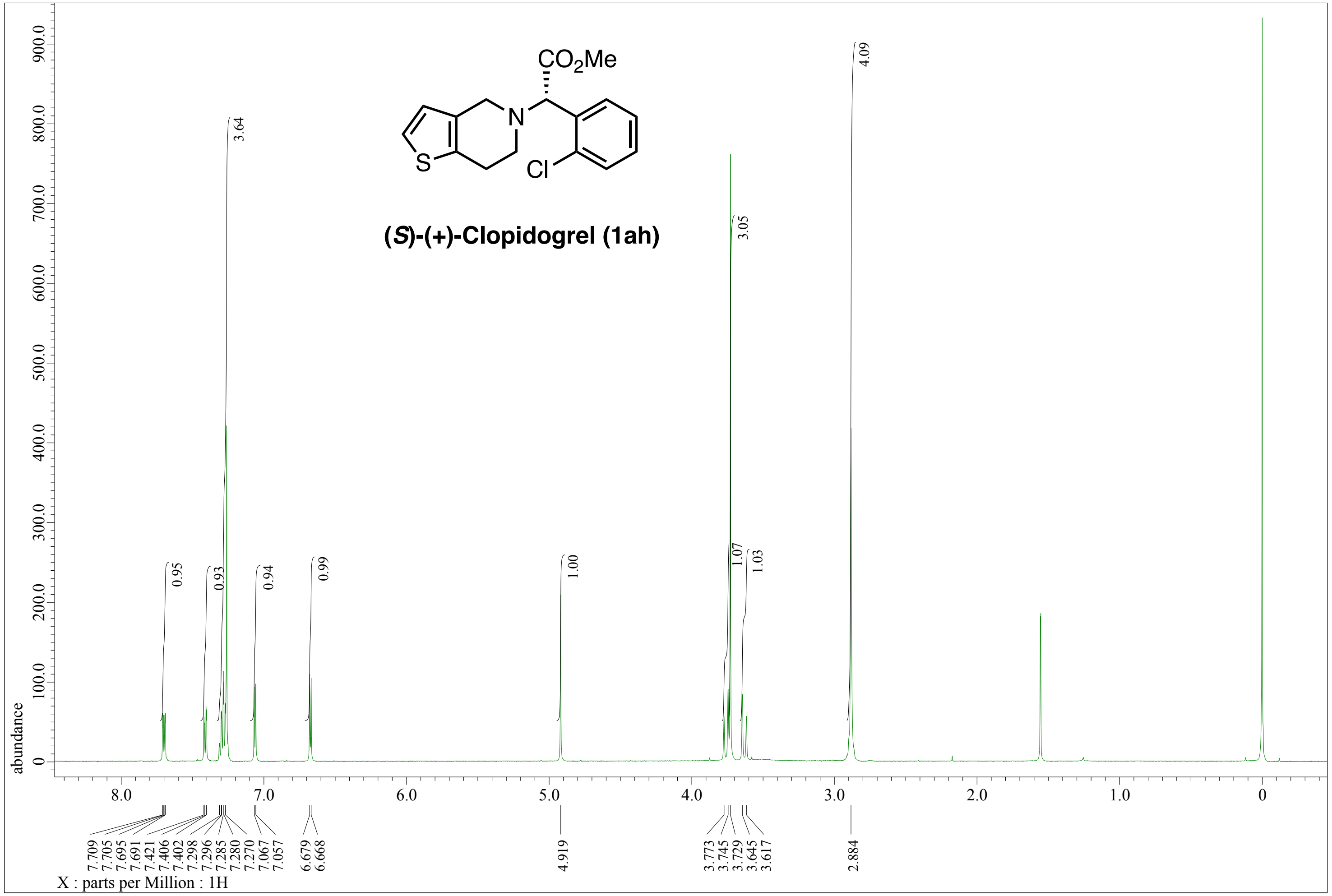

CO2Me
N
S
Cl
(S)-(+)-Clopidogrel (1ah)
abundance
0
100.0
200.0
300.0
400.0
500.0
600.0
700.0
800.0
900.0
0.95
0.93
3.64
0.94
0.99
1.00
1.07
3.05
1.03
4.09
8.0
7.0
6.0
5.0
4.0
3.0
2.0
1.0
0
7.709
7.705
7.695
7.691
7.421
7.406
7.402
7.298
7.296
7.285
7.280
7.270
7.067
7.057
6.679
6.668
4.919
3.773
3.745
3.729
3.645
3.617
2.884
X : parts per Million : 1H

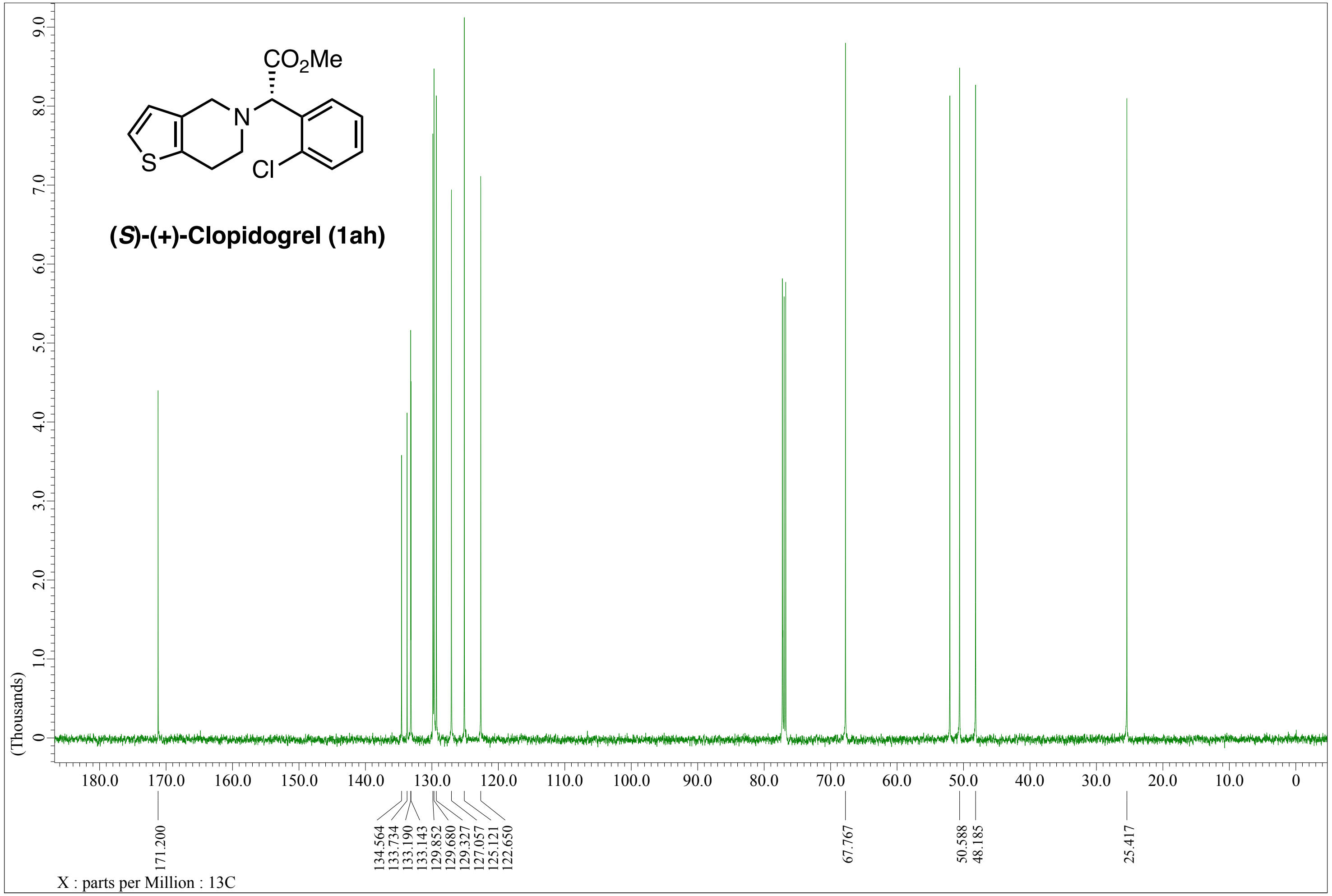
CO2Me
N
S
Cl
(S)-(+)-Clopidogrel (1ah)
(Thousands)
X : parts per Million : 13C
171.200
134.564
133.734
133.190
133.143
129.852
129.680
129.327
127.057
125.121
122.650
67.767
50.588
48.185
25.417

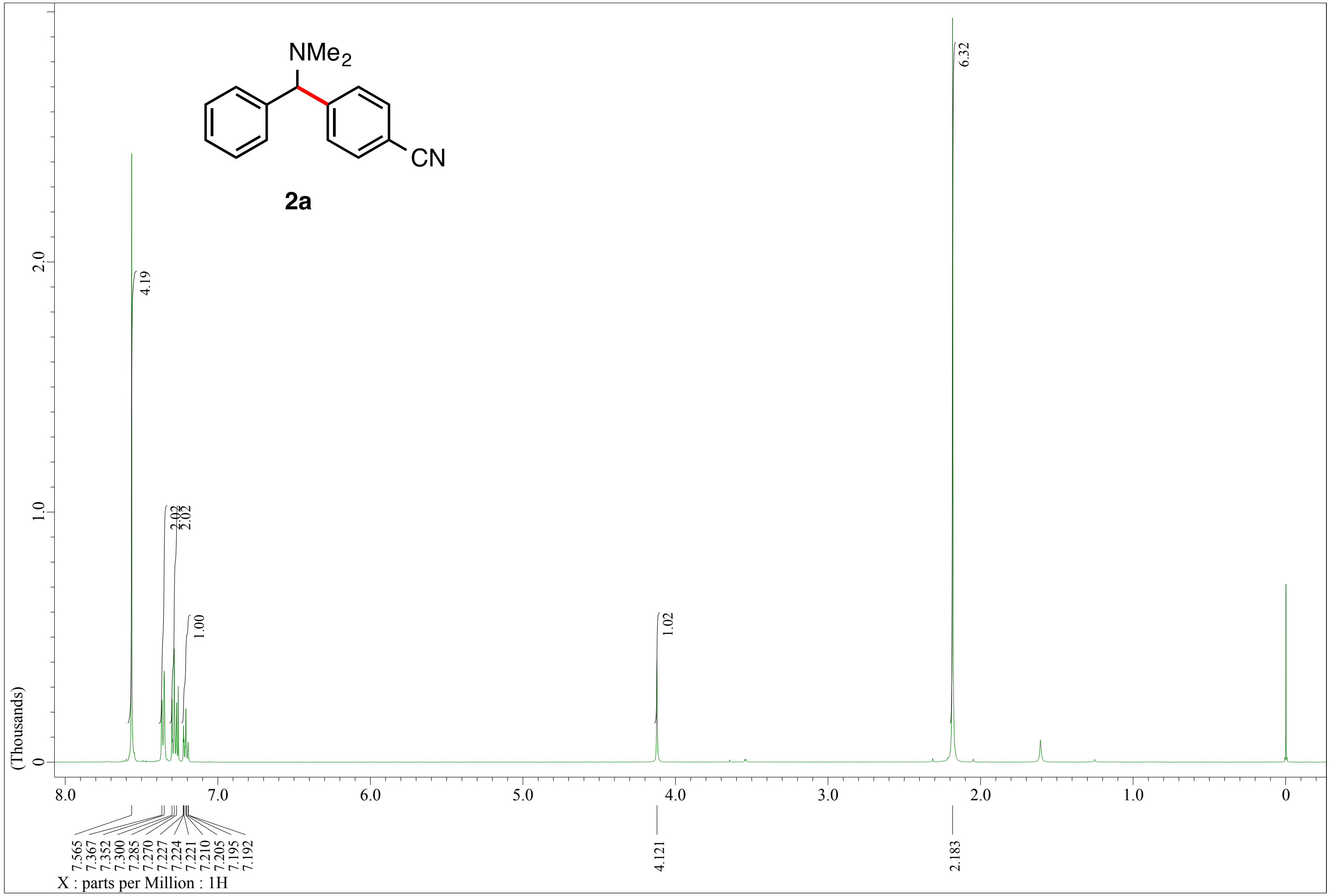
NMe2
CN
2a
(Thousands)
X : parts per Million : 1H
7.565
7.367
7.352
7.300
7.285
7.270
7.227
7.224
7.221
7.210
7.205
7.195
7.192
4.121
2.183
4.19
2.02
2.02
1.00
1.02
6.32

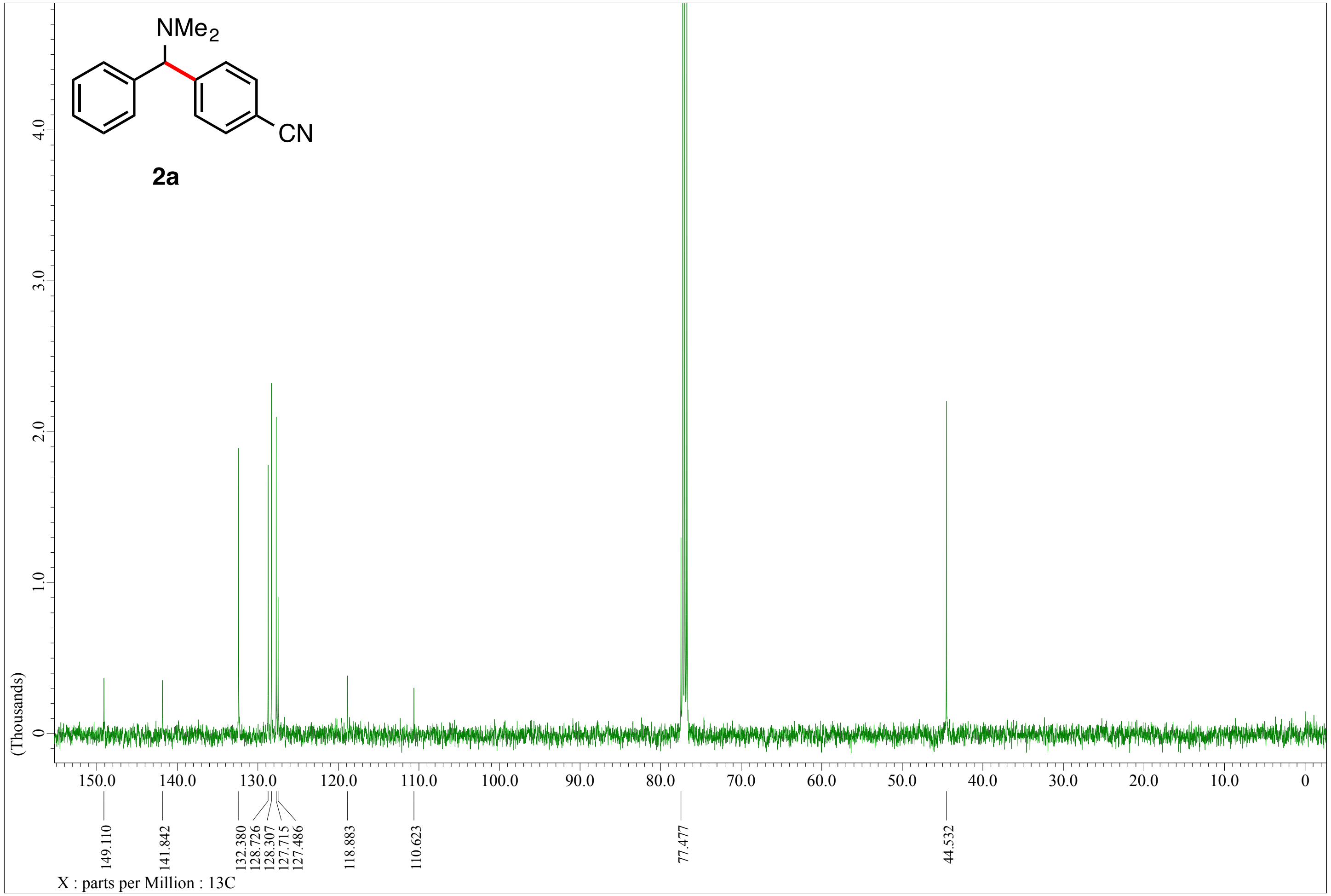
NMe2
CN
2a
(Thousands)
4.0
3.0
2.0
1.0
0
150.0
140.0
130.0
120.0
110.0
100.0
90.0
80.0
70.0
60.0
50.0
40.0
30.0
20.0
10.0
0
149.110
141.842
132.380
128.726
128.307
127.715
127.486
118.883
110.623
77.477
44.532
X : parts per Million : 13C

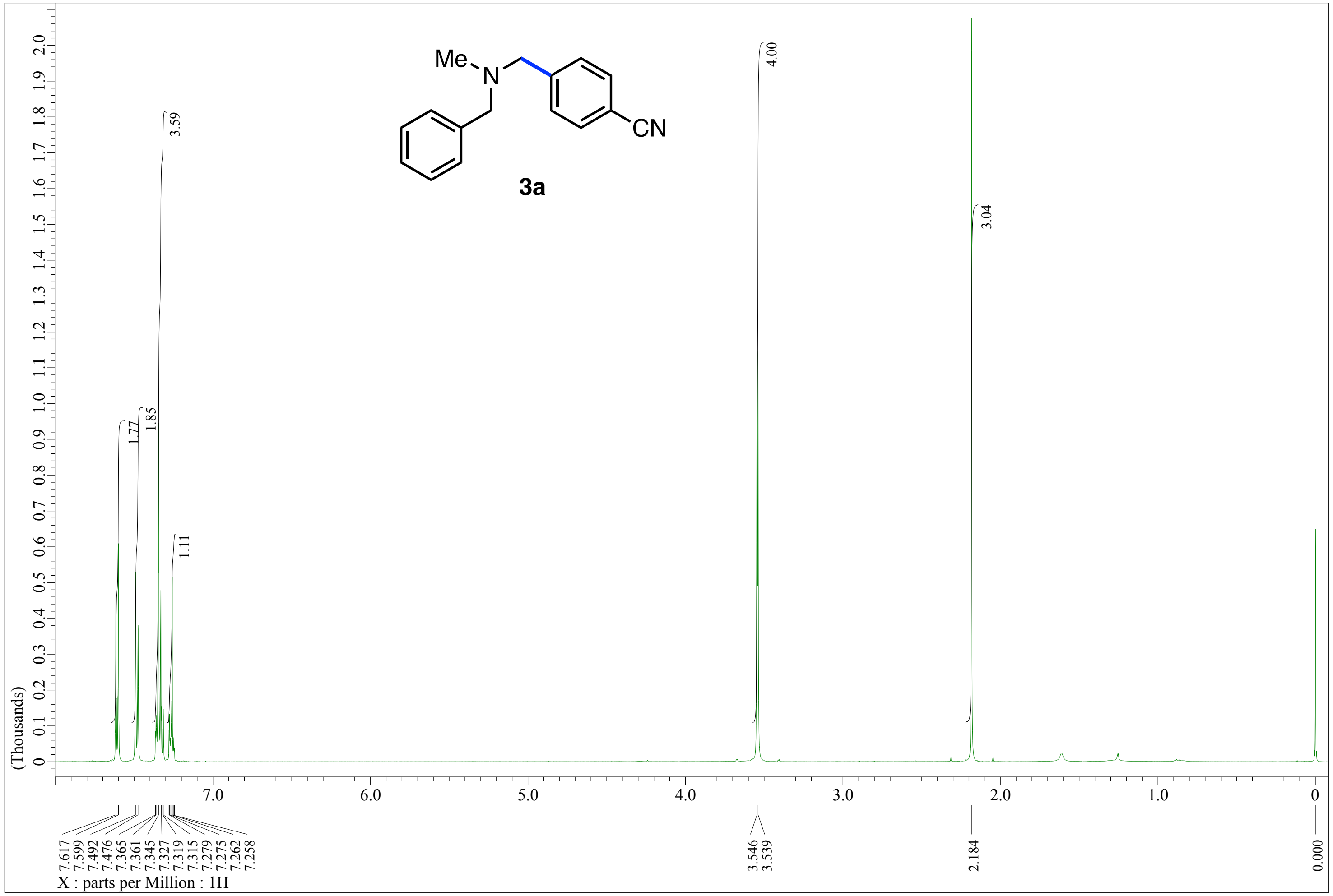
Me
N
CN
3a
4.00
3.59
3.04
1.77
1.85
1.11
(Thousands)
7.0
6.0
5.0
4.0
3.0
2.0
1.0
0
7.617
7.599
7.492
7.476
7.365
7.361
7.345
7.327
7.319
7.315
7.279
7.275
7.262
7.258
3.546
3.539
2.184
0.000
X : parts per Million : 1H

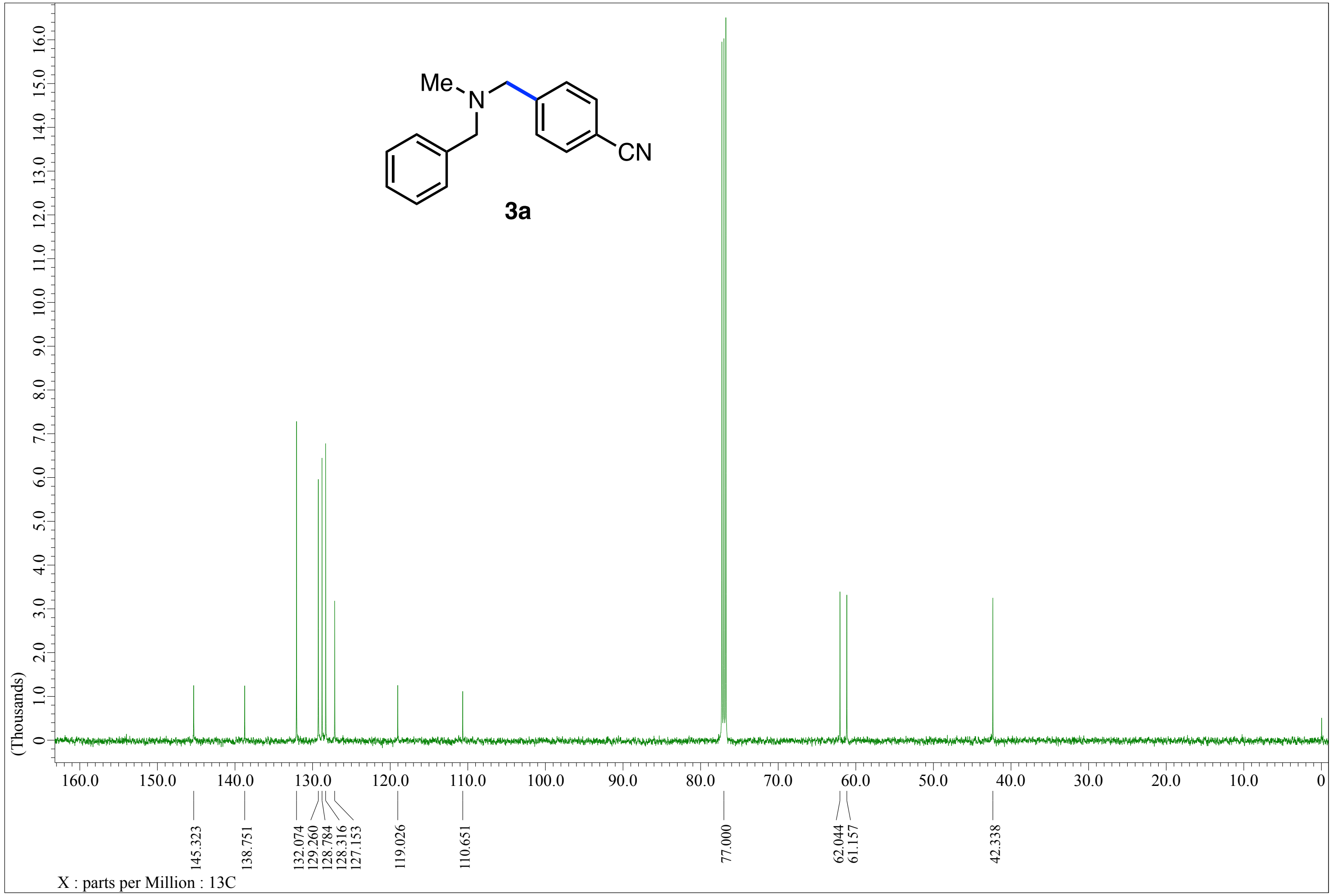
Me
N
CN
3a
(Thousands)
X : parts per Million : 13C
145.323
138.751
132.074
129.260
128.784
128.316
127.153
119.026
110.651
77.000
62.044
61.157
42.338

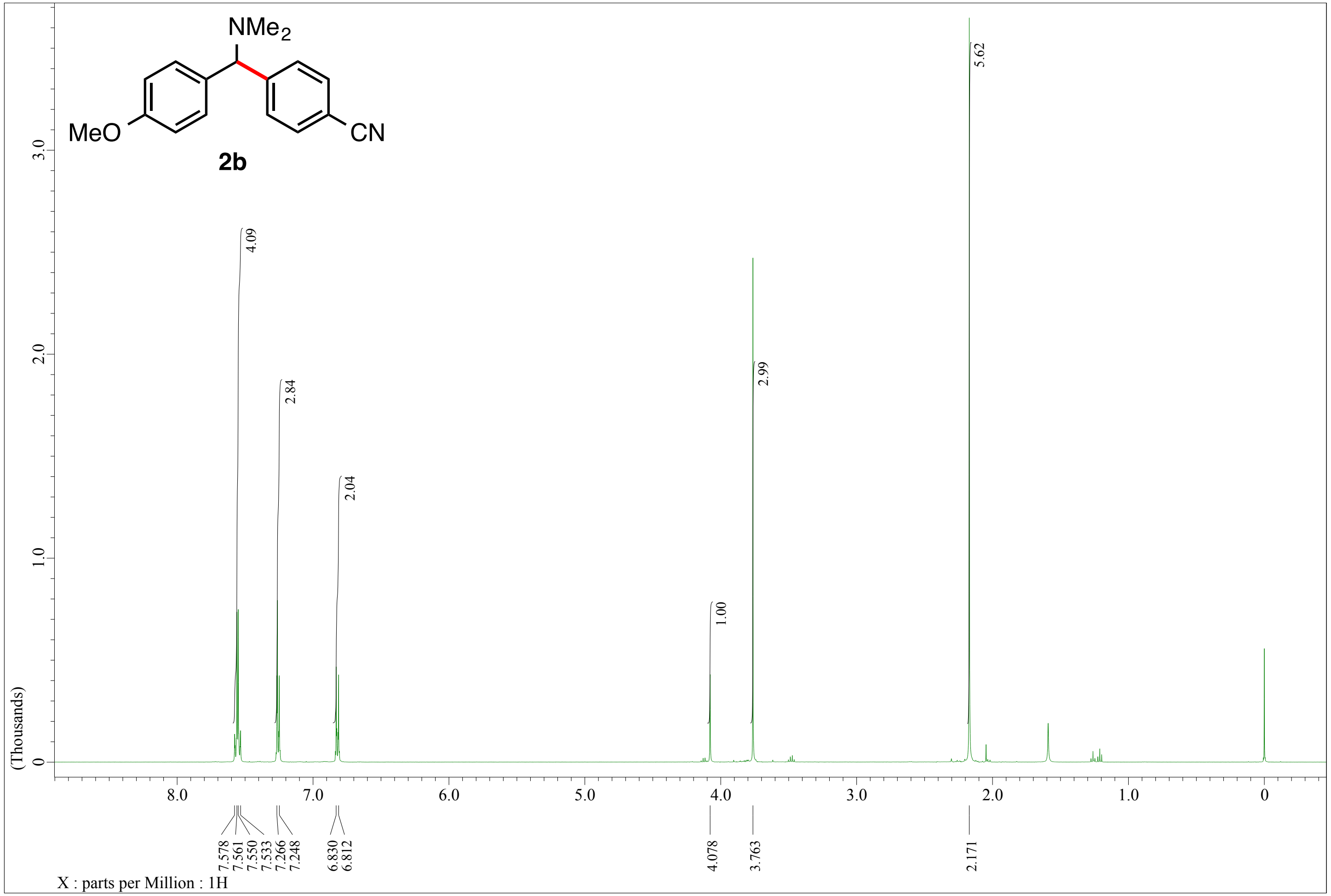
NMe2
MeO
CN
2b
5.62
4.09
2.84
2.04
1.00
2.99
(Thousands)
3.0
2.0
1.0
0
8.0
7.0
6.0
5.0
4.0
3.0
2.0
1.0
0
7.578
7.561
7.550
7.533
7.266
7.248
6.830
6.812
4.078
3.763
2.171
X : parts per Million : 1H

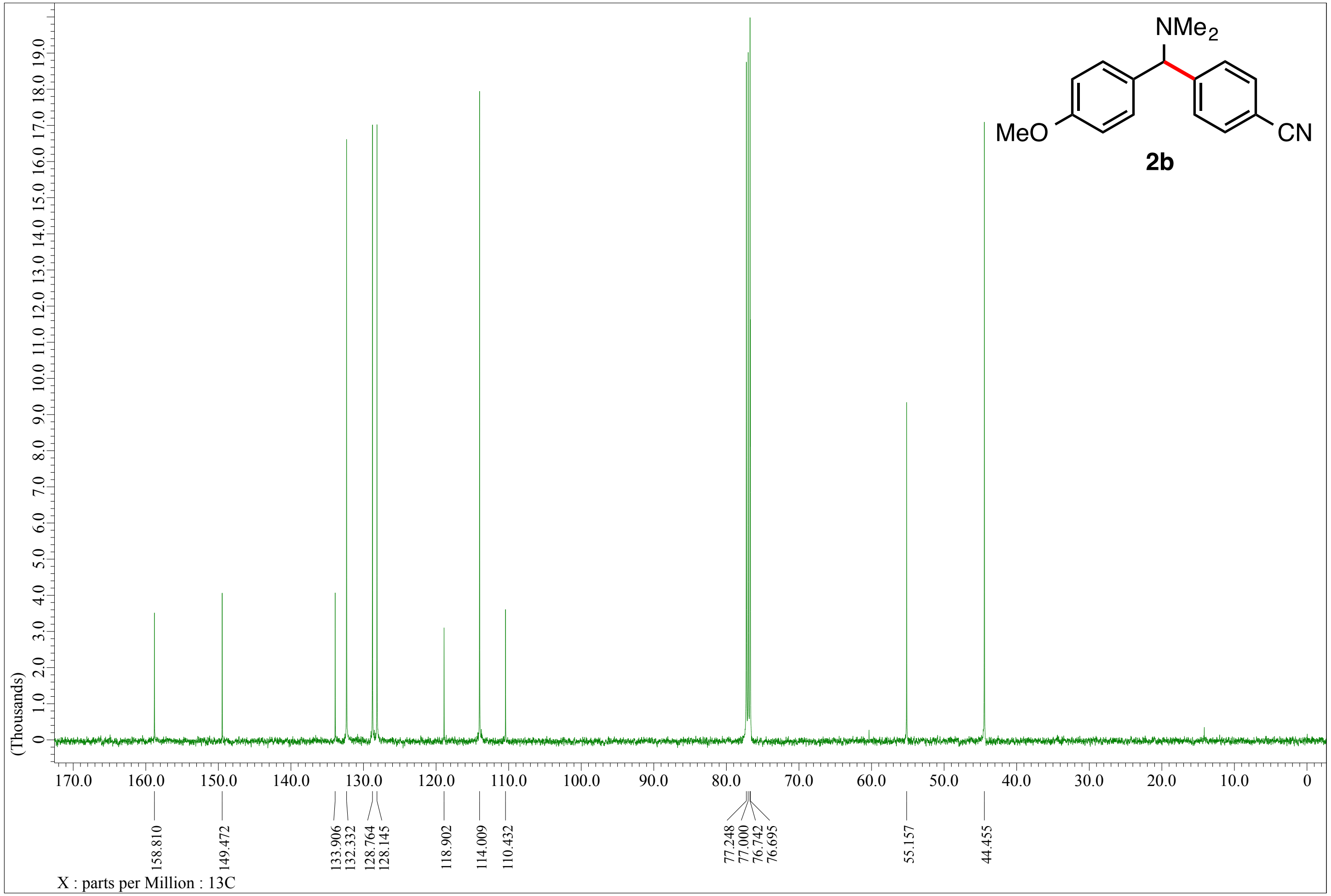
NMe2
MeO
CN
2b
(Thousands)
158.810
149.472
133.906
132.332
128.764
128.145
118.902
114.009
110.432
77.248
77.000
76.742
76.695
55.157
44.455
X : parts per Million : 13C

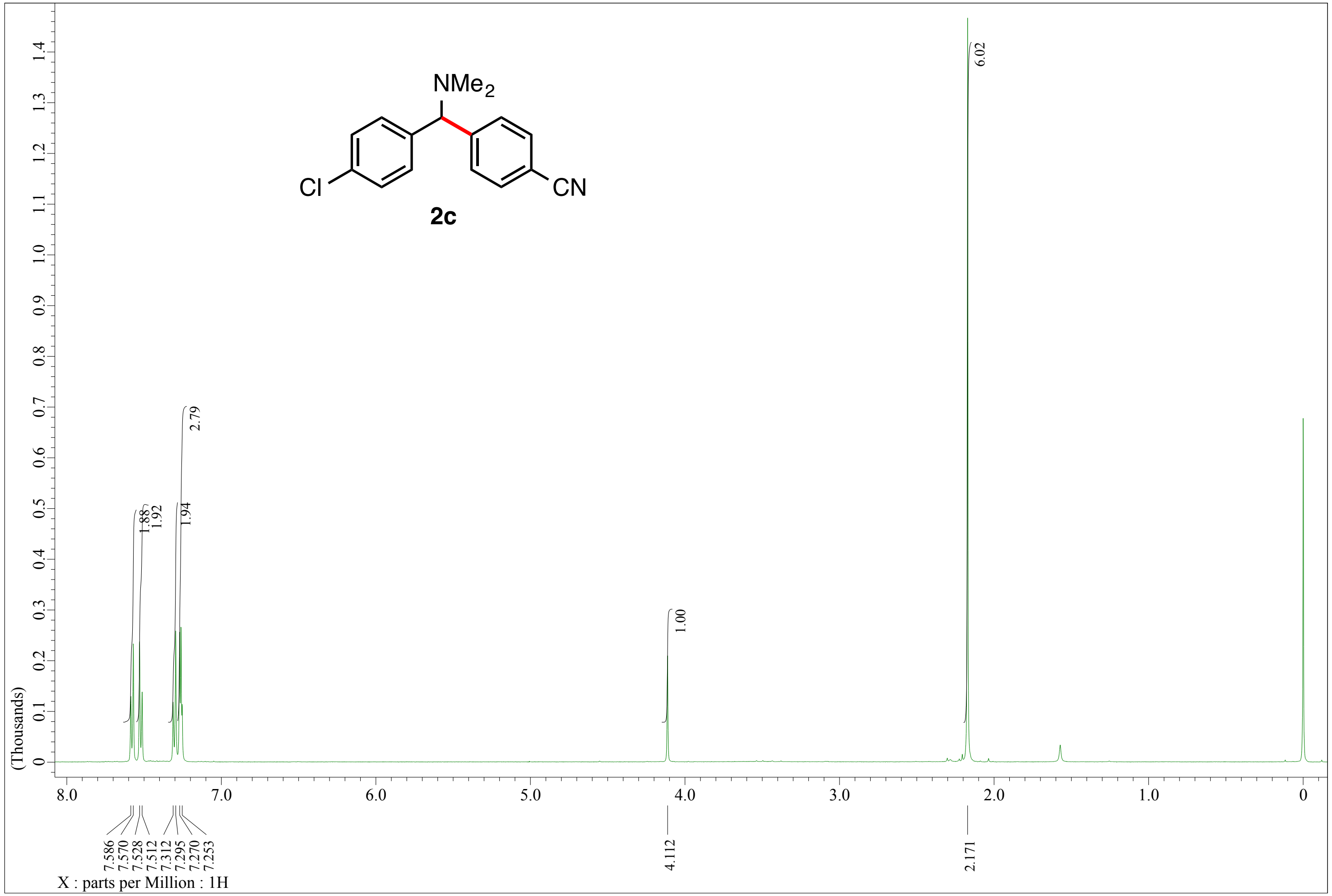
NMe2
Cl
CN
2c
6.02
2.79
1.88
1.92
1.94
1.00
(Thousands)
8.0
7.0
6.0
5.0
4.0
3.0
2.0
1.0
0
7.586
7.570
7.528
7.512
7.312
7.295
7.270
7.253
4.112
2.171
X : parts per Million : 1H

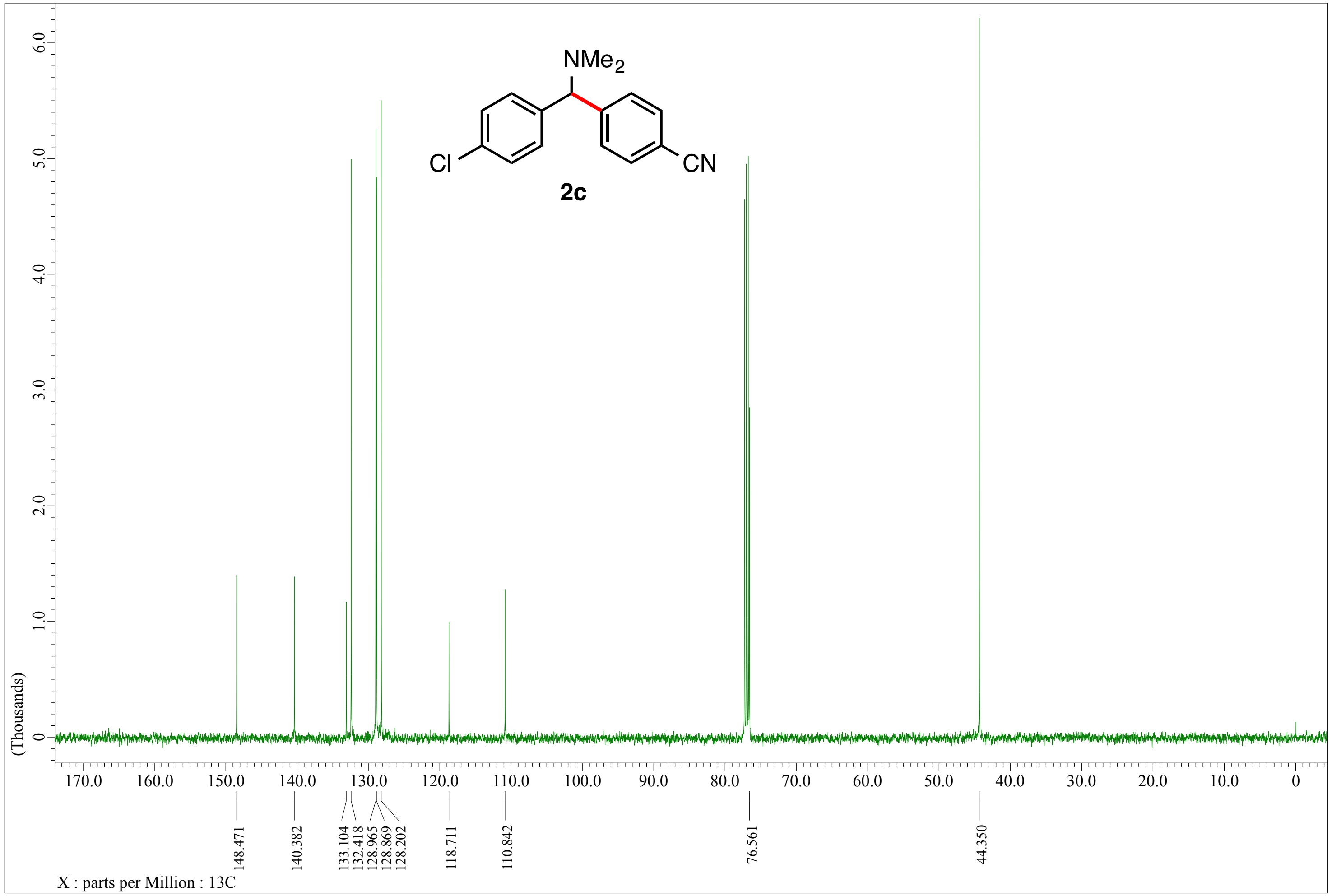
NMe2
Cl
CN
2c
(Thousands)
6.0
5.0
4.0
3.0
2.0
1.0
0
170.0
160.0
150.0
140.0
130.0
120.0
110.0
100.0
90.0
80.0
70.0
60.0
50.0
40.0
30.0
20.0
10.0
0
148.471
140.382
133.104
132.418
128.965
128.869
128.202
118.711
110.842
76.561
44.350
X : parts per Million : 13C

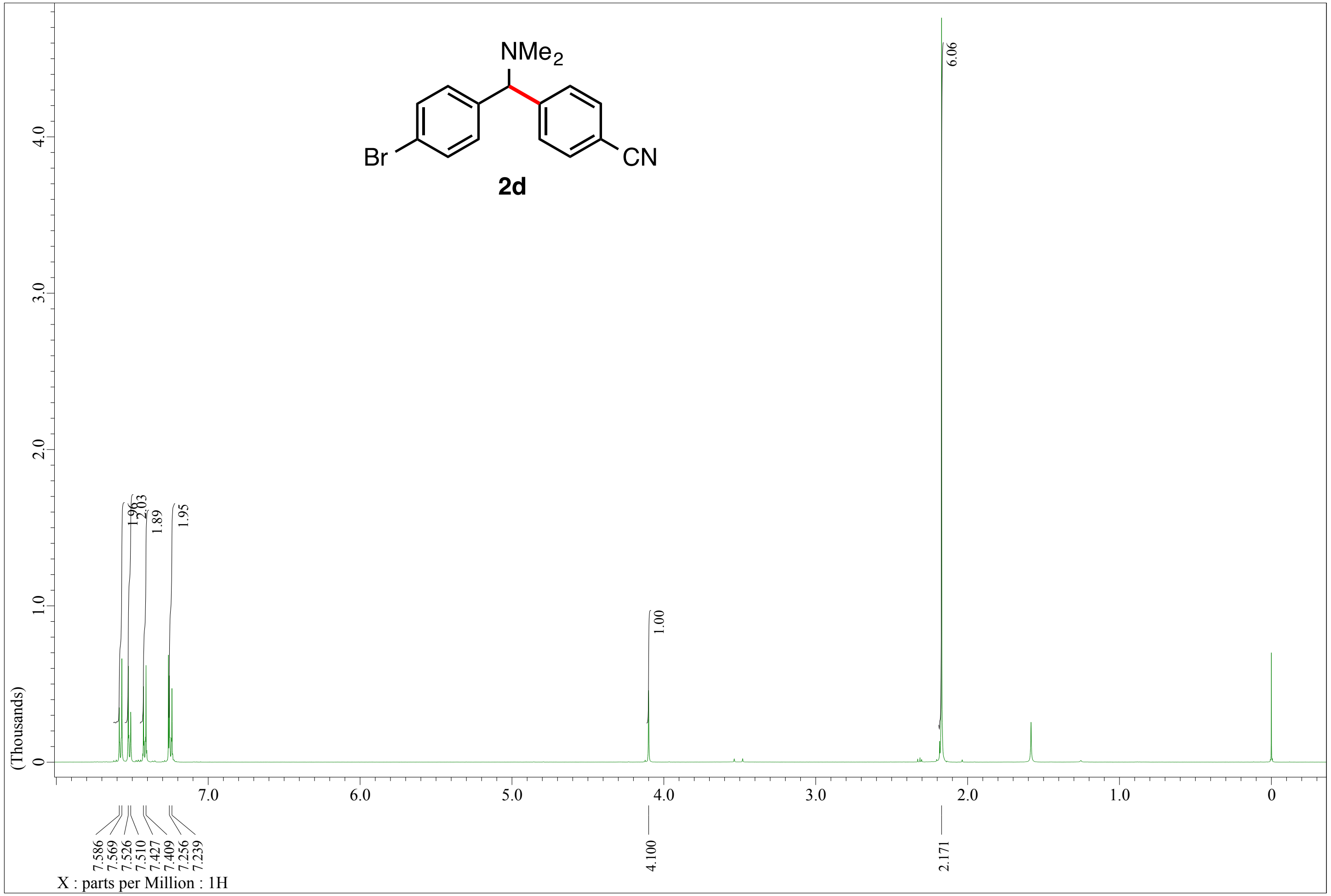
NMe2
Br
CN
2d
6.06
1.96
2.03
1.89
1.95
1.00
(Thousands)
4.0
3.0
2.0
1.0
0
7.0
6.0
5.0
4.0
3.0
2.0
1.0
0
7.586
7.569
7.526
7.510
7.427
7.409
7.256
7.239
4.100
2.171
X : parts per Million : 1H

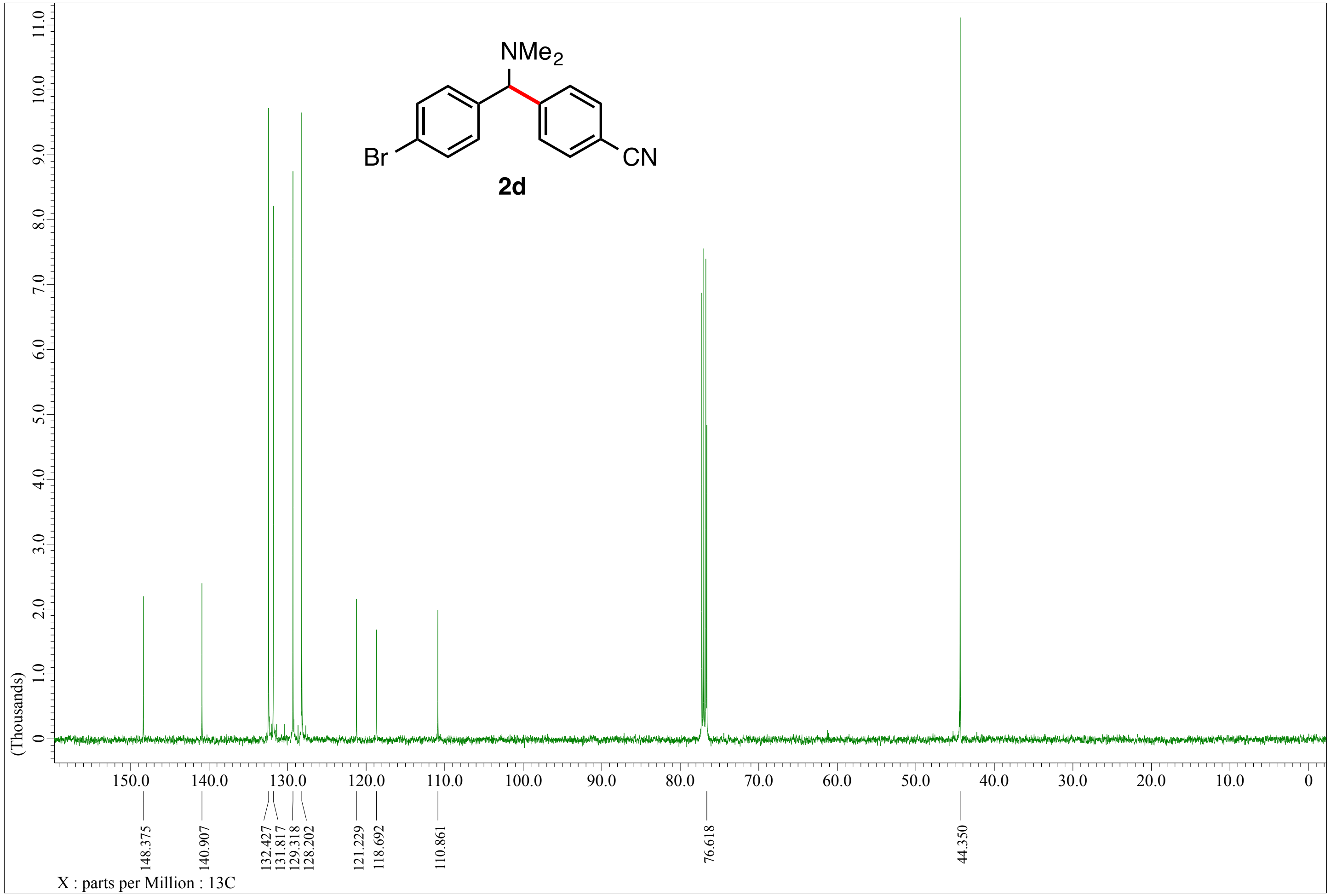
NMe2
Br
CN
2d
(Thousands)
X : parts per Million : 13C
148.375
140.907
132.427
131.817
129.318
128.202
121.229
118.692
110.861
76.618
44.350

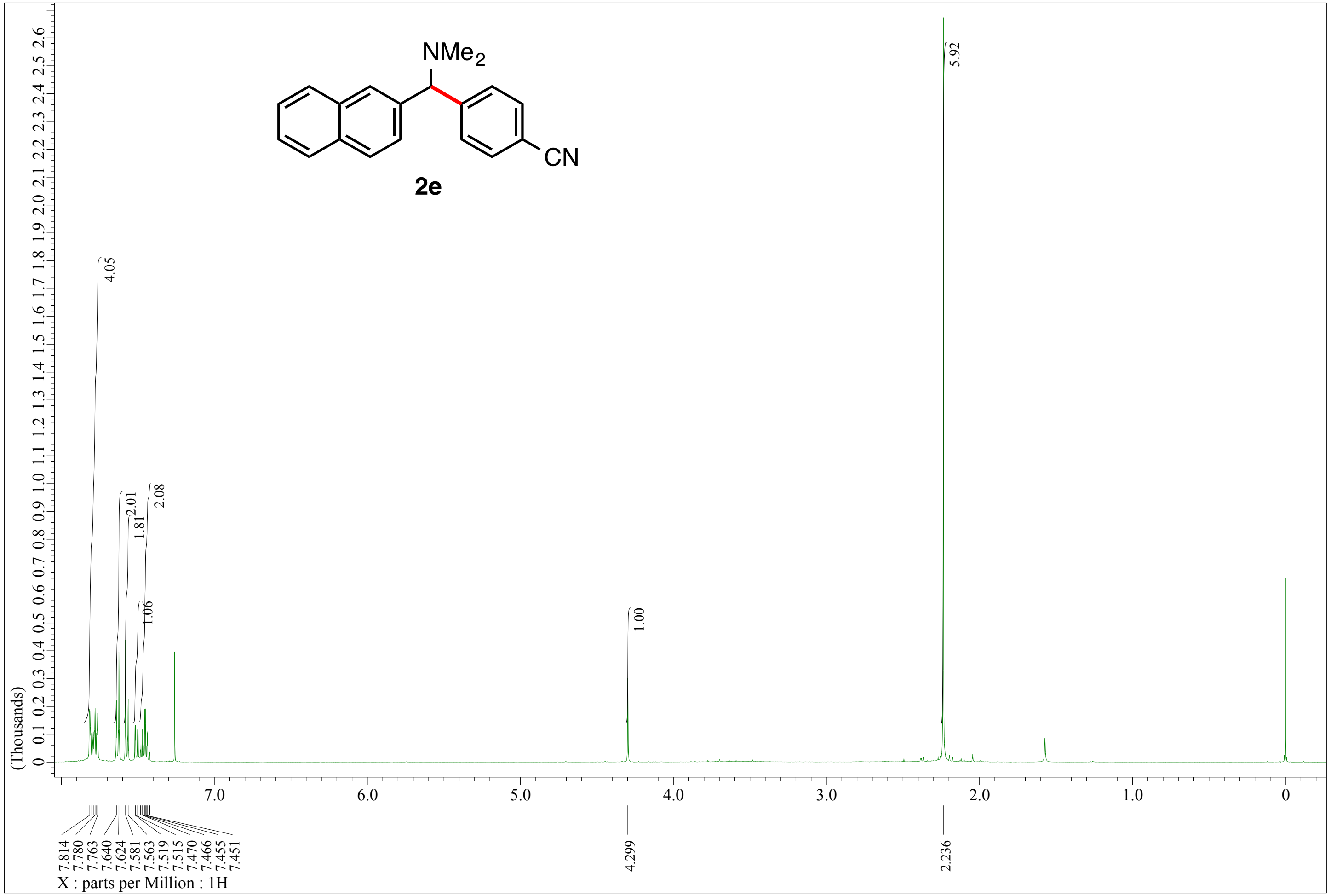
NMe2
CN
2e
(Thousands)
4.05
2.01
1.81
2.08
1.06
1.00
5.92
7.0
6.0
5.0
4.0
3.0
2.0
1.0
0
7.814
7.780
7.763
7.640
7.624
7.581
7.563
7.519
7.515
7.470
7.466
7.455
7.451
4.299
2.236
X : parts per Million : 1H

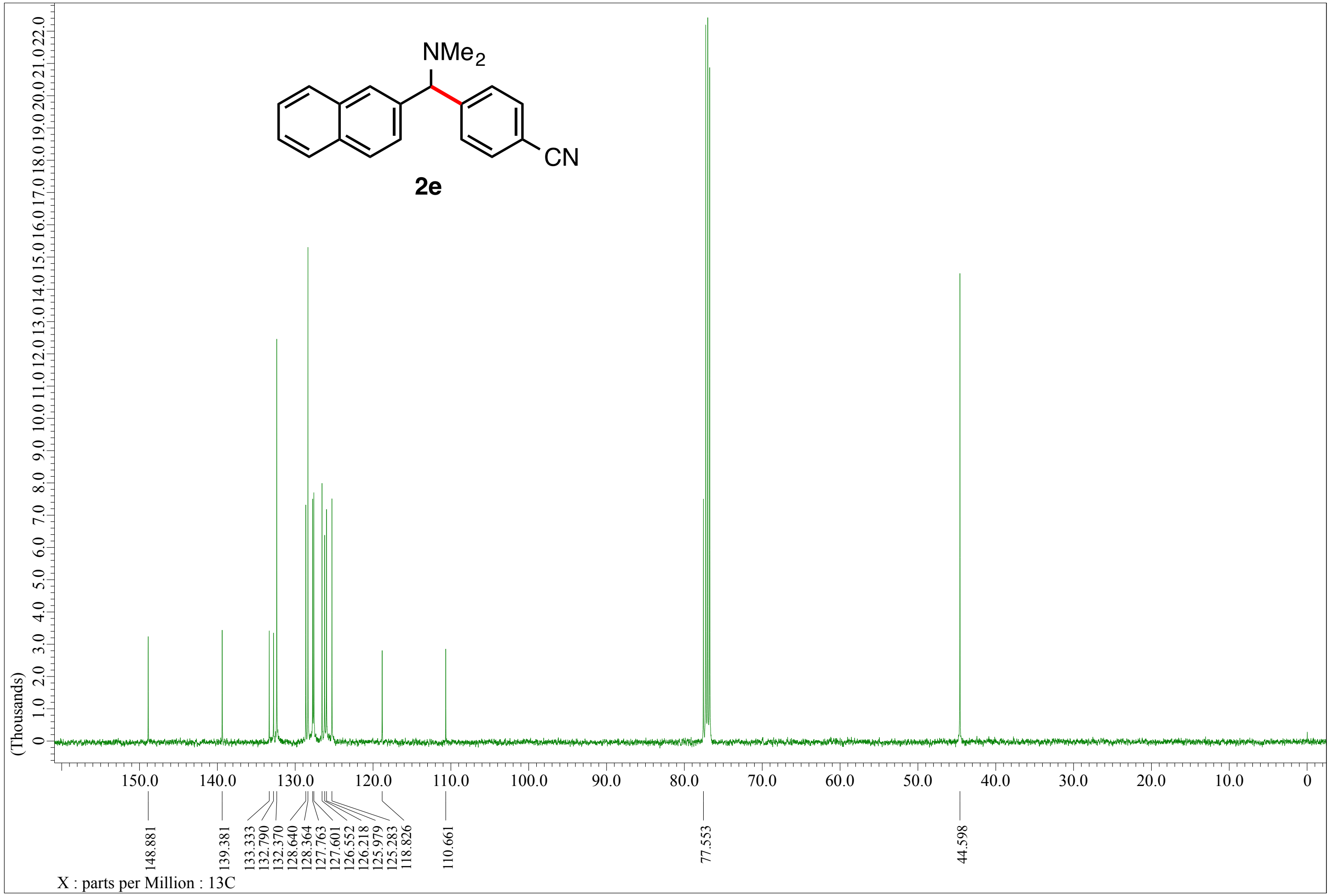
NMe2
CN
2e
(Thousands)
X : parts per Million : 13C
148.881
139.381
133.333
132.790
132.370
128.640
128.364
127.763
127.601
126.552
126.218
125.979
125.283
118.826
110.661
77.553
44.598

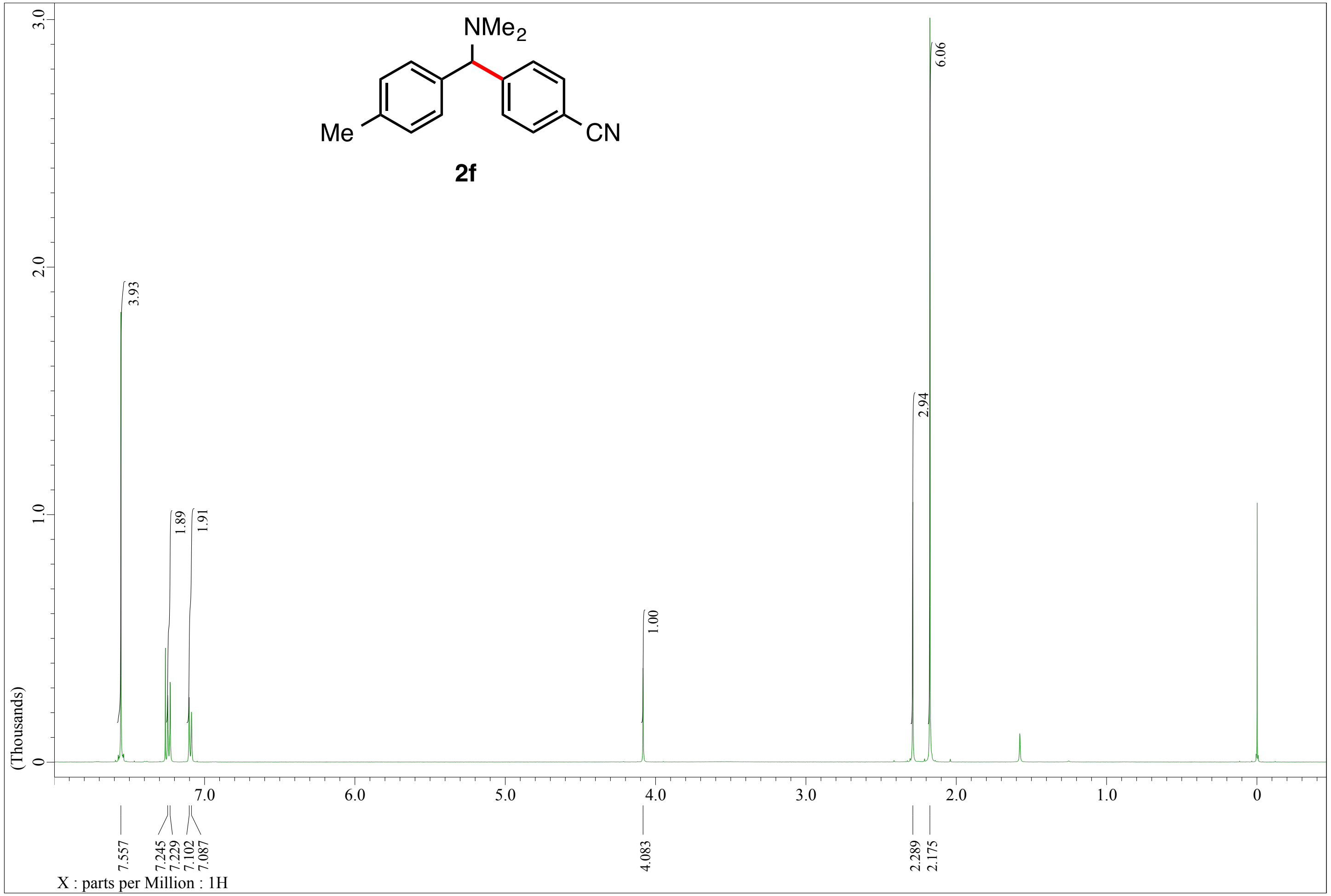
NMe2
Me
CN
2f
3.93
1.89
1.91
1.00
2.94
6.06
(Thousands)
3.0
2.0
1.0
0
7.0
6.0
5.0
4.0
3.0
2.0
1.0
0
7.557
7.245
7.229
7.102
7.087
4.083
2.289
2.175
X : parts per Million : 1H

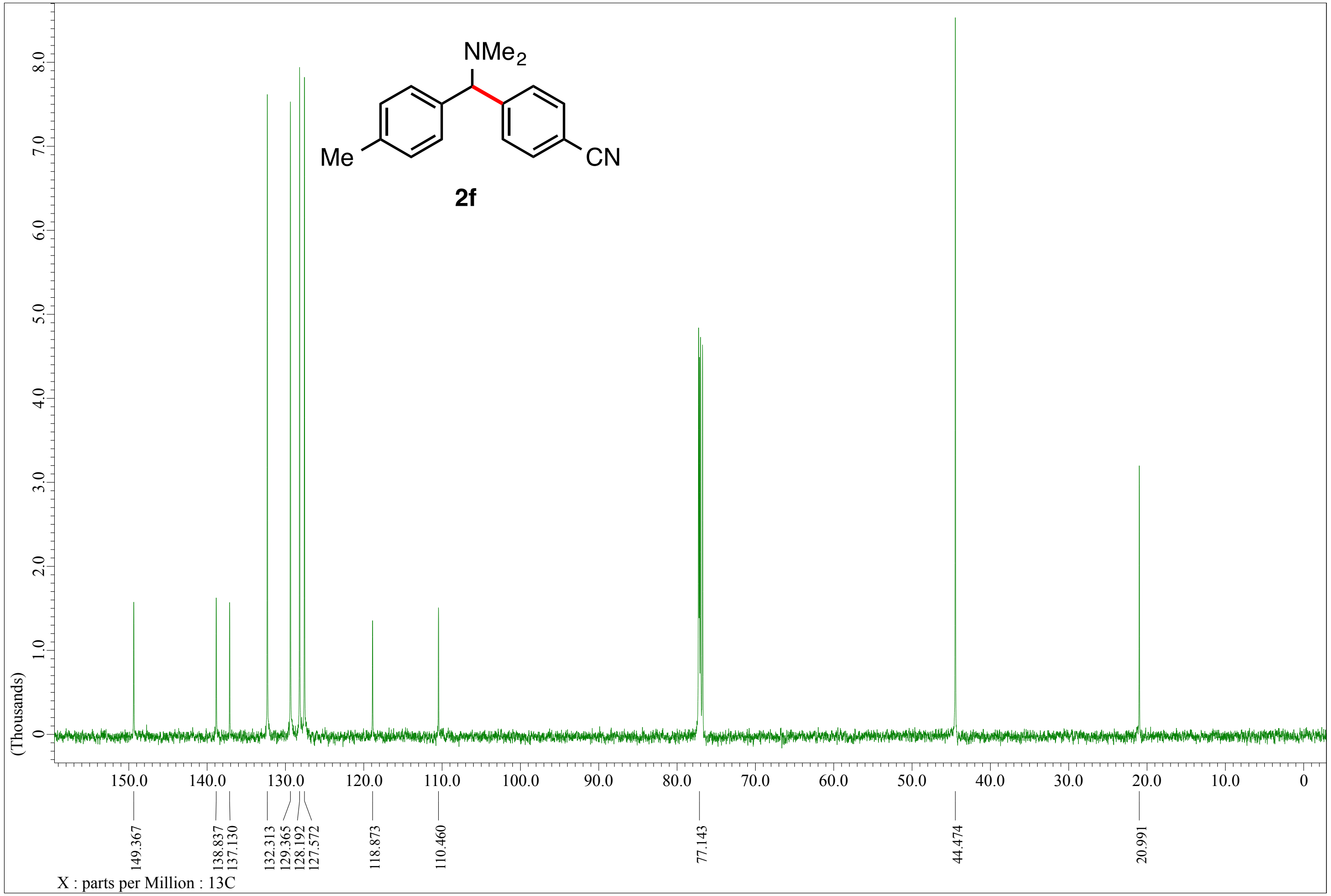
NMe2
Me
CN
2f
(Thousands)
8.0
7.0
6.0
5.0
4.0
3.0
2.0
1.0
0
150.0
140.0
130.0
120.0
110.0
100.0
90.0
80.0
70.0
60.0
50.0
40.0
30.0
20.0
10.0
0
149.367
138.837
137.130
132.313
129.365
128.192
127.572
118.873
110.460
77.143
44.474
20.991
X : parts per Million : 13C

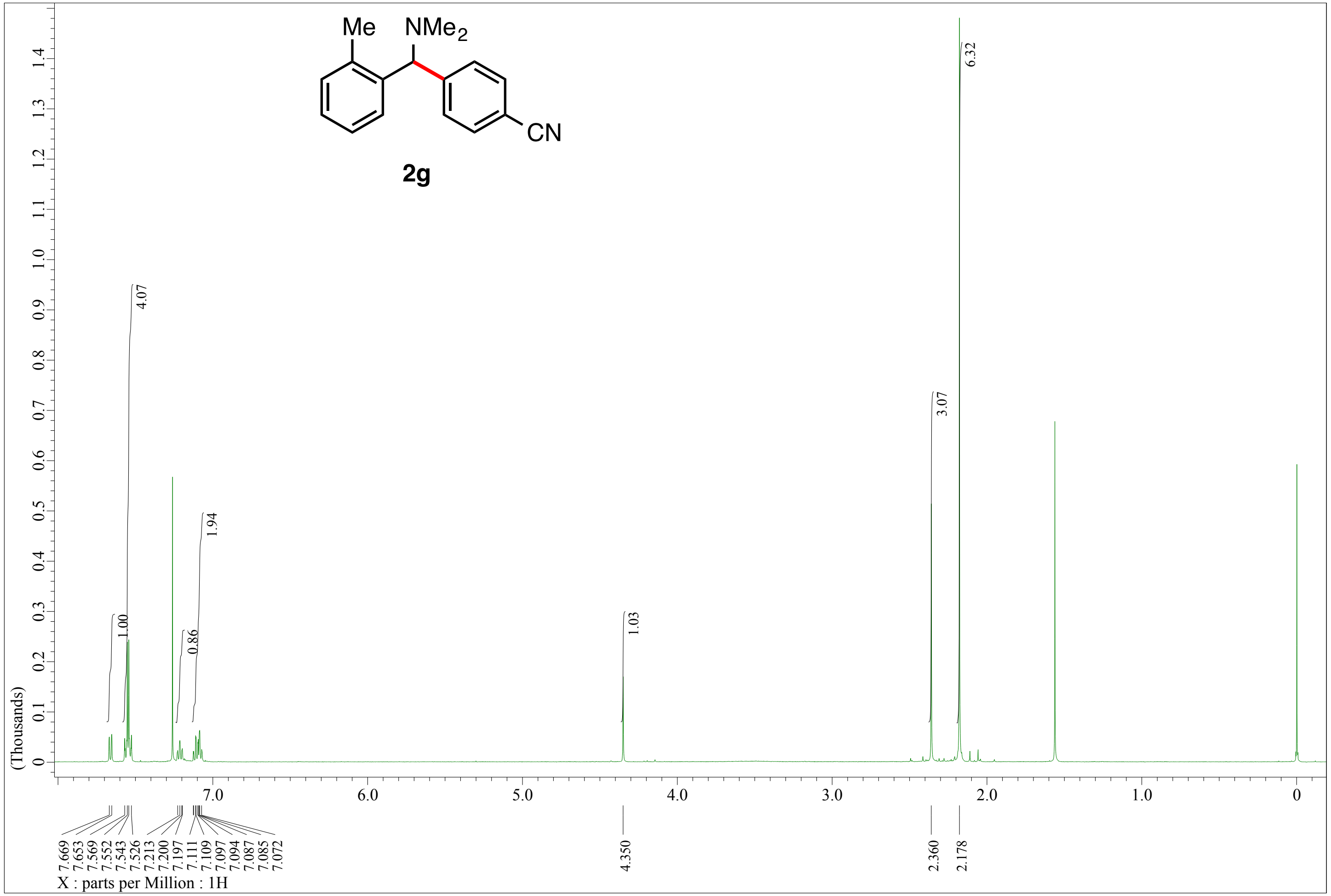
Me
NMe2
CN
2g
(Thousands)
4.07
1.00
0.86
1.94
1.03
3.07
6.32
7.669 7.653 7.569 7.552 7.543 7.526 7.213 7.200 7.197 7.111 7.109 7.097 7.094 7.087 7.085 7.072
4.350
2.360
2.178
X : parts per Million : 1H

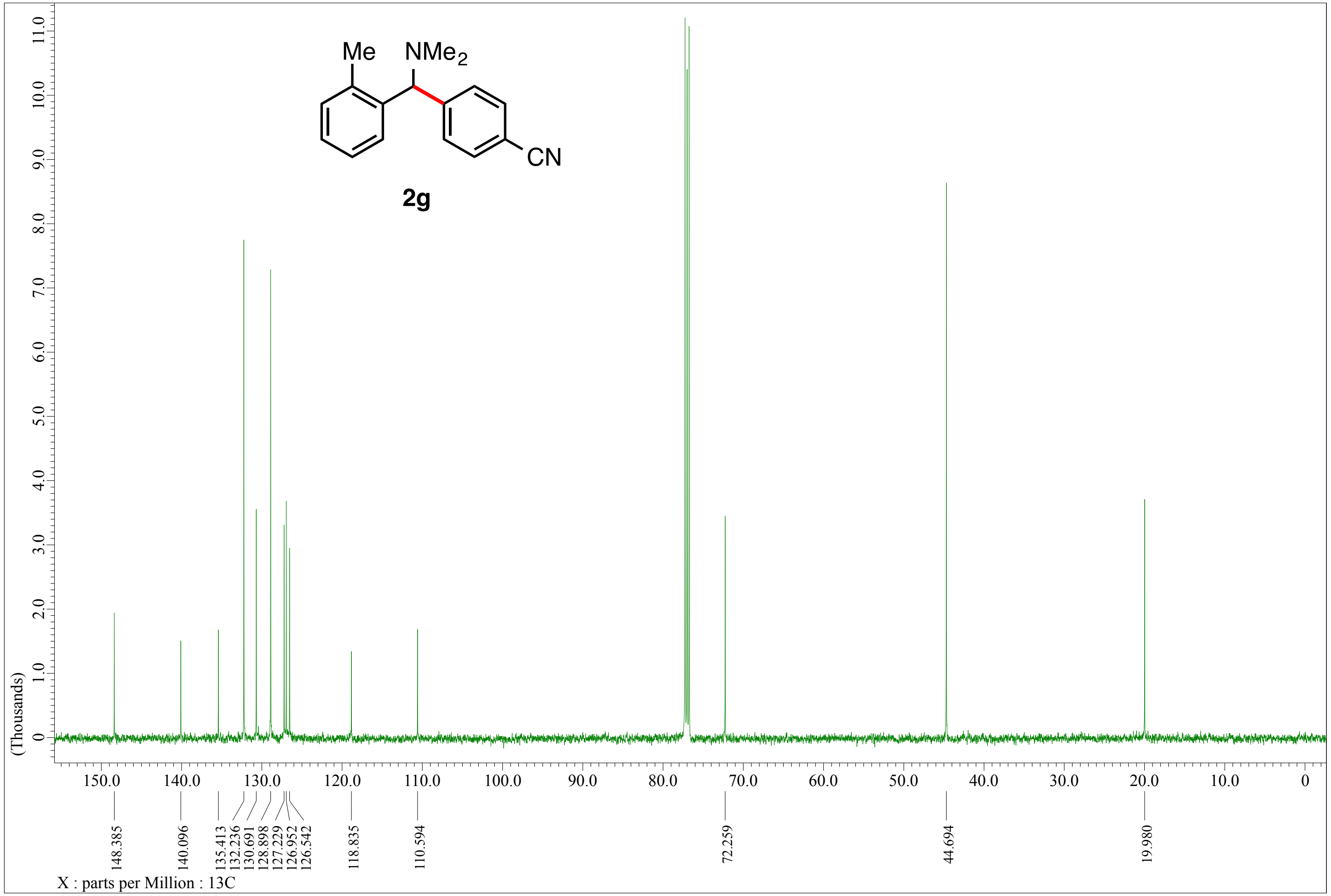
Me
NMe2
CN
2g
(Thousands)
148.385
140.096
135.413
132.236
130.691
128.898
127.229
126.952
126.542
118.835
110.594
72.259
44.694
19.980
X : parts per Million : 13C

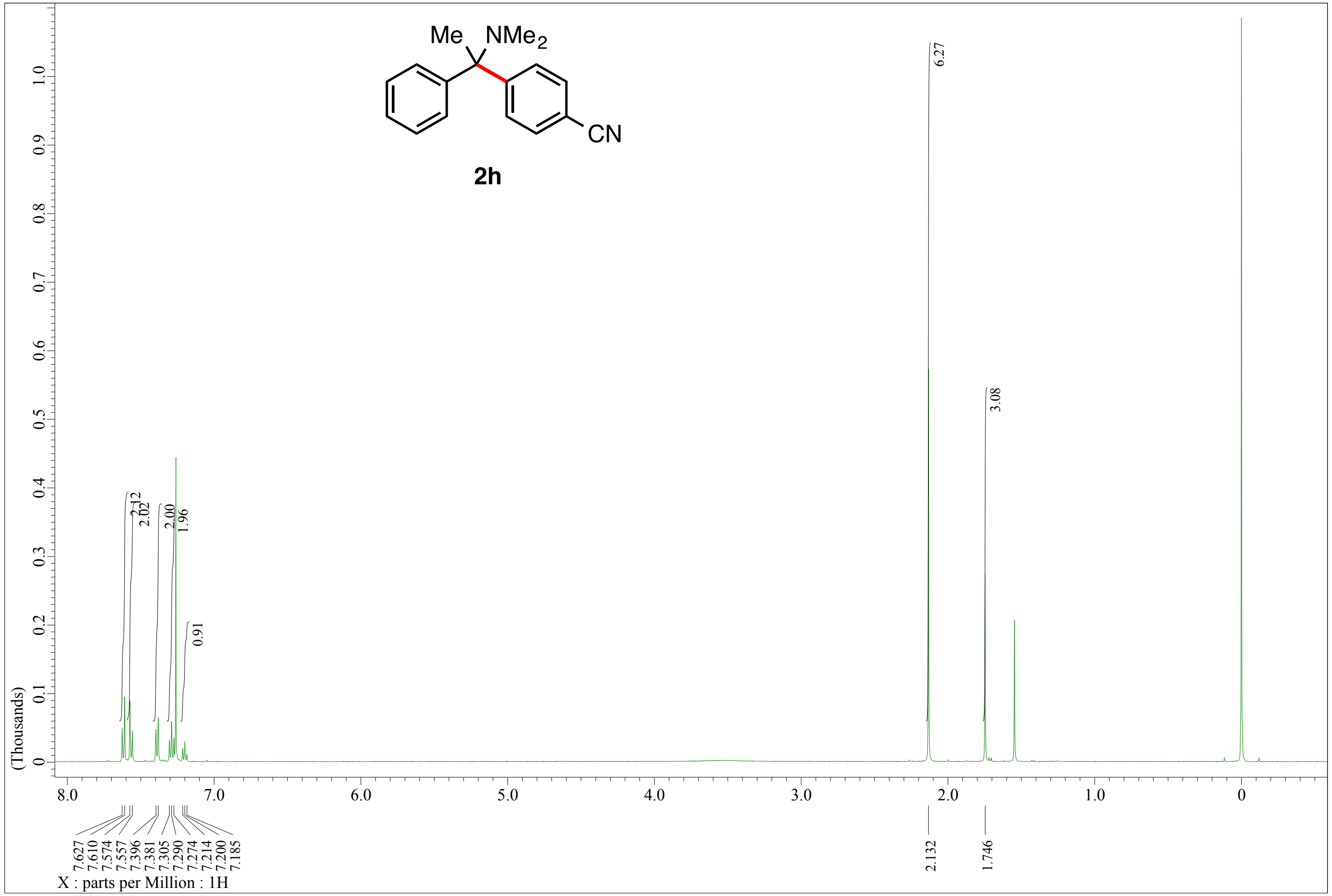

Me
NMe2
CN
2h
6.27
3.08
2.12
2.02
2.00
1.96
0.91
(Thousands)
X : parts per Million : 1H
7.627
7.610
7.574
7.557
7.396
7.381
7.305
7.290
7.274
7.214
7.200
7.185
2.132
1.746

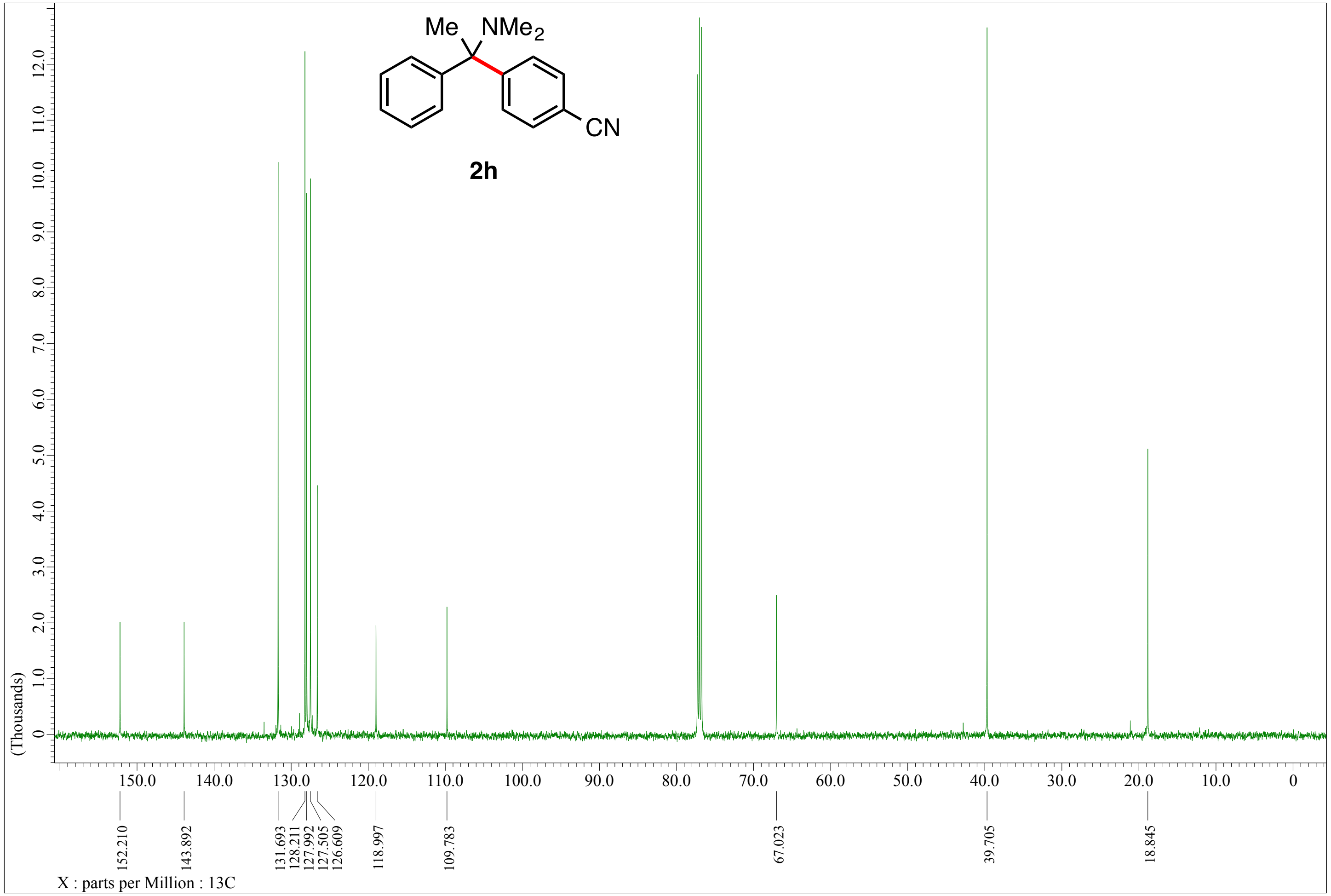
Me
NMe2
CN
2h
(Thousands)
X : parts per Million : 13C
152.210
143.892
131.693
128.211
127.992
127.505
126.609
118.997
109.783
67.023
39.705
18.845

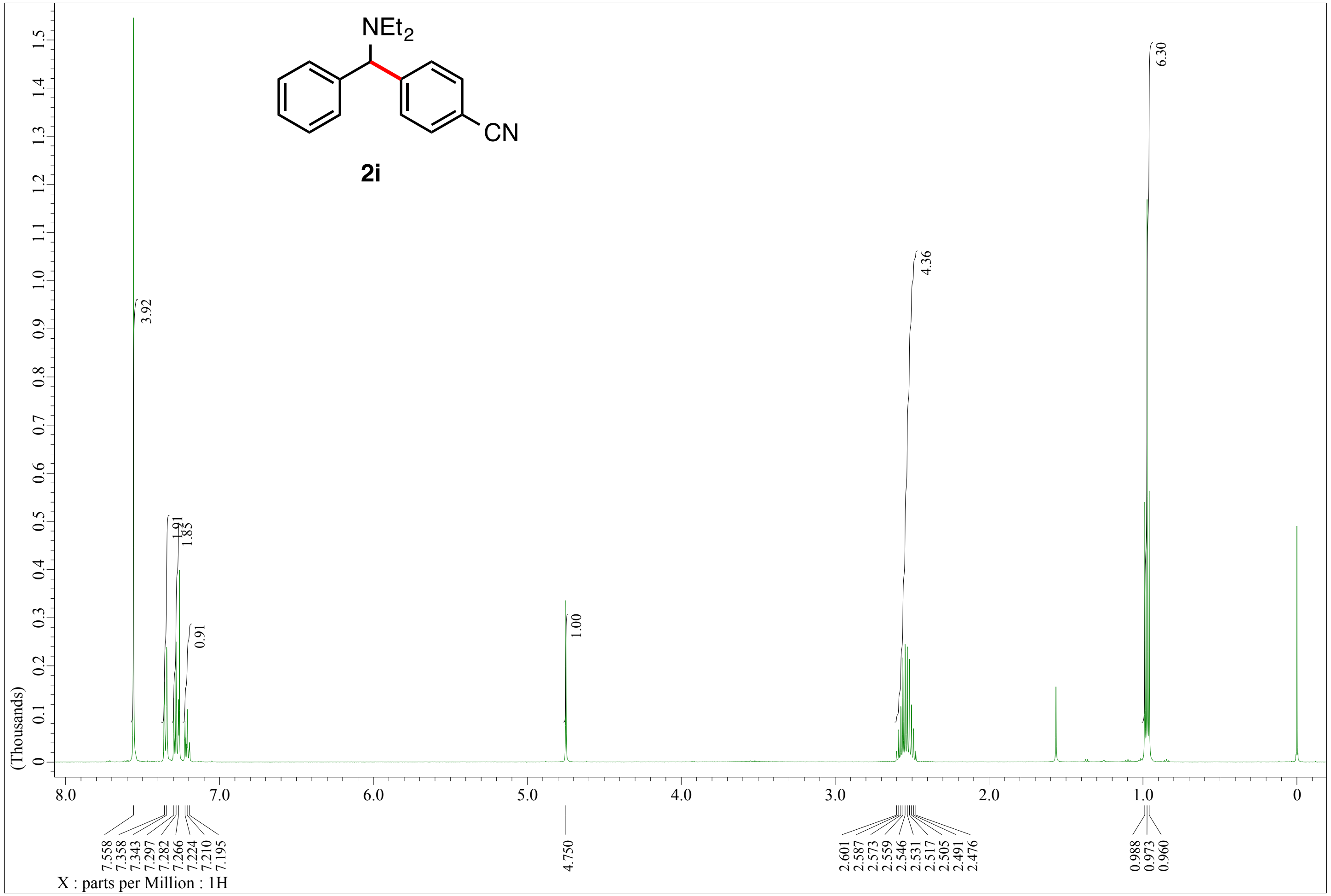
NEt2
CN
2i
(Thousands)
X : parts per Million : 1H
3.92
1.91
1.85
0.91
1.00
4.36
6.30
7.558
7.358
7.343
7.297
7.282
7.266
7.224
7.210
7.195
4.750
2.601
2.587
2.573
2.559
2.546
2.531
2.517
2.505
2.491
2.476
0.988
0.973
0.960

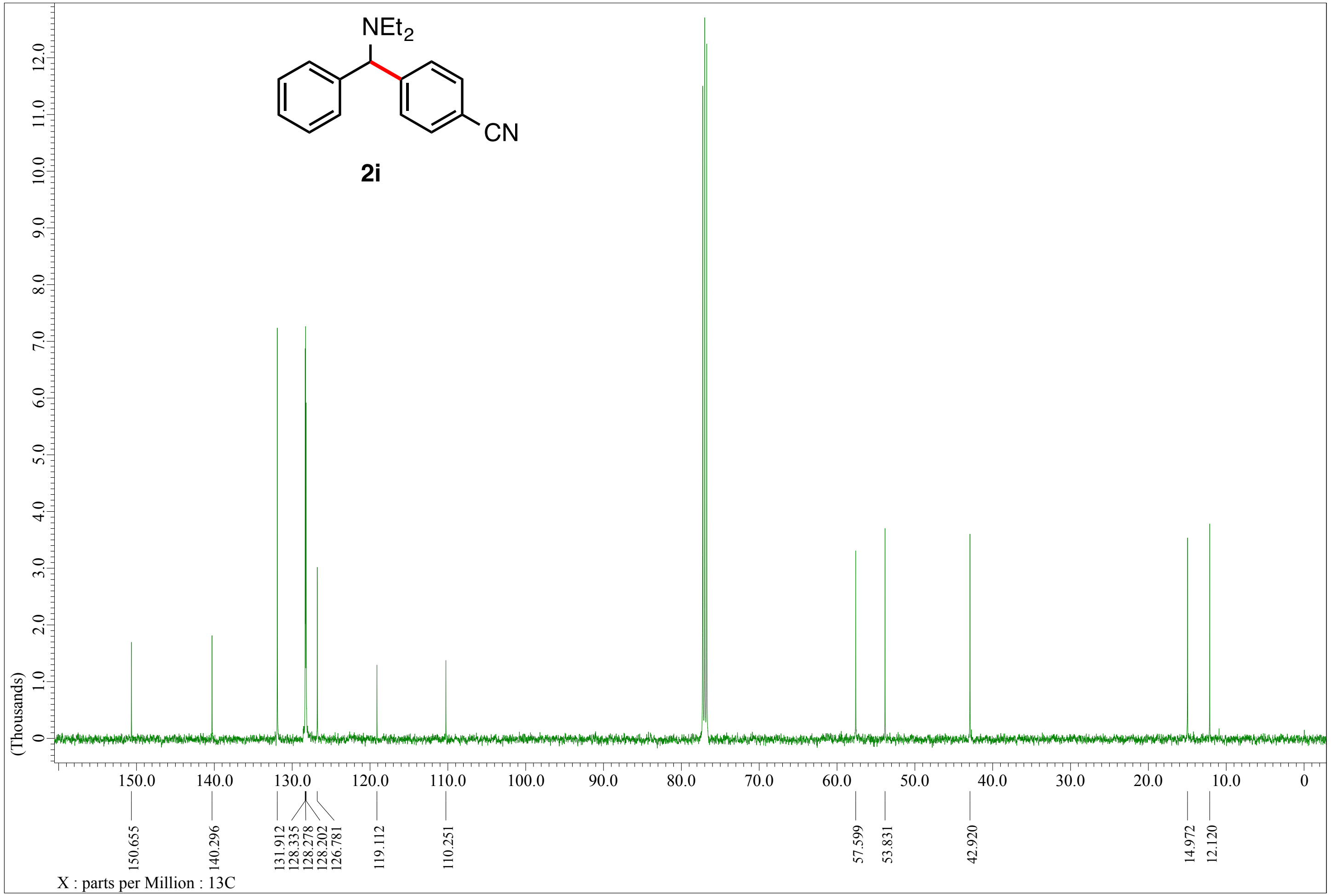
NEt2
CN
2i
(Thousands)
0
1.0
2.0
3.0
4.0
5.0
6.0
7.0
8.0
9.0
10.0
11.0
12.0
150.0
140.0
130.0
120.0
110.0
100.0
90.0
80.0
70.0
60.0
50.0
40.0
30.0
20.0
10.0
0
150.655
140.296
131.912
128.335
128.278
128.202
126.781
119.112
110.251
57.599
53.831
42.920
14.972
12.120
X : parts per Million : 13C

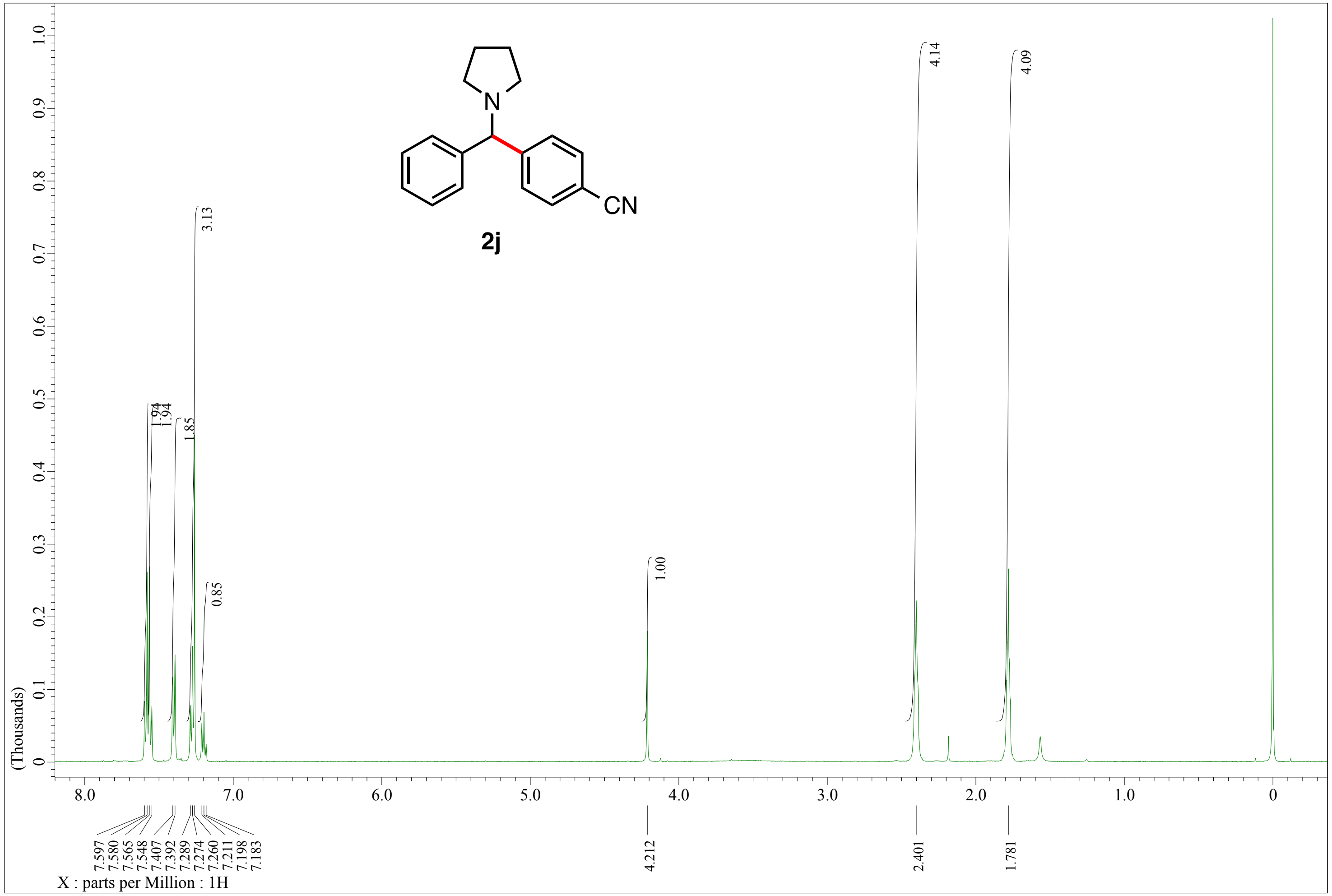
2j
CN
N
X : parts per Million : 1H
(Thousands)
7.597
7.580
7.565
7.548
7.407
7.392
7.289
7.274
7.260
7.211
7.198
7.183
4.212
2.401
1.781
1.94
1.94
1.85
3.13
0.85
1.00
4.14
4.09

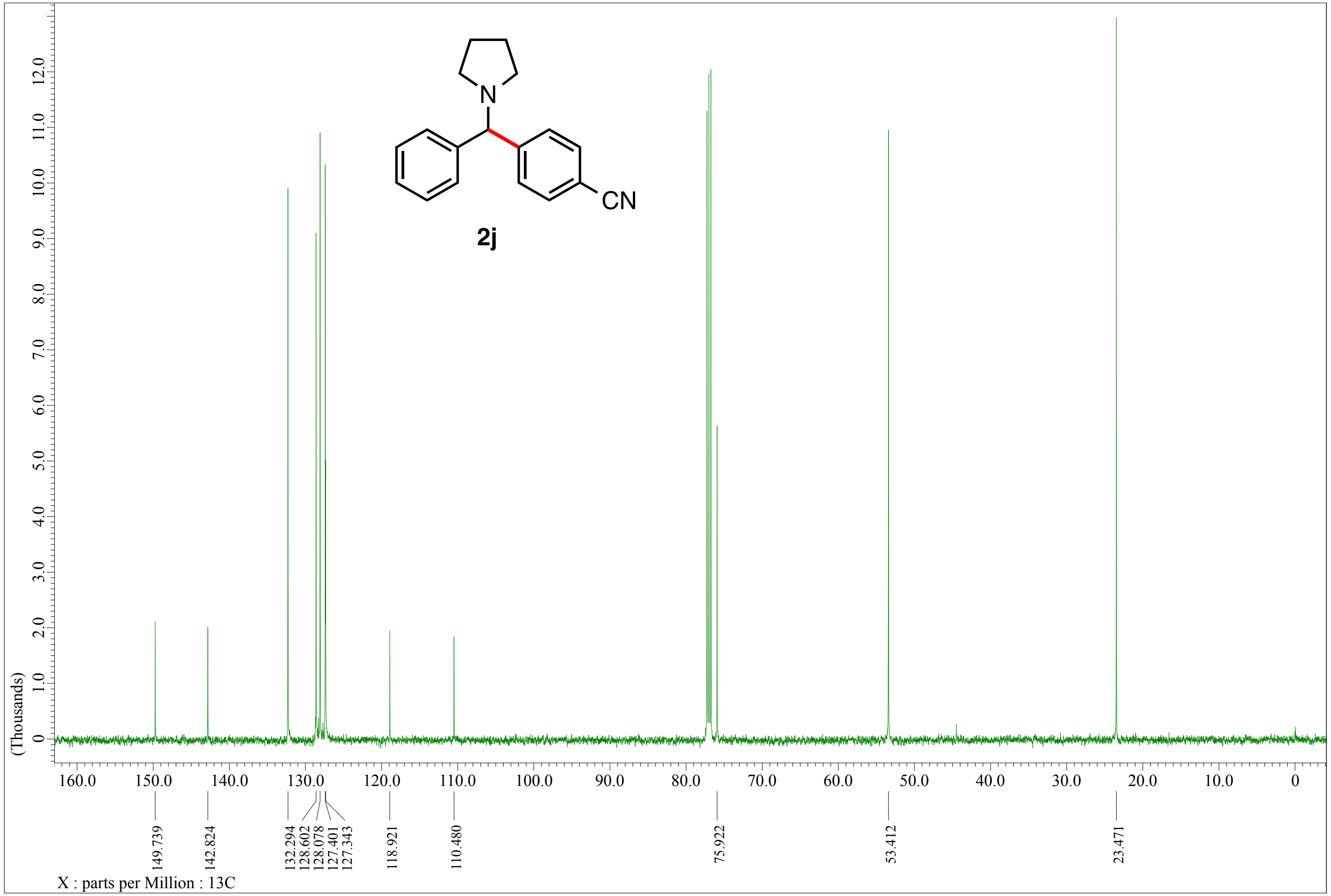
N
CN
2j
(Thousands)
0
1.0
2.0
3.0
4.0
5.0
6.0
7.0
8.0
9.0
10.0
11.0
12.0
160.0
150.0
140.0
130.0
120.0
110.0
100.0
90.0
80.0
70.0
60.0
50.0
40.0
30.0
20.0
10.0
0
149.739
142.824
132.294
128.602
128.078
127.401
127.343
118.921
110.480
75.922
53.412
23.471
X : parts per Million : 13C

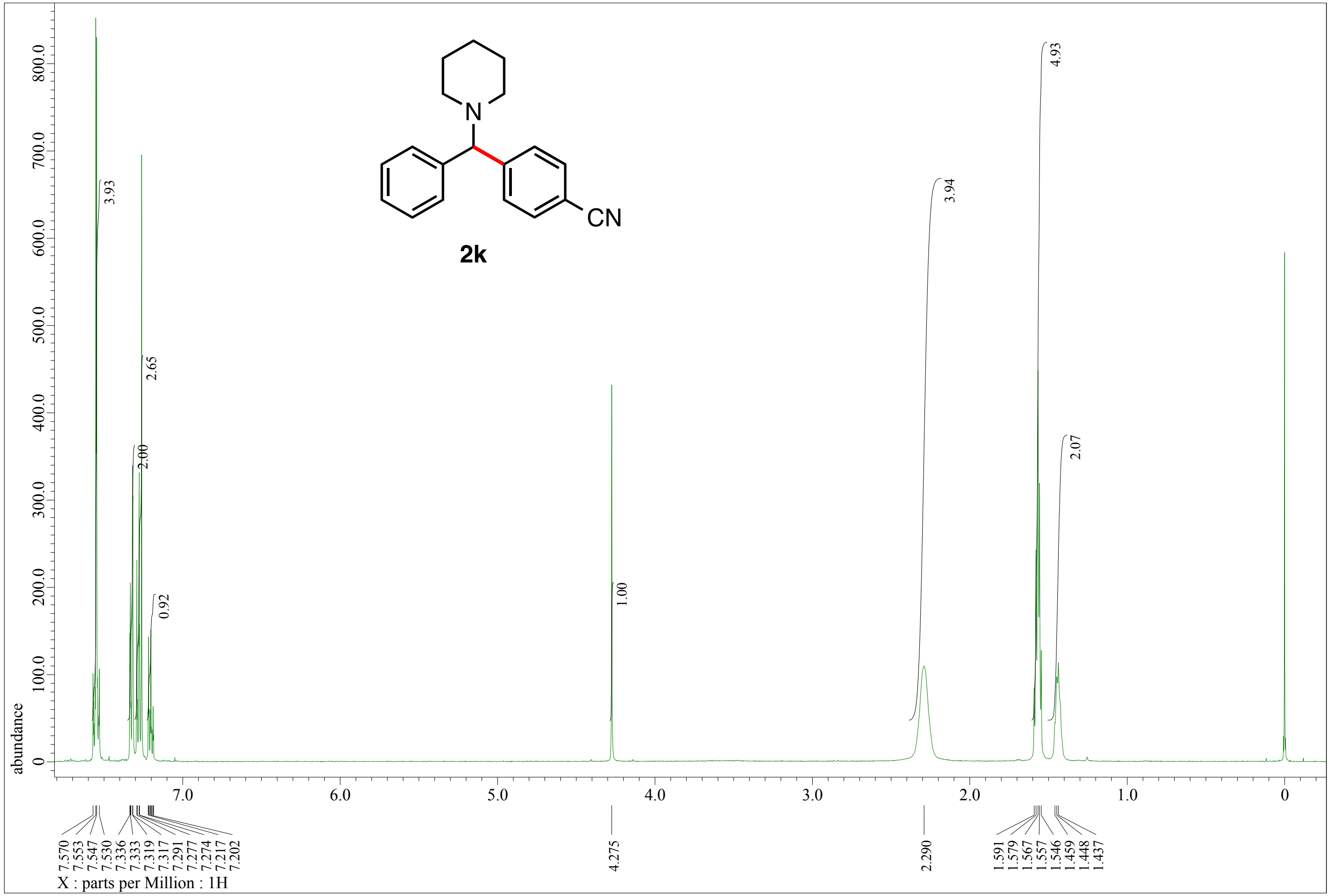
N
CN
2k
800.0
700.0
600.0
500.0
400.0
300.0
200.0
100.0
0
abundance
3.93
2.65
2.00
0.92
1.00
3.94
4.93
2.07
7.0
6.0
5.0
4.0
3.0
2.0
1.0
0
7.570
7.553
7.547
7.530
7.336
7.333
7.319
7.317
7.291
7.277
7.274
7.217
7.202
4.275
2.290
1.591
1.579
1.567
1.557
1.546
1.459
1.448
1.437
X : parts per Million : 1H

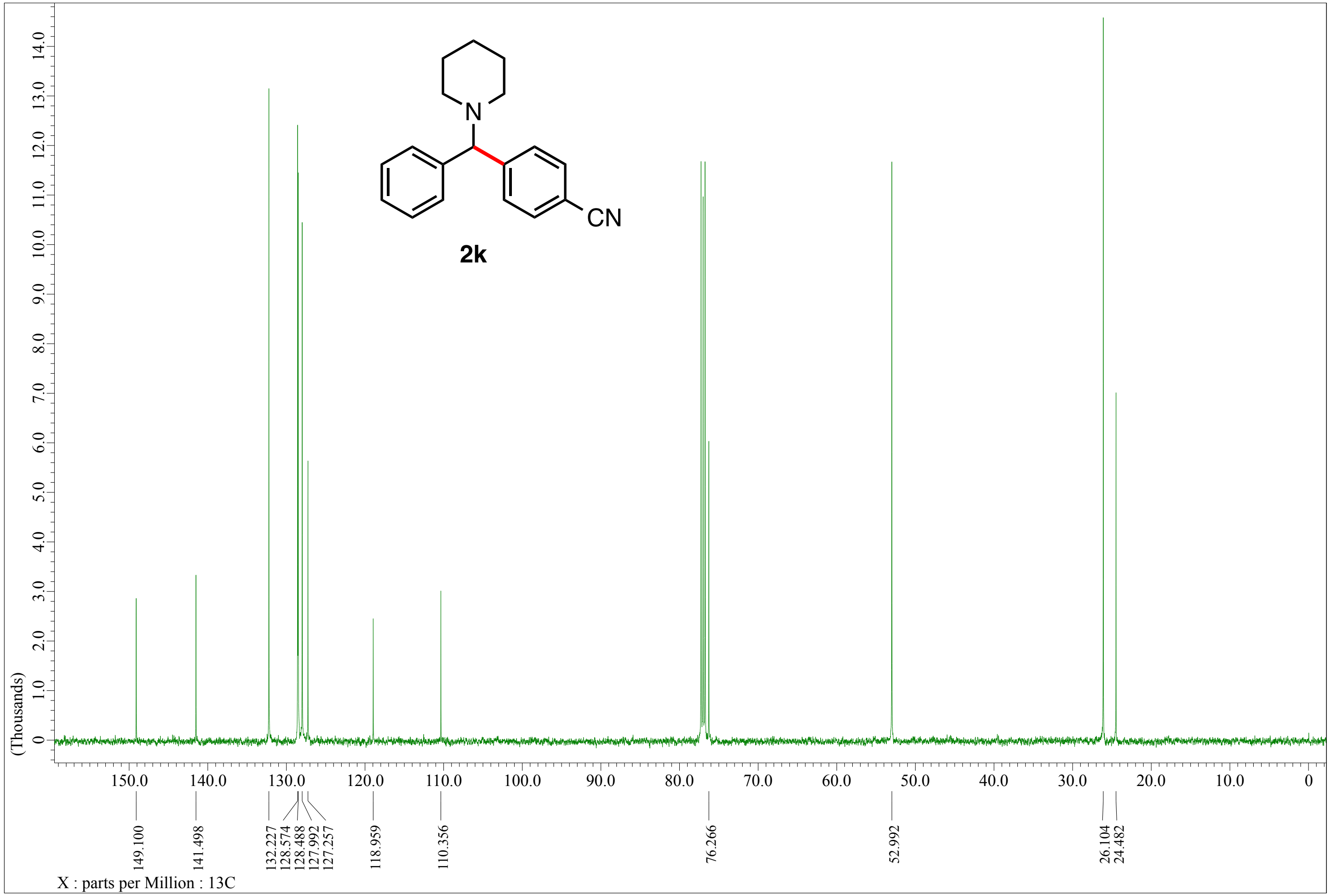

2k
CN
N
(Thousands)
X : parts per Million : 13C
149.100
141.498
132.227
128.574
128.488
127.992
127.257
118.959
110.356
76.266
52.992
26.104
24.482

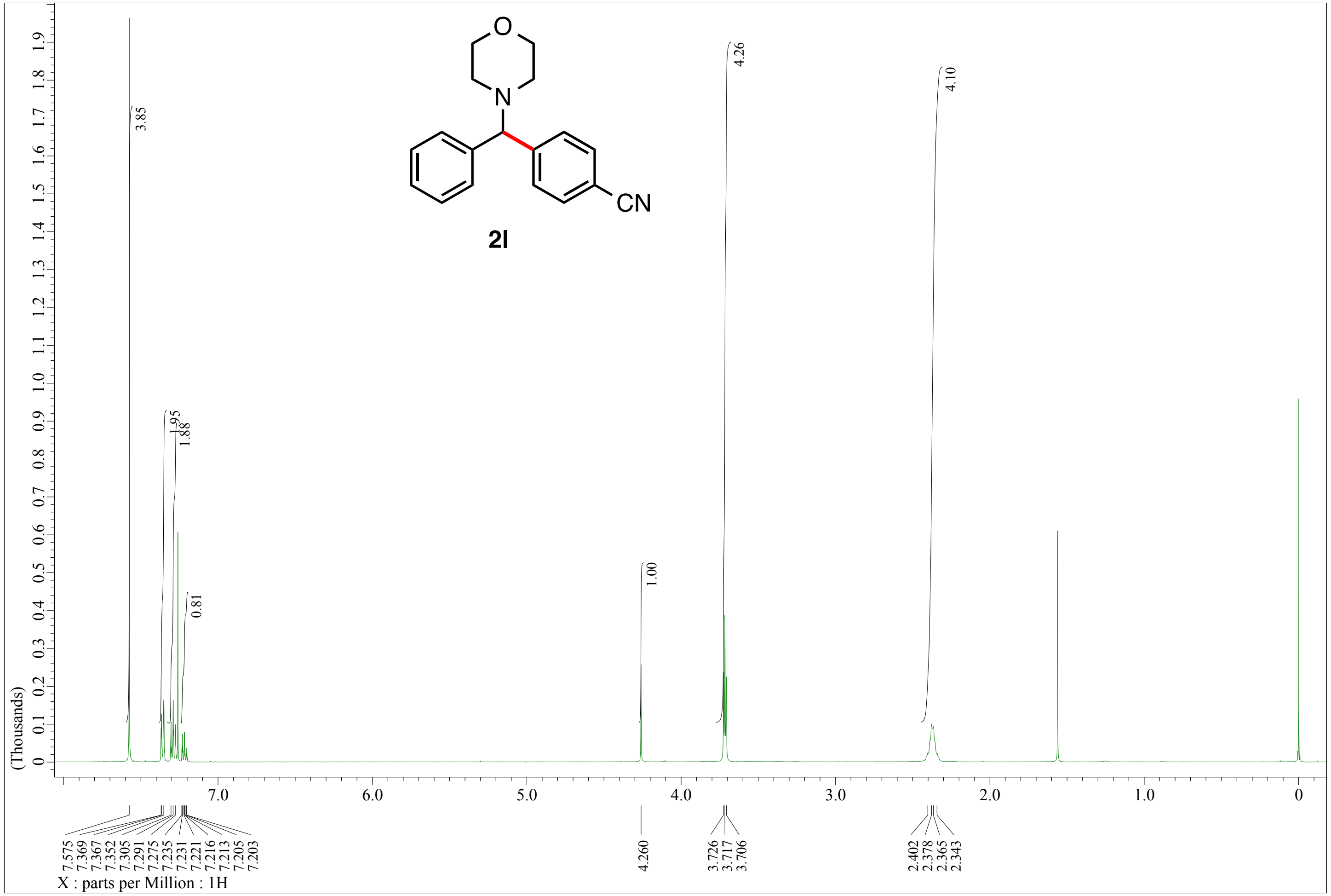
O
N
CN
2l
3.85
1.95
1.88
0.81
1.00
4.26
4.10
(Thousands)
0 0.1 0.2 0.3 0.4 0.5 0.6 0.7 0.8 0.9 1.0 1.1 1.2 1.3 1.4 1.5 1.6 1.7 1.8 1.9
7.0 6.0 5.0 4.0 3.0 2.0 1.0 0
7.575 7.369 7.367 7.352 7.305 7.291 7.275 7.235 7.231 7.221 7.216 7.213 7.205 7.203
4.260
3.726 3.717 3.706
2.402 2.378 2.365 2.343
X : parts per Million : 1H

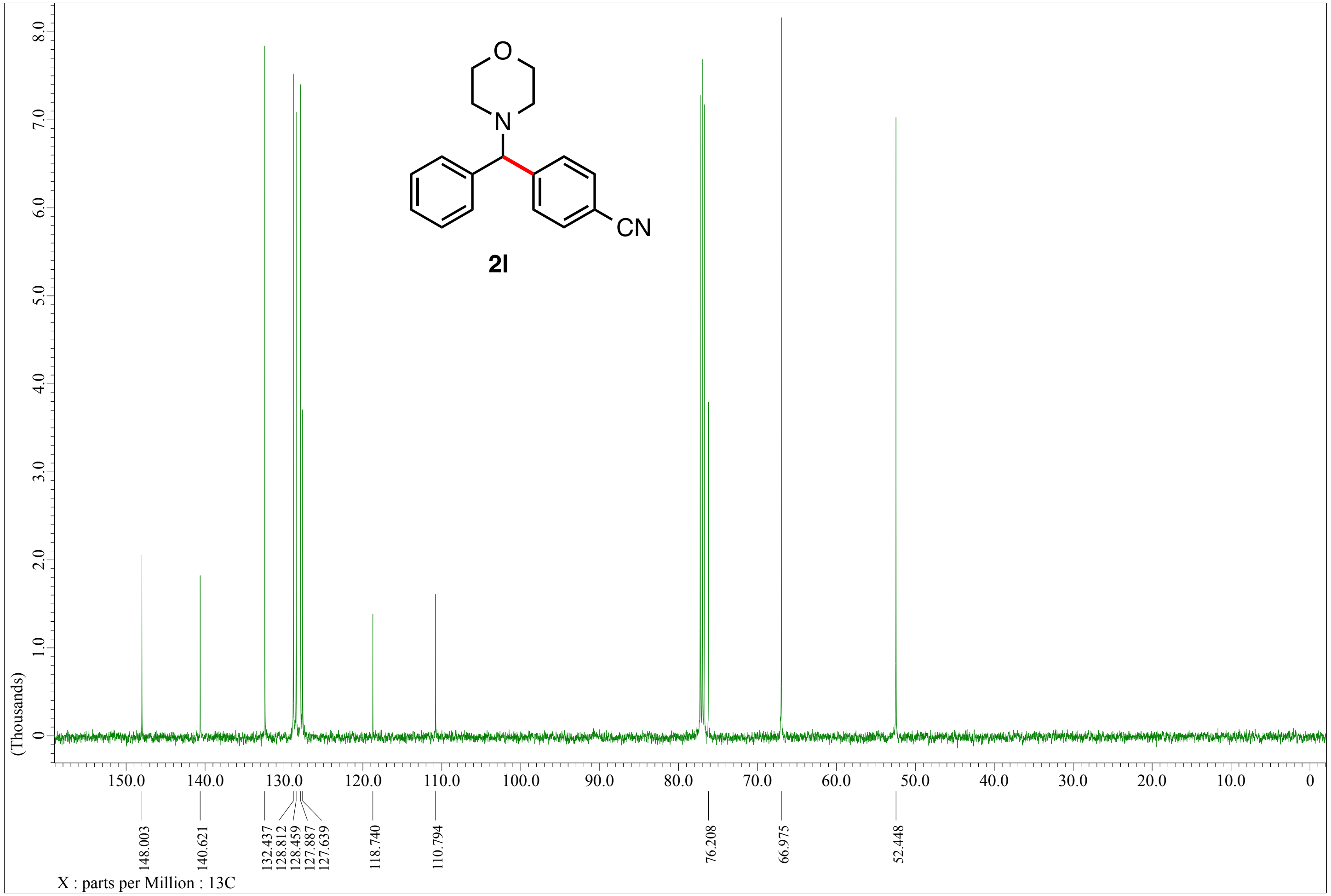

2l
CN
O
N
(Thousands)
8.0
7.0
6.0
5.0
4.0
3.0
2.0
1.0
0
150.0
140.0
130.0
120.0
110.0
100.0
90.0
80.0
70.0
60.0
50.0
40.0
30.0
20.0
10.0
0
148.003
140.621
132.437
128.812
128.459
127.887
127.639
118.740
110.794
76.208
66.975
52.448
X : parts per Million : 13C

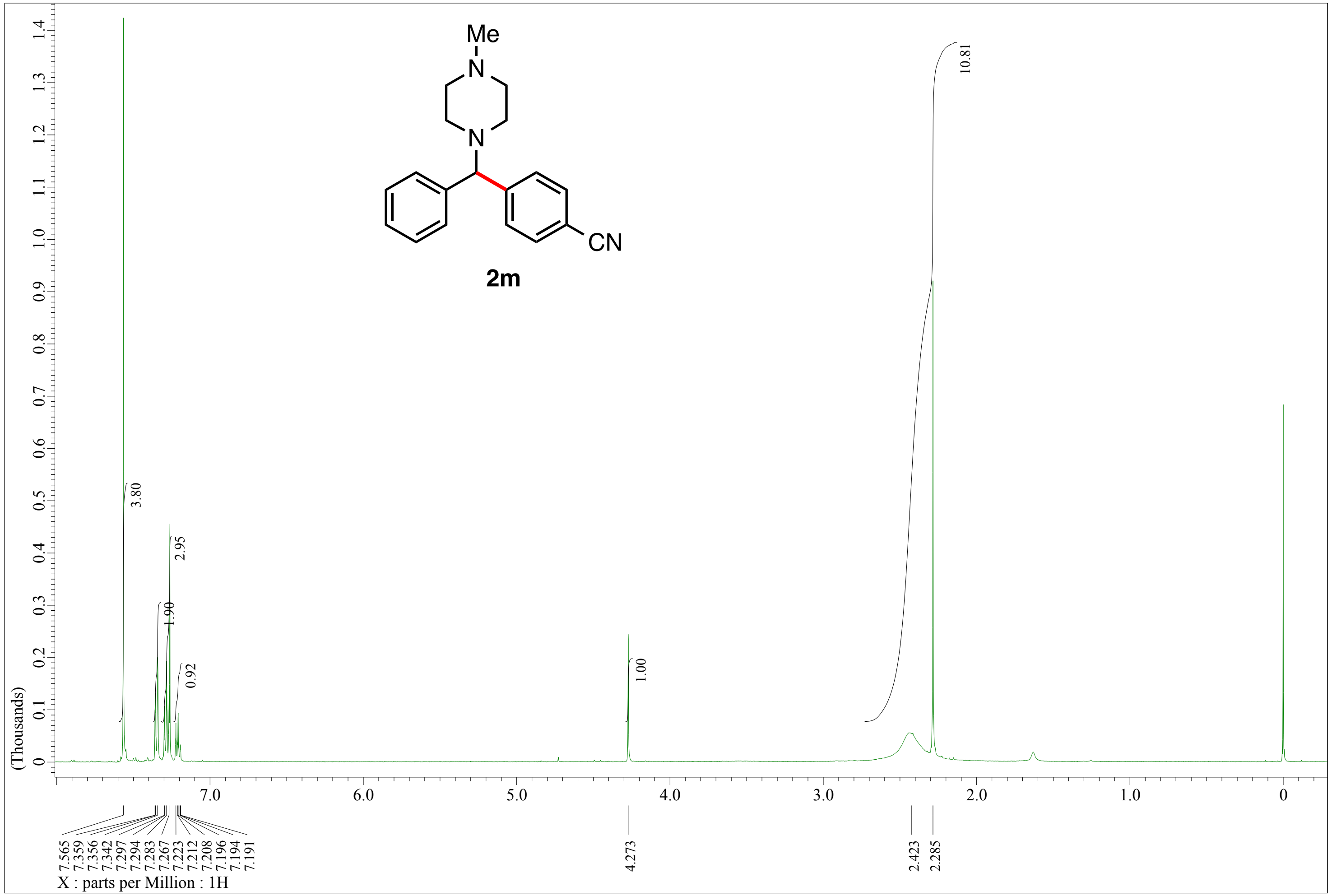
Me
N
N
CN
2m
(Thousands)
3.80
2.95
1.90
0.92
1.00
10.81
7.565
7.359
7.356
7.342
7.297
7.294
7.283
7.267
7.223
7.212
7.208
7.196
7.194
7.191
4.273
2.423
2.285
X : parts per Million : 1H

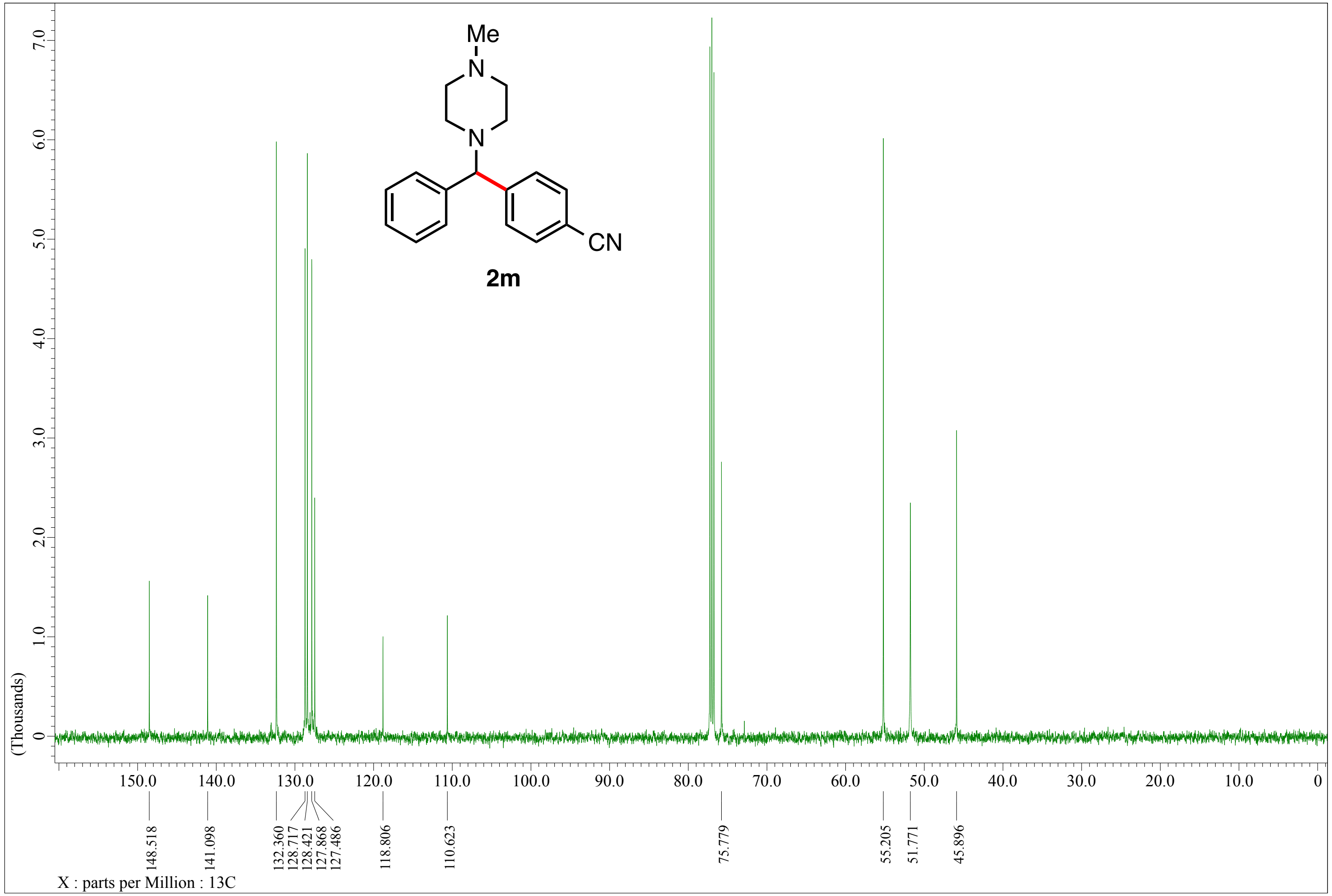
Me
N
N
CN
2m
7.0
6.0
5.0
4.0
3.0
2.0
1.0
0
(Thousands)
150.0
140.0
130.0
120.0
110.0
100.0
90.0
80.0
70.0
60.0
50.0
40.0
30.0
20.0
10.0
0
148.518
141.098
132.360
128.717
128.421
127.868
127.486
118.806
110.623
75.779
55.205
51.771
45.896
X : parts per Million : 13C

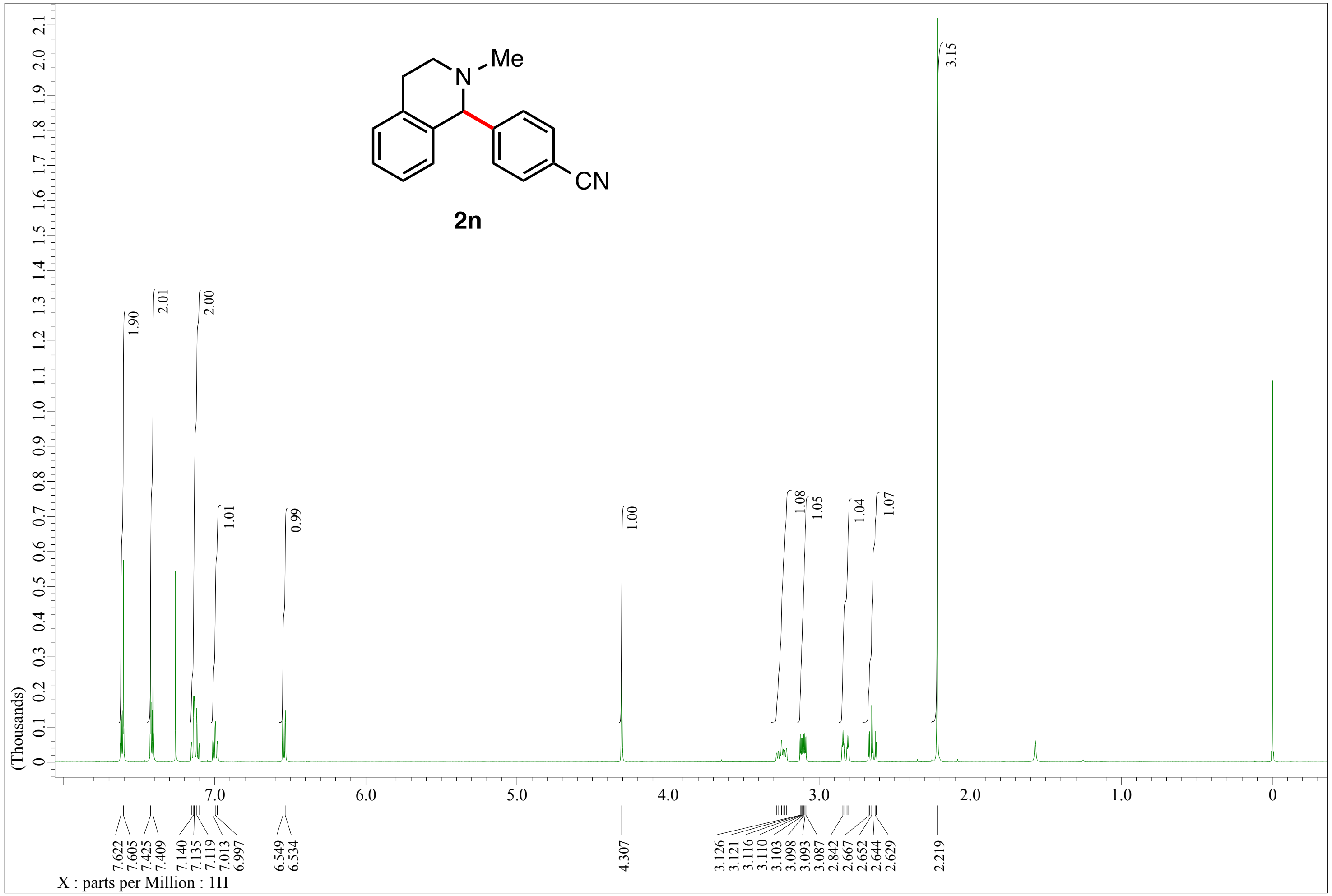

N
Me
CN
2n
X : parts per Million : 1H
(Thousands)

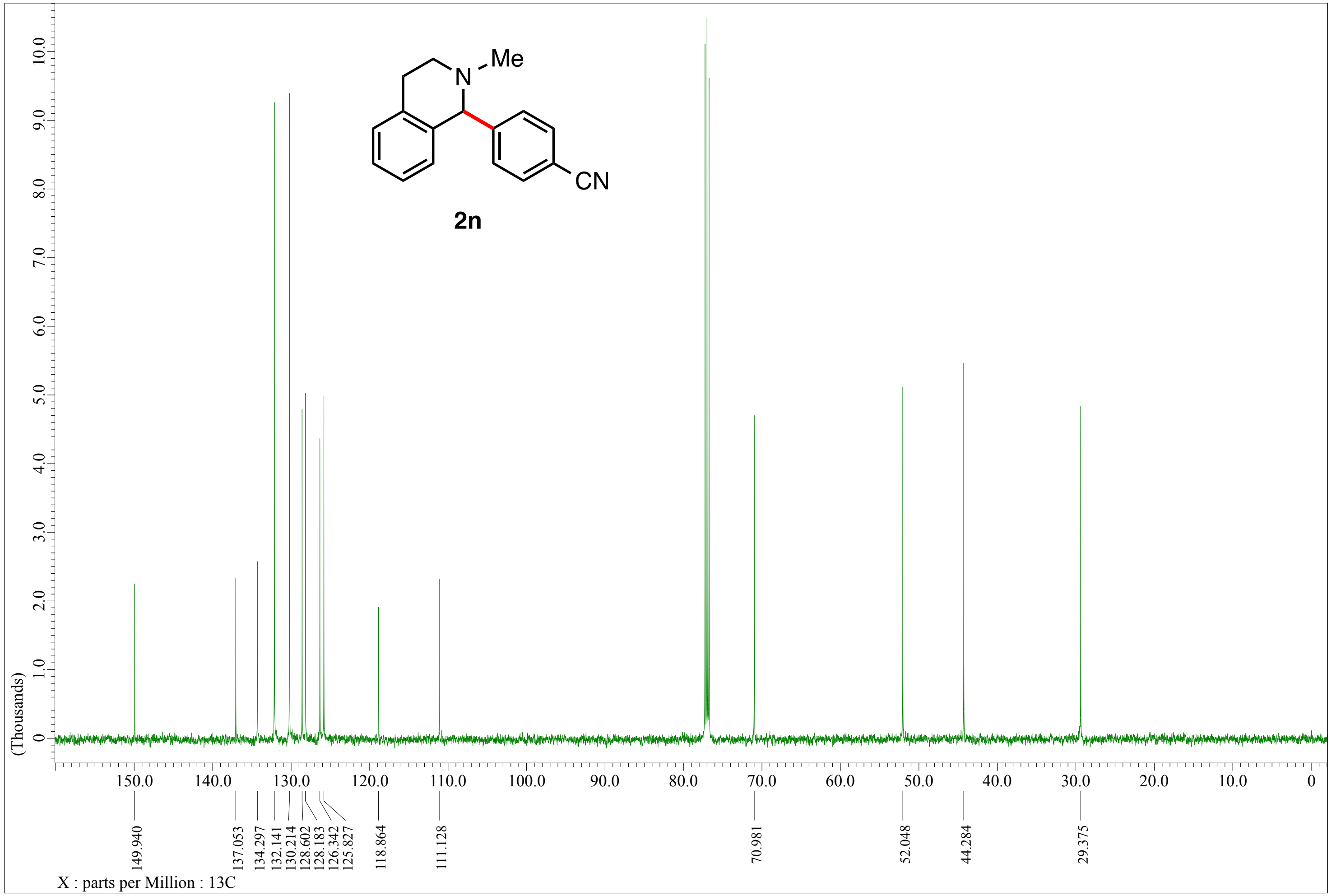
Me
N
CN
2n
(Thousands)
0
1.0
2.0
3.0
4.0
5.0
6.0
7.0
8.0
9.0
10.0
150.0
140.0
130.0
120.0
110.0
100.0
90.0
80.0
70.0
60.0
50.0
40.0
30.0
20.0
10.0
0
149.940
137.053
134.297
132.141
130.214
128.602
128.183
126.342
125.827
118.864
111.128
70.981
52.048
44.284
29.375
X : parts per Million : 13C

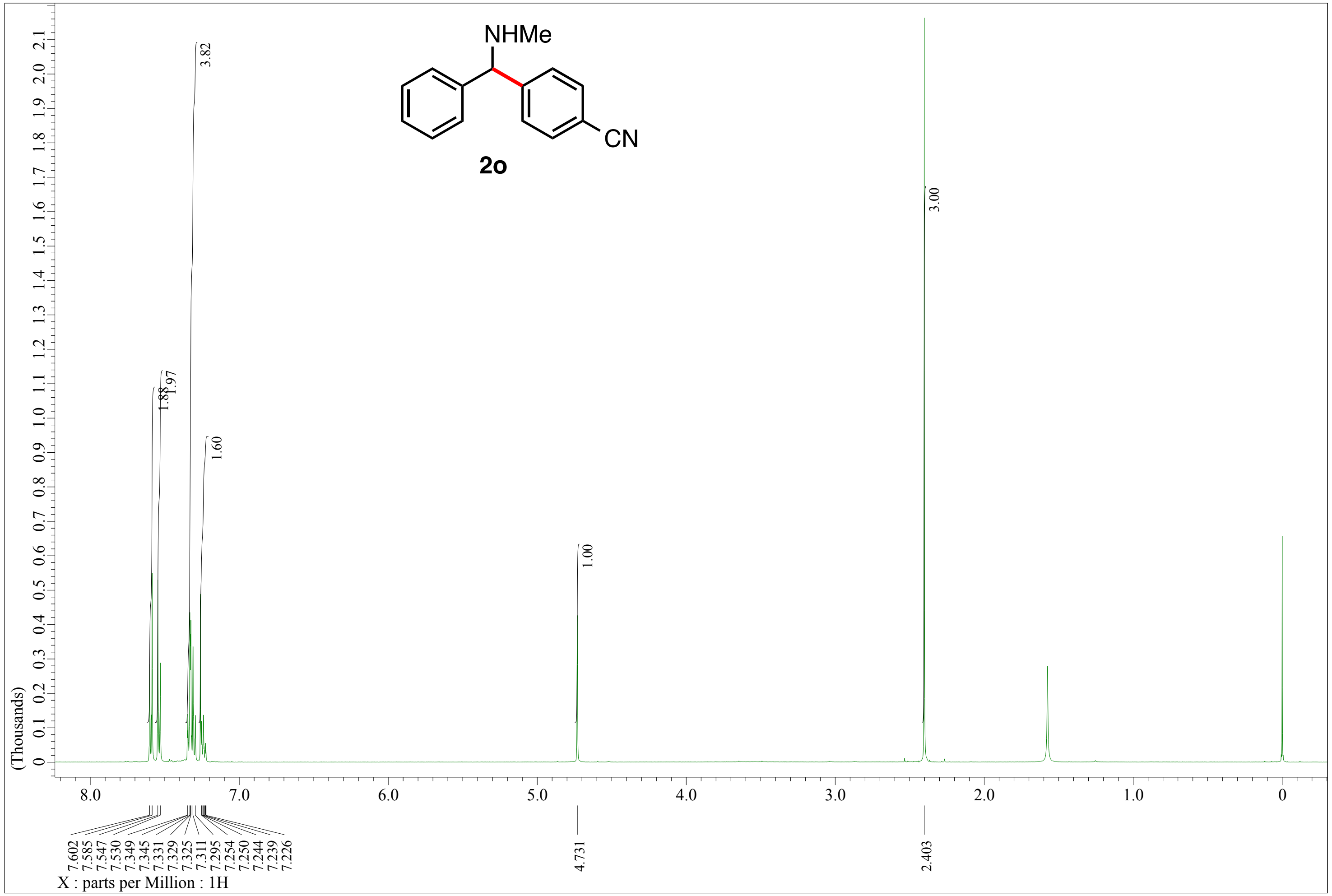
NHMe
CN
2o
(Thousands)
3.82
1.88
1.97
1.60
1.00
3.00
7.602
7.585
7.547
7.530
7.349
7.345
7.331
7.329
7.325
7.311
7.295
7.254
7.250
7.244
7.239
7.226
4.731
2.403
X : parts per Million : 1H

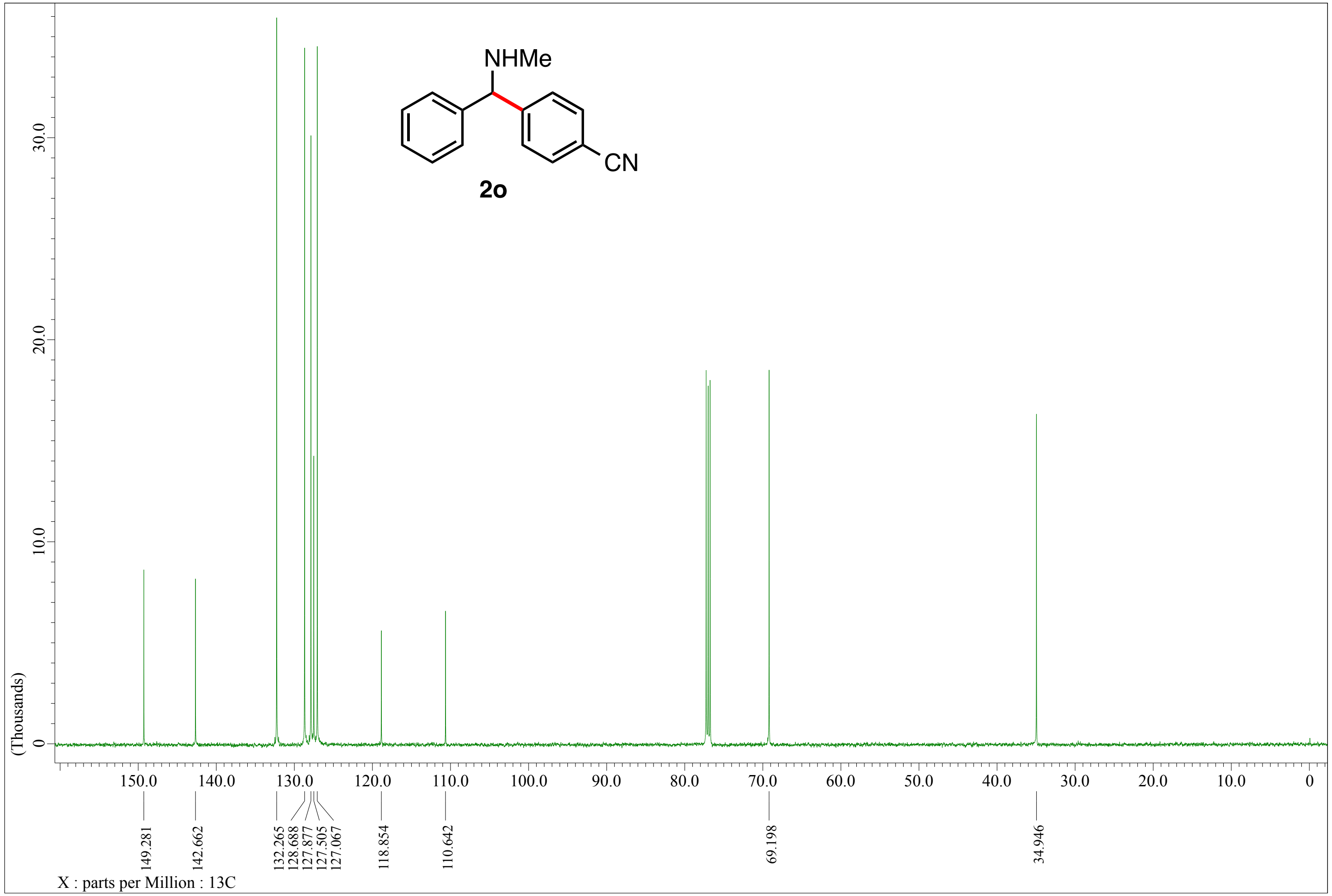
NHMe
CN
2o
(Thousands)
30.0
20.0
10.0
0
150.0
140.0
130.0
120.0
110.0
100.0
90.0
80.0
70.0
60.0
50.0
40.0
30.0
20.0
10.0
0
149.281
142.662
132.265
128.688
127.877
127.505
127.067
118.854
110.642
69.198
34.946
X : parts per Million : 13C

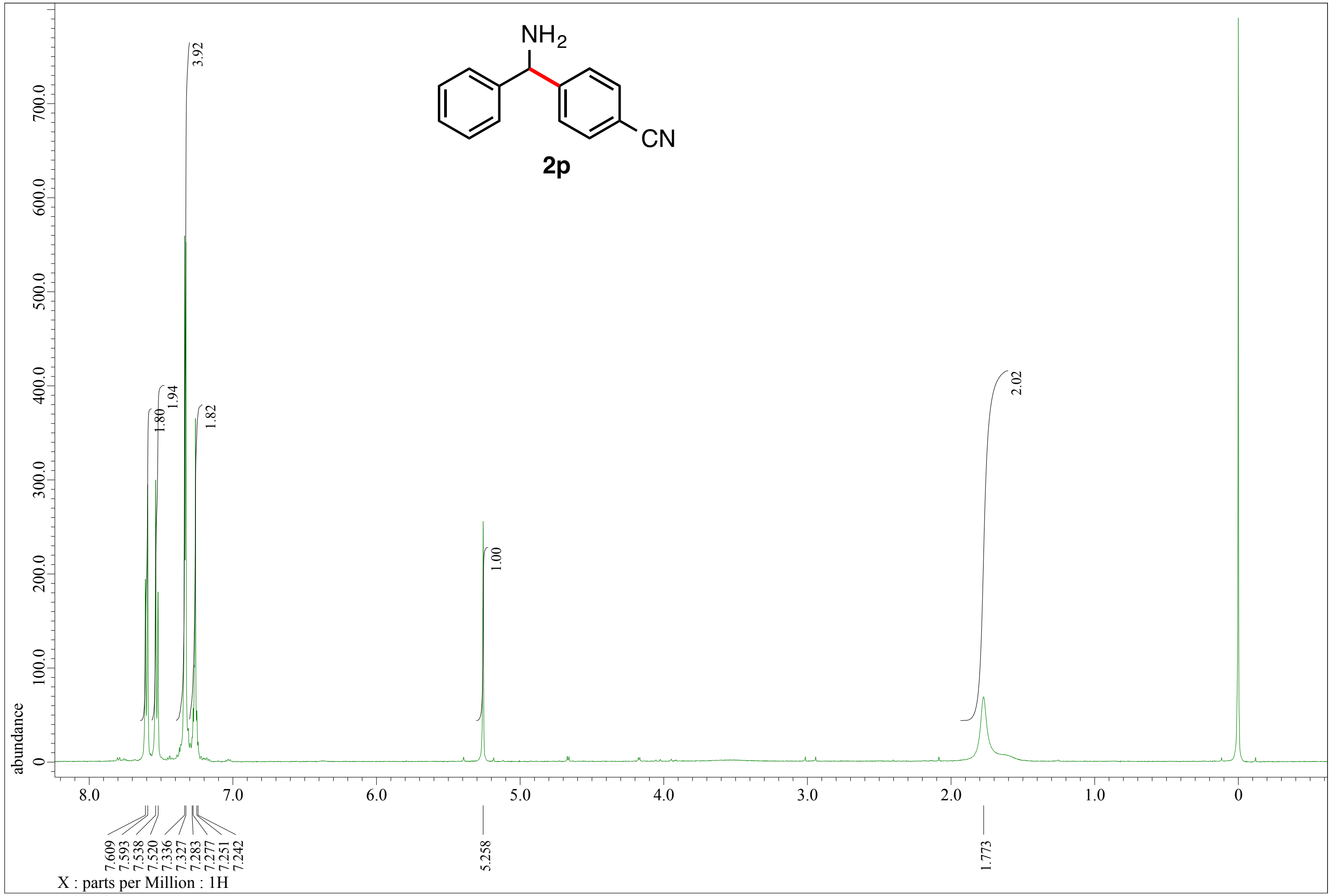
NH2
CN
2p
3.92
1.80
1.94
1.82
1.00
2.02
700.0
600.0
500.0
400.0
300.0
200.0
100.0
0
abundance
8.0
7.0
6.0
5.0
4.0
3.0
2.0
1.0
0
7.609
7.593
7.538
7.520
7.336
7.327
7.283
7.277
7.251
7.242
5.258
1.773
X : parts per Million : 1H

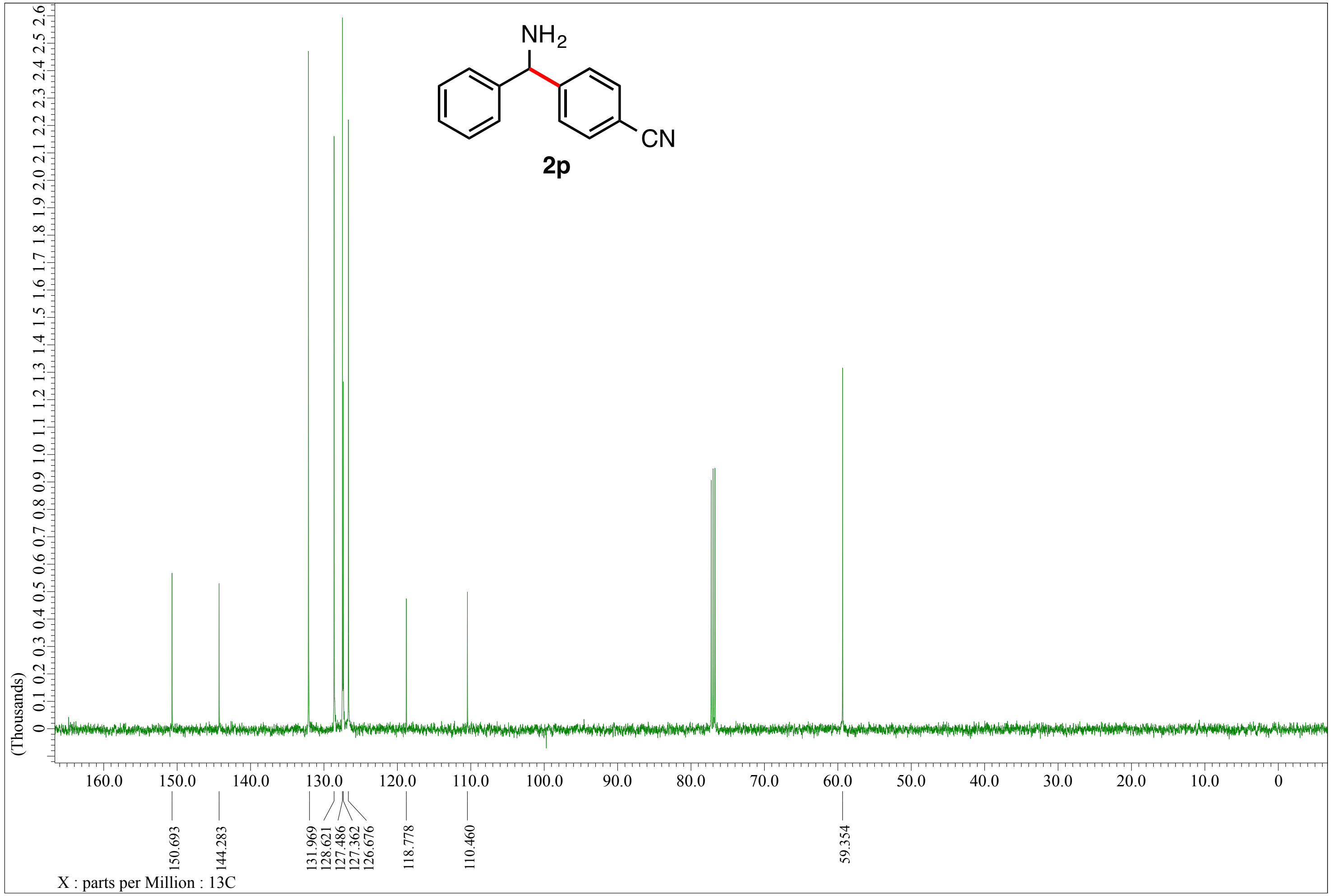
NH2
CN
2p
(Thousands)
X : parts per Million : 13C
150.693
144.283
131.969
128.621
127.486
127.362
126.676
118.778
110.460
59.354

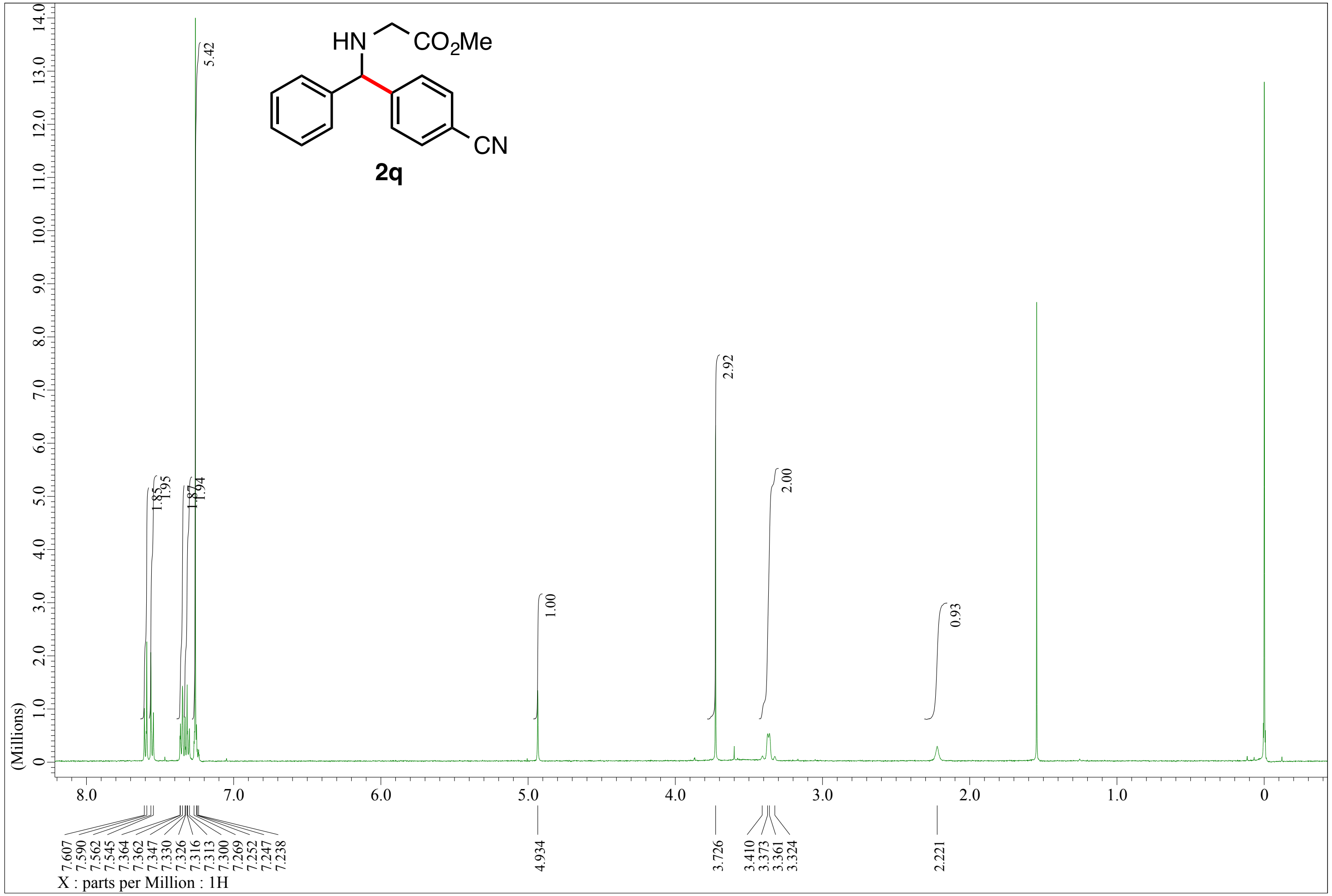
HN
CO2Me
CN
2q
X : parts per Million : 1H
(Millions)
7.607
7.590
7.562
7.545
7.364
7.362
7.347
7.330
7.326
7.316
7.313
7.300
7.269
7.252
7.247
7.238
4.934
3.726
3.410
3.373
3.361
3.324
2.221
1.85
1.95
1.87
1.94
5.42
1.00
2.92
2.00
0.93

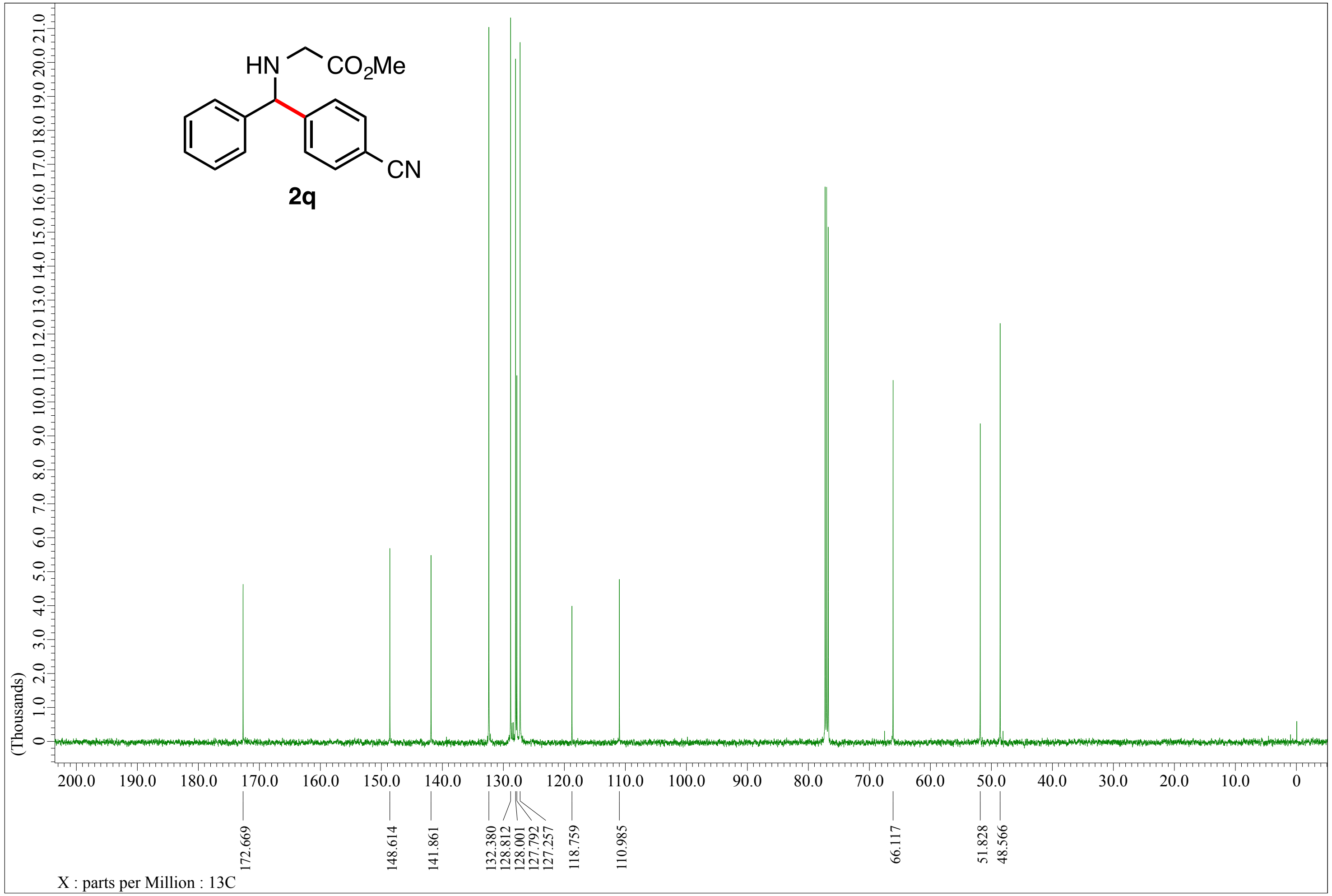
HN
CO2Me
CN
2q
(Thousands)
0
1.0
2.0
3.0
4.0
5.0
6.0
7.0
8.0
9.0
10.0
11.0
12.0
13.0
14.0
15.0
16.0
17.0
18.0
19.0
20.0
21.0
200.0 190.0 180.0 170.0 160.0 150.0 140.0 130.0 120.0 110.0 100.0 90.0 80.0 70.0 60.0 50.0 40.0 30.0 20.0 10.0 0
172.669
148.614
141.861
132.380
128.812
128.001
127.792
127.257
118.759
110.985
66.117
51.828
48.566
X : parts per Million : 13C

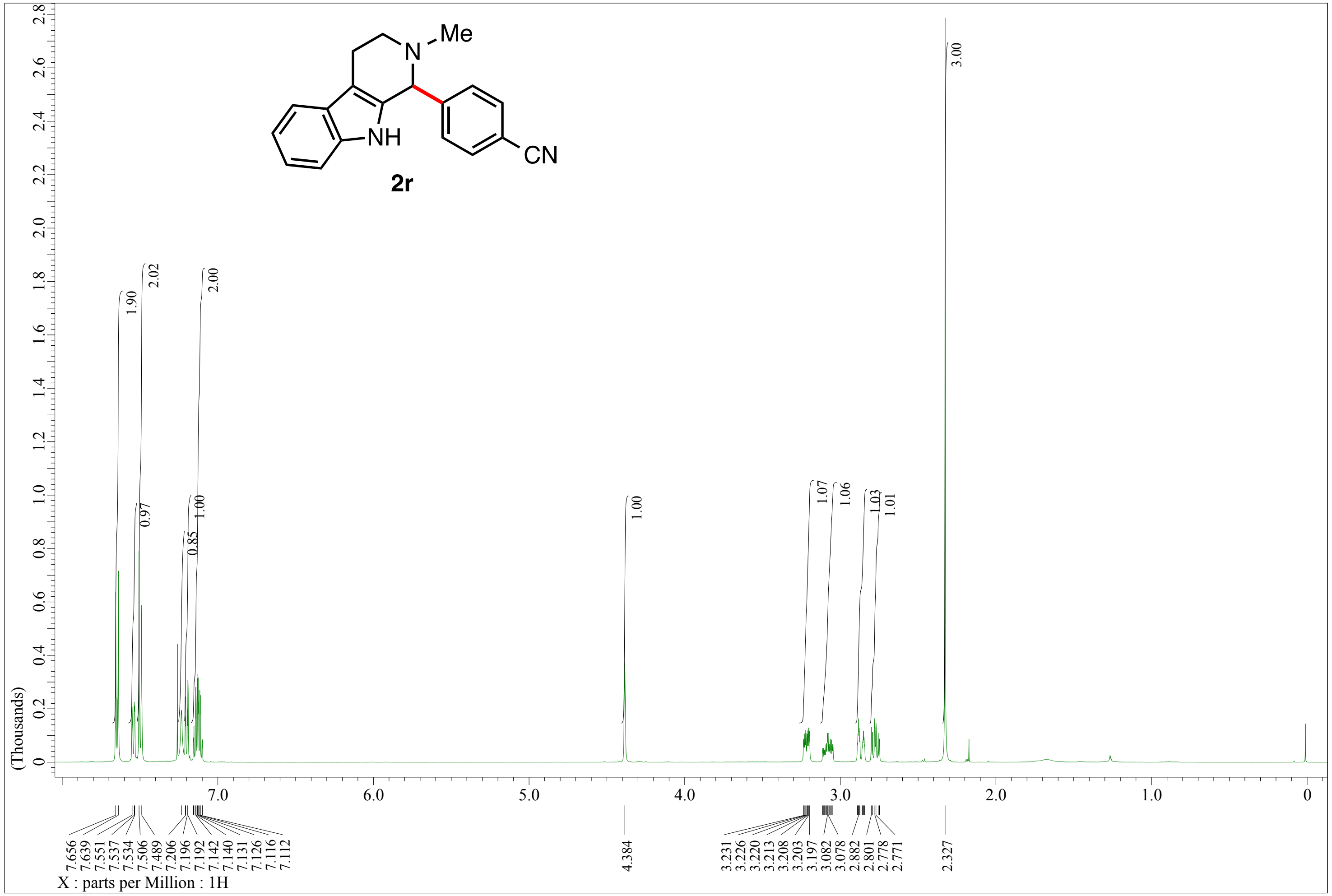

Me
N
NH
CN
2r
(Thousands)
X : parts per Million : 1H
7.656
7.639
7.551
7.537
7.534
7.506
7.489
7.206
7.196
7.192
7.142
7.140
7.131
7.126
7.116
7.112
4.384
3.231
3.226
3.220
3.213
3.208
3.203
3.197
3.082
3.078
2.882
2.801
2.778
2.771
2.327
1.90
0.97
2.02
0.85
1.00
2.00
1.00
1.07
1.06
1.03
1.01
3.00

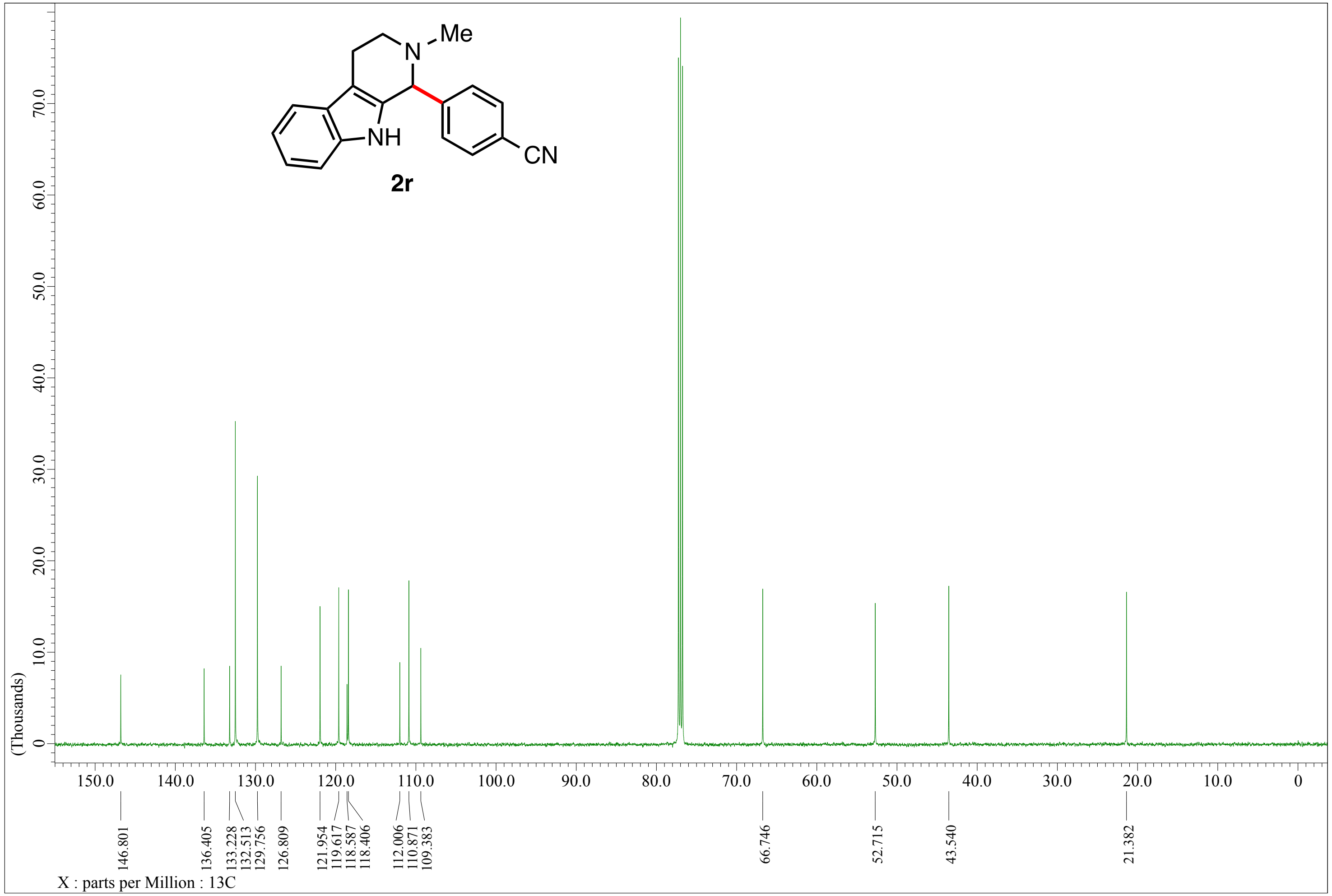

Me
N
NH
CN
2r
(Thousands)
70.0
60.0
50.0
40.0
30.0
20.0
10.0
0
150.0 140.0 130.0 120.0 110.0 100.0 90.0 80.0 70.0 60.0 50.0 40.0 30.0 20.0 10.0 0
146.801
136.405
133.228
132.513
129.756
126.809
121.954
119.617
118.587
118.406
112.006
110.871
109.383
66.746
52.715
43.540
21.382
X : parts per Million : 13C

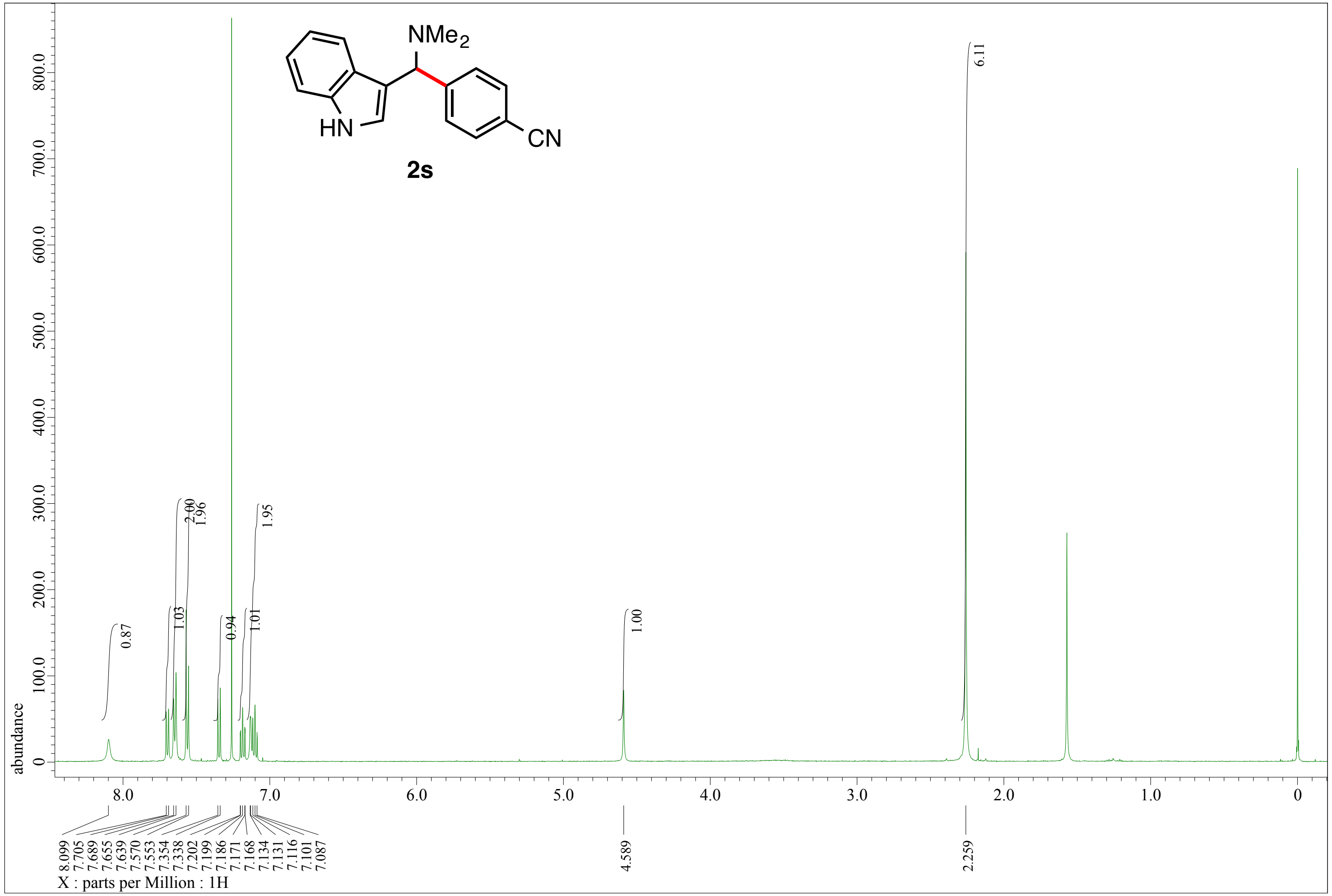
NMe2
HN
CN
2s
abundance
X : parts per Million : 1H
0.87
1.03
2.00
1.96
0.94
1.01
1.95
1.00
6.11
8.099
7.705
7.689
7.655
7.639
7.570
7.553
7.354
7.338
7.202
7.199
7.186
7.171
7.168
7.134
7.131
7.116
7.101
7.087
4.589
2.259

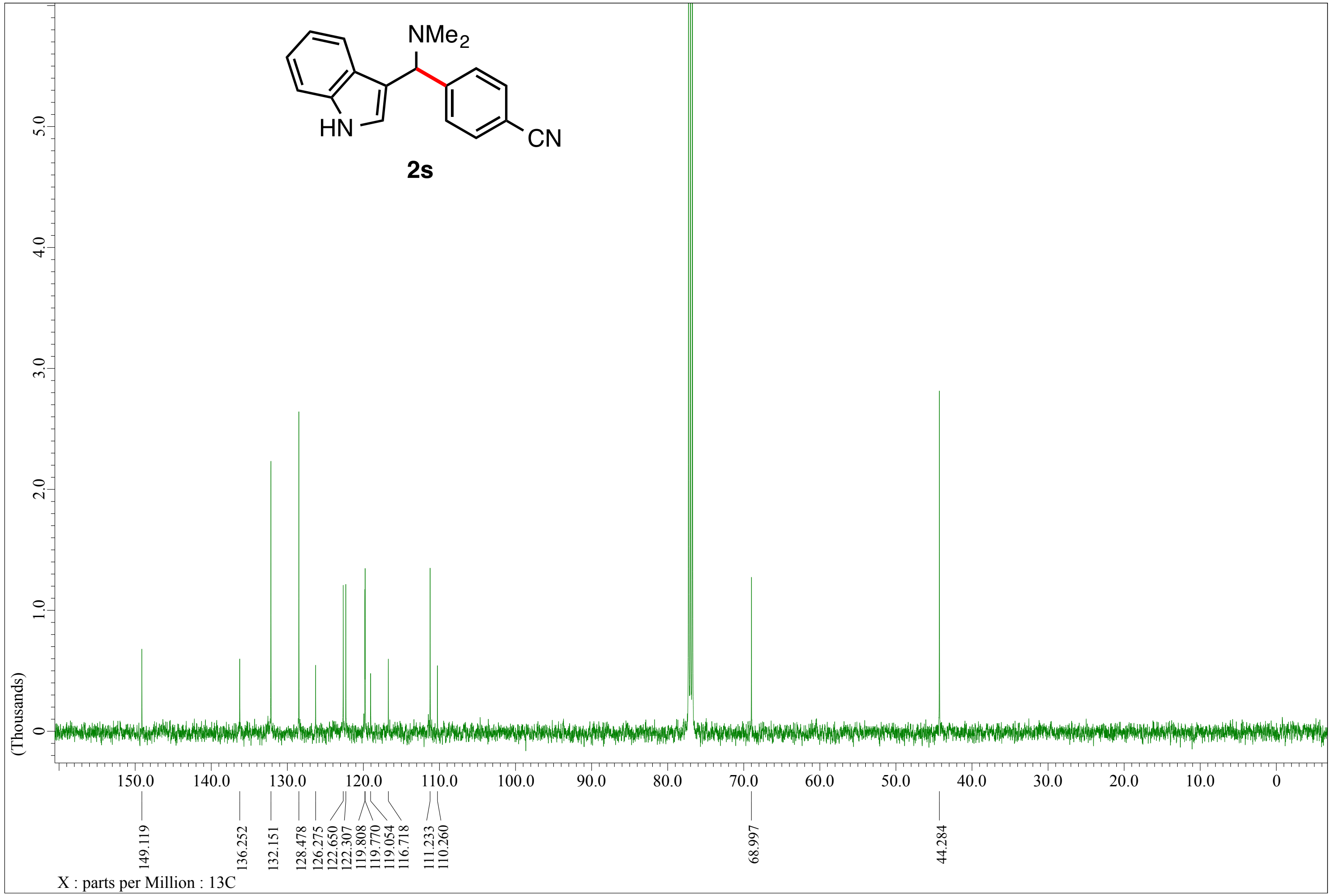

NMe2
HN
CN
2s
(Thousands)
0
1.0
2.0
3.0
4.0
5.0
150.0
140.0
130.0
120.0
110.0
100.0
90.0
80.0
70.0
60.0
50.0
40.0
30.0
20.0
10.0
0
149.119
136.252
132.151
128.478
126.275
122.650
122.307
119.808
119.770
119.054
116.718
111.233
110.260
68.997
44.284
X : parts per Million : 13C

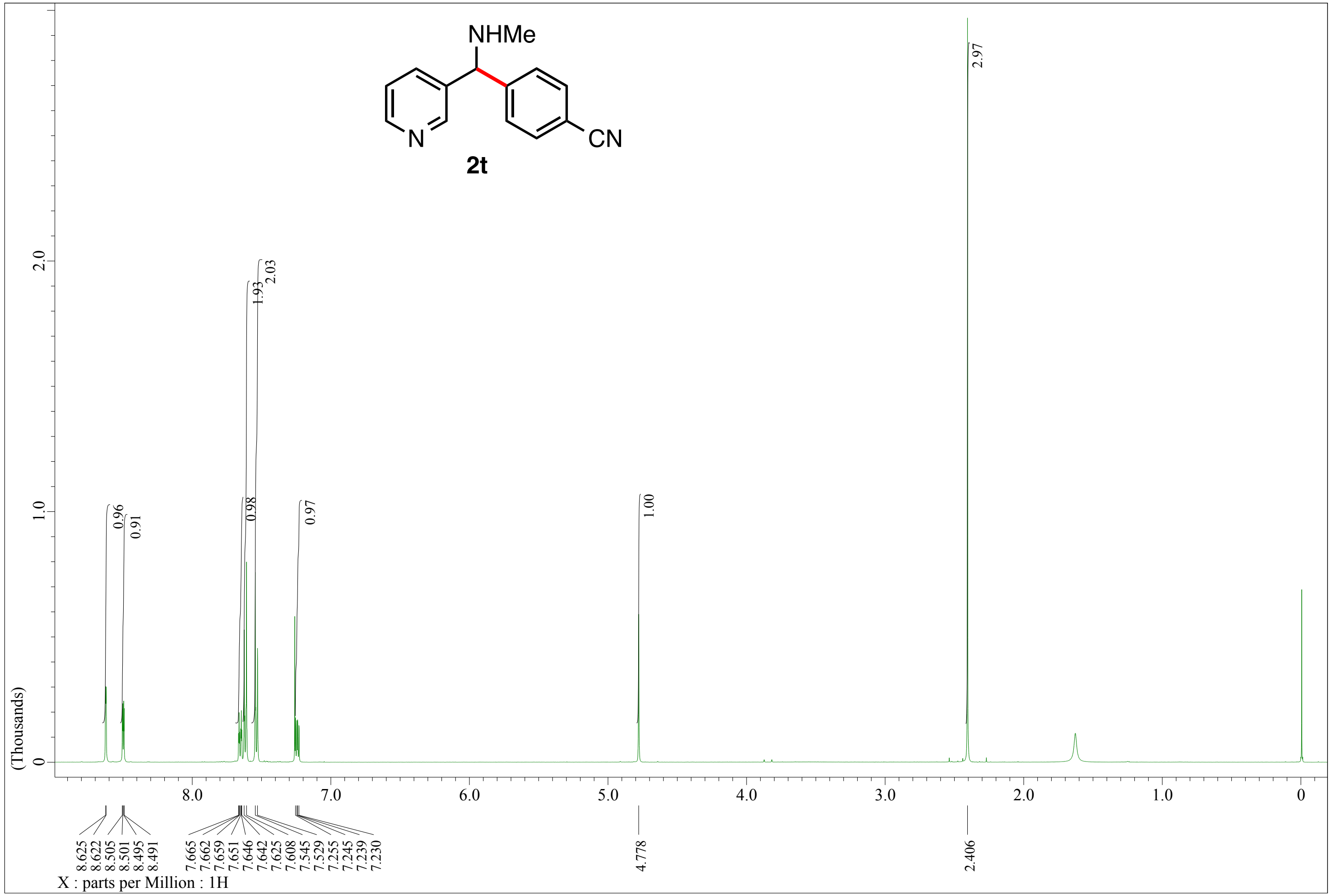
NHMe
N
CN
2t
2.97
2.03
1.93
0.98
0.96
0.91
0.97
1.00
(Thousands)
2.0
1.0
0
8.0
7.0
6.0
5.0
4.0
3.0
2.0
1.0
0
8.625
8.622
8.505
8.501
8.495
8.491
7.665
7.662
7.659
7.651
7.646
7.642
7.625
7.608
7.545
7.529
7.255
7.245
7.239
7.230
4.778
2.406
X : parts per Million : 1H

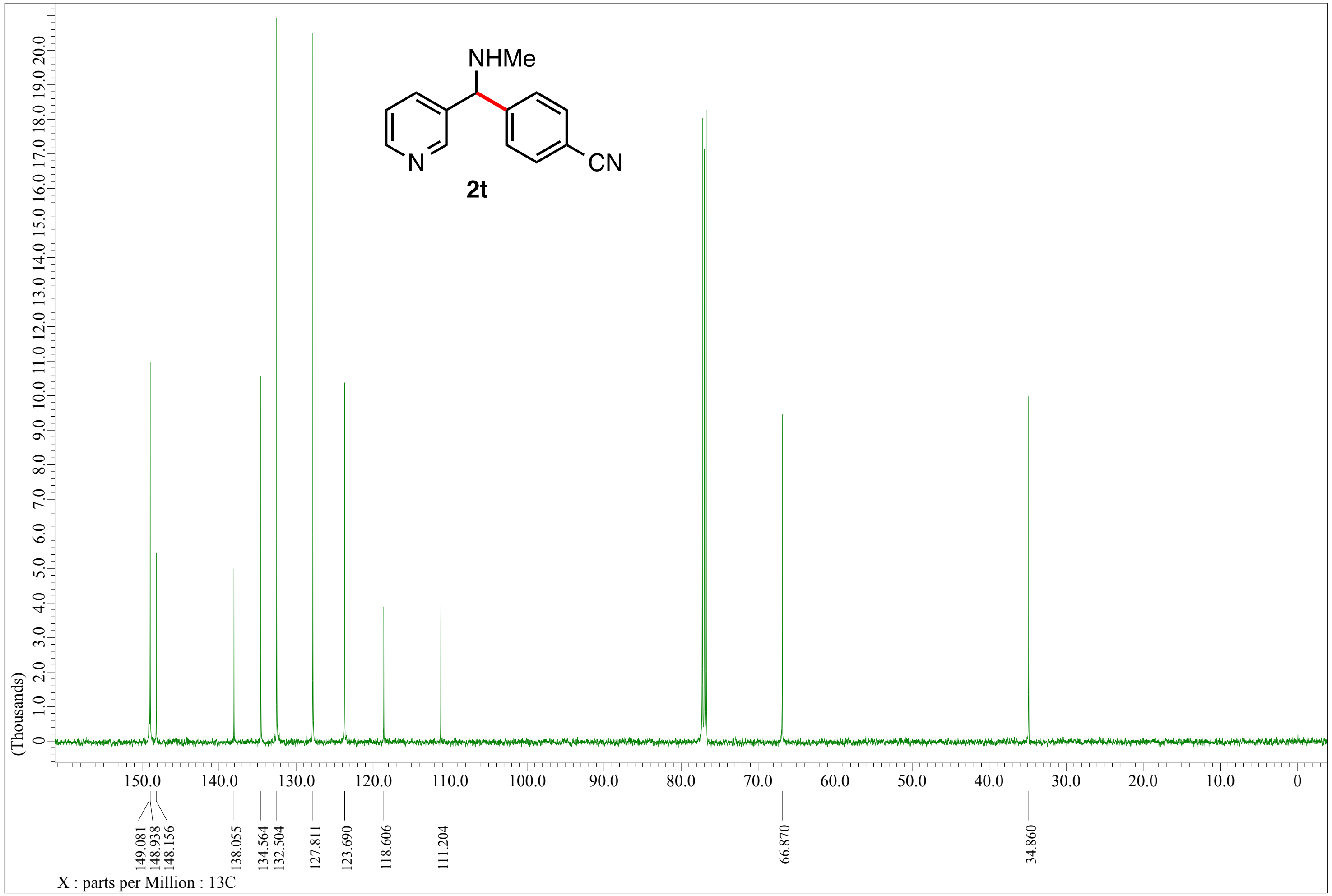
NHMe
N
CN
2t
(Thousands)
X : parts per Million : 13C
149.081
148.938
148.156
138.055
134.564
132.504
127.811
123.690
118.606
111.204
66.870
34.860

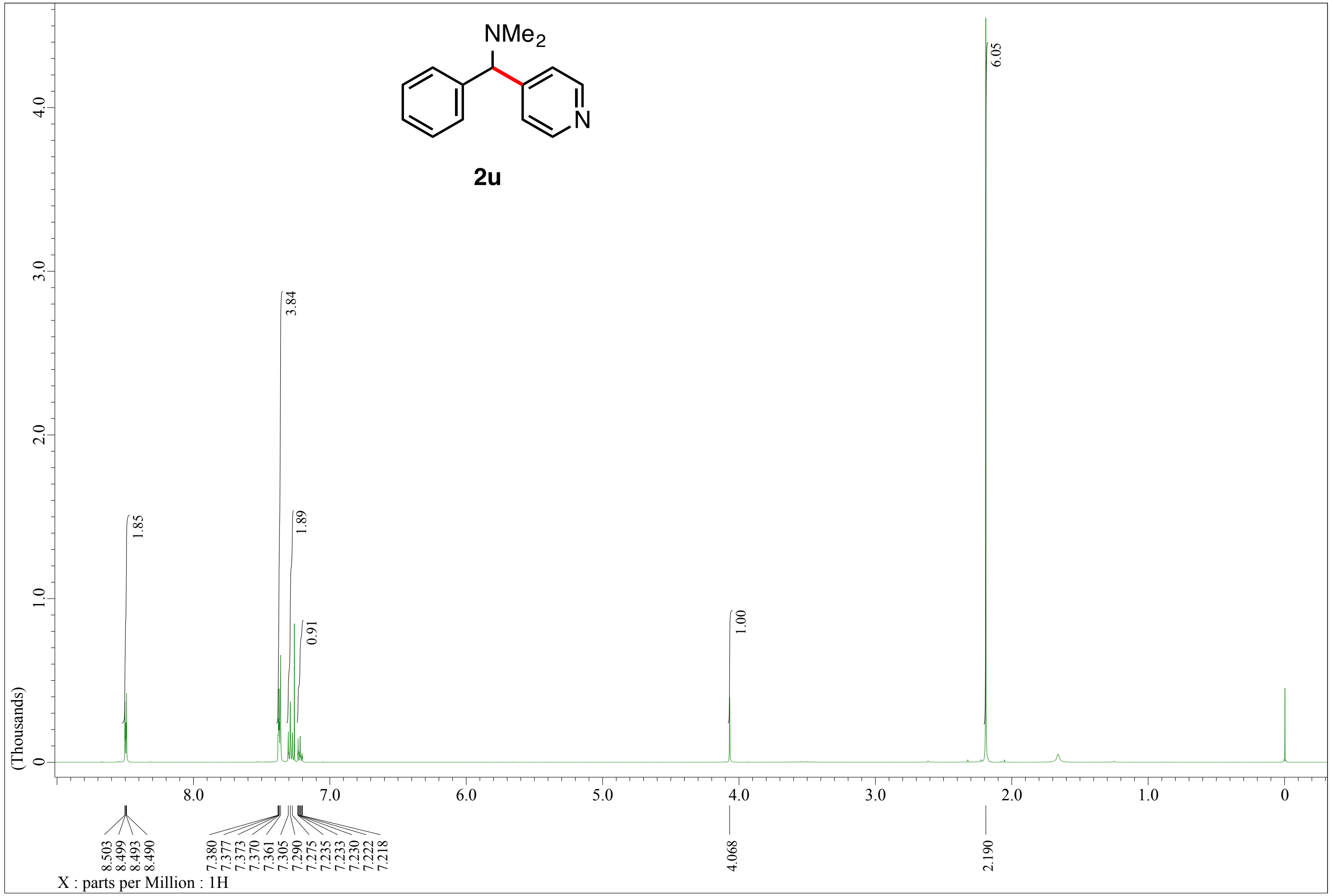
NMe2
N
2u
1.85
3.84
1.89
0.91
1.00
6.05
(Thousands)
0
1.0
2.0
3.0
4.0
8.0
7.0
6.0
5.0
4.0
3.0
2.0
1.0
0
8.503
8.499
8.493
8.490
7.380
7.377
7.373
7.370
7.361
7.305
7.290
7.275
7.235
7.233
7.230
7.222
7.218
4.068
2.190
X : parts per Million : 1H

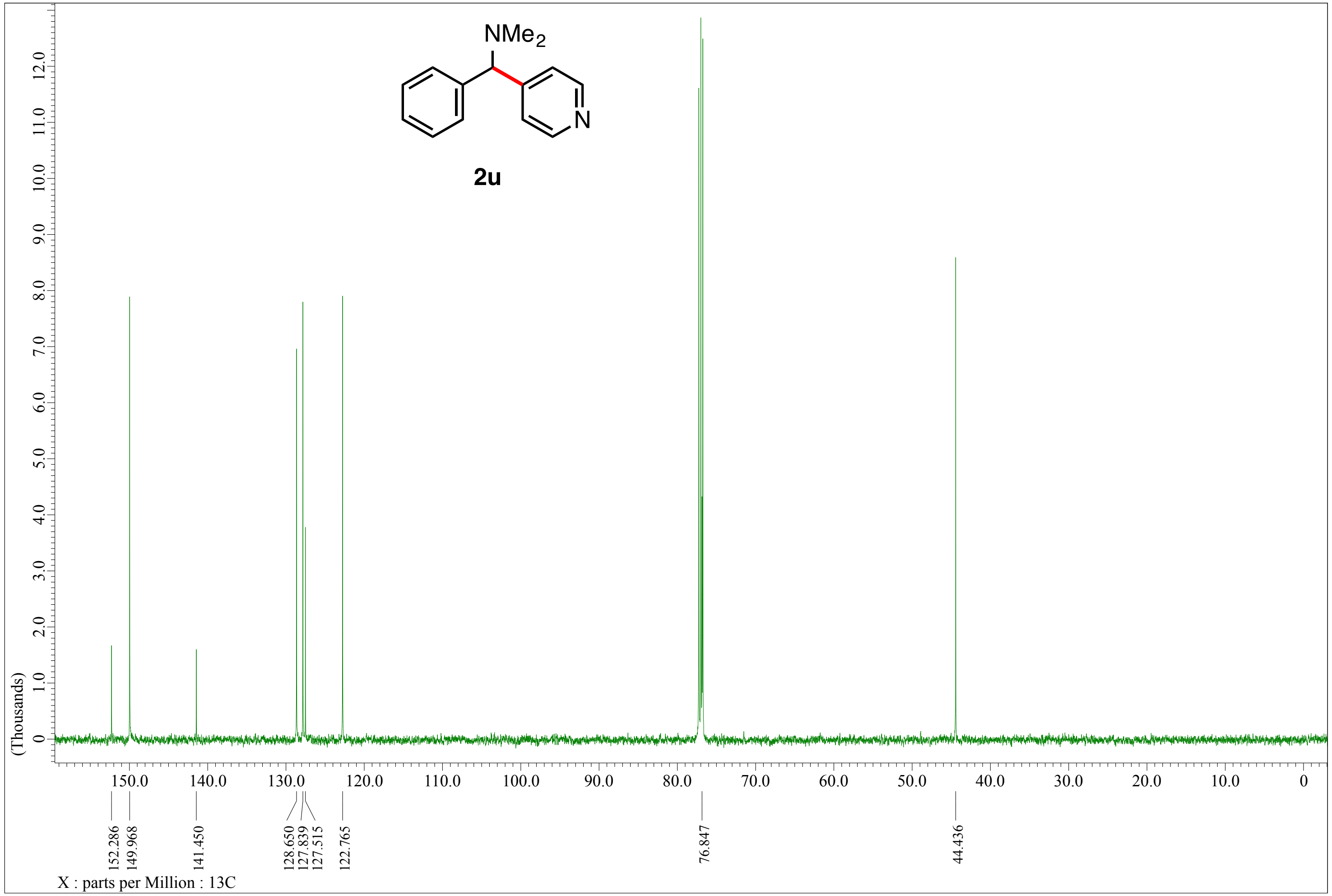

NMe2
N
2u
(Thousands)
X : parts per Million : 13C
152.286
149.968
141.450
128.650
127.839
127.515
122.765
76.847
44.436

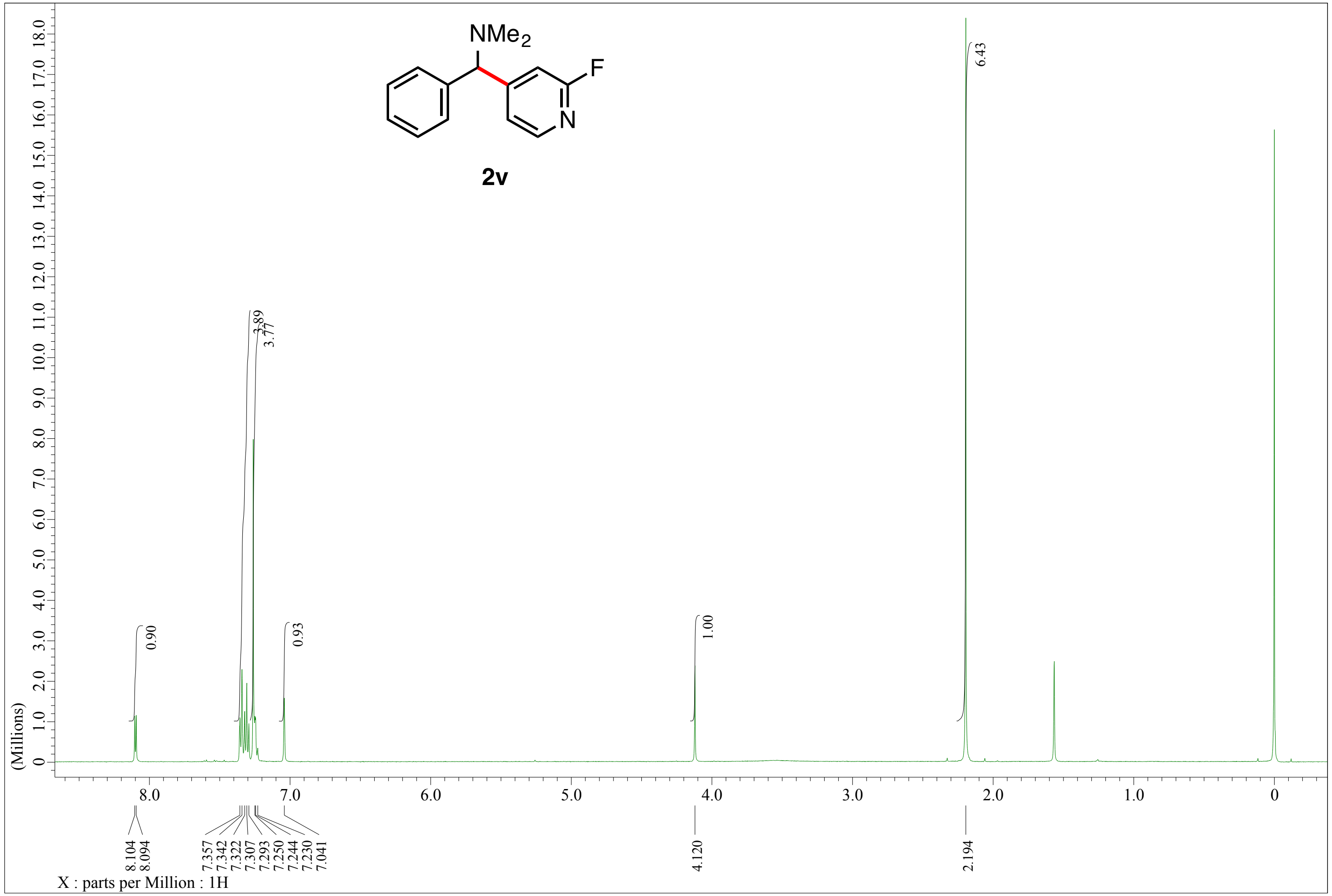

NMe2
F
N
2v
(Millions)
X : parts per Million : 1H
0.90
3.89
3.77
0.93
1.00
6.43
8.104
8.094
7.357
7.342
7.322
7.307
7.293
7.250
7.244
7.230
7.041
4.120
2.194

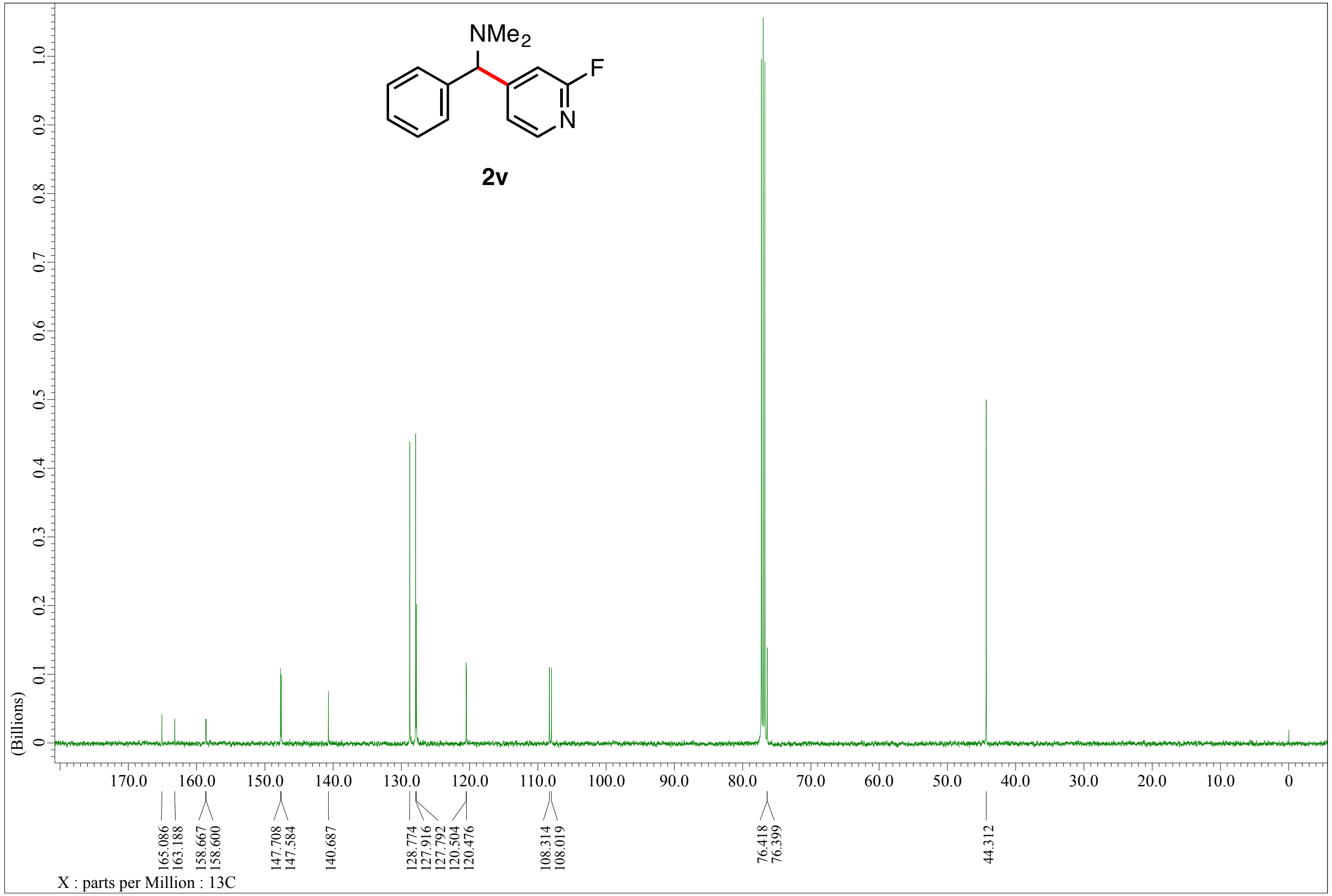
NMe2
F
N
2v
(Billions)
X : parts per Million : 13C
165.086
163.188
158.667
158.600
147.708
147.584
140.687
128.774
127.916
127.792
120.504
120.476
108.314
108.019
76.418
76.399
44.312

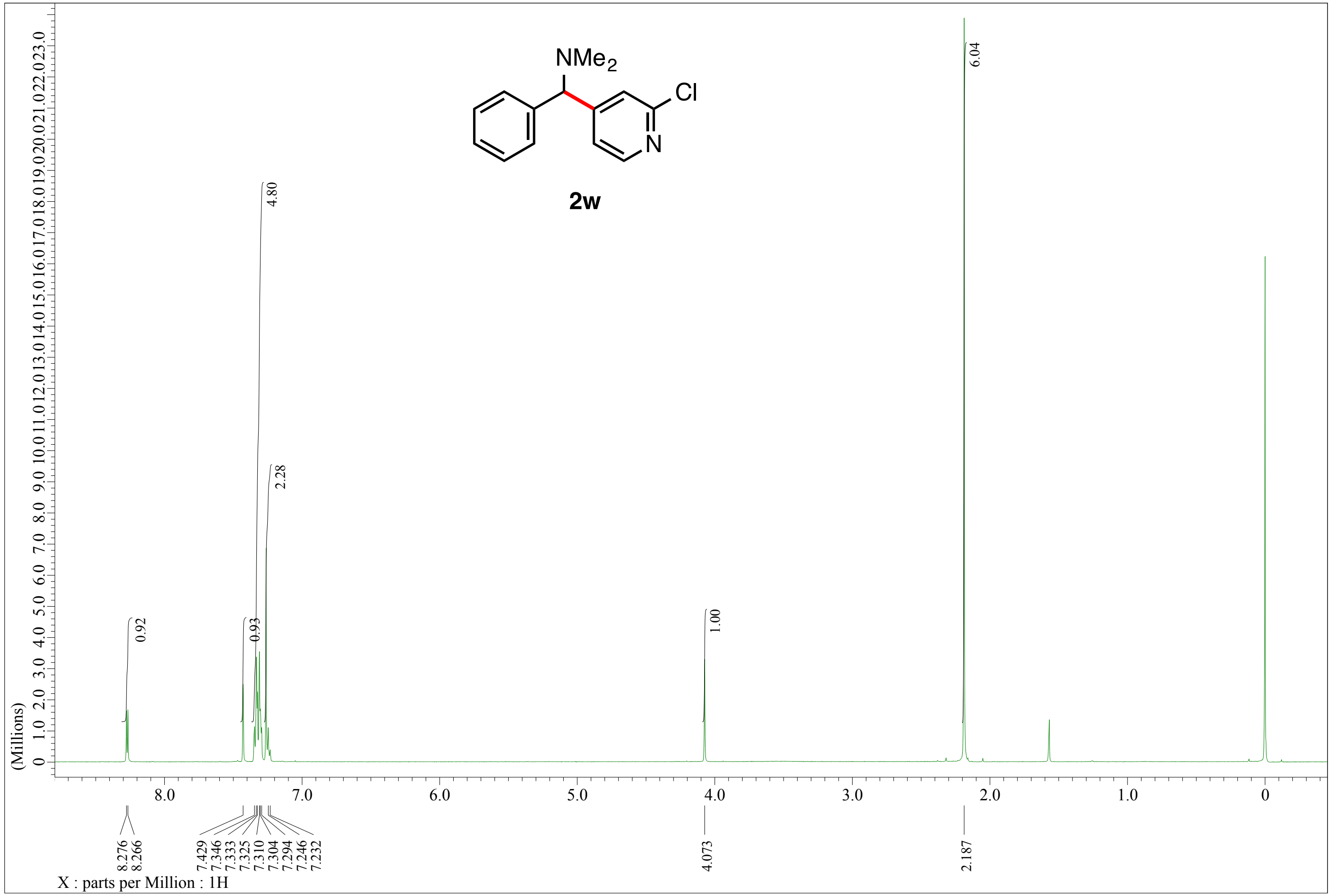
NMe2
Cl
N
2w
(Millions)
X : parts per Million : 1H
0.92
0.93
4.80
2.28
1.00
6.04
8.276
8.266
7.429
7.346
7.333
7.325
7.310
7.304
7.294
7.246
7.232
4.073
2.187

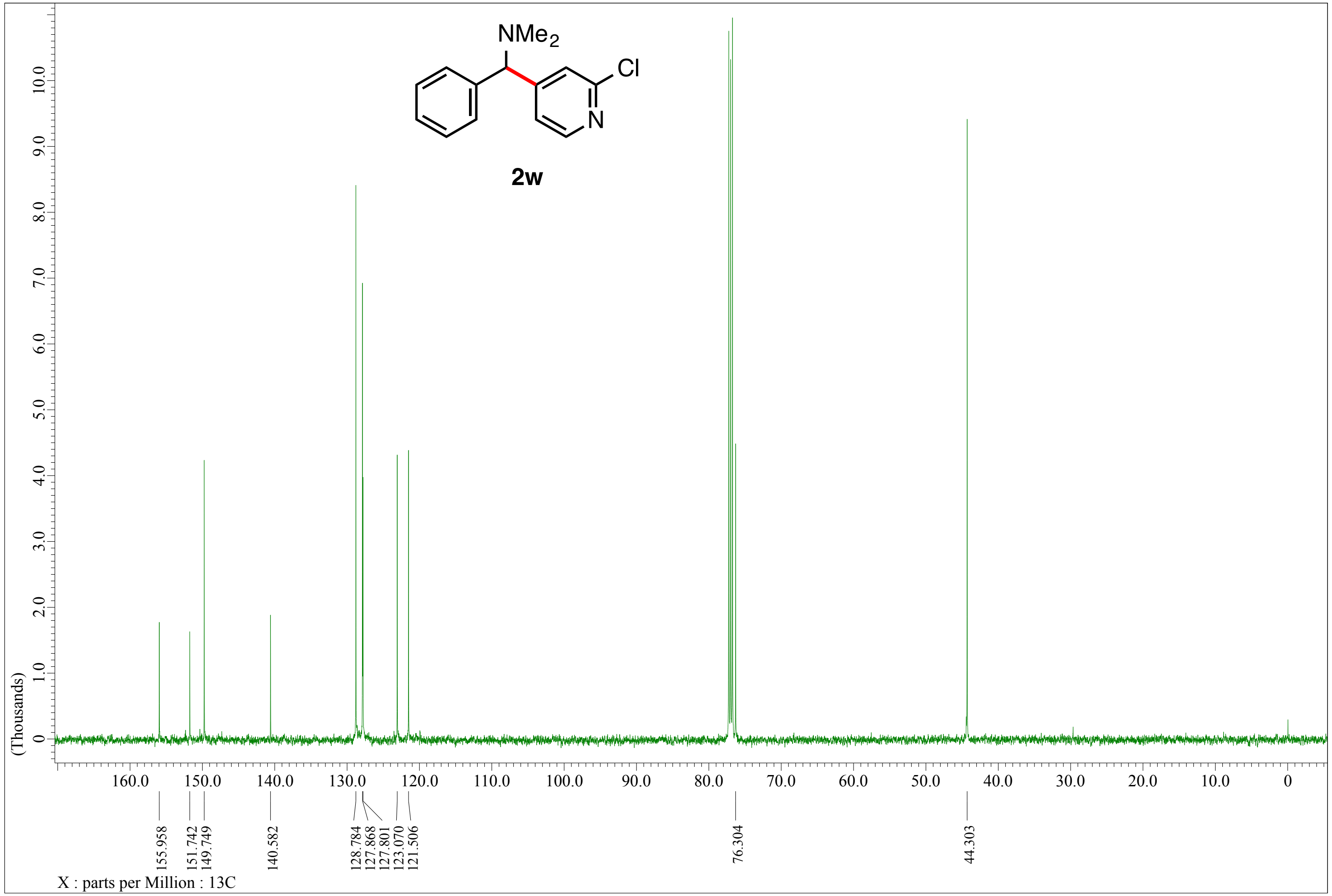
NMe2
Cl
N
2w
155.958
151.742
149.749
140.582
128.784
127.868
127.801
123.070
121.506
76.304
44.303
X : parts per Million : 13C
(Thousands)

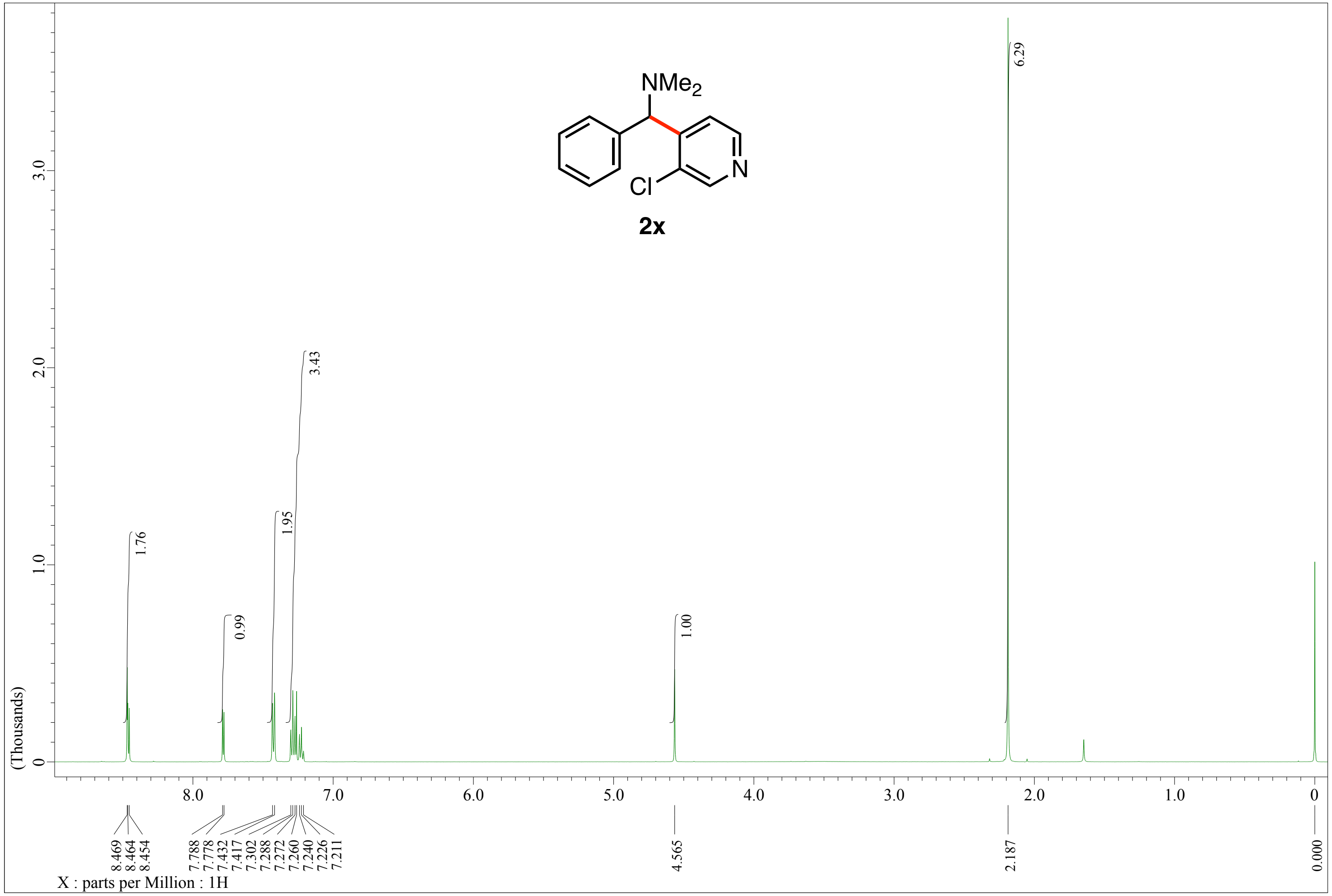
NMe2
Cl
N
2x
6.29
3.43
1.95
1.76
0.99
1.00
3.0
2.0
1.0
0
(Thousands)
8.0
7.0
6.0
5.0
4.0
3.0
2.0
1.0
0
8.469
8.464
8.454
7.788
7.778
7.432
7.417
7.302
7.288
7.272
7.260
7.240
7.226
7.211
4.565
2.187
0.000
X : parts per Million : 1H

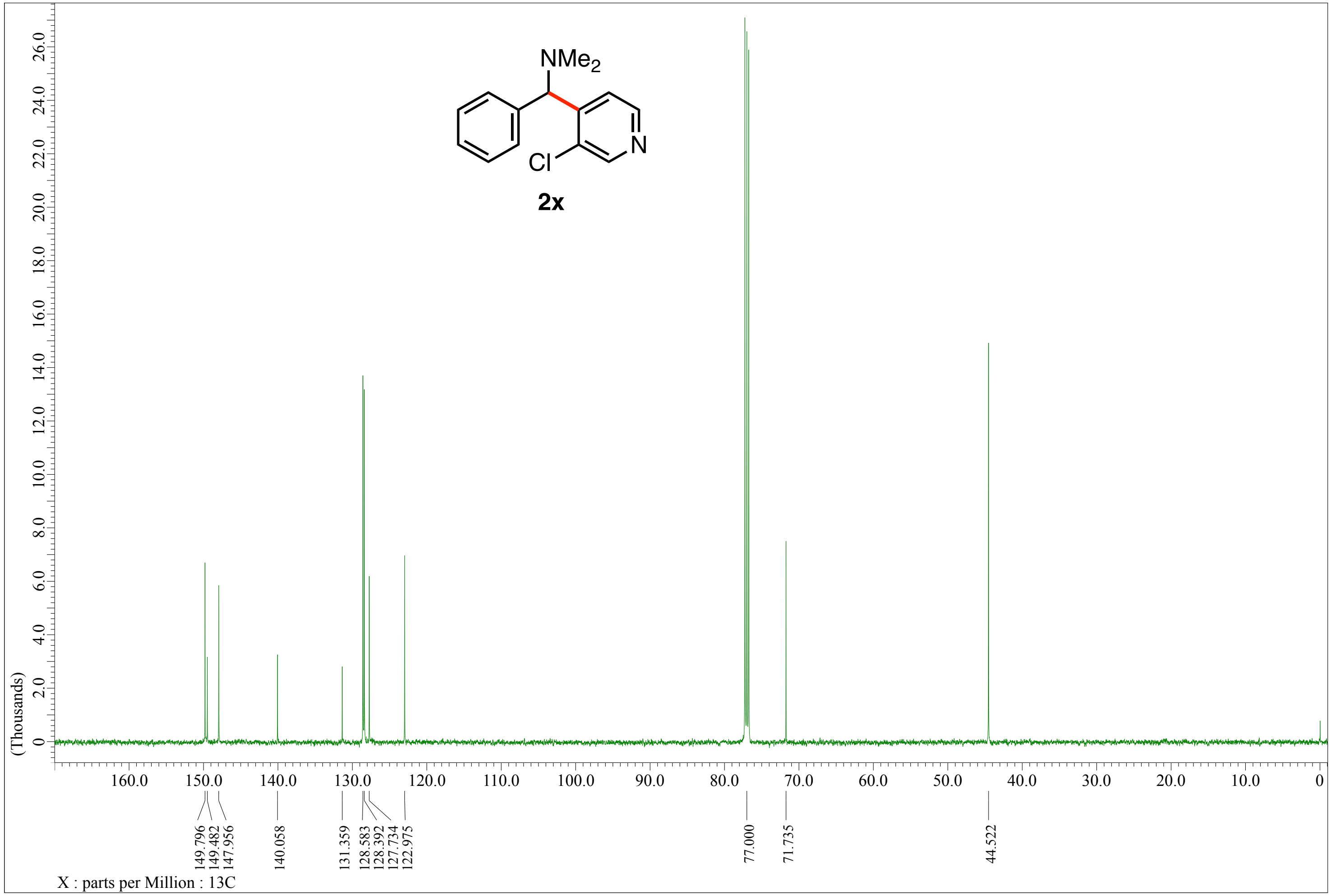
NMe2
Cl
N
2x
(Thousands)
0
2.0
4.0
6.0
8.0
10.0
12.0
14.0
16.0
18.0
20.0
22.0
24.0
26.0
160.0
150.0
140.0
130.0
120.0
110.0
100.0
90.0
80.0
70.0
60.0
50.0
40.0
30.0
20.0
10.0
0
149.796
149.482
147.956
140.058
131.359
128.583
128.392
127.734
122.975
77.000
71.735
44.522
X : parts per Million : 13C

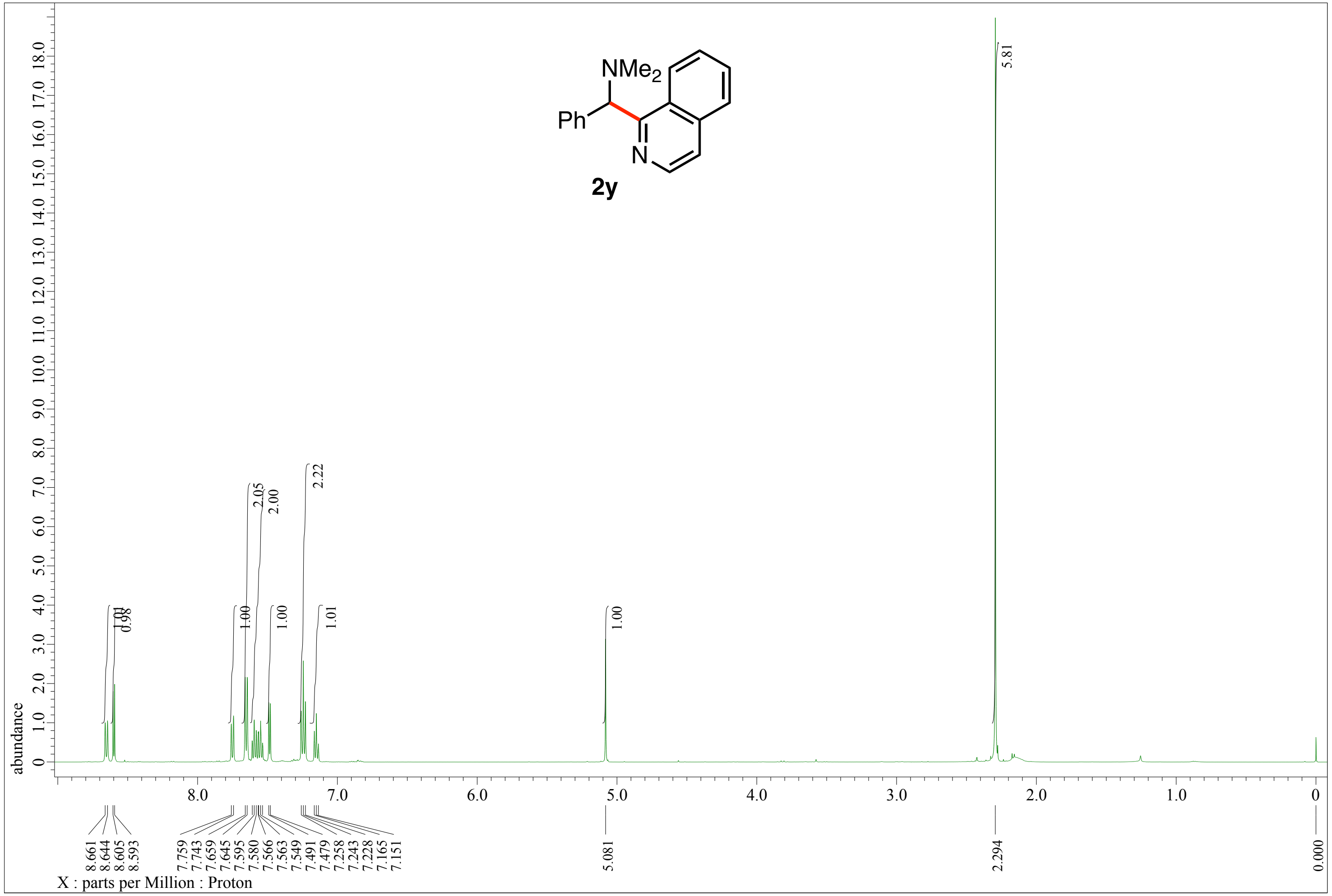

NMe2
Ph
N
2y
abundance
X : parts per Million : Proton
8.661
8.644
8.605
8.593
7.759
7.743
7.659
7.645
7.595
7.580
7.566
7.563
7.549
7.491
7.479
7.258
7.243
7.228
7.165
7.151
5.081
2.294
0.000
5.81
2.22
2.05
2.00
1.01
0.98
1.00
1.00
1.01
1.00

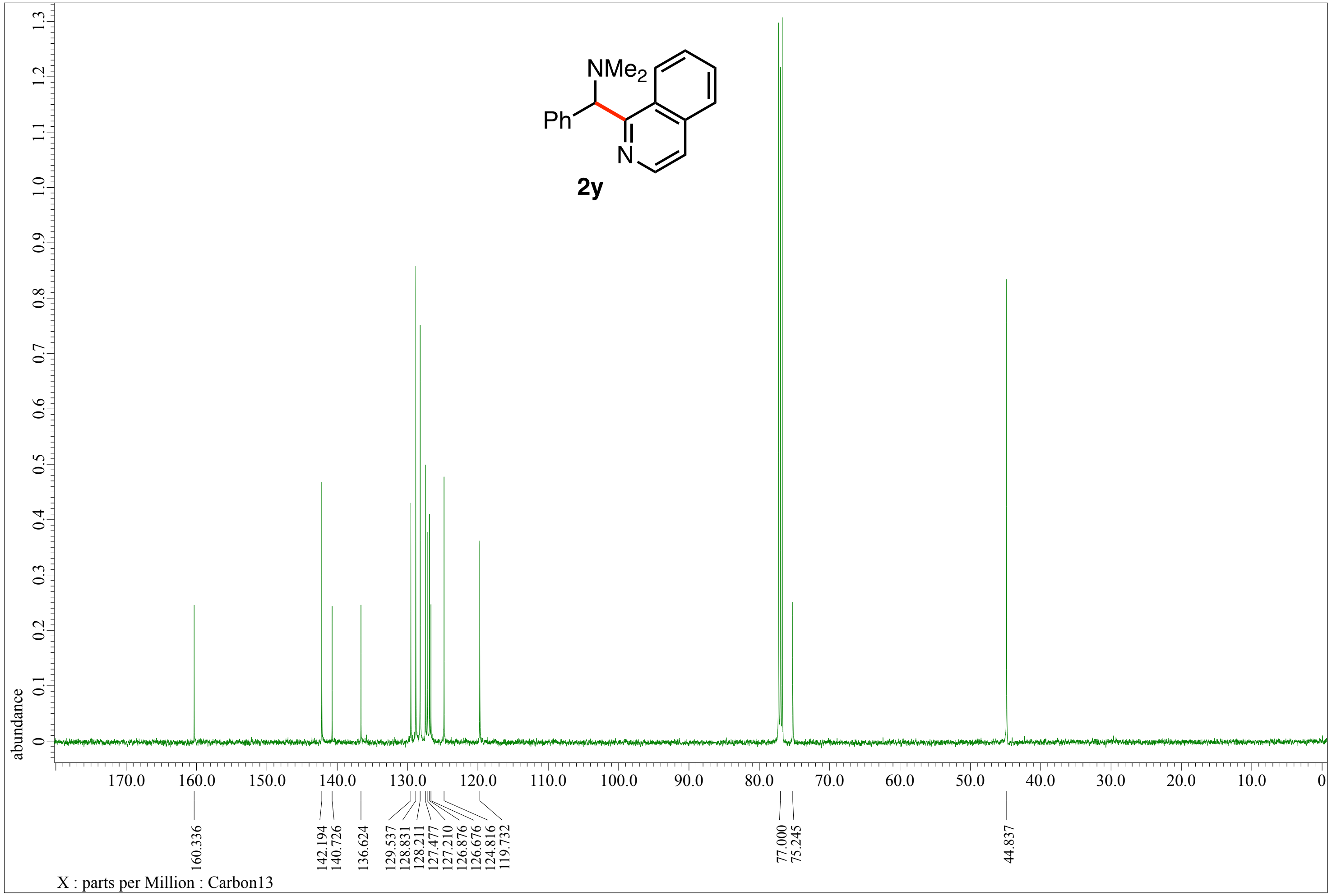
NMe2
Ph
N
2y
abundance
X : parts per Million : Carbon13
160.336
142.194
140.726
136.624
129.537
128.831
128.211
127.477
127.210
126.876
126.676
124.816
119.732
77.000
75.245
44.837

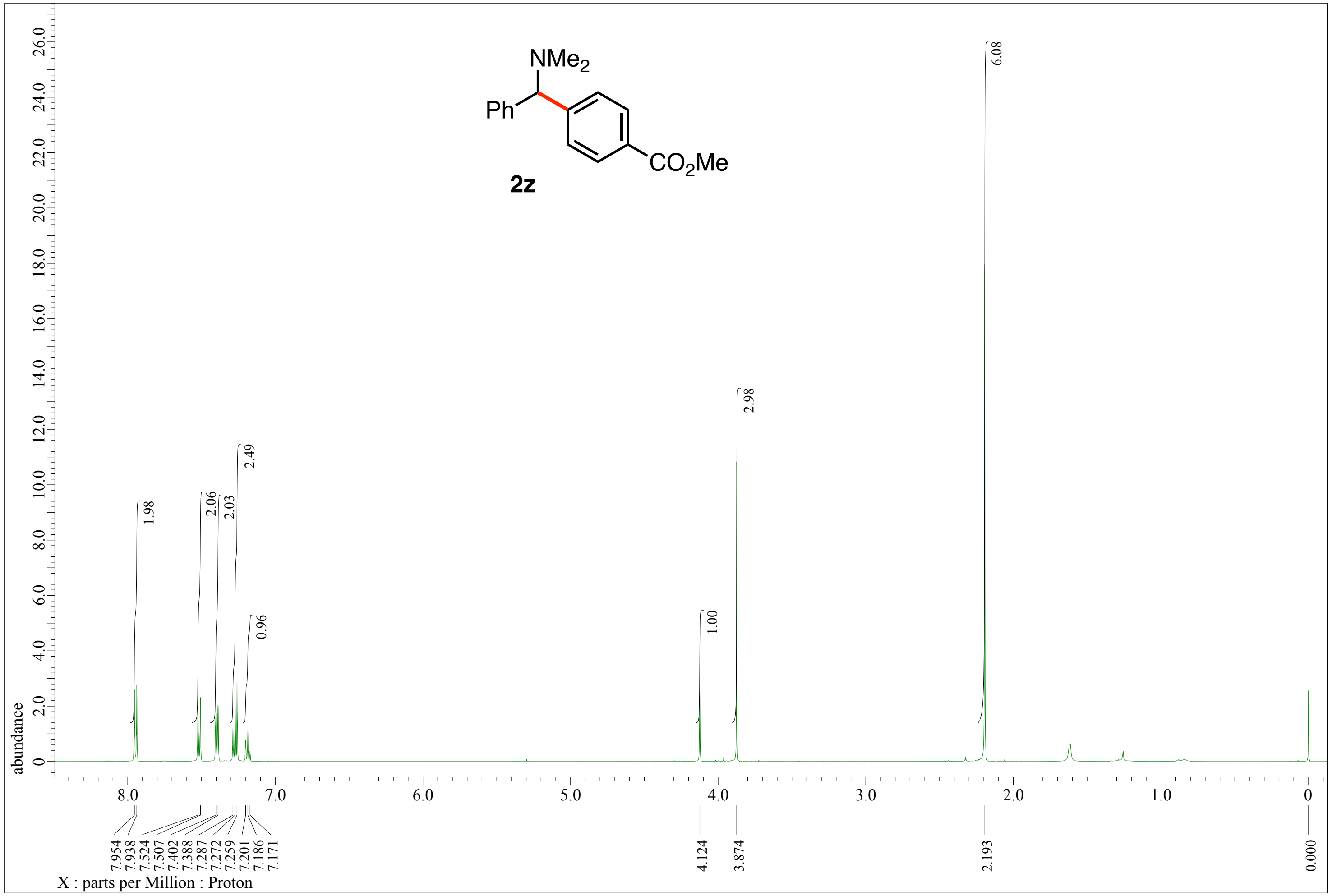
NMe2
Ph
CO2Me
2z
abundance
X : parts per Million : Proton
1.98
2.06
2.03
2.49
0.96
1.00
2.98
6.08
7.954
7.938
7.524
7.507
7.402
7.388
7.287
7.272
7.259
7.201
7.186
7.171
4.124
3.874
2.193
0.000

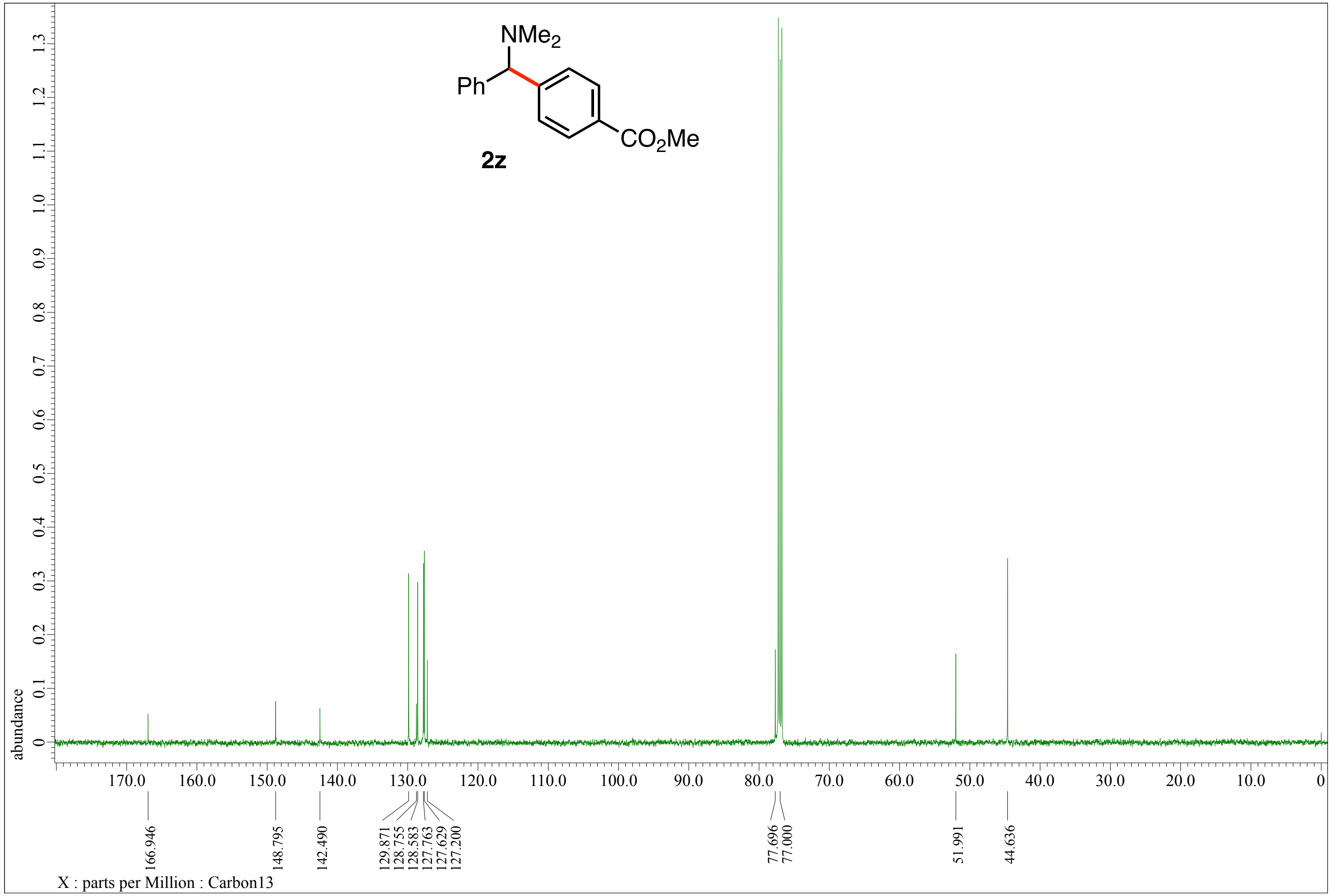
NMe2
Ph
CO2Me
2z
abundance
0 0.1 0.2 0.3 0.4 0.5 0.6 0.7 0.8 0.9 1.0 1.1 1.2 1.3
170.0 160.0 150.0 140.0 130.0 120.0 110.0 100.0 90.0 80.0 70.0 60.0 50.0 40.0 30.0 20.0 10.0 0
166.946
148.795
142.490
129.871
128.755
128.583
127.763
127.629
127.200
77.696
77.000
51.991
44.636
X : parts per Million : Carbon13

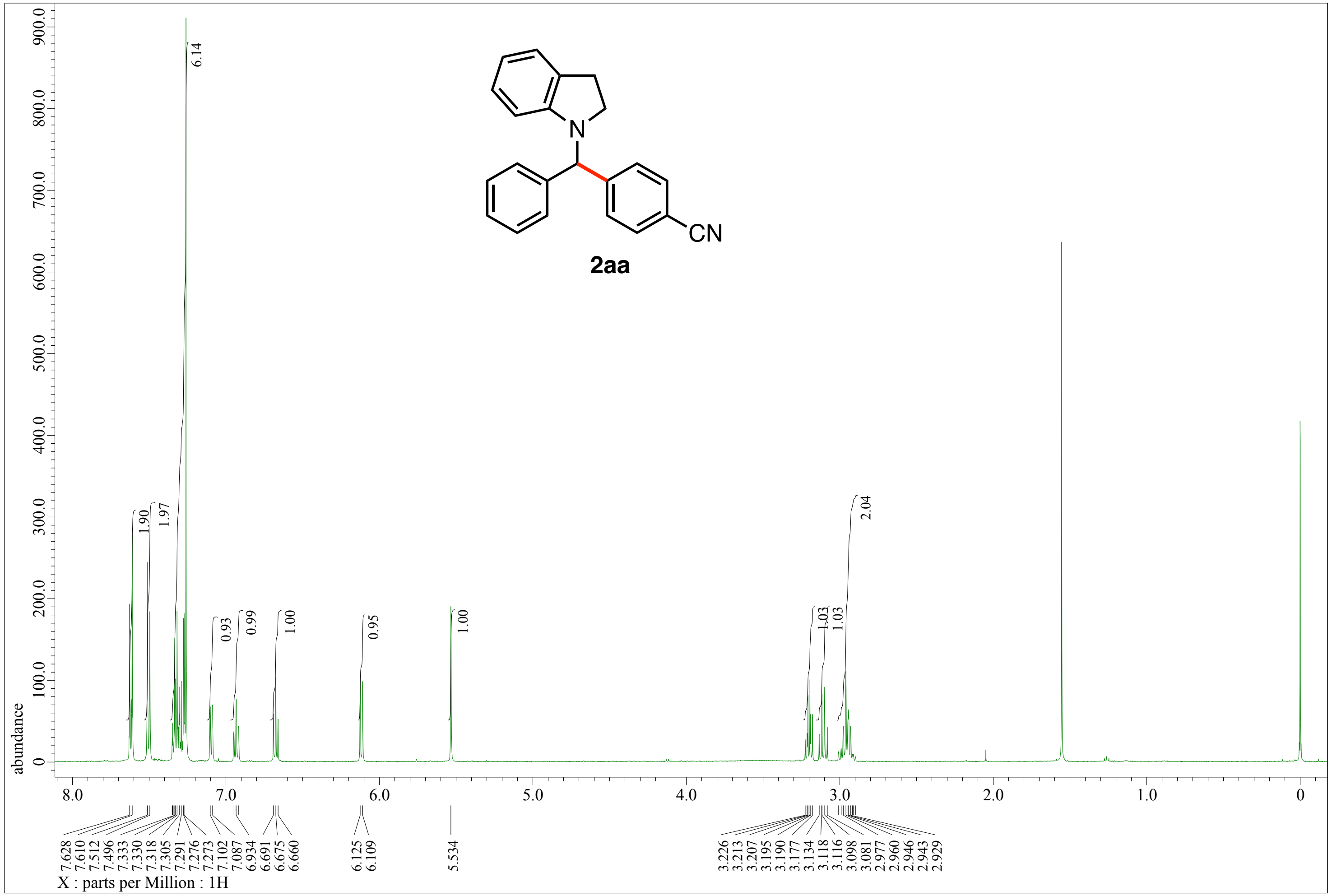

2aa
CN
N
abundance
X : parts per Million : 1H

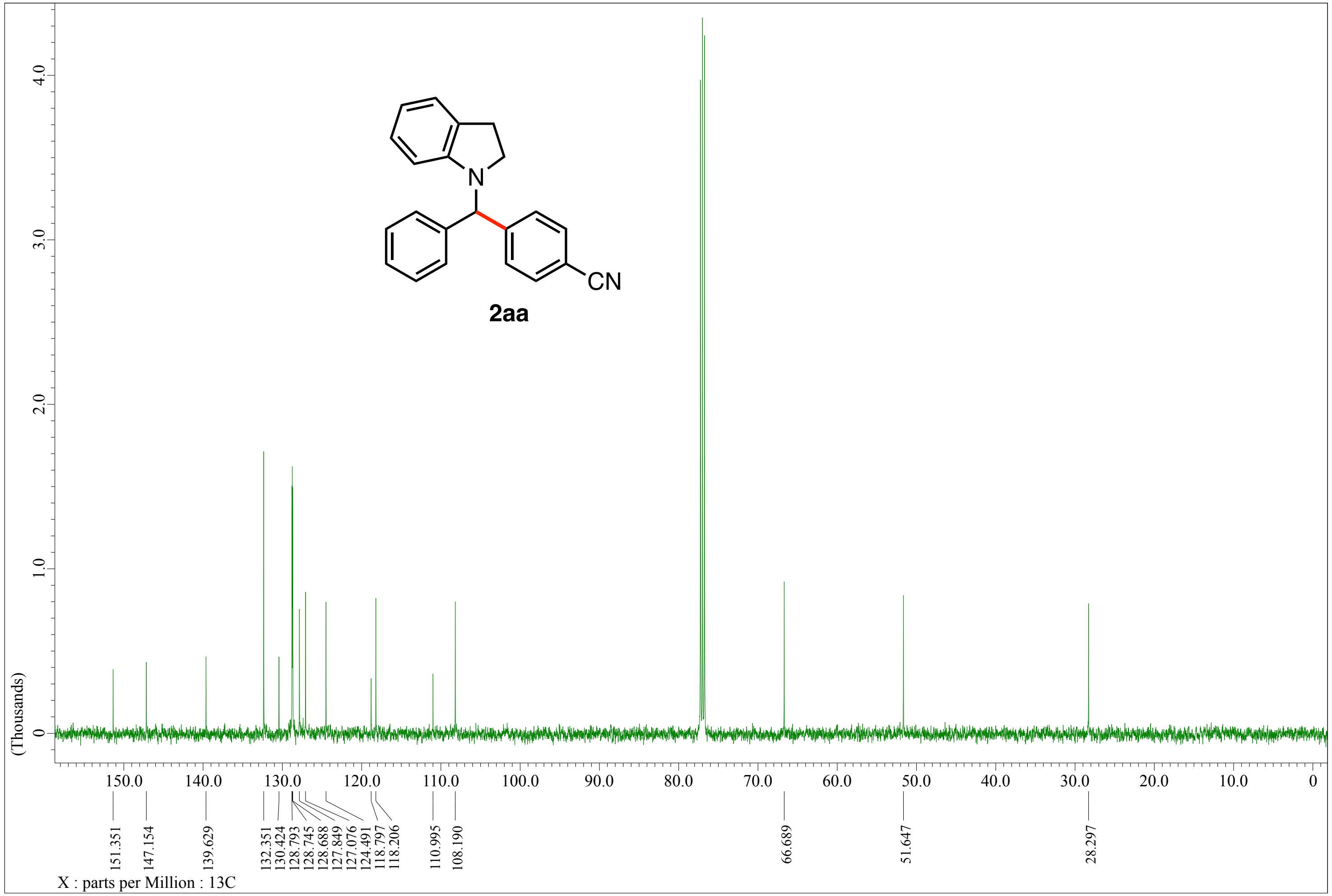

2aa
CN
N
(Thousands)
X : parts per Million : 13C
151.351
147.154
139.629
132.351
130.424
128.793
128.745
128.688
127.849
127.076
124.491
118.797
118.206
110.995
108.190
66.689
51.647
28.297

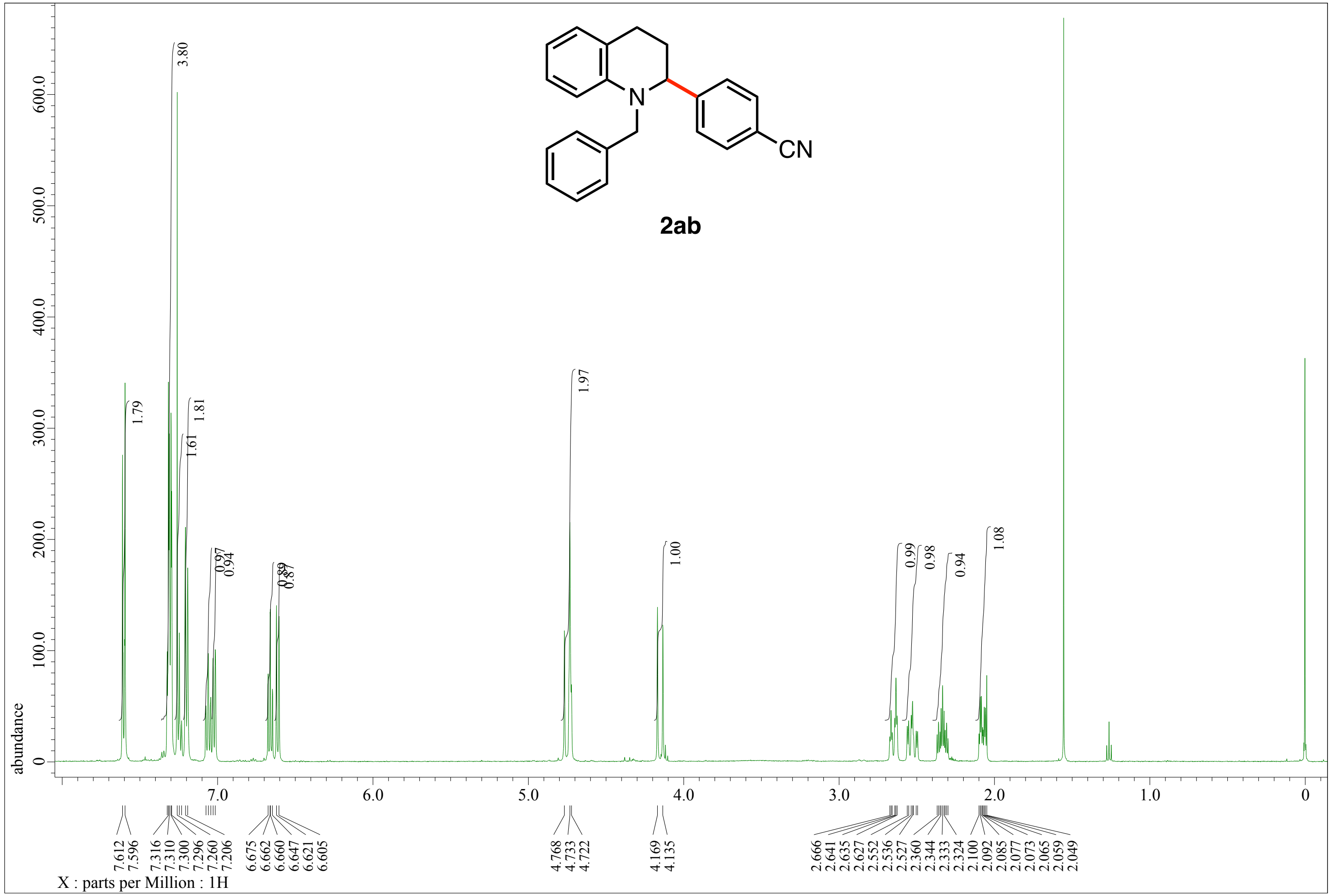

CN
2ab
abundance
X : parts per Million : 1H

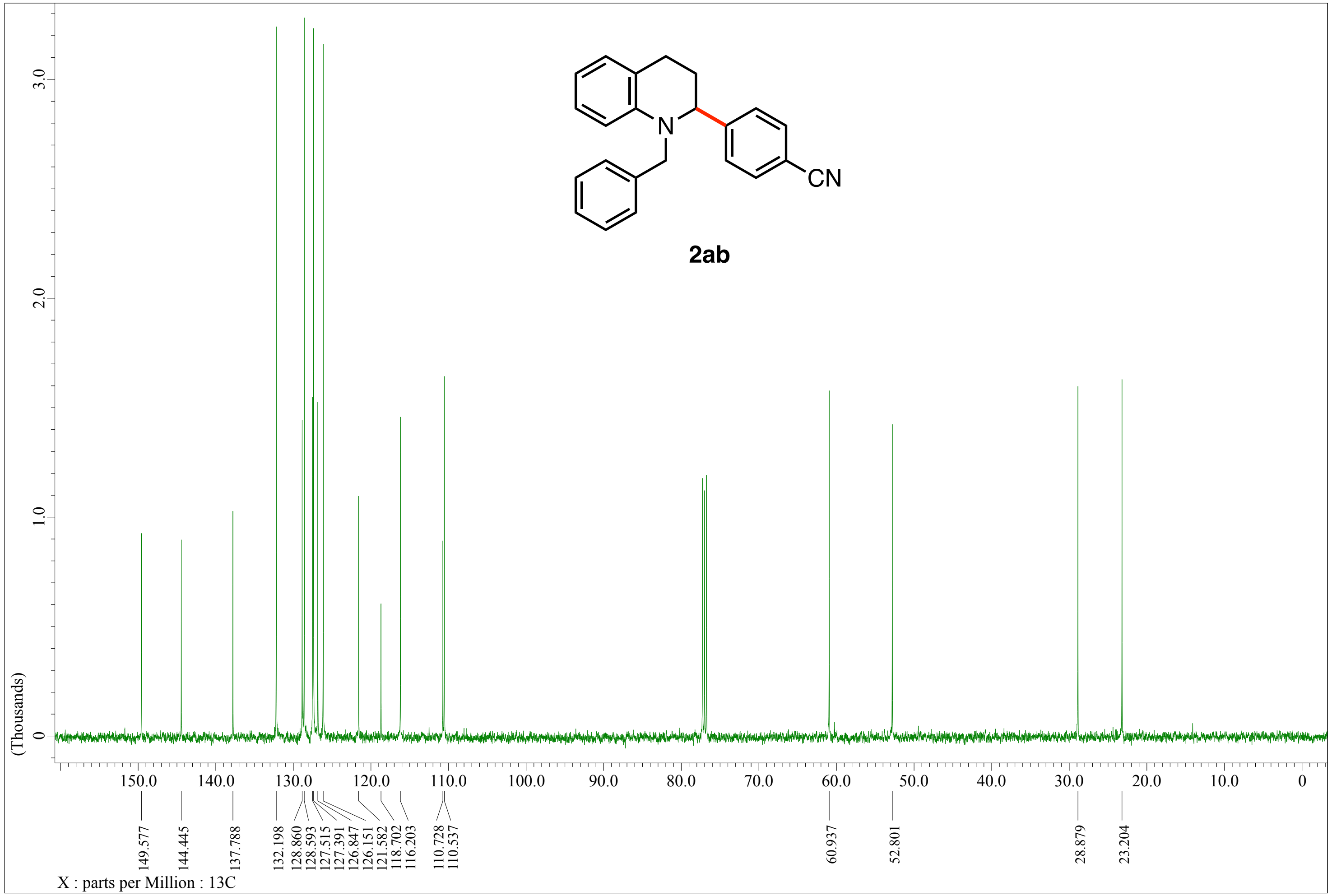

2ab
CN
N
3.0
2.0
1.0
0
(Thousands)
150.0
140.0
130.0
120.0
110.0
100.0
90.0
80.0
70.0
60.0
50.0
40.0
30.0
20.0
10.0
0
149.577
144.445
137.788
132.198
128.860
128.593
127.515
127.391
126.847
126.151
121.582
118.702
116.203
110.728
110.537
60.937
52.801
28.879
23.204
X : parts per Million : 13C

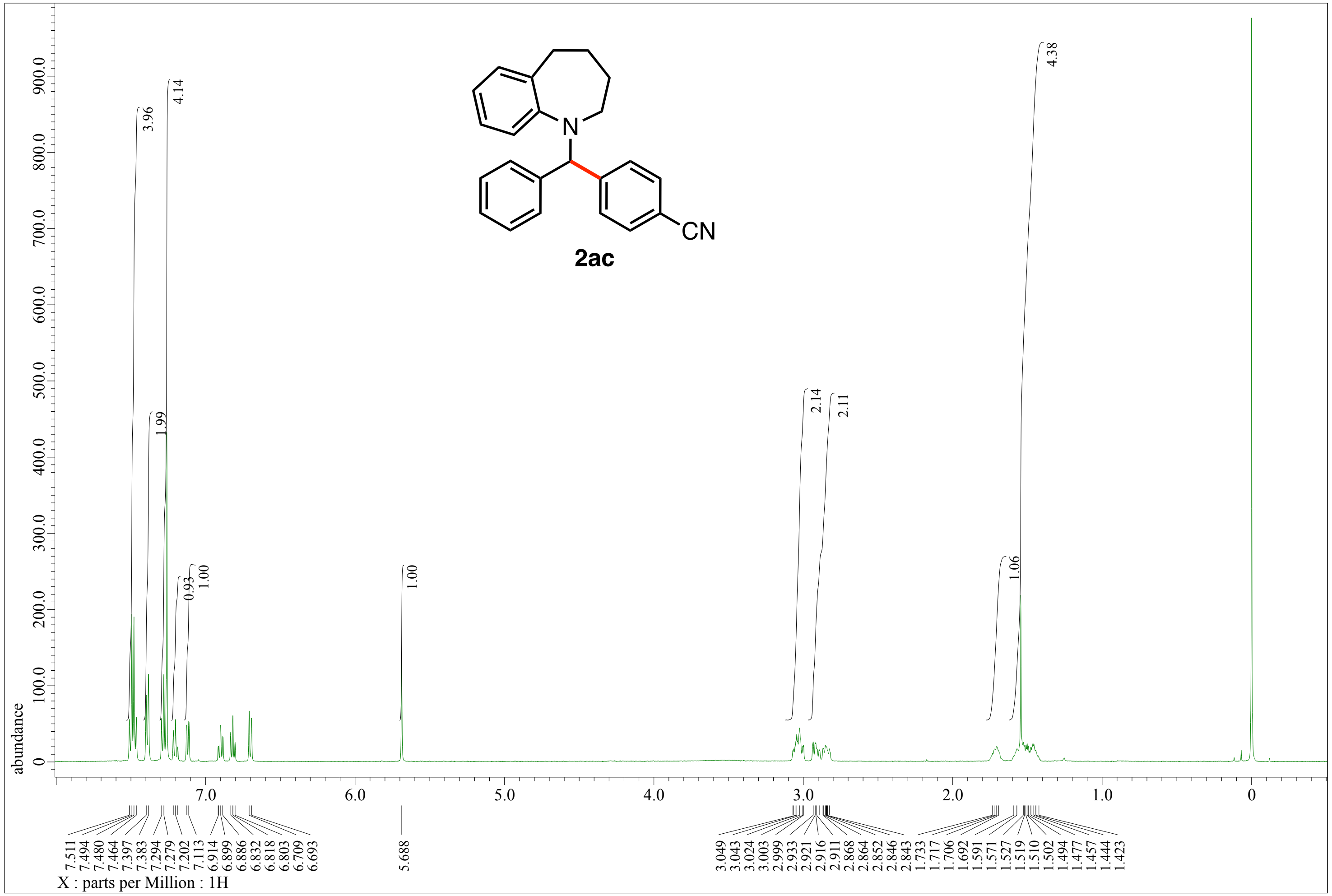
2ac
CN
N
abundance
0
100.0
200.0
300.0
400.0
500.0
600.0
700.0
800.0
900.0
3.96
4.14
1.99
0.93
1.00
1.00
2.14
2.11
1.06
4.38
7.0
6.0
5.0
4.0
3.0
2.0
1.0
0
7.511 7.494 7.480 7.464 7.397 7.383 7.294 7.279 7.202 7.113 6.914 6.899 6.886 6.832 6.818 6.803 6.709 6.693
5.688
3.049 3.043 3.024 3.003 2.999 2.933 2.921 2.916 2.911 2.868 2.864 2.852 2.846 2.843 1.733 1.717 1.706 1.692 1.591 1.571 1.527 1.519 1.510 1.502 1.494 1.477 1.457 1.444 1.423
X : parts per Million : 1H

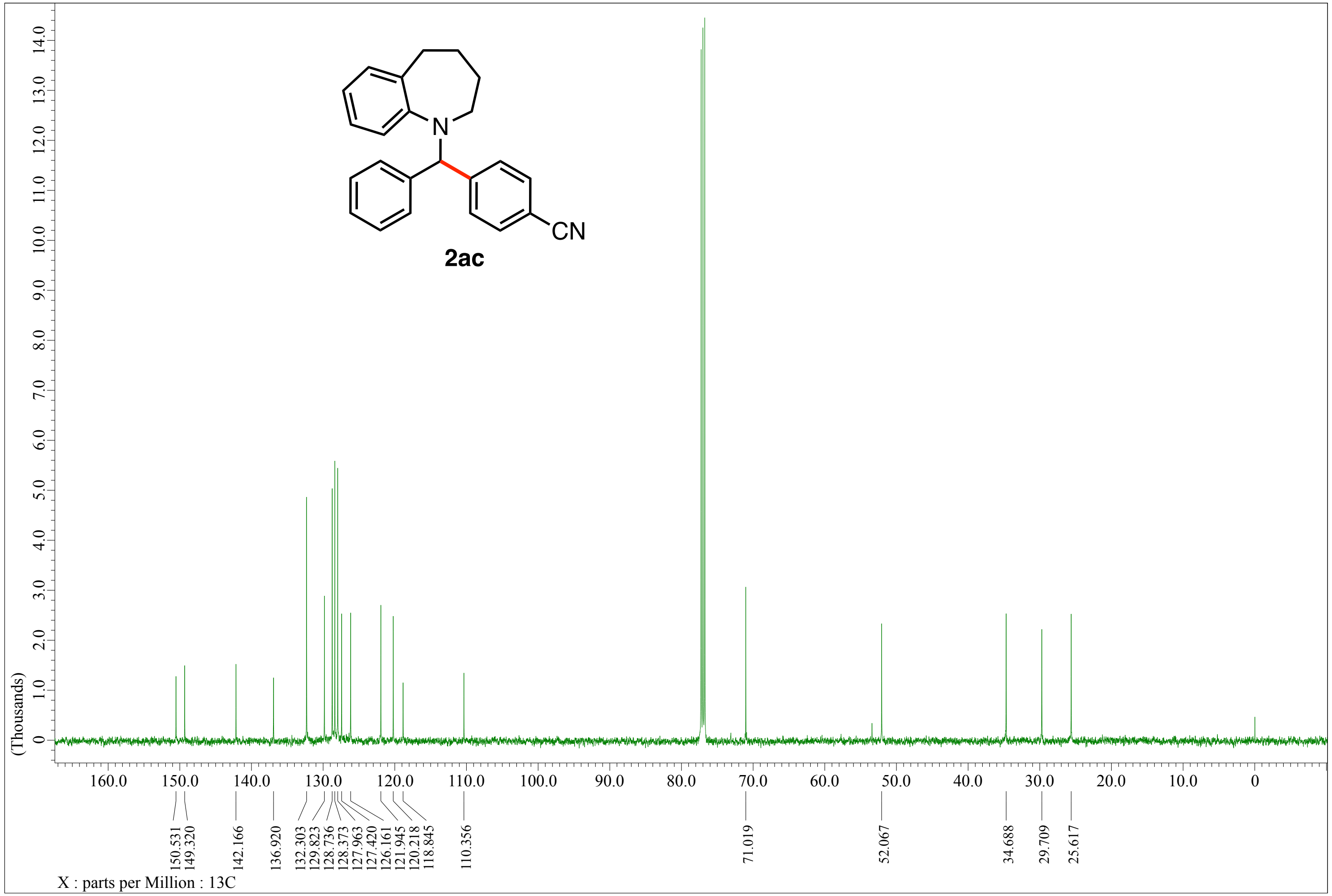

CN
2ac
(Thousands)
X : parts per Million : 13C
150.531
149.320
142.166
136.920
132.303
129.823
128.736
128.373
127.963
127.420
126.161
121.945
120.218
118.845
110.356
71.019
52.067
34.688
29.709
25.617

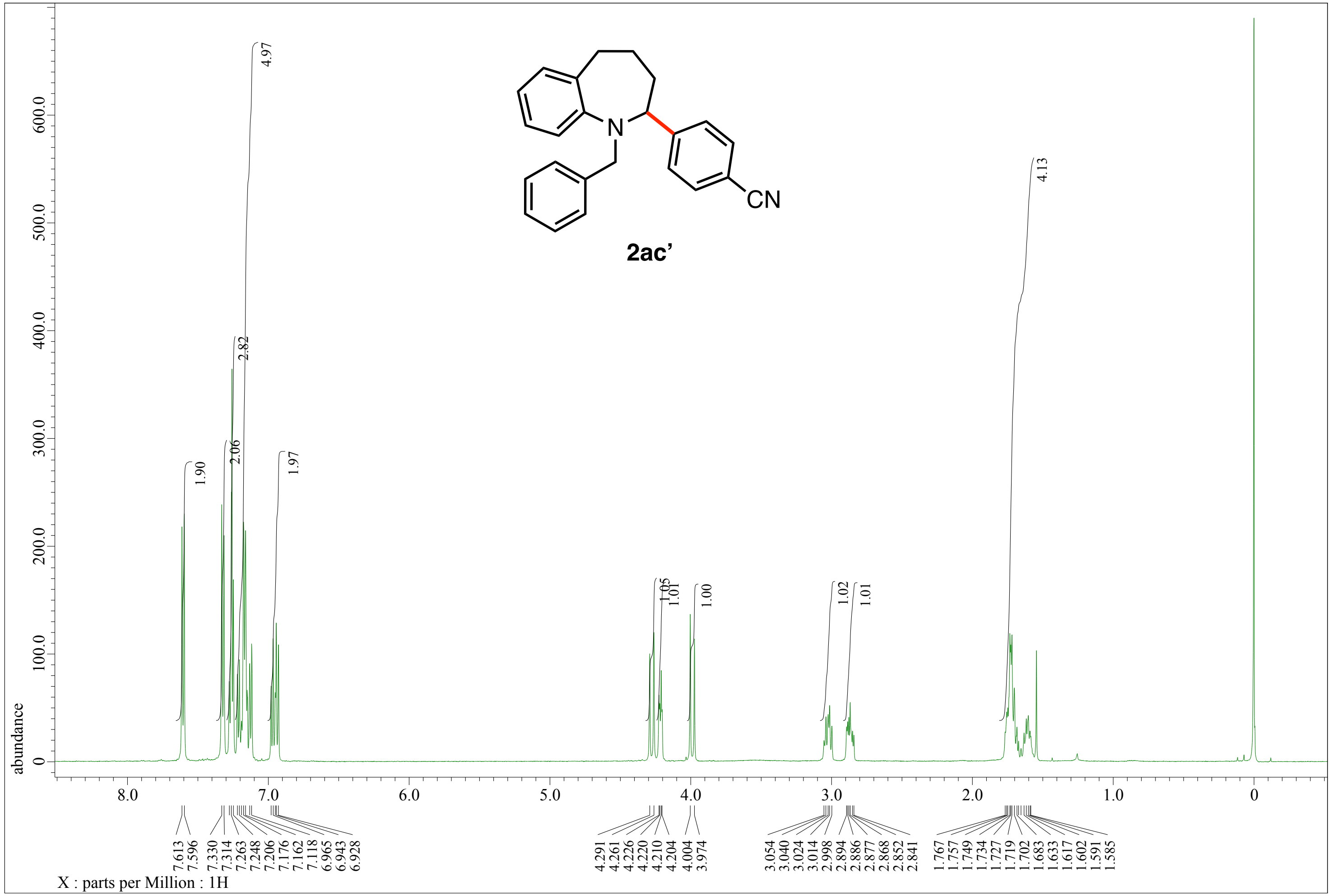
2ac'
CN
N
X : parts per Million : 1H
abundance

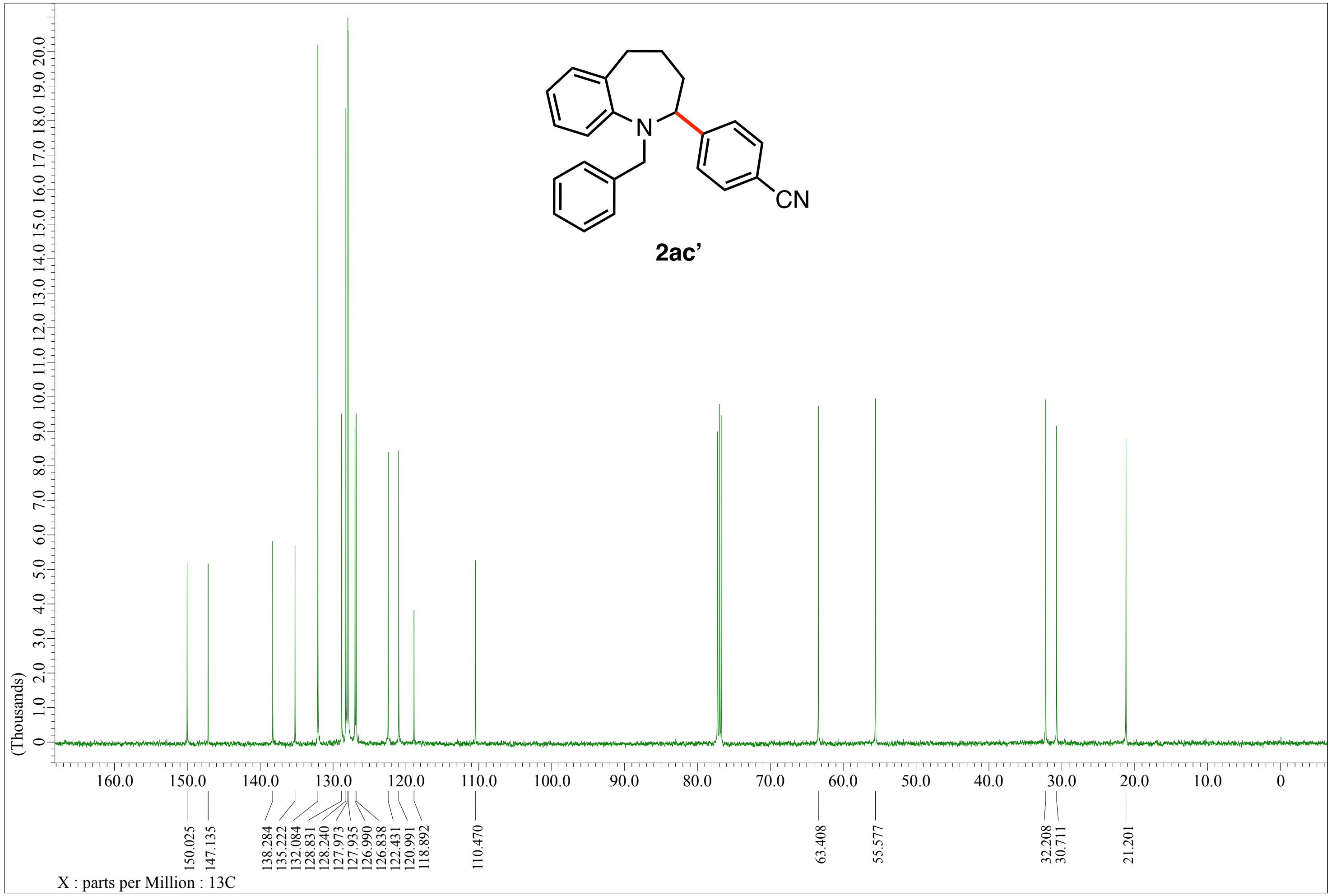

CN
2ac'
(Thousands)
0
1.0
2.0
3.0
4.0
5.0
6.0
7.0
8.0
9.0
10.0
11.0
12.0
13.0
14.0
15.0
16.0
17.0
18.0
19.0
20.0
160.0
150.0
140.0
130.0
120.0
110.0
100.0
90.0
80.0
70.0
60.0
50.0
40.0
30.0
20.0
10.0
0
150.025
147.135
138.284
135.222
132.084
128.831
128.240
127.973
127.935
126.990
126.838
122.431
120.991
118.892
110.470
63.408
55.577
32.208
30.711
21.201
X : parts per Million : 13C

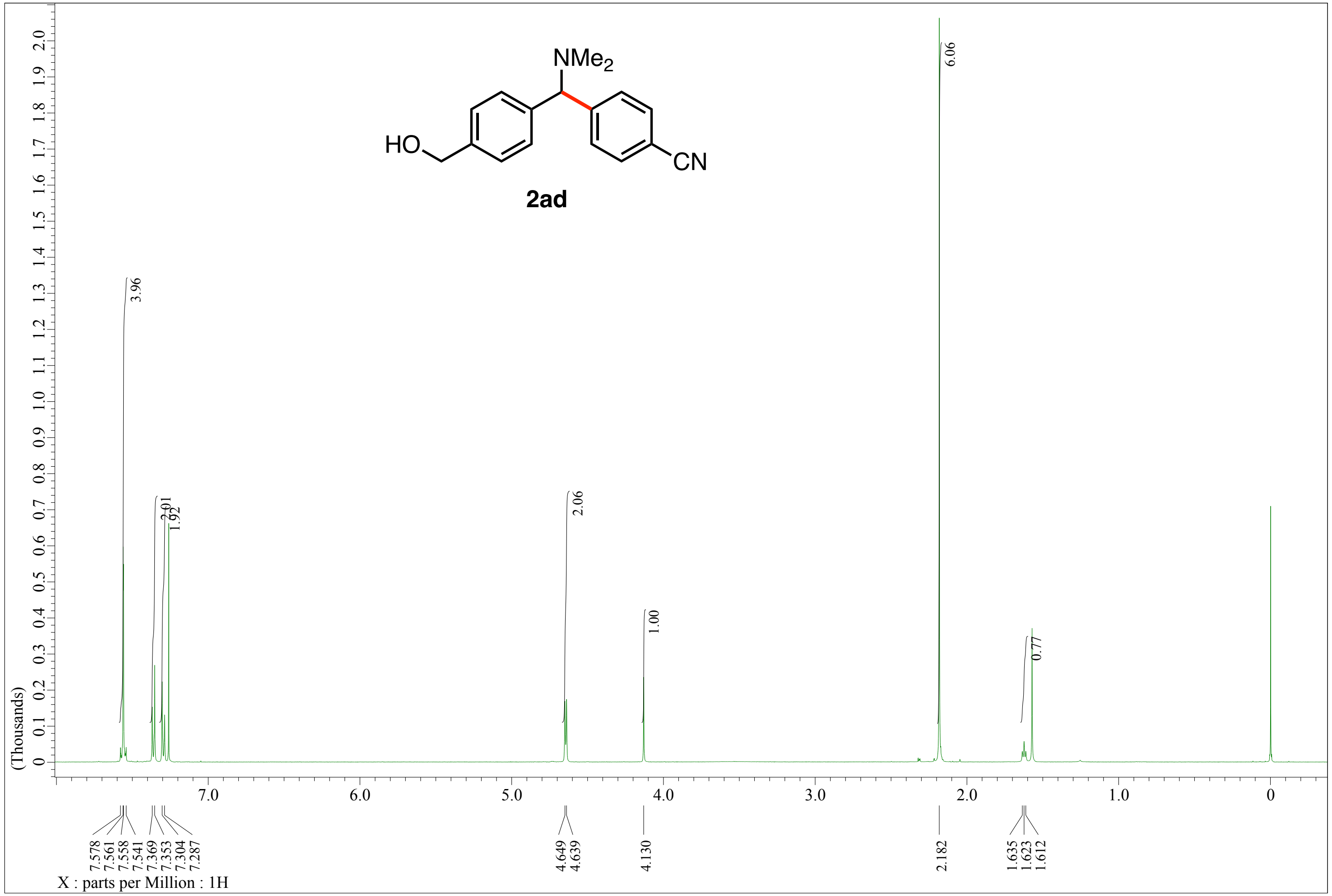
NMe2
HO
CN
2ad
3.96
2.01
1.92
2.06
1.00
6.06
0.77
(Thousands)
7.578
7.561
7.558
7.541
7.369
7.353
7.304
7.287
4.649
4.639
4.130
2.182
1.635
1.623
1.612
X : parts per Million : 1H

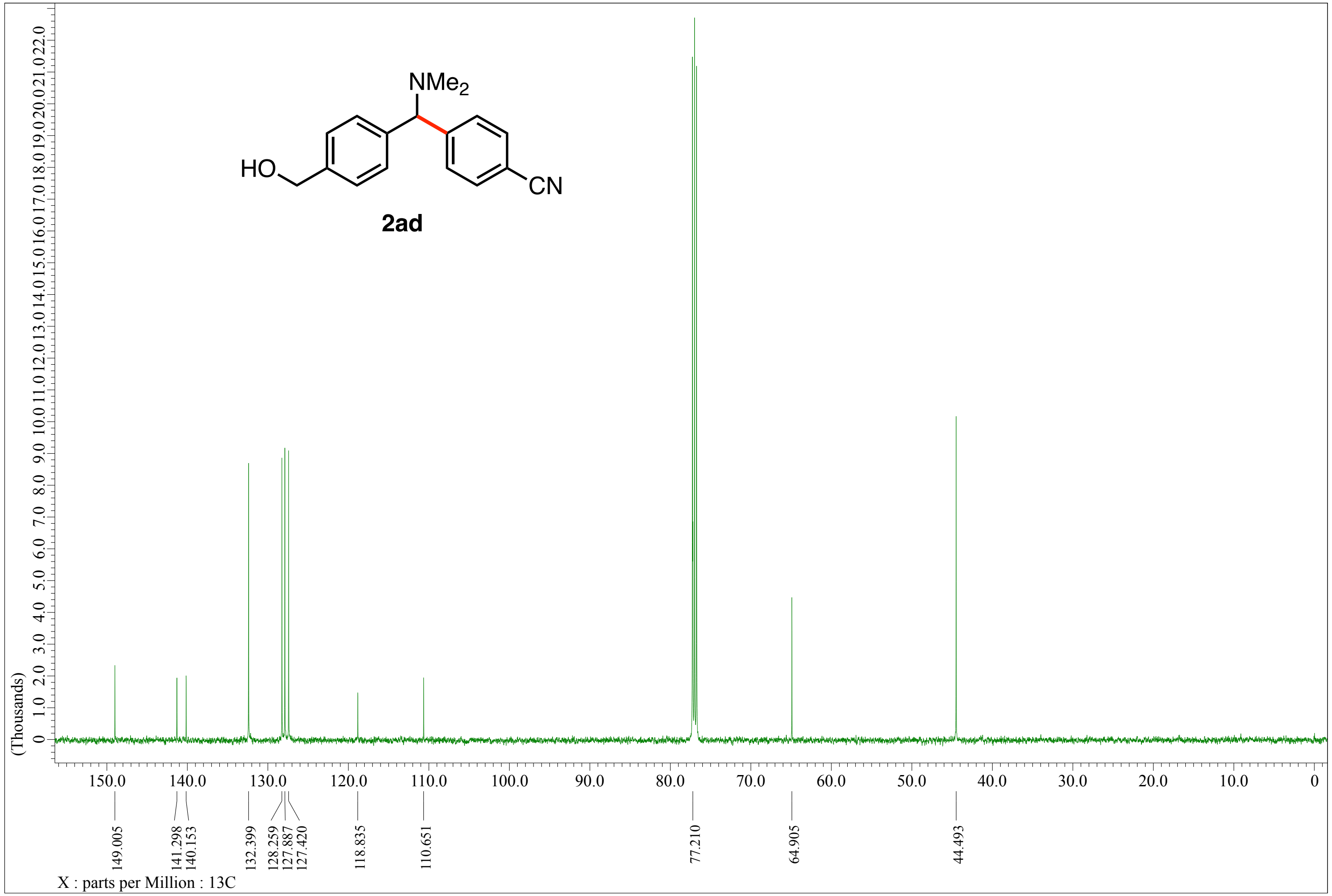
NMe2
HO
CN
2ad
(Thousands)
X : parts per Million : 13C
149.005
141.298
140.153
132.399
128.259
127.887
127.420
118.835
110.651
77.210
64.905
44.493

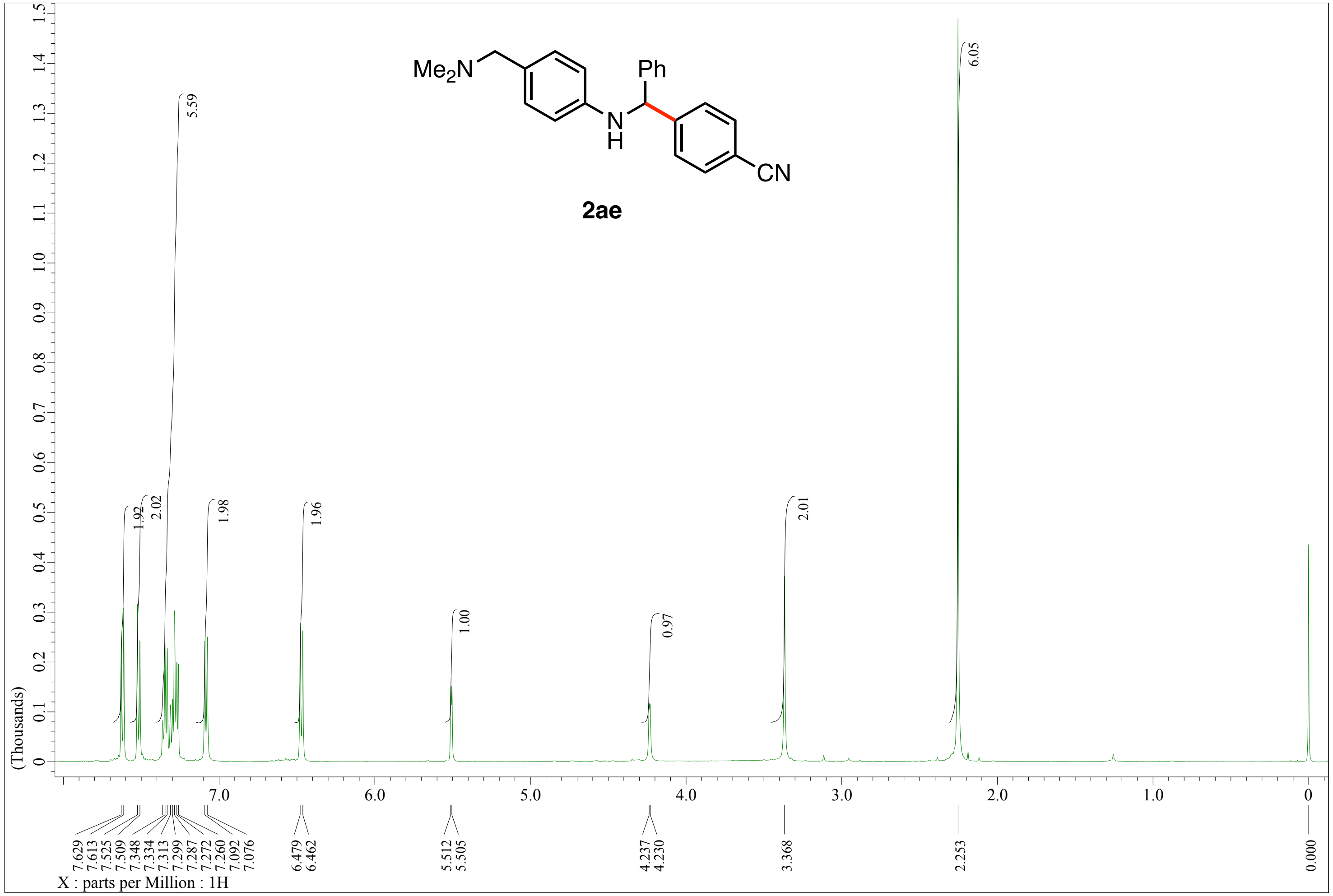
Me2N
Ph
N
H
CN
2ae
(Thousands)
X : parts per Million : 1H
7.629
7.613
7.525
7.509
7.348
7.334
7.313
7.299
7.287
7.272
7.260
7.092
7.076
6.479
6.462
5.512
5.505
4.237
4.230
3.368
2.253
0.000
1.92
2.02
5.59
1.98
1.96
1.00
0.97
2.01
6.05

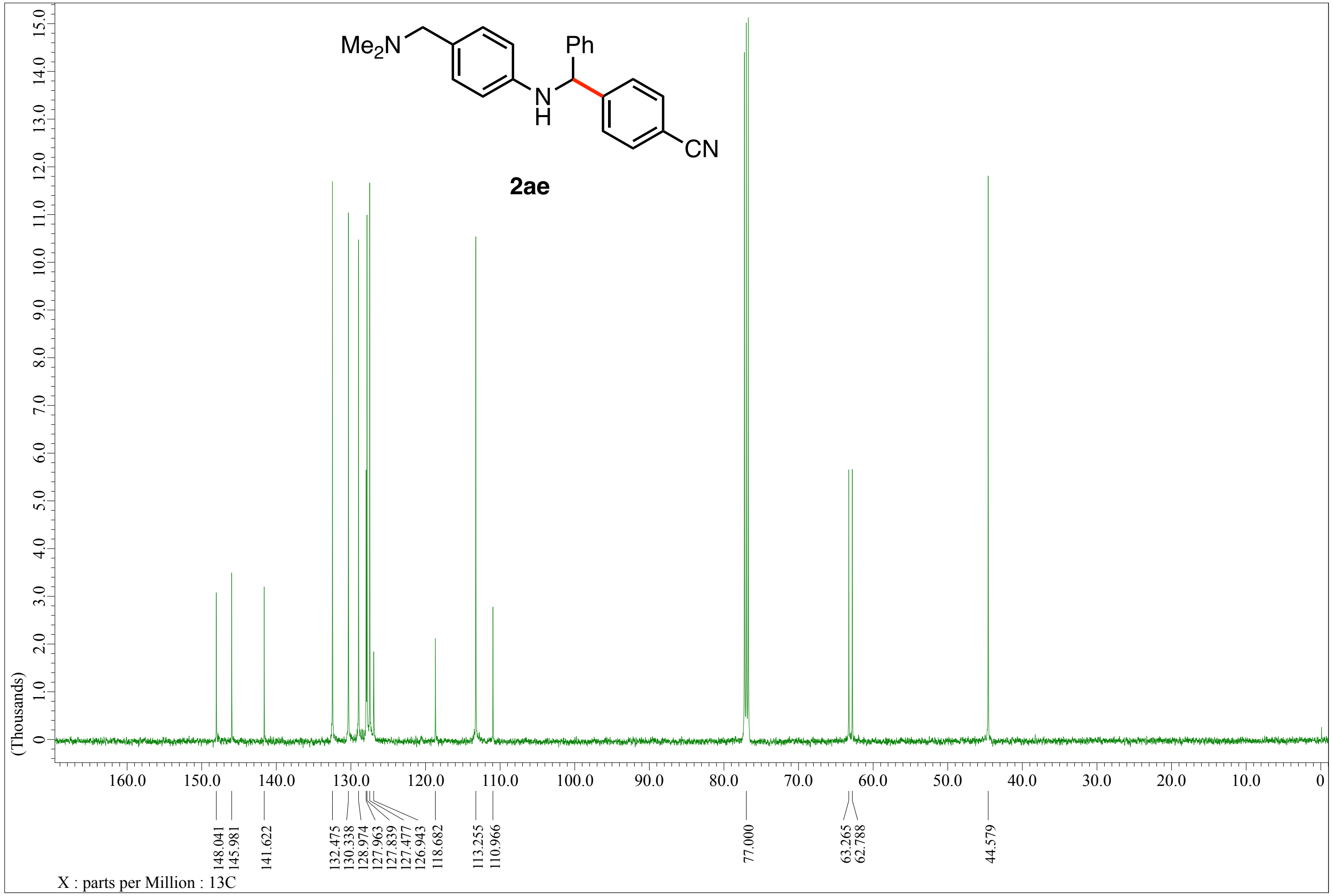
Me2N
Ph
N
H
CN
2ae
(Thousands)
0
1.0
2.0
3.0
4.0
5.0
6.0
7.0
8.0
9.0
10.0
11.0
12.0
13.0
14.0
15.0
160.0
150.0
140.0
130.0
120.0
110.0
100.0
90.0
80.0
70.0
60.0
50.0
40.0
30.0
20.0
10.0
0
148.041
145.981
141.622
132.475
130.338
128.974
127.963
127.839
127.477
126.943
118.682
113.255
110.966
77.000
63.265
62.788
44.579
X : parts per Million : 13C

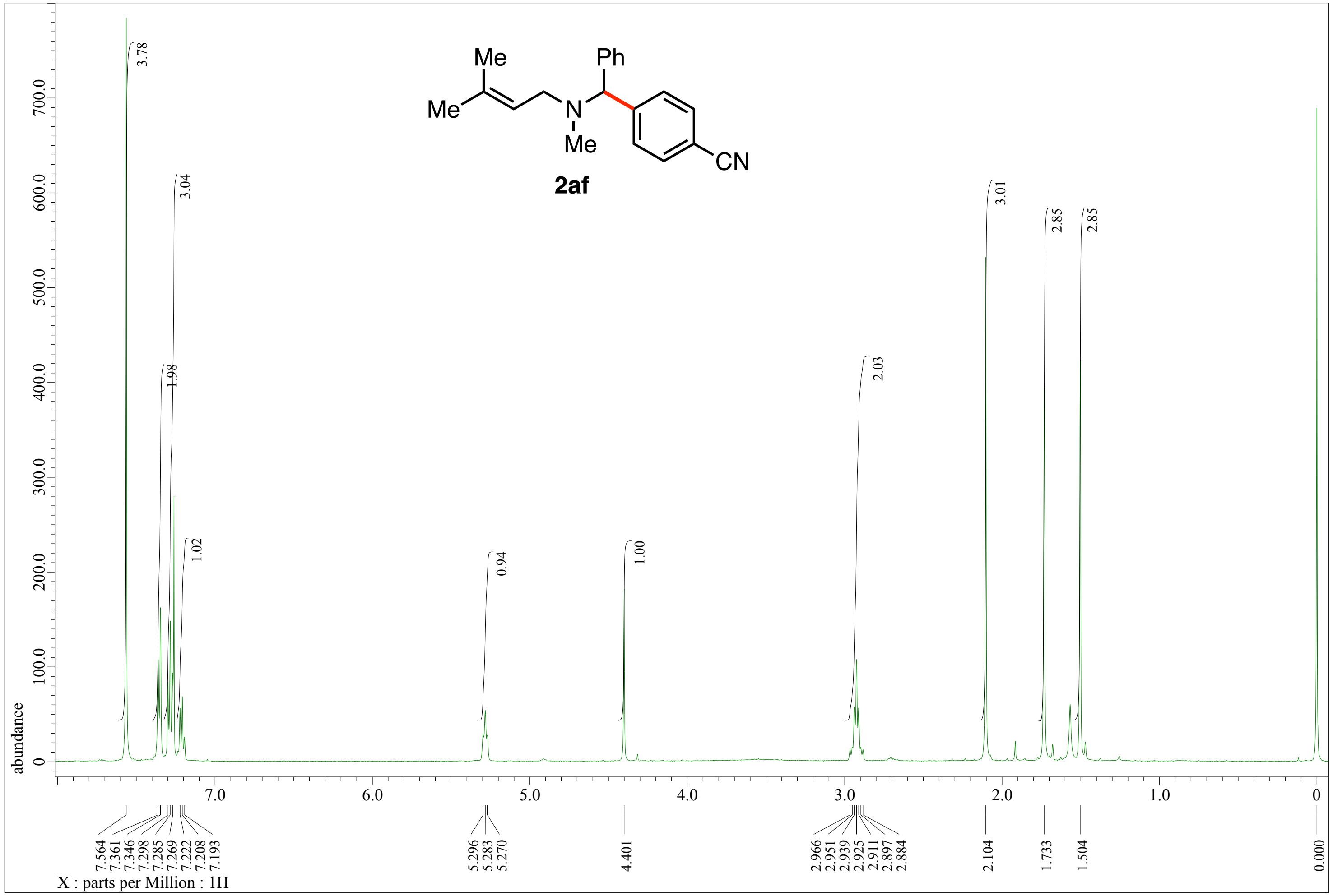

Me
Me
Ph
N
Me
CN
2af
abundance
X : parts per Million : 1H

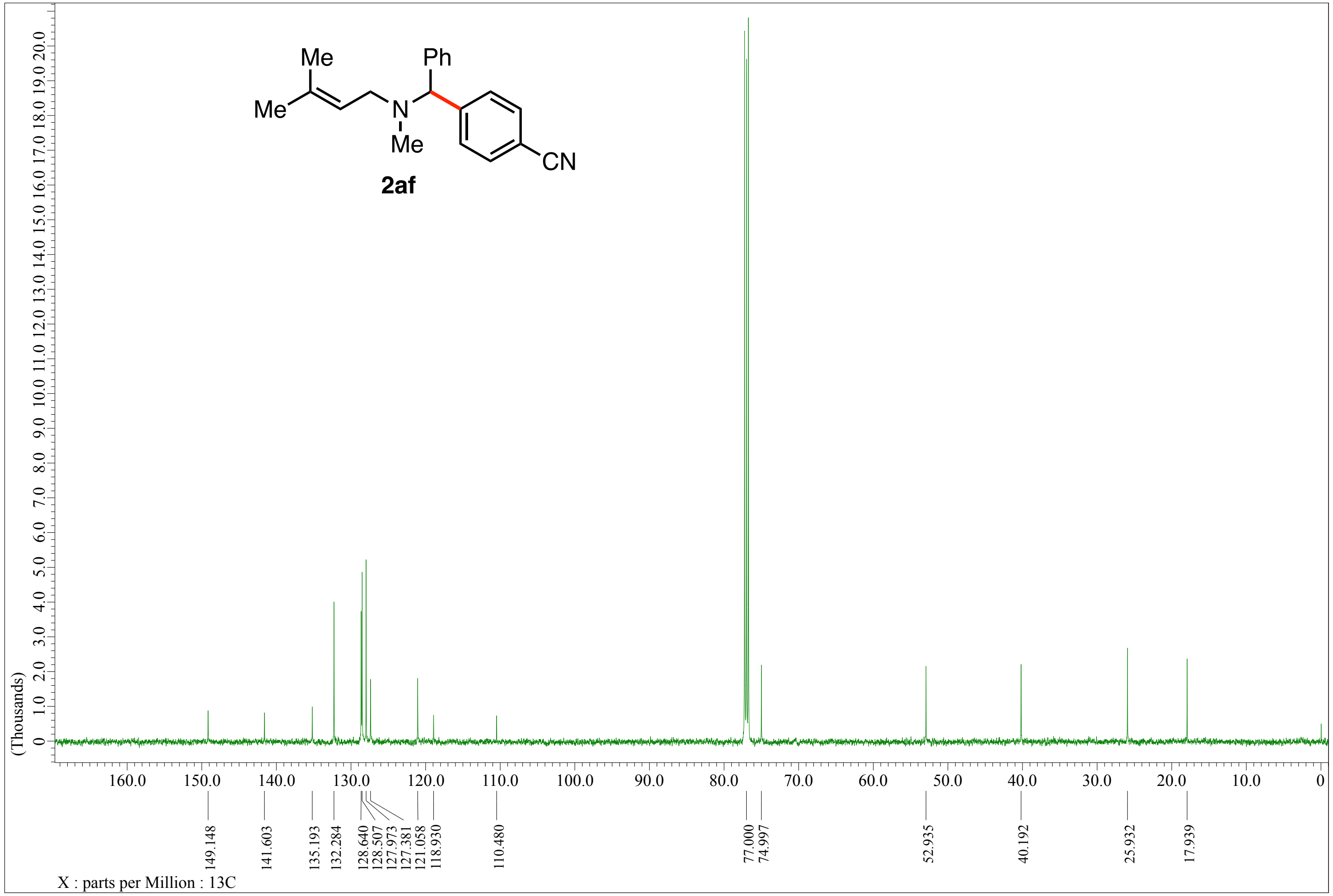
Me
Me
N
Me
Ph
CN
2af
(Thousands)
X : parts per Million : 13C
149.148
141.603
135.193
132.284
128.640
128.507
127.973
127.381
121.058
118.930
110.480
77.000
74.997
52.935
40.192
25.932
17.939

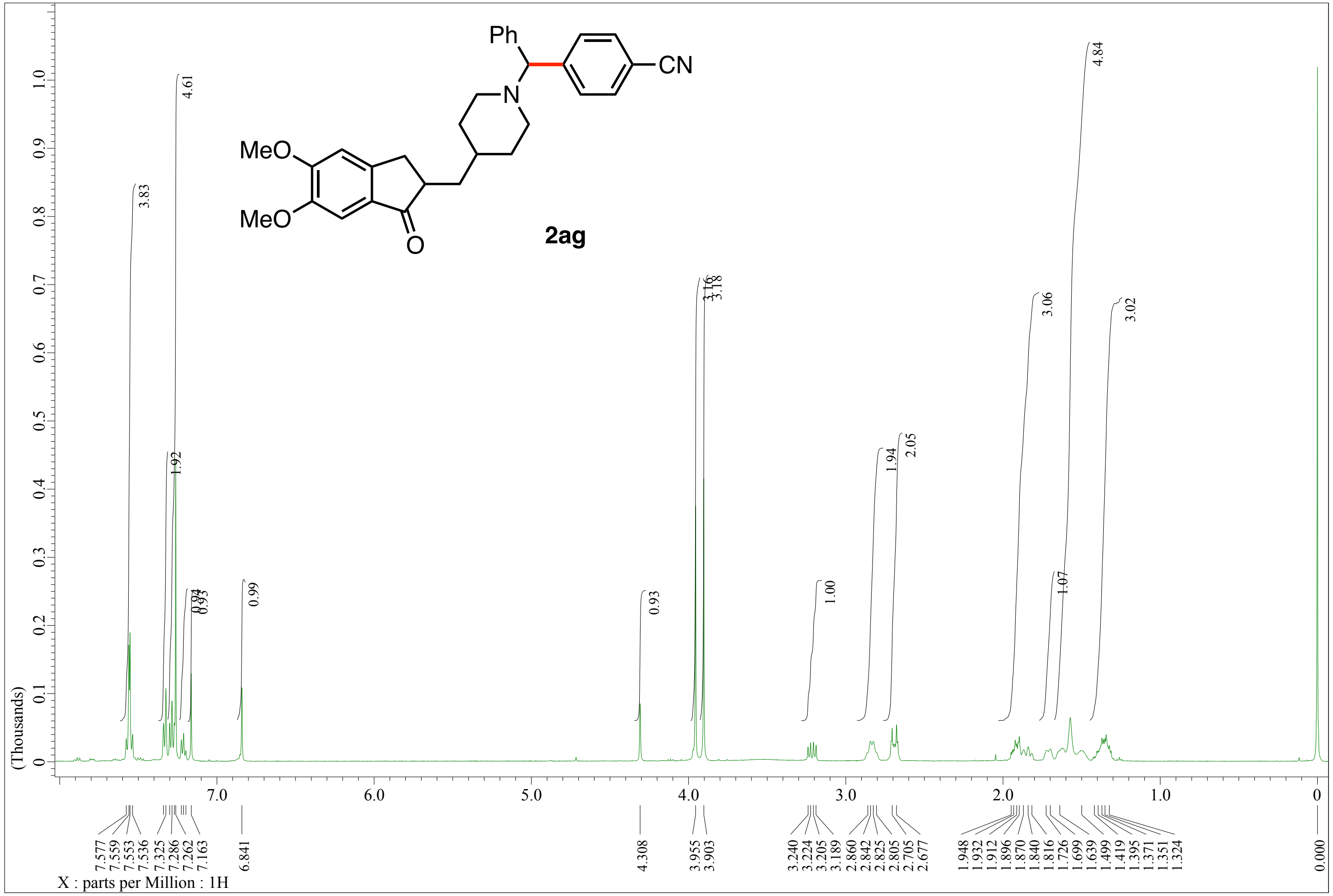

Ph
CN
N
MeO
MeO
O
2ag
(Thousands)
X : parts per Million : 1H
3.83
4.61
1.92
0.94
0.93
0.99
0.93
3.16
3.18
1.00
1.94
2.05
3.06
1.07
4.84
3.02
7.577
7.559
7.553
7.536
7.325
7.286
7.262
7.163
6.841
4.308
3.955
3.903
3.240
3.224
3.205
3.189
2.860
2.842
2.825
2.805
2.705
2.677
1.948
1.932
1.912
1.896
1.870
1.840
1.816
1.726
1.699
1.639
1.499
1.419
1.395
1.371
1.351
1.324
0.000

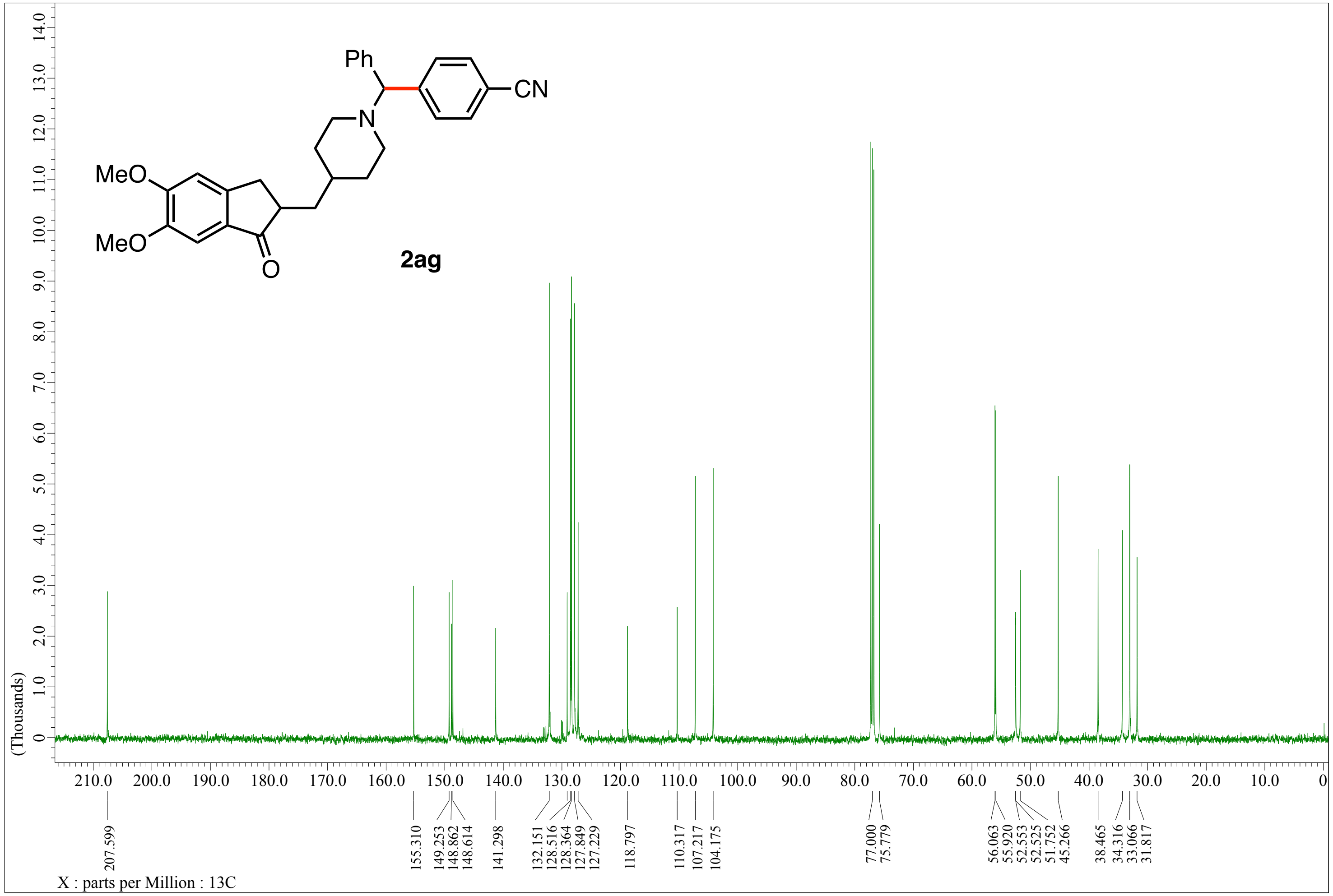

Ph
CN
N
MeO
MeO
O
2ag
(Thousands)
X : parts per Million : 13C
207.599
155.310
149.253
148.862
148.614
141.298
132.151
128.516
128.364
127.849
127.229
118.797
110.317
107.217
104.175
77.000
75.779
56.063
55.920
52.553
52.525
51.752
45.266
38.465
34.316
33.066
31.817

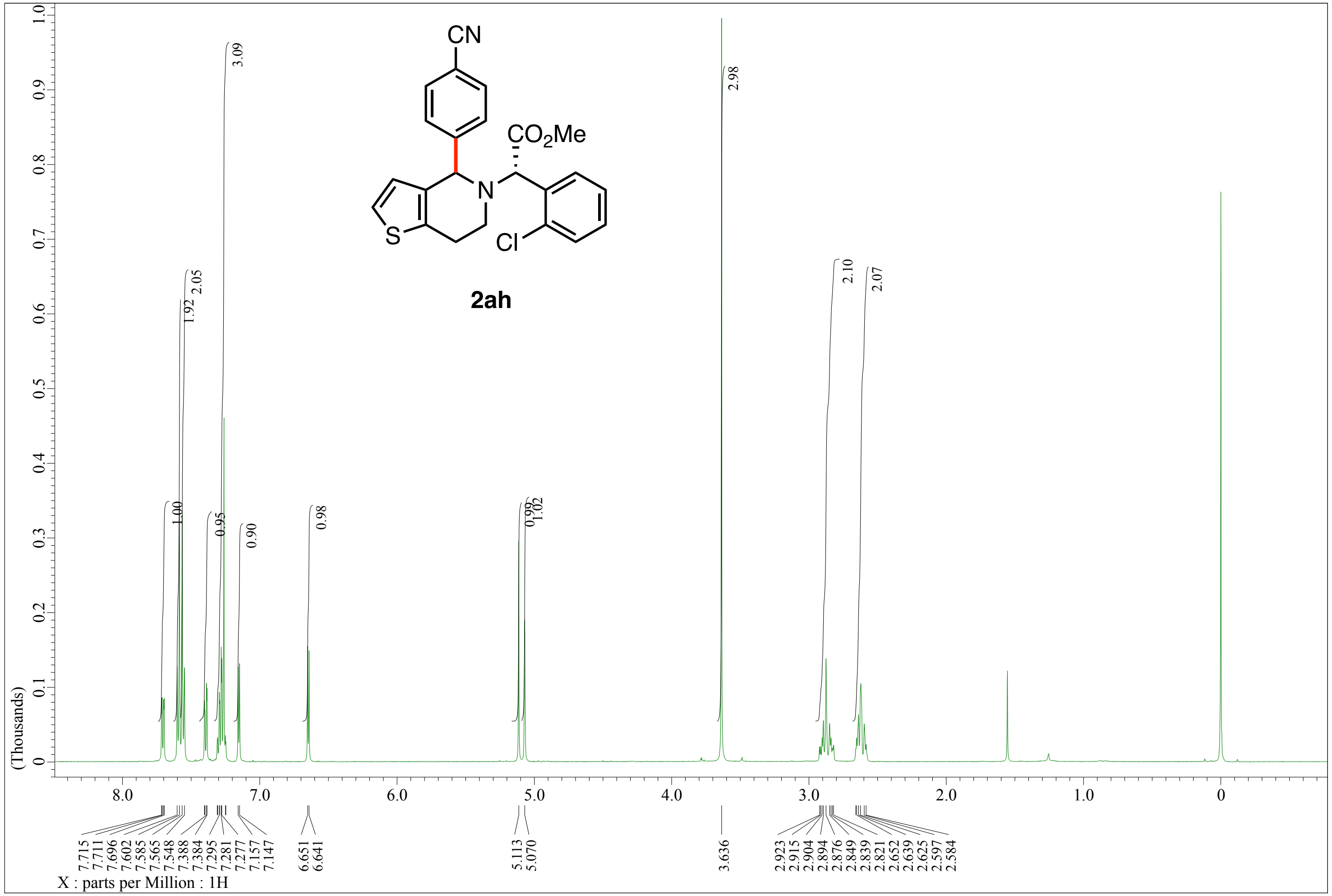
CN
CO2Me
N
S
Cl
2ah
(Thousands)
X : parts per Million : 1H
7.715
7.711
7.696
7.602
7.585
7.565
7.548
7.388
7.384
7.295
7.281
7.277
7.157
7.147
6.651
6.641
5.113
5.070
3.636
2.923
2.915
2.904
2.894
2.876
2.849
2.839
2.821
2.652
2.639
2.625
2.597
2.584

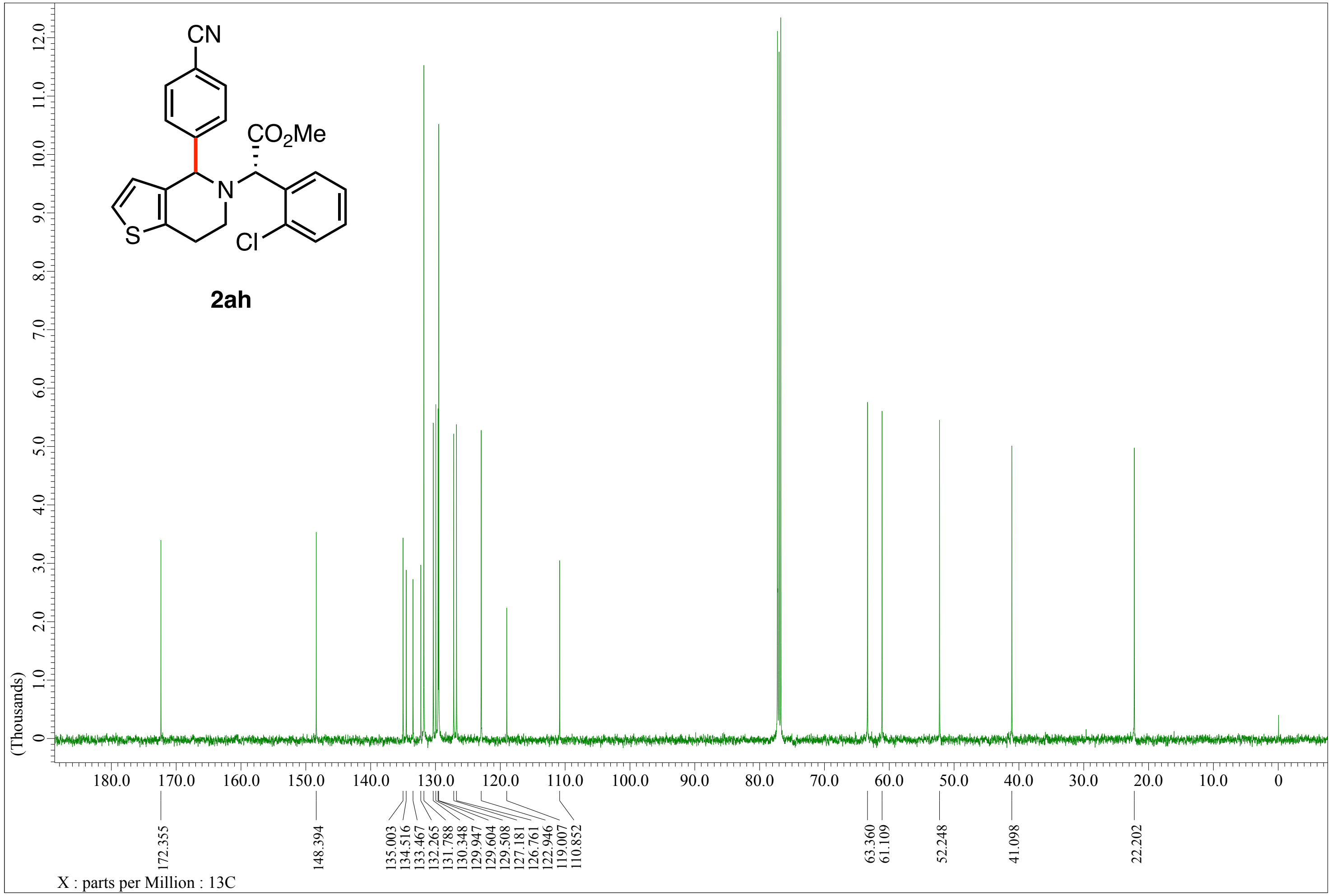
CN
CO2Me
N
S
Cl
2ah
(Thousands)
X : parts per Million : 13C
172.355
148.394
135.003
134.516
133.467
132.265
131.788
130.348
129.947
129.604
129.508
127.181
126.761
122.946
119.007
110.852
63.360
61.109
52.248
41.098
22.202

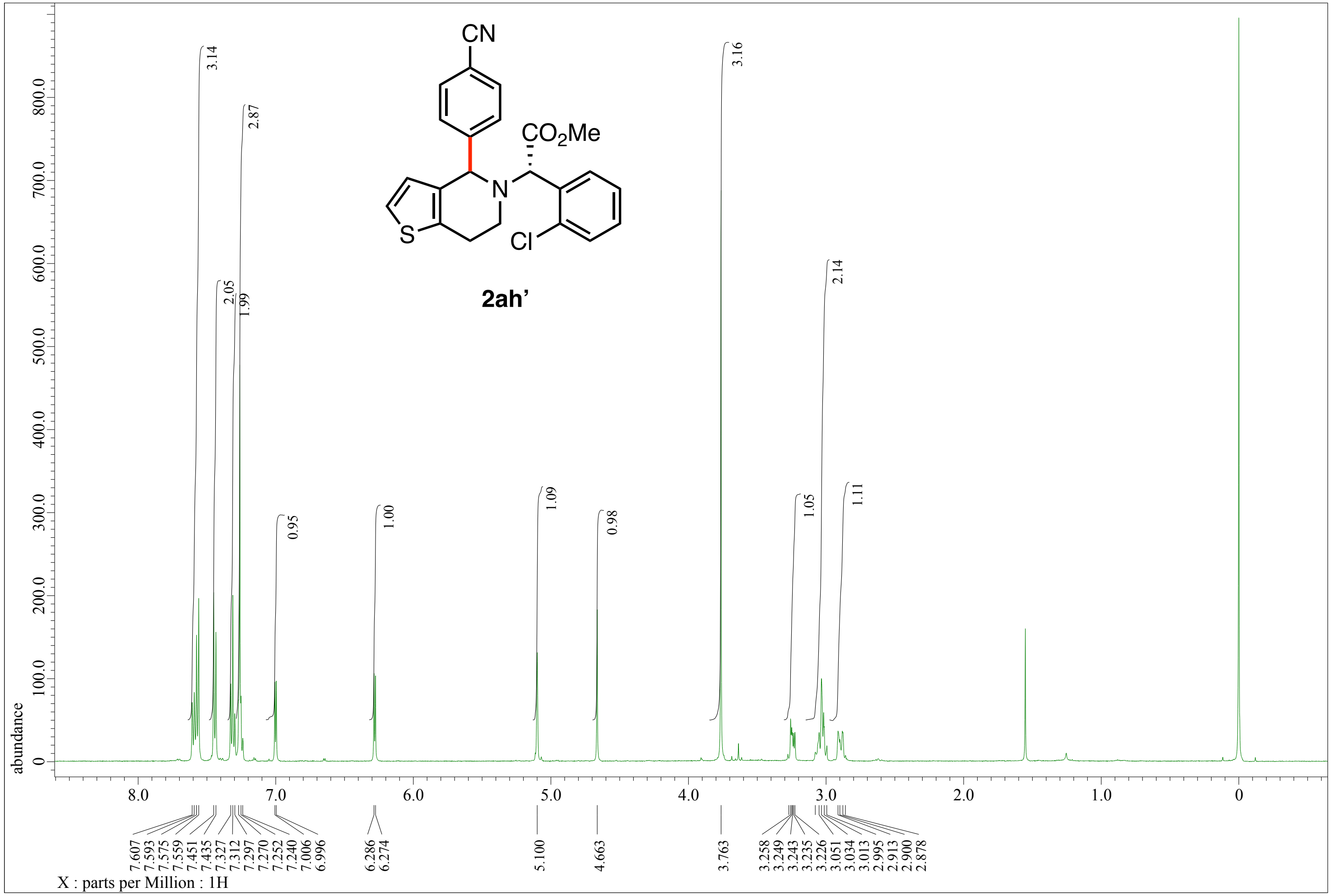
CN
CO2Me
N
S
Cl
2ah'
abundance
X : parts per Million : 1H
3.14
2.05
1.99
2.87
0.95
1.00
1.09
0.98
3.16
1.05
2.14
1.11
7.607
7.593
7.575
7.559
7.451
7.435
7.327
7.312
7.297
7.270
7.252
7.240
7.006
6.996
6.286
6.274
5.100
4.663
3.763
3.258
3.249
3.243
3.235
3.226
3.051
3.034
3.013
2.995
2.913
2.900
2.878

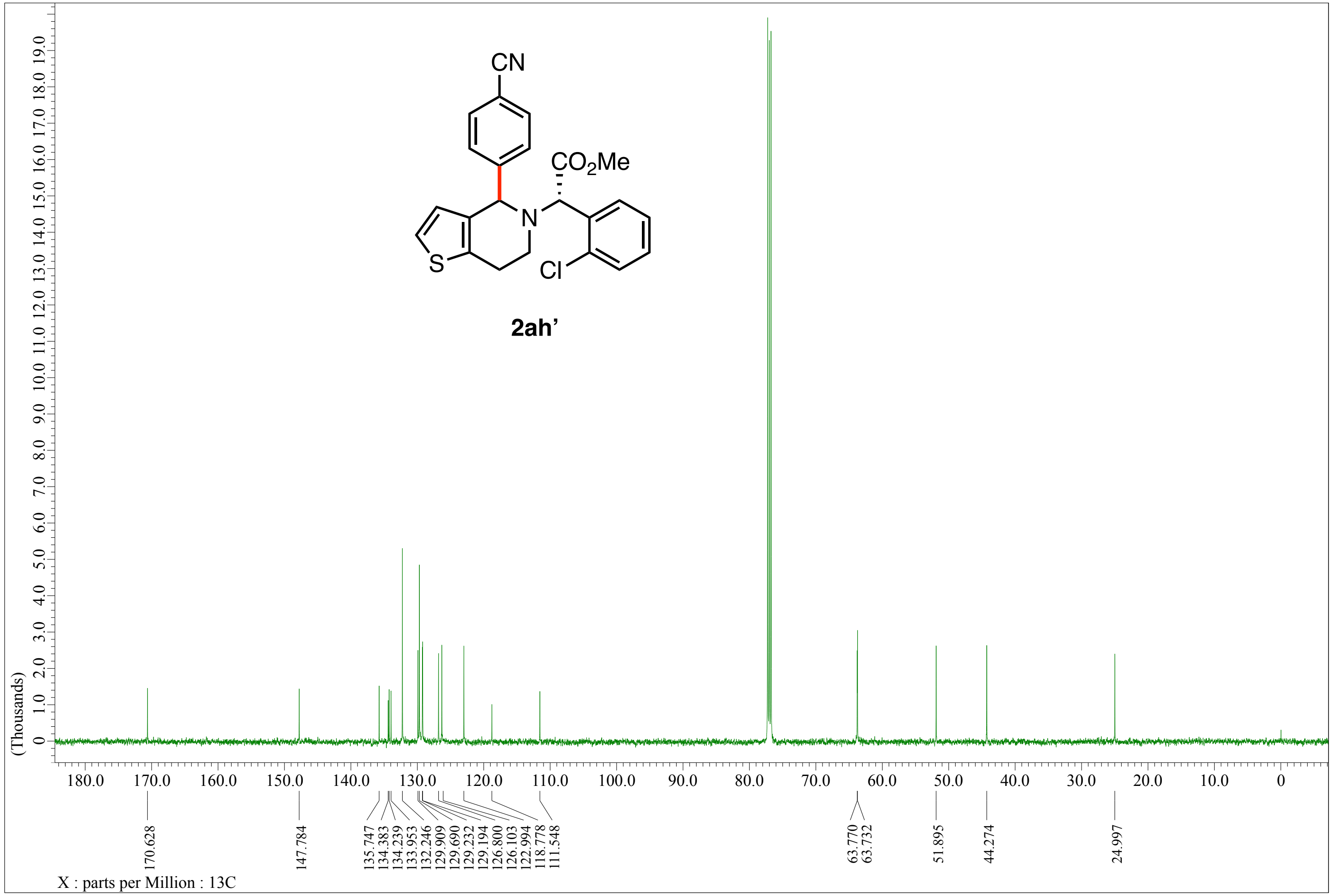

CN
CO2Me
N
S
Cl
2ah'
(Thousands)
X : parts per Million : 13C
170.628
147.784
135.747
134.383
134.239
133.953
132.246
129.909
129.690
129.232
129.194
126.800
126.103
122.994
118.778
111.548
63.770
63.732
51.895
44.274
24.997

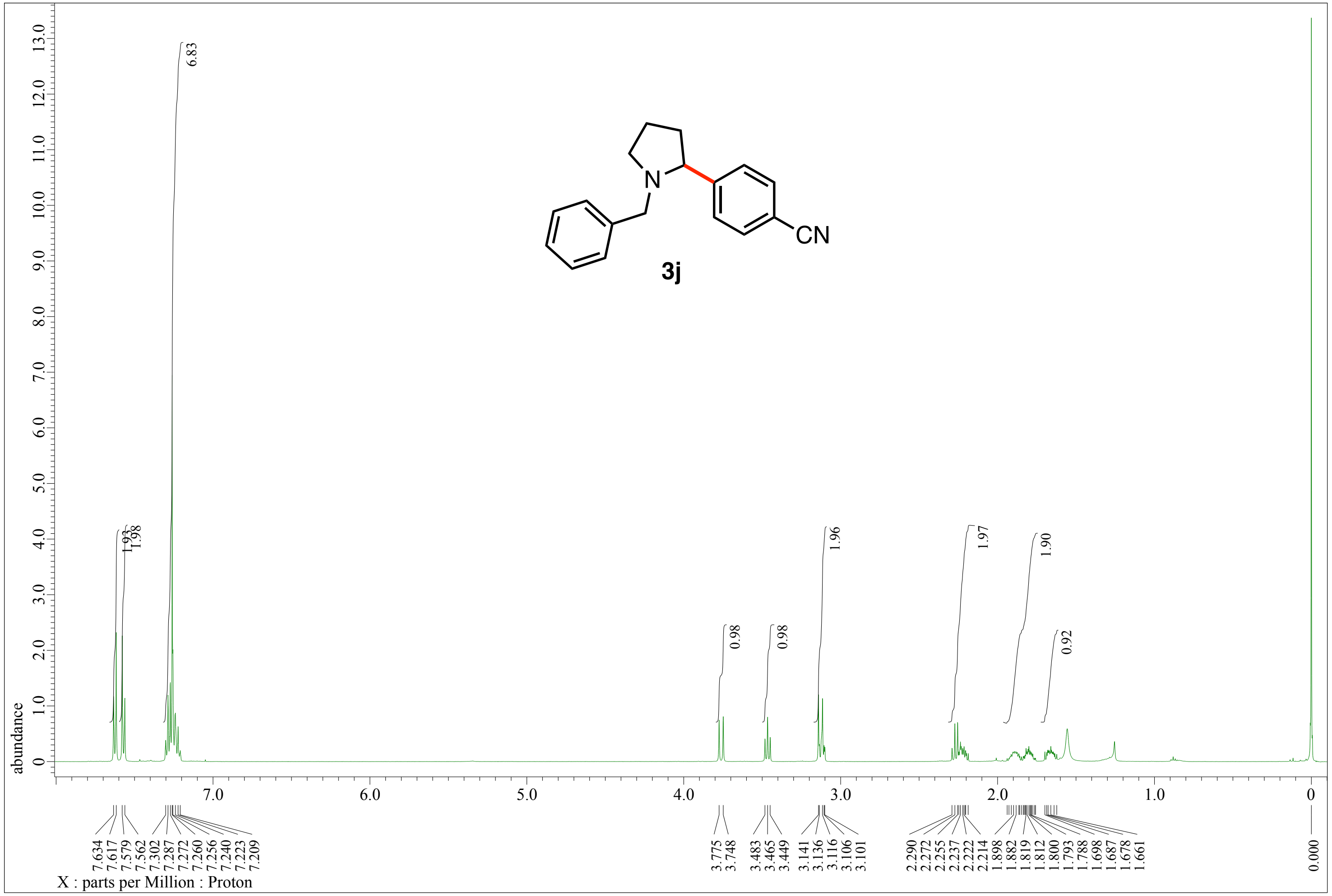

CN
3j
abundance
X : parts per Million : Proton
6.83
1.93
1.98
0.98
0.98
1.96
1.97
1.90
0.92
7.634
7.617
7.579
7.562
7.302
7.287
7.272
7.260
7.256
7.240
7.223
7.209
3.775
3.748
3.483
3.465
3.449
3.141
3.136
3.116
3.106
3.101
2.290
2.272
2.255
2.237
2.222
2.214
1.898
1.882
1.819
1.812
1.800
1.793
1.788
1.698
1.687
1.678
1.661
0.000

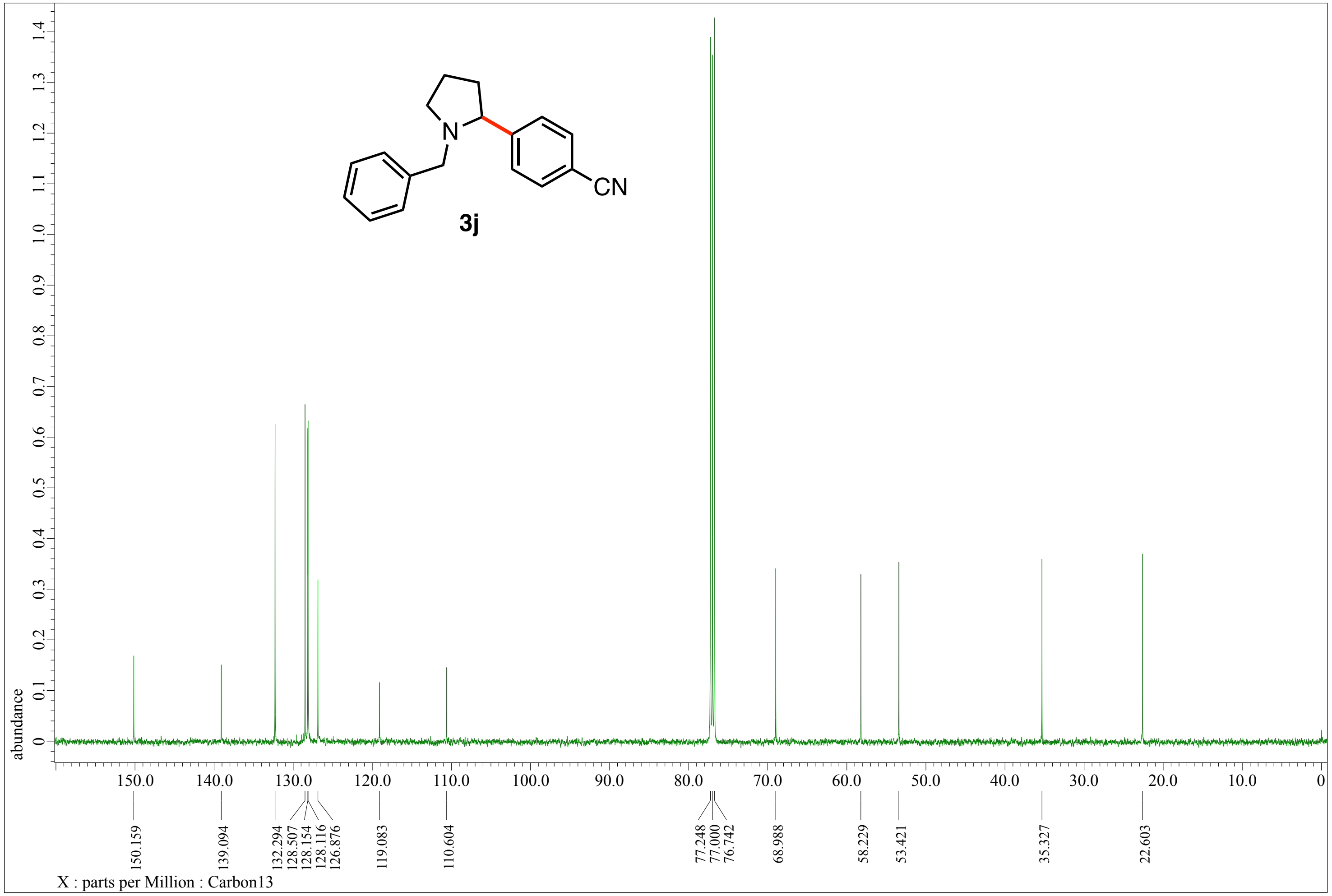

N
CN
3j
abundance
0
0.1
0.2
0.3
0.4
0.5
0.6
0.7
0.8
0.9
1.0
1.1
1.2
1.3
1.4
150.0
140.0
130.0
120.0
110.0
100.0
90.0
80.0
70.0
60.0
50.0
40.0
30.0
20.0
10.0
0
150.159
139.094
132.294
128.507
128.154
128.116
126.876
119.083
110.604
77.248
77.000
76.742
68.988
58.229
53.421
35.327
22.603
X : parts per Million : Carbon13
